# Glucose-induced histone lactylation confers metformin resistance in colorectal cancer stem cells

**DOI:** 10.1038/s41467-026-77051-9

**Published:** 2026-09-07

**Authors:** Xiang-Rui Li, Li Deng, Tian-Ge Zhang, Xin-De Chen, Yun-Han Hu, He-Yang Zhang, Shi-Qi Lin, Zhao-Ting Bu, Hong Zhao, Xiao-Yue Liu, Yue Chen, Zhi-Jian Zhang, Xiang Tao, Min Tang, Tao Huang, Yu-Shu Huang, Yang Zhang, Zhi-Hao Wu, Han-Ping Shi

**Affiliations:** 1 Department of Gastrointestinal Surgery, Department of Clinical Nutrition, Beijing Shijitan Hospital, Capital Medical University, Beijing, 100038 China; 2 Key Laboratory of Cancer FSMP for State Market Regulation, Beijing, 100038 China; 3 Laboratory for Clinical Medicine, Capital Medical University, Beijing, 100038 China; 4 Research laboratory of Tumor Microenvironment, Wannan Medical College, Wuhu, 241001 China; 5 Anhui Province Key laboratory of Basic Research and Transformation of Age-related Diseases, Wannan Medical College, Wuhu, 241001 China; 6 Clinical Medical Experimental Training Center, Wannan Medical College, Wuhu, 241001 China; 7 Institute of Clinical Nutrition, The First Affiliated Hospital of Wenzhou Medical University, Wenzhou, Zhejiang 325000 China

**Keywords:** Cancer metabolism, Cancer stem cells

## Abstract

Cancer stem cells (CSCs) can adapt their metabolism, which limits the effectiveness of therapies targeting metabolic pathways. Here we show that high glucose conditions promote metformin resistance in CD133⁺ colorectal CSCs by driving a switch from glucose dependence to lipid use. Mechanistically, metformin increases LDHA-dependent lactate production in these cells under high glucose conditions. Lactate accumulation enhances histone H3 lysine 18 lactylation (H3K18la), which activates c-JUN and induces CD36 expression. CD36 promotes free fatty acid (FFA) uptake and lipid droplet (LD) formation, providing fuel for fatty acid β-oxidation. This process generates ATP and NADPH, reduces energy stress and reactive oxygen species, and thereby supports CSC survival during metformin treatment. In vitro and in vivo, inhibition of either LDHA or CD36 restores metformin sensitivity in CD133⁺ colorectal CSCs. These findings identify a lactate-driven epigenetic and metabolic pathway underlying metformin resistance and suggest LDHA and CD36 as potential therapeutic targets.

## Abstract (web summary)

Metformin has been proposed as a therapy for cancer, but many reports are showing limited efficacy. Here, the authors show that in high-glucose conditions, histone lactylation induces fatty acid uptake leading to metformin resistance in colorectal cancer stem cells.

## Introduction

Metformin is a widely used biguanide anti-diabetic drug, favored in clinical practice due to its excellent safety profile and effective regulation of systemic blood glucose levels^1^. Numerous studies have demonstrated that metformin exhibits anticancer activity both in vitro and in vivo, primarily through inhibition of mitochondrial complex I, leading to reduced cancer cell proliferation^2^. However, the sensitivity of cancer cells to metformin varies significantly, with some cells adapting and resisting its effects by altering their metabolic pathways, such as enhancing glycolysis, glutamine metabolism, branched-chain amino acid oxidation, and asparagine synthesis^3–6^.

As a major source of exogenous metabolic substrates, diet can significantly influence the nutritional landscape and metabolic pathways within the tumor microenvironment, thereby potentially affecting cancer cell sensitivity to metabolic drugs, including metformin. In modern society, high-carbohydrate diets have become a common nutritional pattern, characterized by elevated glycemic index (GI) and glycemic load (GL), with a substantial portion of caloric intake derived from easily digestible carbohydrates such as glucose^7,8^. This dietary pattern may impact tumor metabolism by continuously supplying glucose. However, systematic studies evaluating whether high-carbohydrate diets interfere with the antitumor efficacy of metformin are currently lacking.

Cancer stem cells (CSCs) are notorious for their high heterogeneity and plasticity, enabling them to metastasize, resist chemotherapy, and adapt to the constantly changing cancer microenvironment, significantly complicating cancer eradication^9^. Despite research indicating that some CSCs rely on oxidative phosphorylation (OXPHOS), making them potential targets for biguanide drugs^10–13^, these CSCs can develop resistance through metabolic reprogramming. In pancreatic ductal adenocarcinoma (PDAC), a small subset of CD133⁺ cells exhibit low mitochondrial mass and rely on glycolytic metabolism, rendering them resistant to metformin^11^. The presence of these cells in PDAC may explain the observed tumor recurrence in mouse models and provide an alternative explanation for the negative outcomes observed in the initial clinical trials of metformin in PDAC patients^14^. Additionally, studies have reported that stem cell-like cancer cells utilize nicotinamide adenine dinucleotide (NADH)-dependent C-terminal binding protein (CtBP) transcriptional regulators to maintain high glycolytic levels, thereby counteracting the effects of metformin^15^. Lactate is a key metabolic byproduct generated during metformin-induced glycolytic reprogramming and exerts multiple biological functions. Recent studies have identified lactate’s significant role in cancer resistance, promoting DNA damage repair and chemoresistance^16,17^. However, it remains unclear whether lactate plays a mediating role in CSC resistance to metformin.

In this study, we found that a high-carbohydrate diet attenuates the anticancer efficacy of metformin against colorectal cancer (CRC), with CD133⁺ CSCs identified as key mediators of this resistance. Mechanistically, the high-carbohydrate diet provides abundant glucose to cancer cells, which, under metformin treatment, induces metabolic reprogramming in CD133⁺ CSCs, leading to excessive lactate production. Accumulated lactate promotes histone lactylation, which in turn activates the expression of the transcription factor c-JUN, subsequently upregulating the fatty acid transporter CD36. Previous studies have shown that CD36 plays a critical role in CSC-associated drug resistance^18–20^. CD36-mediated lipid droplet (LD) metabolic reprogramming not only supplies ATP to CSCs but also counteracts reactive oxygen species (ROS), thereby enabling CSCs to survive under metabolic stress. Importantly, targeting LDHA or CD36 was found to reverse this resistance. This study reveals a mechanism by which dietary composition modulates tumor metabolism and interferes with the anticancer activity of metformin, providing insight into the metabolic basis of metformin resistance and identifying potential targets for therapeutic intervention.

## Results

### A high-carbohydrate diet attenuates the antitumor effect of metformin in CRC

**Fig. 1:**
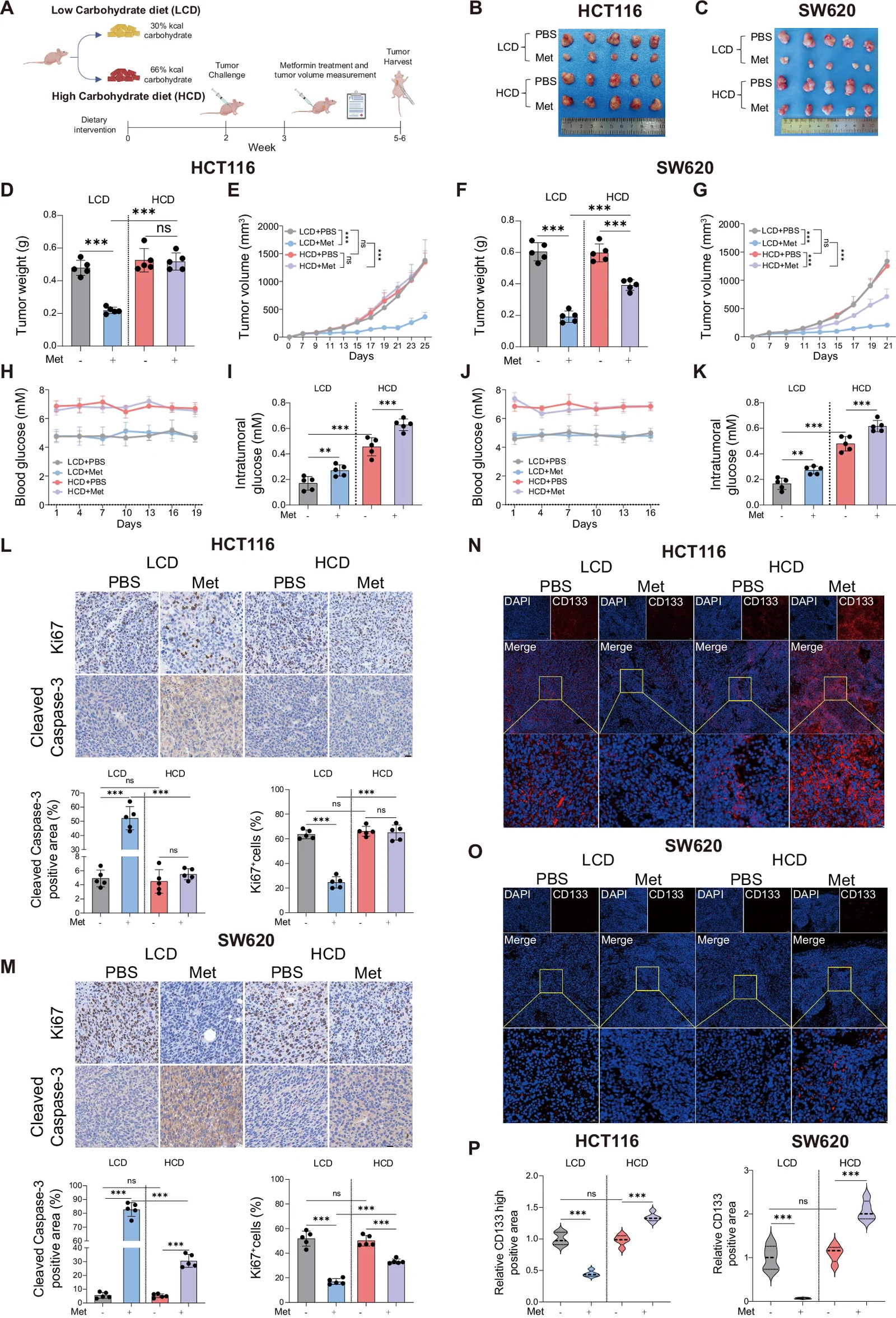
A high-carbohydrate diet attenuates the antitumor effect of metformin in CRC. **A** Schematic illustration of CRC tumor model establishment in mice fed an HCD or LCD and the corresponding Met treatment regimen. Mice received intraperitoneal injections of Met at a dose of 200 mg/kg every two days, and tumor measurements were recorded at the same interval. Created in BioRender. Li, X. (2026) https://BioRender.com/2qboh1g. Representative tumors are shown in **B** and **C**; tumor weights are quantified in **D** and **F**; and growth curves are shown in **E** and **G**. Random (non-fasted) blood glucose was monitored every 3 days by tail-vein sampling (**H**, **J**), and intratumoral glucose was quantified at the experimental endpoint (**I**, **K**). **L**, **M** Immunohistochemical staining of tumor tissues for Cleaved Caspase-3 and Ki67 expression, with quantitative analysis presented. Immunofluorescence staining of CD133 in HCT116 (**N**) and SW620 (**O**) tumors, with quantification of relative CD133-high positive area in **P**. Data are presented as mean values ± SD from *n* = 5 mice per group. Statistical significance was determined by two-sided two-way ANOVA followed by Tukey’s multiple-comparison test for **D**, **F**, **I**, **K**, **L**, **M** and **P**. Tumor growth curves in **E** and **G** were analyzed by ordinary two-way ANOVA followed by Tukey’s multiple-comparison test. Scale bars, 20 μm for immunohistochemical images (**L**, **M**) and immunofluorescence images (**N**, **O**). HCD, high-carbohydrate diet; LCD, low-carbohydrate diet; Met, metformin. ns, no significance; *P < 0.05; **P < 0.01; ***P < 0.001. Source data are provided as a Source Data file.

We initially observed that metformin significantly inhibited the growth of HCT116- and SW620-derived CRC xenografts under a low-carbohydrate diet (LCD) in xenograft mouse models. However, this antitumor effect was markedly diminished under a high-carbohydrate diet (HCD), and completely abrogated in the HCT116 model (Fig. 1A–G). Although peripheral blood glucose values in both dietary groups remained within the physiological range, HCD-fed mice exhibited significantly higher peripheral glucose and higher intratumoral glucose concentrations than LCD-fed mice (Fig. 1H–K). Notably, metformin did not reduce peripheral blood glucose in either dietary group; instead, it further increased intratumoral glucose, consistent with previous findings^21^. Because our initial measurements captured random glucose levels that might not coincide with the pharmacodynamic window of metformin, and given that the drug’s half-life in mice is approximately 2.7 h^22^, we additionally assessed glucose in peripheral blood and tumor tissue at 3 and 6 h post-dose. Metformin again failed to lower peripheral glucose in either diet, and intratumoral glucose in HCD-fed mice remained consistently higher than in LCD-fed mice at both time points (Figure S1A–S1D). Immunohistochemistry showed that in both HCT116 and SW620 xenografts, metformin robustly suppressed the proliferation marker Ki67 and induced the apoptotic marker Cleaved Caspase-3 under LCD conditions. Under HCD, these effects were markedly attenuated in HCT116 tumors, whereas in SW620 tumors they remained detectable but with weaker pro-apoptotic activity and less pronounced inhibition of proliferation compared with LCD (Fig. 1L, M).

To determine whether this dietary modulation persists in an immunocompetent setting, we employed a CT26 syngeneic CRC model in BALB/c mice and treated animals under LCD or HCD conditions. Mirroring the xenograft data, HCD markedly blunted metformin efficacy and was accompanied by an increased proportion of CD133⁺ cancer cells (Fig. S1E–S1L), indicating that glucose-dependent resistance also arises in immunocompetent hosts.

To further validate the contribution of elevated glucose availability to metformin resistance, we used an additional model in which mice received 30% glucose (D30) drinking water for 3 weeks prior to tumor inoculation to systemically raise blood glucose (Fig. S2A). Consistent with the above, metformin significantly inhibited tumor growth and promoted apoptosis in control mice but failed to do so in D30-treated mice (Fig. S2B–S2E). Given the frequent involvement of CSCs in therapeutic resistance, we examined CD133 and found a marked enrichment of CD133⁺ cells following metformin treatment in both HCD-fed and D30-treated groups (Fig. 1N–P, S1M, S2F). These findings suggest that increased glucose availability may compromise the therapeutic efficacy of metformin in CRC by promoting the enrichment of CD133⁺ CSCs.

### CD133^+^ colorectal CSCs exhibit resistance to metformin under high-glucose conditions

**Fig. 2:**
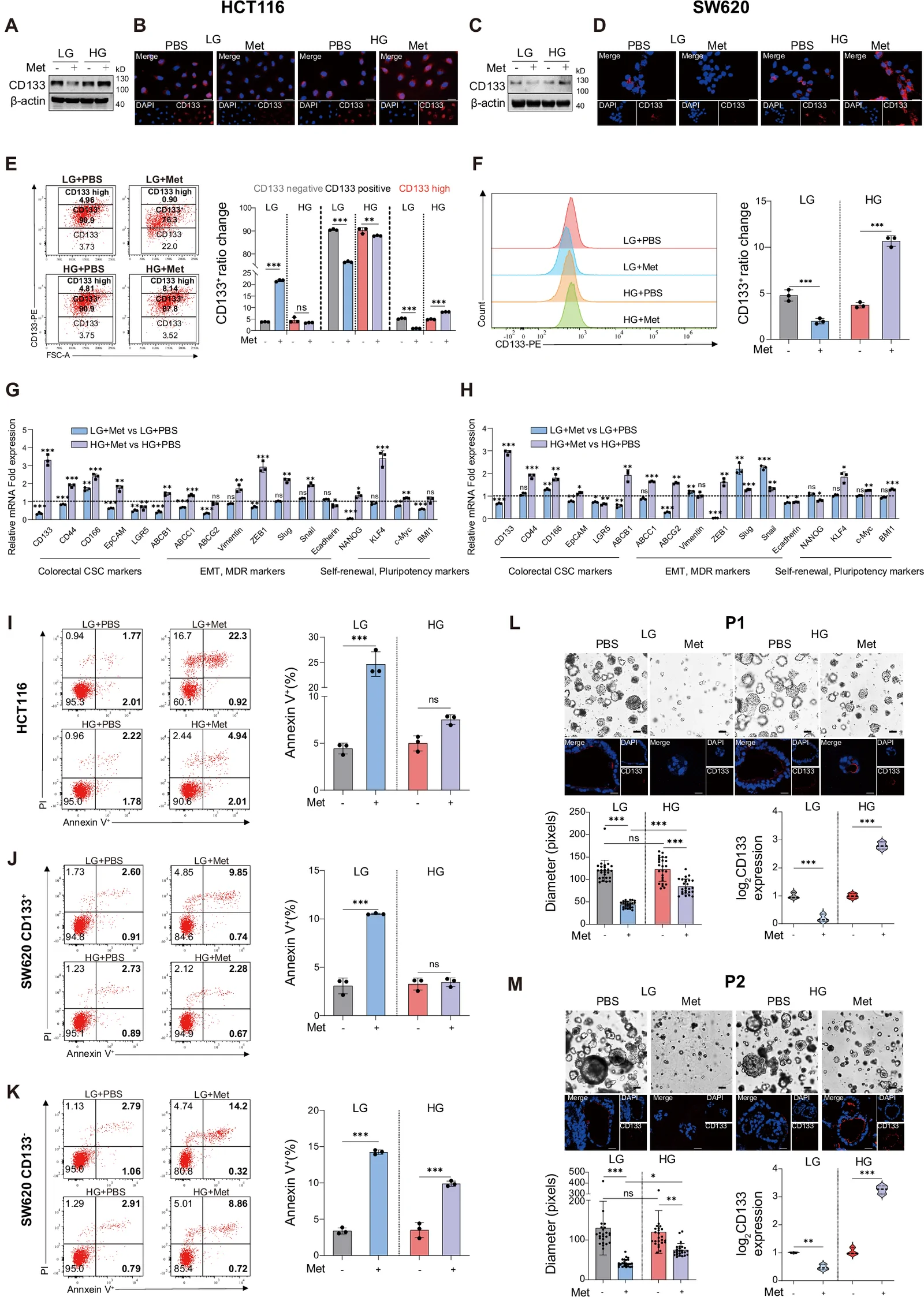
CD133^+^ colorectal CSCs exhibit resistance to metformin under HG conditions. CD133 expression was analyzed by western blotting (**A**, **C**) and immunofluorescence staining (**B,** **D**) in HCT116 and SW620 cells treated with metformin (Met, 10 mM, 24 h) under low-glucose (LG, 5.5 mM) or high-glucose (HG, 25 mM) conditions. In the immunofluorescence images, nuclei are counterstained with DAPI (blue), and CD133 is shown in red. In **A** and **C**, CD133 and β-actin were detected on the same blot, with β-actin serving as the loading control. Flow-cytometric analysis of CD133-negative, CD133-positive and CD133-high fractions in HCT116 cells (**E**), and the CD133⁺ fraction in SW620 cells (**F**) following Met (10 mM, 24 h) treatment under different glucose media (*n* = 3 independent biological replicates). Expression of the indicated genes is presented as fold change relative to PBS controls (mean of PBS-treated samples) in HCT116 (**G**) and SW620 (**H**) cells (*n* = 3 independent biological replicates). Met-induced apoptosis (Annexin V⁺) in HCT116 (**I**), SW620 CD133⁺ cells (**J**), and SW620 CD133⁻ bulk cells (**K**) (*n* = 3 independent biological replicates). Scatter plots show the percentage of Annexin V⁺ cells, and bar graphs summarize the quantification. **L**, **M** Representative images and quantification of CRC PDOs treated with PBS or Met under LG and HG culture conditions. Met (2 mM) was added 3–4 days after organoid seeding and continuously administered for 5 days. PDO size and number were assessed microscopically at the end of treatment. PDOs were subsequently subjected to quantification and immunofluorescence staining for CD133, followed by quantitative analysis. Organoid diameter was quantified from individual organoids within each Matrigel dome, and CD133 expression was quantified from 3 immunofluorescence images of individual organoids per group. Data are presented as mean values ± SD. Statistical significance was determined by two-sided two-way ANOVA followed by Tukey’s multiple-comparison test for **E,** **F** and **I**–**M**. For gene-expression comparisons in **G** and **H**, two-sided unpaired Student’s t-tests were performed for each gene. Scale bars, 20 μm for immunofluorescence images in **B** and **D**, 100 μm for representative organoid bright-field images in **L** and **M**, and 25 μm for organoid immunofluorescence images in **L** and **M**. HG, high glucose; LG, low glucose; Met, metformin. ns, no significance; *P < 0.05; **P < 0.01; ***P < 0.001. Source data are provided as a Source Data file.

To test our hypothesis, we modulated glucose in culture to define the conditions under which metformin impacts CD133⁺ colorectal CSCs. Metformin markedly increased CD133 expression under 25 mM glucose, but significantly suppressed CD133 at 2.5 mM and 5.5 mM (Fig. S3A). We, therefore, designated 25 mM as high glucose (HG) and, to ensure adequate proliferation in a lower-glucose milieu, selected 5.5 mM as low glucose (LG). Across three CRC cell lines, we confirmed that glucose counteracted metformin-mediated repression of CD133 (Fig. 2A, C, S3B). Immunofluorescence and flow cytometry further showed that, under HG, metformin increased the fraction of CD133⁺ cells in SW620 and HCT15 (Fig. 2B, D, F, and S3C–S3D). In HCT116 cells, which were predominantly CD133⁺ by flow cytometry, metformin induced a glucose-dependent shift in CD133 distribution. Specifically, metformin expanded the CD133⁻ fraction under LG conditions but increased the CD133-high subset under HG conditions (Fig. 2E; CD133-high was defined as the top 5% of cells in the LG + PBS control), supporting the use of HCT116 as the primary model for subsequent CD133⁺ CSC-focused in vivo experiments. Notably, glucose concentration selectively modulated the antitumor activity of metformin but did not appreciably alter the efficacy of standard CRC chemotherapeutics (Fig. S3E). Among all stemness, epithelial–mesenchymal transition (EMT)-related, and multidrug resistance (MDR)-associated genes surveyed, CD133 was uniquely and consistently regulated by metformin in a glucose-dependent manner across the three lines, being downregulated under LG and upregulated under HG (Fig. 2G, H, S3F). Other markers changed variably or in a cell line–restricted fashion, but none reproduced the uniform pattern observed for CD133. This intrinsic difference in CD133⁺ abundance reconciles the distinct in vivo outcomes between the HCT116 and SW620 models in Fig. 1. Under HCD, HCT116 tumors—composed almost entirely of CD133⁺ cells—were refractory to metformin, presenting with larger volumes, reduced Cleaved Caspase-3, and pronounced CD133⁺ enrichment by IF (Fig. 1D, E, L). In contrast, SW620 tumors—predominantly CD133⁻—remained responsive to metformin under HCD, exhibiting smaller volumes, increased Cleaved Caspase-3, and decreased Ki67, consistent with the absence of a CSC-enriched compartment (Fig. 1F, G, M). Collectively, these findings indicate that the blunted antitumor efficacy of metformin under HG/HCD conditions is primarily driven by the resistance of CD133⁺ CSCs rather than by properties of the bulk tumor population.

Colony-formation assays corroborated these observations. Under LG, metformin markedly impaired the clonogenicity of CD133⁺ cells (fewer colonies), whereas this inhibition was largely abolished under HG (Fig. S3G). Because sphere formation in vitro and limiting-dilution transplantation in immunocompromised mice are gold-standard CSC assays^23^, we next evaluated the stemness of metformin-induced CD133⁺ cells under HG. Metformin reduced both sphere number and size under LG, but failed to inhibit sphere formation under HG (Fig. S3H). We then magnetically isolated CD133⁺ and CD133⁻ subpopulations and assessed apoptosis after metformin. Under HG, metformin did not significantly induce apoptosis in CD133⁺ cells from SW620 or HCT15 and only modestly increased apoptosis in HCT116 (Fig. 2I, J, S3I). In contrast, CD133⁻ cells exhibited a significantly higher rate of apoptosis regardless of glucose condition (Fig. 2K, S3J).

We extended these findings to patient-derived organoids (PDOs). Under LG, metformin significantly curtailed PDO growth and reduced the proportion of CD133⁺ cells. Under HG, metformin still limited overall PDO expansion but unexpectedly increased the CD133⁺ fraction (Fig. 2L, M). Immunofluorescence revealed that CD133⁺ cells in HG plus metformin PDOs retained strong Ki67 comparable to untreated controls, indicating preserved proliferative activity despite drug exposure, whereas under LG plus metformin both the number of CD133⁺ cells and their Ki67 mean fluorescence intensity were markedly reduced (Fig. S4A–S4B). H&E staining and cell-diameter measurements showed no change in individual cell size, suggesting that reduced PDO volume reflected decreased cell number rather than cellular shrinkage (Fig. S4C–S4D). Together, these data demonstrate that under HG conditions, CD133⁺ colorectal CSCs are intrinsically resistant to metformin.

### CD133⁺ colorectal CSCs resist metformin under high-glucose conditions by upregulating CD36

**Fig. 3:**
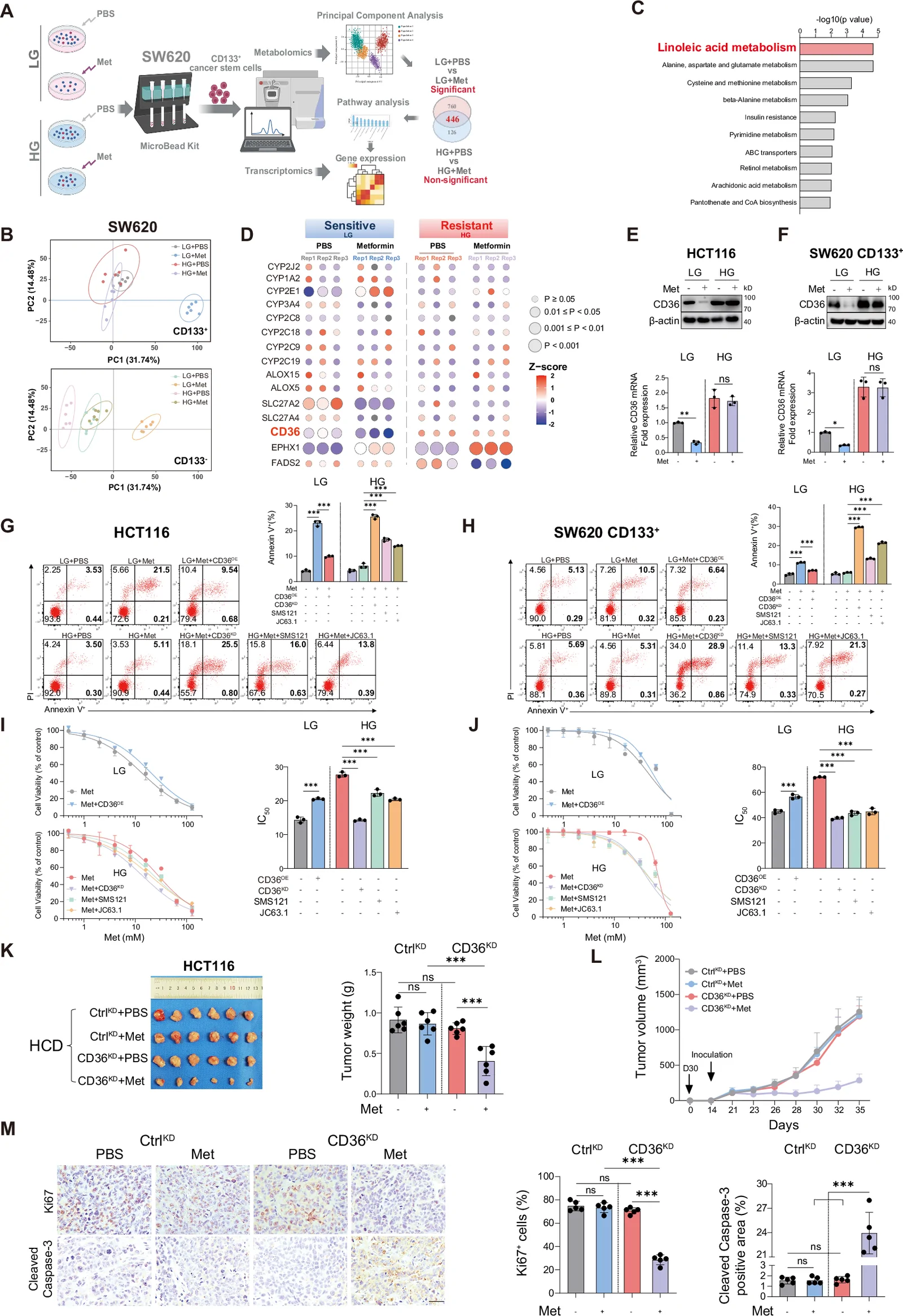
CD133⁺ colorectal CSCs resist metformin under high-glucose conditions by upregulating CD36. **A** Workflow for identifying and validating metformin-regulated metabolic pathways and genes in SW620 CD133⁺ cells under LG and HG conditions (*n* = 6 independent biological replicates). Metabolites with *P* < 0.05 were considered significantly changed. Created in BioRender. Li, X. (2026) https://BioRender.com/2qboh1g. **B** PCA plots of all detected metabolites in SW620 CD133⁺ cells treated with PBS or metformin under LG and HG conditions. **C** The top 10 enriched KEGG metabolic pathways among the 446 overlapping metabolites, ranked by their -log10 (*P* value). **D** Dot plot showing expression of genes in the LA metabolism pathway in metformin-sensitive and metformin-resistant CD133⁺ CSCs. Color indicates normalized expression, and dot size reflects the significance of the difference between Met- and PBS-treated samples. **E**, **F** CD36 protein and mRNA levels (*n* = 3 independent biological replicates) in metformin-sensitive and metformin-resistant CD133⁺ CSCs treated with PBS or metformin. CD36 protein expression was assessed by western blotting, and CD36 and β-actin were detected on the same blot, with β-actin serving as the loading control. **G**, **H** Flow cytometry analysis of Annexin V and PI staining in CD133^+^ CSCs under different glucose conditions, treated with PBS or Met, with or without CD36 overexpression (CD36^OE^), knockdown (CD36^KD^), or inhibitors (SMS121) and the CD36 blocking antibody JC63.1 (*n* = 3 independent biological replicates). The scatter plots show the percentage of Annexin V^+^ cells. The bar graph displays the quantification of Annexin V^+^ cells. **I**, **J** Cell viability assays of CD133^+^ CSCs under different glucose conditions after treatment with various concentrations of Met, with or without CD36^OE^, CD36^KD^, or SMS121 and JC63.1. The line graphs show the percentage of viable cells relative to control, while the bar graph displays the IC_50_ values for each condition (*n* = 3 independent biological replicates). **K** Images of tumors and tumor weights at the end of the experiment (*n* = 6 mice per group). **L** Tumor growth curves of HCT116^shNC^ and HCT116^shCD36^ xenografts in mice fed an HCD and treated with PBS or metformin (*n* = 6 mice per group). **M** Immunohistochemical staining of tumor tissues for Cleaved Caspase-3 and Ki67 expression, with quantitative analysis presented (*n* = 5 mice per group). Data are presented as mean ± SD. Statistical significance was determined by two-sided two-way ANOVA followed by Tukey’s multiple-comparison test for factorial comparisons in **E,** **F,** **K** and **M**. For comparisons involving multiple treatment groups in **G**–**J**, statistical significance was determined by ordinary one-way ANOVA followed by Tukey’s multiple-comparison test. Tumor growth curves in **L** were analyzed by ordinary two-way ANOVA followed by Tukey’s multiple-comparison test. Scale bars, 25 μm for immunohistochemical images (**M**). HG, high glucose; LG, low glucose; HCD, high-carbohydrate diet; Met, metformin. ns, no significance; *P < 0.05; **P < 0.01; ***P < 0.001. Source data are provided as a Source Data file.

We investigated mechanisms underlying metformin resistance in CD133⁺ colorectal CSCs under HG conditions. CD133⁺ CSCs were isolated after PBS or metformin treatment under both HG and LG conditions, and their metabolic and transcriptional responses were profiled (Fig. 3A). Principal component analysis (PCA) of global metabolites revealed clear separation between PBS- and metformin-treated SW620 CD133⁺ cells under LG, whereas under HG the two groups displayed substantial overlap (Fig. 3B). In CD133⁻ cells, PCA showed pronounced differences between PBS and metformin under both LG and HG (Fig. 3B). These data indicate that metformin broadly reprograms metabolism in CD133⁺ CSCs under LG conditions, but exerts limited metabolic impact under HG.

Under LG, metformin altered 446 metabolites in CD133⁺ CSCs (Fig. S5A), whereas no significant changes were detected under HG. Kyoto Encyclopedia of Genes and Genomes (KEGG) pathway enrichment showed significant clustering across multiple pathways, with linoleic acid (LA) metabolism and alanine/aspartate/glutamate metabolism most prominent (Fig. 3C). Given prior reports that aspartate, glutamate, and glucose can attenuate metformin sensitivity and promote proliferation^4,6,24^, we focused subsequent analyses on LA metabolism. Integrating RNA sequencing (RNA-seq) with reverse-transcription quantitative PCR (RT-qPCR) validation in CD133⁺ CSCs (HCT116 and SW620) (Fig. S5B, S5C) revealed that most pathway genes changed modestly; however, CD36 exhibited robust, reproducible regulation across models and emerged as a key candidate (Fig. 3D). Specifically, metformin significantly suppressed CD36 expression under LG conditions, whereas its effect on CD36 was negligible under HG conditions. Moreover, CD36 expression remained higher under HG than under LG conditions, regardless of PBS or metformin treatment (Fig. 3E, F). Consistent patterns were observed in previously established HCT116 tumor tissues in Fig. 1B (Fig. S6A). Notably, when comparing CD133⁻ and CD133⁺ fractions in HCT15 and SW620, baseline CD36 levels were comparable; upon HG plus metformin, CD36 was strongly upregulated only in the CD133⁺ fraction, with minimal change in CD133⁻ cells (Fig. S6B, S6C), suggesting a CSC-specific adaptive response to metformin stress.

Given CD36’s reported roles in CSC survival, chemoresistance, metabolic fitness, and metastasis^19,20,25,26^, we prioritized CD36 for functional interrogation. Using lentiviral CD36 overexpression (CD36^OE^) or shRNA knockdown (CD36^KD^) in CD133⁺ CSCs (Fig. S6D, S6E), we found that under LG, CD36 overexpression attenuated metformin-induced apoptosis and increased resistance (Fig. 3G–J). Conversely, under HG, CD36 knockdown enhanced metformin-induced apoptosis and reduced resistance. Pharmacologic inhibition with a CD36 inhibitor (SMS121) and blockade with the anti-CD36 antibody JC63.1 likewise potentiated metformin-induced apoptosis and diminished resistance in CD133⁺ CSCs (Fig. 3G–J). In vivo, CD36 knockdown rendered CD133⁺ colorectal CSCs responsive to metformin under HCD (Fig. 3K–M). Collectively, these findings demonstrate that CD36 mediates the resistance of CD133⁺ colorectal CSCs to metformin under HG conditions.

### CD36-mediated lipid metabolism contributes to metformin resistance in CD133^+^ colorectal CSCs under HG conditions

**Fig. 4:**
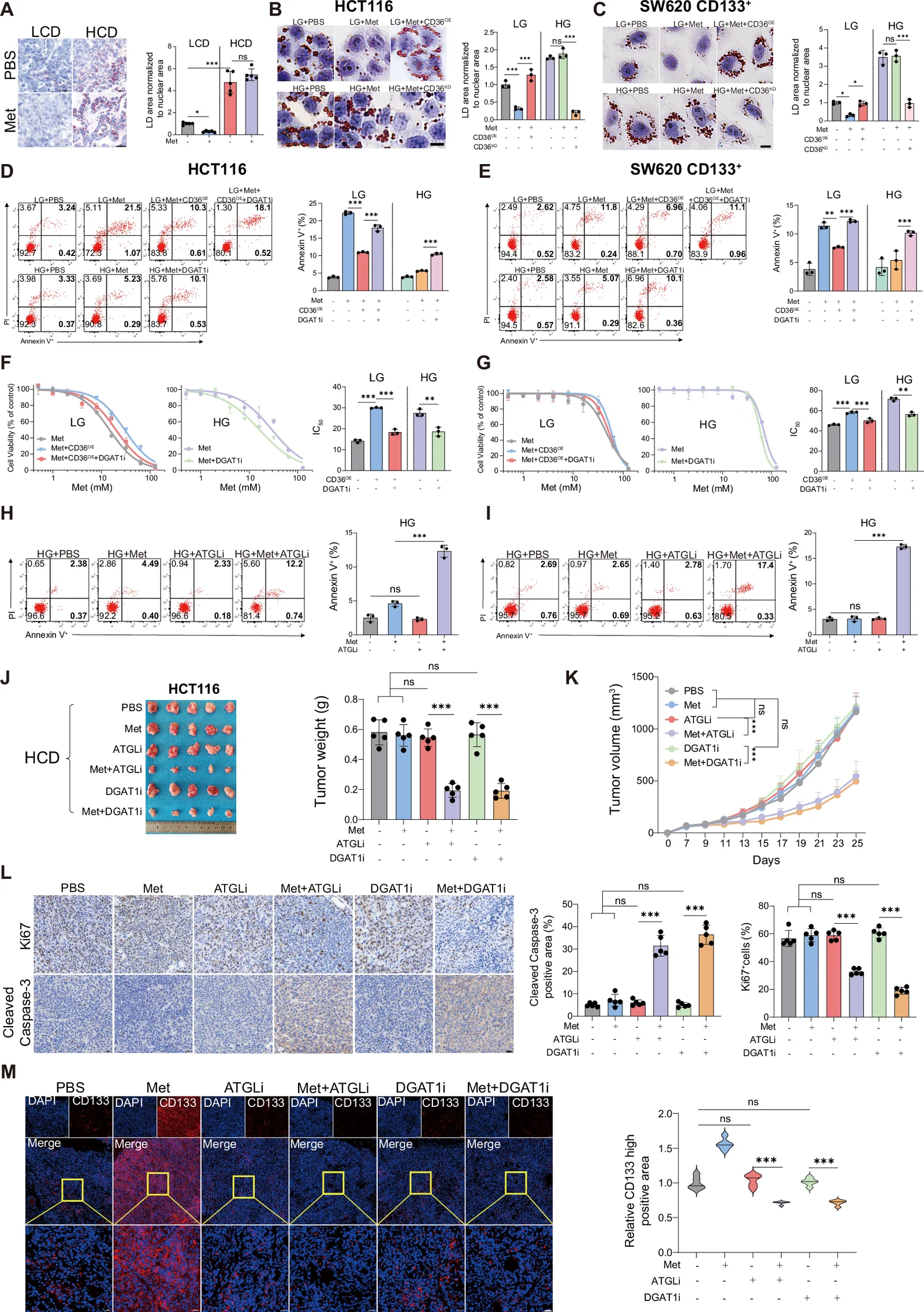
CD36-mediated lipid metabolism contributes to metformin resistance in CD133^+^ colorectal CSCs under high glucose conditions. **A** Representative images and quantification of LDs in tumor tissues from mice treated with Met under LCD and HCD conditions. Lipid accumulation was assessed by Oil Red O (ORO) staining, and LD area was quantitatively analyzed (*n* = 5 mice per group). **B**, **C** Representative images and quantification of ORO-stained LDs in CD133^+^ CSCs under different glucose conditions, with 50 μM LA, treated with PBS or Met, with or without CD36^OE^ or CD36^KD^. The bar graph shows the relative LDs area normalized to the nucleus area (*n* = 3 independent biological replicates). **D**, **E** Flow cytometry analysis of Annexin V and PI staining in CD133^+^ CSCs under different glucose conditions, treated with PBS or Met, with or without CD36^OE^ and DGAT1i. The bar graph displays the quantification of Annexin V^+^ cells (*n* = 3 independent biological replicates). **F**, **G** Cell viability assays of CD133^+^ CSCs under different glucose conditions, treated with Met, with or without CD36^OE^ and DGAT1i. The line graphs show the percentage of viable cells relative to control. The bar graph displays the IC_50_ values (*n* = 3 independent biological replicates). **H**, **I** Flow cytometry analysis of Annexin V and PI staining in CD133^+^ CSCs under HG conditions, treated with PBS, Met, ATGLi, or both Met and ATGLi (*n* = 3 independent biological replicates). The scatter plots show the percentage of Annexin V^+^ cells. The bar graph displays the quantification of Annexin V^+^ cells. **J** Representative images and quantification of tumor weights at the end of the experiment in mice bearing CD133⁺ CSC-derived tumors under HCD conditions, following treatment with PBS, Met, ATGLi, a combination of Met and ATGLi, DGAT1i, or a combination of Met and DGAT1i (*n* = 5 mice per group). **K** Tumor growth curves over the course of treatment. (*n* = 5 mice per group). **L** Immunohistochemical staining of tumor tissues for Cleaved Caspase-3 and Ki67 expression, with quantitative analysis presented (*n* = 5 mice per group). **M** Immunofluorescence staining of tumor tissues for CD133 expression, with quantitative analysis. Data are presented as mean ± SD. Statistical significance was determined by two-sided two-way ANOVA followed by Tukey’s multiple-comparison test for factorial comparisons in **A**, **H** and **I**. For comparisons involving multiple treatment groups in **B**–**G,** **J,** **L** and **M**, statistical significance was determined by ordinary one-way ANOVA followed by Tukey’s multiple-comparison test. Tumor growth curves in **K** were analyzed by ordinary two-way ANOVA followed by Tukey’s multiple-comparison test. Scale bars: 20 μm (**A**), 10 μm (**B**, **C**), and 20 μm (**L**, **M**). HG, high glucose; LG, low glucose; HCD, high-carbohydrate diet; Met, metformin; ATGLi, ATGL inhibitor; DGAT1i, DGAT1 inhibitor. ns, no significance; *P < 0.05; **P < 0.01; ***P < 0.001. Source data are provided as a Source Data file.

CD36 plays a pivotal role in the uptake of free fatty acids (FFAs), LD formation, and metabolic reprogramming—processes that mediate cancer resistance and metastasis^27,28^. We first analyzed LD accumulation in tumor tissues (Fig. 1B). Under LCD, metformin significantly reduced LD formation; under HCD, LD content was markedly elevated and showed no difference between the metformin and control groups (Fig. 4A). We next examined LD formation in CD133⁺ colorectal CSCs after PBS or Met across glucose conditions. LD abundance was higher under HG than LG; moreover, metformin decreased LD formation in Met-sensitive cells but increased LD accumulation in Met-resistant cells (Fig. 4B, C). Because targeting CD36 can reduce LDs^29^, we modulated CD36 and found that CD36 overexpression increased LD accumulation and attenuated Met-induced apoptosis under LG, whereas CD36 knockdown reduced LDs in HG-conditioned Met-resistant cells (Fig. 4B, C). Functionally, under LG, CD36 overexpression attenuated Met-induced apoptosis and enhanced resistance, whereas pharmacologic inhibition of LD biogenesis with a diacylglycerol O-acyltransferase 1 inhibitor (DGAT1i) reduced LDs (Fig. S7A–S7B) and restored metformin sensitivity (Fig. 4D–G). Conversely, in HG-conditioned Met-resistant cells, DGAT1i decreased LD accumulation, potentiated Met-induced apoptosis, and reduced resistance.

To verify causality, we applied DGAT1i while establishing HCT116 resistant populations. Blocking LD biogenesis markedly delayed or prevented resistance, whereas inhibitor withdrawal partially restored resistance with renewed lipid accumulation (Fig. S7C, S7D), indicating that DGAT1-dependent lipid storage supports metformin resistance in CD133⁺ CRC CSCs. To distinguish whether LD accumulation arises from de novo lipogenesis or from the uptake of exogenous fatty acids, we quantified triglycerides (TG) and lipogenic markers. Consistent with lipid staining, TG levels were significantly higher under HG conditions, particularly in metformin-treated cells, than under LG conditions; moreover, metformin markedly reduced TG under LG but had minimal impact under HG (Fig. S8A). Temporal profiling of FASN and p-ACC further supported this view: in metformin-treated cells under LG, p-ACC remained elevated from 3 to 24 h and FASN was markedly reduced at 24 h, indicating sustained suppression of de novo lipogenesis; in metformin-treated cells under HG, p-ACC increased at 3–12 h but returned toward baseline by 24 h, while FASN showed only a slight decrease, suggesting a transient inhibition followed by partial recovery (Fig S8B). Together with the 24-h increase in LDs, these findings favor enhanced uptake and storage of exogenous fatty acids rather than increased de novo synthesis under HG.

We then asked whether resistance depends chiefly on LD storage (buffering oxidative stress)^30^ or fatty-acid oxidation (FAO) supporting ATP/NADPH and redox defense^31–33^. FAO was significantly elevated in resistant cells (Fig. S8C). In CD133⁺ CSCs, LG+Met markedly decreased ATP and NADPH and increased ROS; under HG, Met had little effect on ATP or NADPH, ROS was unchanged, and ATP/NADPH exceeded LG regardless of treatment (Fig. S8D–S8F); lipid peroxidation did not differ among groups (Fig. S8G). In parallel, redox metabolite profiling showed that in Met-sensitive cells, antioxidants (N-acetylcysteine, NAC; reduced glutathione, GSH) decreased while oxidized glutathione (GSSG) increased (Fig. S8H), indicating that under metformin-induced metabolic stress, CD133⁺ cells must maintain redox homeostasis to avert cell death.

Prior work has shown that metformin can enhance FAO via activation of AMP-activated protein kinase (AMPK)^34,35^. Consistently, we observed robust AMPK phosphorylation under LG; however, because LD substrates are scarce, FAO remained low and cells persisted in an energy crisis. Under HG, metformin strongly activated p-AMPK at early time points (3–12 h), followed by a decline with metabolic adaptation (Fig. S8B), consistent with dynamic upregulation of FAO to partially alleviate energy stress. Given that metformin impairs the electron transport chain (ETC), we investigated how resistant cells sustain FAO: transmission electron microscopy (TEM) revealed increased mitochondrial number with dense, intact cristae, and Seahorse analyses confirmed preserved/enhanced FAO capacity (Fig. S8I, S8J). Functionally, under HG, pretreatment with an adipose triglyceride lipase inhibitor (ATGLi) to block lipid-droplet mobilization significantly reduced NADPH and potentiated metformin-induced apoptosis of CD133⁺ cells (Fig. 4H–I, S8E), underscoring FAO’s role in maintaining energy supply and redox balance. In vivo, under HCD, co-administration of metformin with a DGAT1i or ATGLi significantly altered tissue LD content and FAO (Fig. S8K, S8L) and effectively reversed metformin resistance in CD133⁺ colorectal CSCs (Fig. 4J–M). Collectively, these findings show that, under HG conditions, CD36-mediated lipid metabolic reprogramming promotes fatty acid uptake and oxidation, thereby sustaining energy homeostasis and the glutathione-dependent antioxidant system. This mechanism enables CD133⁺ colorectal CSCs to acquire resistance to metformin.

### Lactate from metabolic reprogramming induces metformin resistance in CD133^+^ colorectal CSCs under HG conditions

**Fig. 5:**
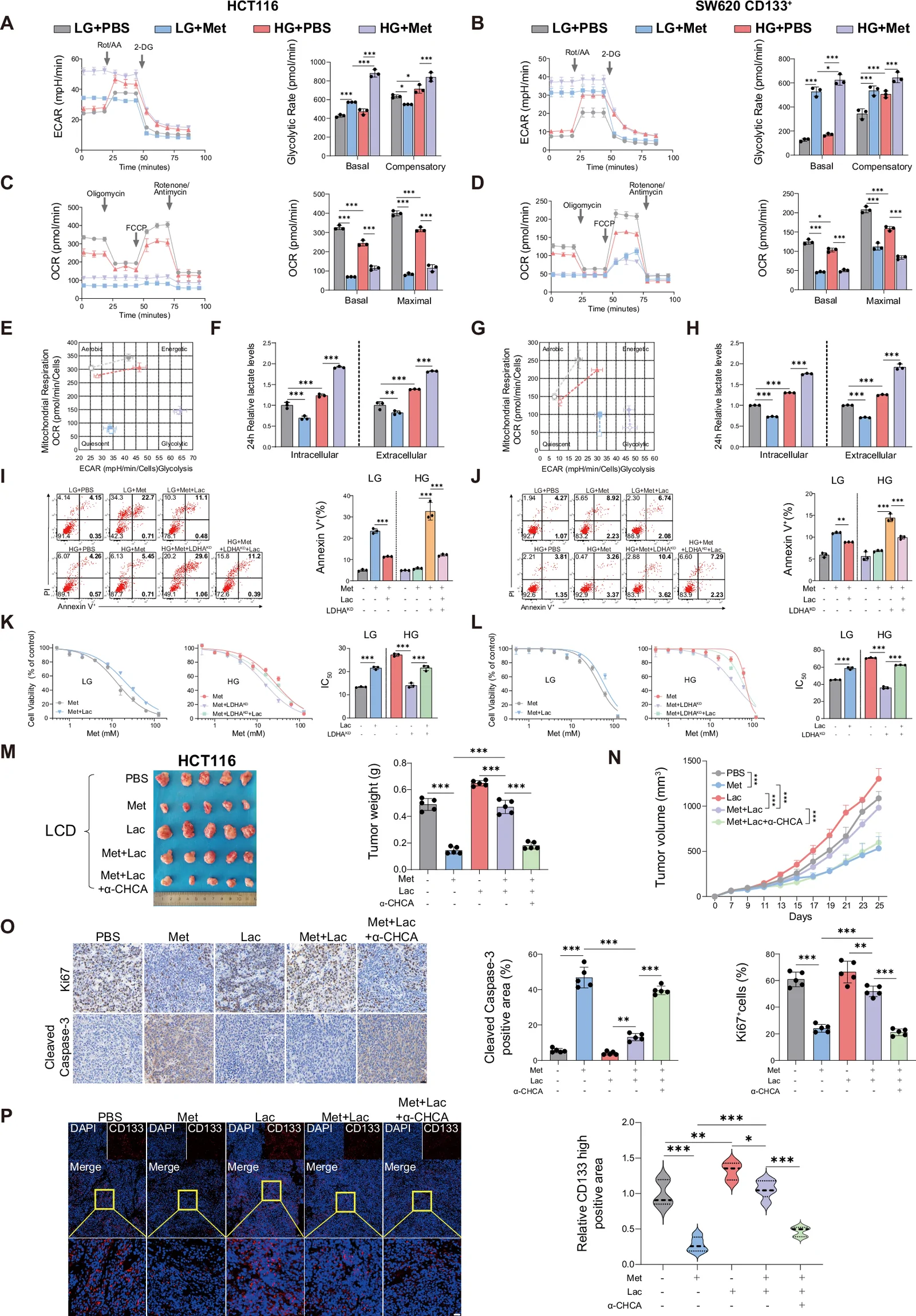
Lactate from metabolic reprogramming induces metformin resistance in CD133^+^ colorectal CSCs under high glucose conditions. **A**, **B** The extracellular acidification rate (ECAR) over time and the calculated glycolytic rates at different stages of measurement are shown (*n* = 3 independent biological replicates). The effects of Met on glycolytic rate were assessed in CD133^+^ CSCs treated for 24 h under different glucose conditions using the Seahorse Glycolysis Rate Assay. **C**, **D** Cellular oxygen consumption rates (OCRs) over time and OCRs at different stages of measurement are shown (*n* = 3 independent biological replicates). The effects of Met on mitochondrial respiration were assessed in CD133^+^ CSCs treated for 24 h under different glucose conditions using the Seahorse Mitochondrial Stress Test kit. **E**, **G** Energy phenotype of CD133^+^ CSCs in basal (empty shapes) or metabolic stress (full shapes) conditions (*n* = 3 independent biological replicates). The effects of Met on energy phenotype were assessed in CD133^+^ CSCs treated for 24 h under different glucose conditions using the Seahorse Energy Phenotype Test kit. **F**, **H** 24-h relative extracellular and intracellular Lac levels in CD133^+^ CSCs treated with or without Met under different glucose conditions (*n* = 3 independent biological replicates). **I**, **J** Flow cytometry analysis of Annexin V and PI staining in CD133^+^ CSCs under different glucose conditions, treated with PBS, Met, Lac, LDHA^KD^, or a combination of Met, Lac, and LDHA^KD^ (*n* = 3 independent biological replicates). The scatter plots show the percentage of Annexin V^+^ cells. The bar graph displays the quantification of Annexin V^+^ cells. **K**, **L** Cell viability assays of CD133^+^ CSCs under different glucose conditions, treated with Met, Lac, LDHA^KD^, or a combination of Met, Lac, LDHA^KD^ (*n* = 3 independent biological replicates). The line graphs show the percentage of viable cells relative to control, and the bar graphs show IC₅₀ values. **M** Representative images and quantification of tumor weights at the end of the experiment in mice bearing CD133⁺ CSC-derived tumors under LCD conditions following treatment with PBS, Met, Lac, a combination of metformin and Lac, or a combination of Met, Lac, and α-CHCA (*n* = 5 mice per group). **N** Tumor growth curves over the course of treatment (*n* = 5 mice per group). **O** Immunohistochemical staining of tumor tissues for Cleaved Caspase-3 and Ki67 expression, with quantitative analysis presented (*n* = 5 mice per group). **P** Immunofluorescence staining of tumor tissues for CD133 expression, with quantitative analysis. Data are presented as mean ± SD. Statistical significance was determined by two-sided two-way ANOVA followed by Tukey’s multiple-comparison test for factorial comparisons in **A**–**H**. For comparisons involving multiple treatment groups in **I**–**M**, **O** and **P**, statistical significance was determined by ordinary one-way ANOVA followed by Tukey’s multiple-comparison test. Tumor growth curves in **N** were analyzed by ordinary two-way ANOVA followed by Tukey’s multiple-comparison test. Scale bars, 20 μm for immunohistochemical images (**O**) and immunofluorescence images (**P**). HG, high glucose; LG, low glucose; LCD, low-carbohydrate diet; Met, metformin; Lac, lactate; α-CHCA, α-cyano-4-hydroxycinnamic acid; ns, no significance; *P < 0.05; **P < 0.01; ***P < 0.001. Source data are provided as a Source Data file.

Preclinical data indicate that metformin inhibits respiratory-chain complex I (NADH: ubiquinone oxidoreductase), thereby altering electron transport chain function and suppressing OXPHOS^2^. This inhibition increases cellular reliance on glycolysis and promotes lactate production and export^36,37^. To test this in CD133⁺ colorectal CSCs, we profiled glucose metabolism and mitochondrial respiration across glucose conditions. Metformin significantly increased basal and compensatory glycolysis (except under LG in HCT116), with stronger effects under HG (Fig. 5A, B), and it inhibited mitochondrial respiration across conditions (Fig. 5C, D). Under LG, CD133⁺ CSCs were more dependent on mitochondrial respiration, consistent with the notion that glucose limitation determines metformin sensitivity^24^. Accordingly, metformin-treated CD133⁺ CSCs exhibited a strong glycolytic phenotype, particularly under HG (Fig. 5E, G). To further confirm the impact of metformin on glycolysis in CD133^+^ colorectal CSCs, we measured intracellular and extracellular lactate levels. The results consistently showed that after 24 h of metformin treatment under HG conditions, cellular lactate levels are highest. Although metformin also increased glycolytic rates under LG conditions, intracellular and extracellular lactate levels were reduced rather than increased after 24 h (Fig. 5F, H). In vivo, intratumoral lactate decreased with metformin under LCD but increased under HCD relative to HCD controls (Fig. S9A). Analysis of lactate production curves revealed that while metformin initially promotes lactate production under LG conditions, the rate decreases significantly over time, possibly due to restricted glucose supply limiting overall glycolytic rates (Fig. S9B, S9C). Moreover, metformin significantly inhibits cell proliferation (Fig. 1L), resulting in reduced overall lactate production over time. In summary, metformin induces metabolic reprogramming in CD133^+^ colorectal CSCs characterized by increased glycolytic flux and enhanced lactate production.

CSCs are known to possess remarkable metabolic plasticity and adaptability to maintain survival, evade host immune responses, and meet their nutritional needs under drug treatment pressure^38^. A study on metformin and CSCs further confirms this, demonstrating that pancreatic CSCs exhibit a glycolytic/respiratory intermediate phenotype through metabolic reprogramming, enabling adaptation to metformin-induced energy crisis and apoptosis, ultimately leading to the emergence of drug-resistant CSC clones^11^. In our study, metformin-treated CD133^+^ colorectal CSCs exhibit a strong glycolytic phenotype to meet biosynthetic and energy demands, producing large amounts of lactate under HG conditions, a hallmark of drug-resistant cancer cells^16,39–41^. To ascertain the role of lactate in metformin resistance in CD133^+^ colorectal CSCs, we supplemented lactate in LG culture medium, which attenuated metformin-induced apoptosis and enhanced cell resistance to metformin (Fig. 5I–L). Lactate dehydrogenase A (LDHA) is a key glycolytic enzyme that facilitates lactate production^42^. Silencing LDHA by siRNA under HG reduced lactate levels (Fig. S9D–S9G), thereby enhancing metformin-induced apoptosis and decreasing resistance. Notably, metformin does not alter LDHA expression (Fig. S10A). In addition, supplementation with 10 mM lactate reversed the pro-apoptotic and sensitizing effects of LDHA knockdown (Fig. 5I–L). Similarly, in vivo, lactate supplementation promoted resistance to metformin in CD133⁺ colorectal CSCs under LCD conditions, accompanied by an expansion of CD133-high positive areas. However, this resistance was abolished upon administration of the monocarboxylate transporter (MCT) inhibitor α-cyano-4-hydroxycinnamic acid (α-CHCA), which blocks the cellular uptake of exogenous lactate, leading to a reduction in CD133-high positive areas (Fig. 5M–P). Overall, these findings illustrate that lactate mediates metformin resistance in CD133^+^ colorectal CSCs under HG conditions.

### Lactate promotes CD36 expression through transcriptional activation of c-JUN

**Fig. 6:**
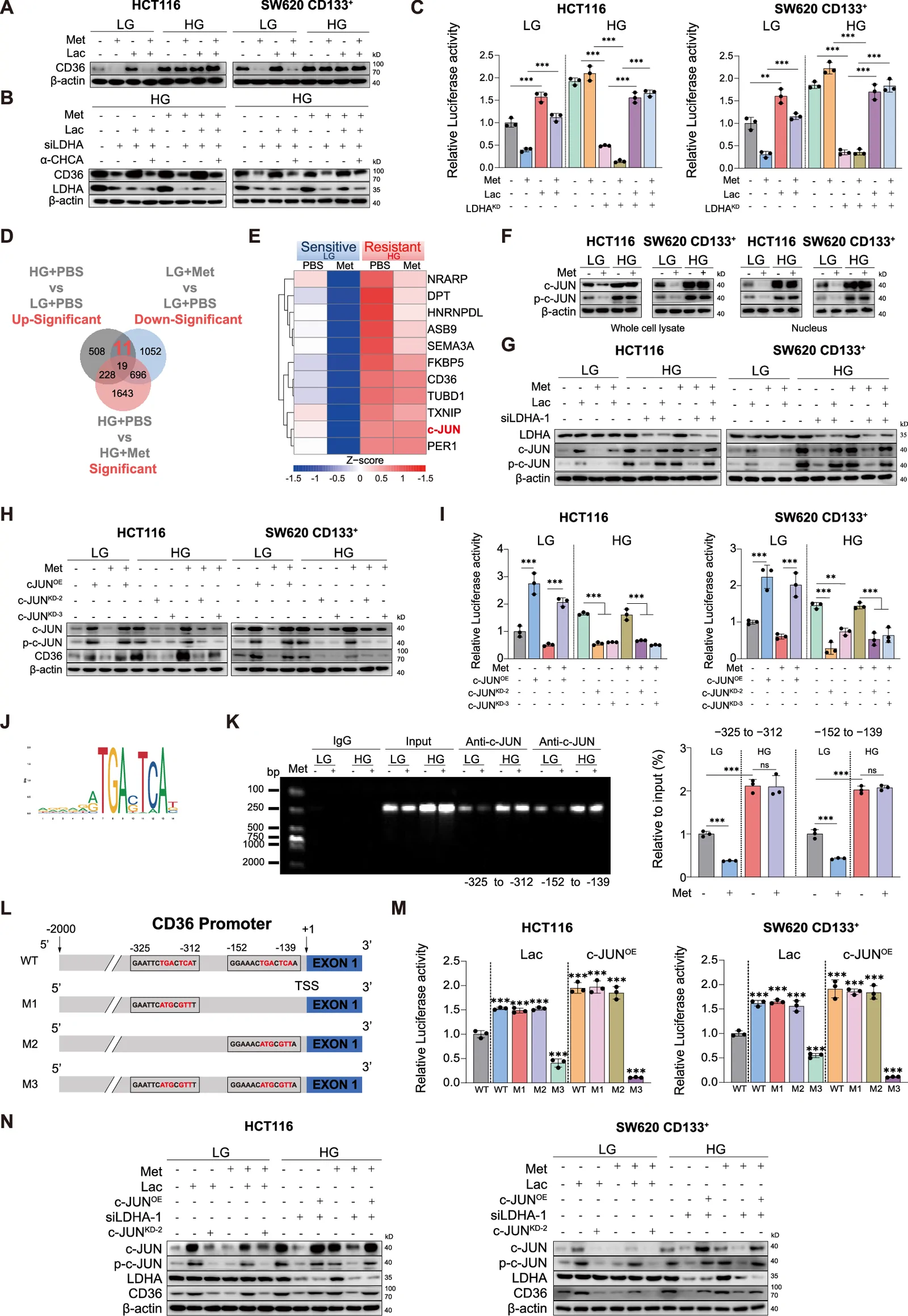
Lactate promotes CD36 expression through transcriptional activation of c-JUN. **A** Western blot analysis of CD36 expression in CSCs under different glucose conditions, treated with or without Met and Lac. CD36 and β-actin were detected on the same blot, with β-actin serving as the loading control. **B** Western blot analysis of CD36 and LDHA expression in CSCs under HG conditions, treated with or without Met, Lac, LDHA^KD^, and α-CHCA (5 mM). CD36 and β-actin were detected on the same blot, with β-actin serving as the loading control, whereas LDHA was detected on a separate blot. The samples derive from the same experiment, and the blots were processed in parallel. **C** Relative CD36 promoter-luciferase activity in CSCs under different glucose conditions, treated with or without Met, Lac, and LDHA^KD^. Luciferase activity was determined and normalized using the dual luciferase reporter system (*n* = 3 independent biological replicates). **D** Venn diagram illustrating the strategy for identifying transcription factors regulating CD36. **E** Heatmap showing expression levels of genes identified in **D**. **F** Western blot analysis of c-JUN and p–c-JUN in whole-cell lysates and nuclear extracts from CSCs under different glucose conditions, treated with or without Met. For whole-cell lysates, different blots for c-JUN, p-c-JUN, and β-actin were processed in parallel. For nuclear extracts, c-JUN and Histone H3 were detected on the same blot, whereas p-c-JUN was detected on a separate blot processed in parallel. β-actin and histone H3 served as loading controls for whole-cell lysates and nuclear extracts, respectively. The samples derive from the same experiment. **G** Western blot analysis of LDHA, c-JUN, and p-c-JUN expression in CSCs under HG conditions, treated with or without Met, Lac, and LDHA^KD^. The samples derive from the same experiment, but c-JUN, p-c-JUN, LDHA, and β-actin were detected on separate blots processed in parallel. **H** Western blot analysis of c-JUN, p-c-JUN and CD36 expression in CSCs under different glucose conditions, treated with or without Met, c-JUN^OE^, and c-JUN^KD^ (shc-JUN-2 and shc-JUN-3). CD36 and β-actin were detected on the same blot, with β-actin serving as the loading control, whereas c-JUN and p-c-JUN were detected on separate blots. The samples derive from the same experiment, and the blots were processed in parallel. **I** Relative CD36 promoter-luciferase activity in CSCs under different glucose conditions, treated with or without Met, c-JUN^OE^, and c-JUN^KD^ (*n* = 3 independent biological replicates). **J** Sequence representing the binding motif of c-JUN. **K** ChIP-PCR showing c-JUN binding to the CD36 promoter regions (−325 to −312 and −152 to −139) in HCT116 cells under different glucose conditions, treated with or without Met. IgG and input controls are included for comparison (*n* = 3 independent biological replicates). **L** Schematic representation of the wild-type (WT) and mutant (M1, M2, M3) CD36 promoter constructs. The specific c-JUN binding sites (−325 to −312 and −152 to −139) and their mutations are indicated. **M** Relative CD36 promoter-luciferase activity in CSCs transfected with WT or mutant CD36 promoter constructs, treated with Lac or c-JUN^OE^ (*n* = 3 independent biological replicates). **N** Western blot analysis of c-JUN, p-c-JUN, LDHA, and CD36 expression in CSCs under different glucose conditions, treated with or without Met, Lac, c-JUN^OE^, siLDHA-1, and c-JUN^KD^. c-JUN, p-c-JUN, CD36, and β-actin were detected on separate blots. The samples derive from the same experiment, and all blots were processed in parallel. Data are presented as mean ± SD. Statistical significance was determined by ordinary one-way ANOVA followed by Tukey’s multiple-comparison test for comparisons involving multiple treatment groups in **C**, **I** and **M**. For factorial comparisons in **K**, statistical significance was determined by two-sided two-way ANOVA followed by Tukey’s multiple-comparison test. HG, high glucose; LG, low glucose; Met, metformin; Lac, lactate. ns, no significance; *P < 0.05; **P < 0.01; ***P < 0.001. Source data are provided as a Source Data file.

To determine whether lactate leads to an increase in CD36 expression, we added 10 mM exogenous lactate to the culture medium of CD133^+^ colorectal CSCs. Under LG conditions, lactate upregulated the expression of CD36, whereas under HG conditions, the effect on CD36 expression was not significant, possibly due to saturated expression levels (Fig. 6A). In HG, reducing endogenous lactate with LDHA siRNA decreased CD36, and exogenous lactate restored its expression (Fig. 6B). Because lactate transport is mediated by MCT1 (*SLC16A1*) and MCT4 (*SLC16A3*)^43^, we used α-CHCA to prevent exogenous lactate from entering cells, along with siRNA targeting LDHA (siLDHA) to inhibit endogenous lactate production, which decreased CD36 expression (Fig. 6B). Building on our prior work that lactate can act via GPR81 and acidify the media to regulate EMT^44,45^, we next asked whether lactate regulates CD36 through GPR81 or extracellular pH. After confirming that metformin does not affect GPR81 expression (Fig. S10A), we found that lactate still promoted CD36 expression after GPR81 knockdown, and acidifying the medium with HCl failed to induce CD36 (Fig. S10B). Thus, lactate-driven CD36 upregulation depends on lactate uptake and availability but is not mediated by GPR81 signaling or extracellular acidification.

We then investigated how lactate regulates CD36. First, we tested post-transcriptional routes. Exogenous lactate, like metformin, did not alter *CD36* mRNA decay or protein stability (Fig. S11), pointing to transcriptional control. To verify this hypothesis, we constructed a reporter plasmid containing the *CD36* promoter, and the results demonstrated that lactate markedly enhanced reporter gene activity (Fig. 6C). To identify transcription factors regulating *CD36*, we used a screening strategy shown in Fig. 6D, identifying 11 candidate genes, with *c-JUN* drawing particular attention (Fig. 6E). c-JUN, a member of the oncogenic transcription factor family AP-1 (Activator protein-1), binds to cognate DNA sites within gene promoter elements to regulate a variety of target genes. It plays a critical role in cell differentiation, migration, proliferation, and apoptosis, and is upregulated or overexpressed in various cancers^46^. We then measured total and phosphorylated c-JUN (p-c-JUN) protein levels in nuclear and whole-cell extracts. Under LG conditions, metformin markedly suppressed c-JUN and p-c-JUN expression, whereas under HG it had little effect; both PBS- and Met-treated cells showed higher c-JUN/p-c-JUN than under LG, mirroring CD36 (Fig. 6F). Notably, lactate supplementation increased both total and p-c-JUN levels, while LDHA knockdown significantly reduced them (Fig. 6G), indicating that lactate not only enhances c-JUN expression but also promotes its activation.

Subsequently, CD133^+^ colorectal CSCs were infected with c-JUN overexpression lentivirus (c-JUN^OE^) or c-JUN shRNA (c-JUN^KD^) to evaluate its functional role in metformin resistance (Fig. S12A). Functionally, c-JUN overexpression significantly attenuated metformin-induced apoptosis and enhanced cell resistance, whereas c-JUN knockdown or pharmacologic inhibition of upstream JNK signaling using SP600125 restored metformin sensitivity and markedly enhanced apoptosis (Fig. S12B–S12E). In vivo, metformin combined with c-JUN knockdown or JNK inhibition substantially reduced tumor growth and decreased the proportion of CD133-high cells under HCD conditions, while c-JUN overexpression conferred resistance to metformin and suppressed its pro-apoptotic effect (Fig. S13A–S13D).

We next examined whether c-JUN directly regulates CD36 expression in CD133^+^ colorectal CSCs. Consistent with this hypothesis, overexpression of *c-JUN* promoted CD36 expression under LG conditions in both PBS- and Met-treated cells, whereas c-JUN knockdown suppressed CD36 expression under HG conditions (Fig. 6H). Dual-luciferase assays demonstrated that c-JUN regulates *CD36* promoter activity (Fig. 6I). Bioinformatic analysis supported c-JUN binding within the *CD36* promoter (Supplementary Table 1; sites in Fig. 6J). Chromatin immunoprecipitation (ChIP) assays confirmed c-JUN binding at −325 to −312 and −152 to −139, with metformin inhibiting this interaction under LG but not HG; ChIP-qPCR verified significant enrichment at both sites, higher in HG than LG in both PBS- and Met-treated groups (Fig. 6K). Subsequently, we designed CD36 promoter sequences harboring mutations at the predicted binding sites. The results showed that mutation of both predicted binding sites abolished the effects of lactate and c-JUN on CD36 promoter activity (Fig. 6L, M). These results confirmed the regulatory relationship between c-JUN and CD36 expression. Finally, we reconfirmed the relationship between lactate, c-JUN, and CD36, showing that lactate can regulate CD36 expression, but this process requires mediation by LDHA and c-JUN (Fig. 6N). Together, these findings identify c-JUN as the key transcriptional mediator of lactate-induced CD36 upregulation in CD133⁺ colorectal CSCs.

### Lactate-driven histone lactylation promotes *c-JUN* transcription and confers metformin resistance

**Fig. 7:**
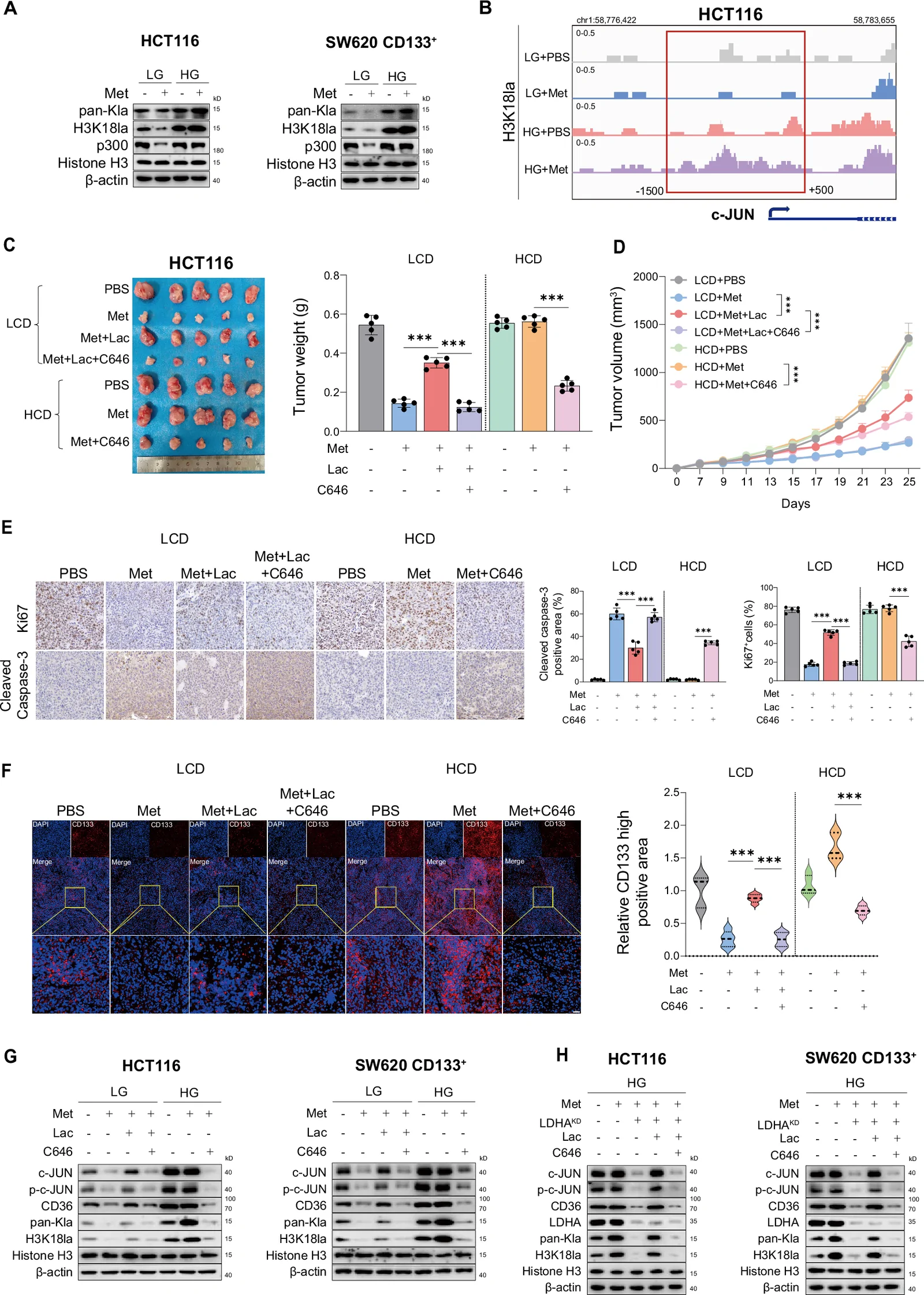
Lactate-driven histone lactylation promotes *c-JUN* transcription and confers metformin resistance. **A** Western blot analysis of pan-Kla, H3K18la, and p300 in HCT116 and SW620 CD133⁺ CSCs cultured under LG or HG conditions, treated with PBS or Met. Histone H3, p300, and β-actin were detected on the same blot. H3K18la and pan-Kla were detected on separate blots. The samples derive from the same experiment, and all blots were processed in parallel. **B** CUT&Tag tracks showing the enrichment of H3K18la at the *c-JUN* promoter region in HCT116 cells under different glucose conditions, treated with PBS or Met. The red box highlights the region of interest (−1500 to +500 relative to the TSS of *c-JUN*). Representative endpoint tumors and quantification of tumor weights (**C**) and tumor growth curves (**D**) of HCT116 xenografts in mice fed a LCD or HCD and treated with PBS or Met, with additional Lac supplementation and/or the p300 inhibitor C646 as indicated (*n* = 5 mice per group). **E** IHC staining of xenograft tumors for Ki67 and cleaved Caspase-3 under the indicated dietary and treatment conditions, with quantitative analyses shown (*n* = 5 mice per group). **F** IF staining of xenograft tumors for CD133, with quantification of CD133-high positive area (*n* = 5 mice per group). **G** Western blot analysis of c-JUN, p-c-JUN, CD36, pan-Kla, and H3K18la in HCT116 and SW620 CD133⁺ CSCs cultured under LG or HG and treated with Met, Lac, and/or C646 as indicated. CD36, Histone H3, and β-actin were detected on the same blot, whereas pan-Kla, H3K18la, c-JUN, and p-c-JUN were detected on separate blots. The samples derive from the same experiment, and all blots were processed in parallel. **H** Western blot analysis in HG-cultured CSCs showing the effects of LDHA knockdown, Lac supplementation, and/or C646 on pan-Kla/H3K18la and the c-JUN/p-c-JUN/CD36 axis under metformin treatment. CD36, pan-Kla, and β-actin were detected on the same blot, whereas LDHA, Histone H3, H3K18la, c-JUN, and p-c-JUN were detected on separate blots. The samples derive from the same experiment, and all blots were processed in parallel. Data are presented as mean ± SD. Statistical significance was determined by ordinary one-way ANOVA followed by Tukey’s multiple-comparison test for **C**, **E** and **F**. Tumor growth curves in **D** were analyzed by ordinary two-way ANOVA followed by Tukey’s multiple-comparison test. HG, high glucose; LG, low glucose; Met, metformin; Lac, lactate; C646, p300 inhibitor; ns, no significance; *P < 0.05; **P < 0.01; ***P < 0.001. Source data are provided as a Source Data file.

Histone lysine lactylation (Kla) is an epigenetic modification that can directly stimulate transcription^47^. In tumor tissues shown in Fig. 1B, metformin decreased pan-lysine lactylation (pan-Kla) and H3K18 lactylation (H3K18la) in CD133⁺ tumors under LCD, whereas under HCD it increased both marks in CD133⁺ CRC tissues (Fig. S14A). Western blotting confirmed the changes in H3K18la (Fig. S14B). In line with these in vivo findings, in vitro analyses showed that under HG conditions, pan-Kla and H3K18la were elevated and were further enhanced by metformin (Fig. 7A; Fig. S14C). By contrast, under LG conditions, metformin suppressed the expression of p300, a reported “writer” of histone lactylation^47^, and concomitantly reduced cellular lactate production (Fig. 5F, H), leading to diminished histone lactylation. Moreover, the data in Fig. S10 indicate that lactate regulates c-JUN predominantly at the transcriptional level. Consistently, CUT&Tag profiling of the *c-JUN* promoter revealed robust enrichment of H3K18la in HG, metformin-resistant cells, whereas H3K18la signal at the promoter was markedly reduced in LG, metformin-sensitive cells (Fig. 7B).

Based on these observations, we next performed functional studies to determine whether histone lactylation is causally involved in the development of metformin resistance. Pharmacological inhibition of lactylation with the p300 inhibitor C646 significantly attenuated metformin resistance in CD133⁺ colorectal CSCs under HG conditions both in vitro and in vivo; conversely, under LG conditions, suppressing lactylation effectively reversed the metformin-resistant phenotype induced by exogenous lactate (Fig. 7C–F; Fig. S15A–S15D).

To delineate the hierarchical relationship within the histone lactylation-c-JUN/CD36 axis, we found that inhibition of H3K18la concomitantly reduced c-JUN and CD36 expression (Fig. 7G). Under HG conditions, LDHA knockdown decreased endogenous lactate production, reduced cellular lactylation, and markedly suppressed c-JUN/CD36 expression as well as p-c-JUN. Exogenous lactate restored lactylation and rescued c-JUN, CD36, and p-c-JUN levels, whereas re-inhibition of lactylation abrogated these rescue effects (Fig. 7H). Moreover, blockade of JNK signaling or c-JUN knockdown prevented elevated lactylation from inducing CD36 expression; in contrast, under lactylation-inhibited conditions, c-JUN overexpression remained sufficient to promote CD36 expression (Fig. S16A). Consistently in vivo, reducing tumor lactylation enhanced CSC sensitivity to metformin, whereas enforced expression of c-JUN or CD36 restored the resistant phenotype (Fig. S16B–S16E). Collectively, these data establish histone lactylation-dependent activation of the c-JUN/CD36 axis as a key regulatory mechanism driving metformin resistance. We next evaluated whether the lipid metabolic phenotypes lie downstream of the histone lactylation-c-JUN/CD36 axis. Inhibition of histone lactylation markedly reduced FAO and LD accumulation; conversely, even under lactylation-inhibited conditions, enforced expression of c-JUN or CD36 partially rescued these lipid metabolic phenotypes. Meanwhile, upon c-JUN or CD36 knockdown, supplementation with exogenous lactate no longer promoted FAO or LD accumulation (Fig. S17). Collectively, these results indicate that FAO and lipid storage represent downstream metabolic outputs of lactylation-driven activation of the c-JUN/CD36 axis.

In parallel, we excluded a major contribution of histone acetylation to this phenotype. Both global acetylation (pan-Kac) and H3K18 acetylation (H3K18ac) were reduced under LG+Met, whereas no obvious differences were observed among the other conditions (LG, HG, and HG+Met). Moreover, ChIP-seq profiling of H3K18ac occupancy around the c-JUN promoter revealed no obvious enrichment differences among these groups, arguing against a promoter acetylation–driven mechanism for c-JUN activation (Fig. S18A, S18B). Consistently, increasing histone acetylation with 10 mM sodium acetate (NaAc) did not alter c-JUN or CD36 expression (Fig. S18C). In vitro and in vivo data support the conclusion that resistance of CD133⁺ colorectal CSCs to metformin is primarily mediated by histone lactylation rather than acetylation (Fig. S18D–S18H, S19).

Notably, under HG conditions, metformin further increased histone lactylation, whereas c-JUN and CD36 levels did not increase accordingly, seemingly inconsistent with a lactylation-driven mechanism. To address this, dose-response experiments demonstrated that metformin and exogenous lactate increased pan-Kla, H3K18la, and c-JUN/CD36 expression in a concentration-dependent manner, in parallel with rising extracellular lactate levels (Fig. S20A, S20B). However, when lactate exceeded ~5 mM, c-JUN and CD36 expression reached a plateau, indicating a saturable effect of lactate-induced histone lactylation on *c-JUN* transcription. Time-course analyses further showed that metformin or lactate rapidly and persistently elevated histone lactylation, whereas c-JUN and CD36 exhibited a defined plateau phase (approximately 6–12 h after metformin treatment; ~18 h under the HG background; and ~3–6 h after lactate treatment); inhibition of lactylation significantly suppressed c-JUN and CD36 expression (Fig. S20C, S20D).

Lipid metabolic phenotypes displayed a similar saturation behavior. TG accumulation, LD storage, and FAO entered a plateau phase after prolonged treatment (Fig. S20E–S20G). Accordingly, at the 24 h endpoint, HG and HG plus metformin groups showed comparable lipid metabolic readouts, whereas inhibition of histone lactylation markedly reduced TG accumulation, LD storage, and FAO. Together, these findings indicate that, under HG conditions, CD133^+^ colorectal CSCs adopt a lactylation-driven metabolic program in which downstream transcriptional and metabolic outputs rapidly approach a plateau and are subsequently maintained, thereby sustaining metabolic adaptation and the metformin-resistant phenotype.

Additionally, to test whether mitochondrial utilization of lactate, via conversion to pyruvate and participation in the NAD⁺/NADH redox cycle-contributes to resistance, we first quantified intracellular NAD⁺/NADH ratios. Metformin-sensitive cells exhibited markedly lower ratios than resistant cells, yet exogenous lactate did not significantly raise this ratio in sensitive cells despite promoting resistance (Fig. S21A). We next used two complementary interventions. α-Ketobutyrate (α-KB), which regenerates NAD⁺ from NADH to mimic lactate oxidation, neither attenuated metformin-induced apoptosis nor altered drug sensitivity (Fig. S21B–S21E). Likewise, despite effective inhibition of mitochondrial pyruvate import by the MPC inhibitor UK5099 (Fig. S21F), UK5099 had no significant effect on IC₅₀ values or apoptosis under either LG or HG conditions (Fig. S21G, S21H). Together, these results demonstrate that lactate-induced metformin resistance occurs independently of lactate oxidation and NAD⁺/NADH homeostasis, and instead primarily relies on lactate-driven epigenetic regulation, specifically H3K18la, which promotes *c-JUN* transcription and consequently upregulates CD36 expression.

### Targeting LDHA attenuates lactylation-dependent resistance of CD133⁺ colorectal CSCs to metformin

LDHA is a key enzyme involved in lactate production and has been demonstrated to promote protein lactylation^48^. As shown above, LDHA regulates metformin resistance in CD133⁺ colorectal CSCs under HG conditions (Fig. 5I–L). On this basis, we hypothesized that combining metformin with LDHA targeting could reduce the proportion of CD133⁺ CSCs in CRC.

**Fig. 8:**
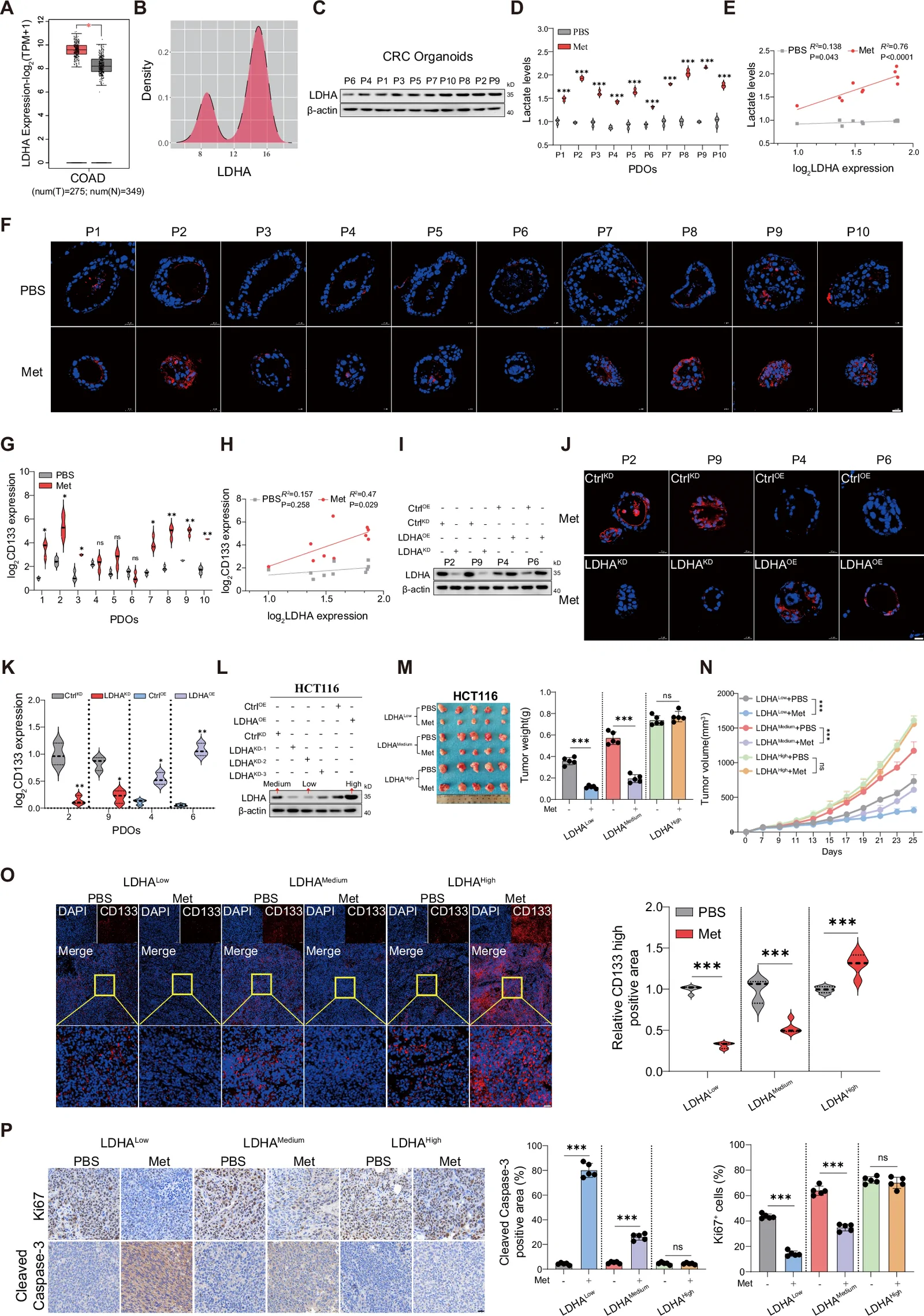
Targeting LDHA attenuates lactylation-dependent resistance of CD133⁺ colorectal CSCs to metformin. **A** LDHA expression in colon adenocarcinoma (COAD) tumor tissues (red) and normal tissues (gray) was analyzed using GEPIA based on TCGA and GTEx datasets. Expression levels are shown as log₂ (TPM + 1). The center line indicates the median, the box bounds indicate the 25th and 75th percentiles, the whiskers extend to 1.5 × IQR, and dots represent individual samples. Tumor, *n* = 275 samples; normal, *n* = 349 samples. *P < 0.01. **B** Kernel density plot showing the distribution of LDHA expression levels in CRC tissues. **C** Western blot analysis of LDHA expression in samples from 10 CRC PDOs (P1-P10). LDHA and β-actin were detected on separate blots. The samples derive from the same experiment, and the blots were processed in parallel. **D** Lactate production in 10 CRC PDO lines before and after Met treatment. **E** Correlation analysis between lactate production and LDHA expression across the 10 CRC PDO lines. Measurements were performed in three independent biological replicates per PDO line. **F** Immunofluorescence staining showing CD133 expression in 10 PDOs from CRC following Met treatment. **G** Relative quantification of CD133 expression in 10 CRC PDO lines before and after Met treatment, based on immunofluorescence analysis. **H** Correlation analysis between CD133 expression and LDHA expression across the 10 CRC PDO lines. Measurements were performed in three independent biological replicates per PDO line. **I** Western blot analysis showing LDHA expression in P2 and P9 PDOs following LDHA knockdown, and in P4 and P6 PDOs following LDHA overexpression. LDHA and β-actin were detected on the same blot, with β-actin serving as the loading control. **J** Immunofluorescence images show the expression of CD133 following Met treatment in PDOs subjected to LDHA expression induction in **I**. **K** Relative quantification of CD133 expression was performed. **L** Western blot analysis of LDHA expression in CSCs transfected with vector (Ctrl^OE^), LDHA overexpression construct LDHA^OE^, or shRNA constructs targeting LDHA (LDHA^KD-1^, LDHA^KD-2^, LDHA^KD-3^) and negative control shRNA (Ctrl^KD^). Among these, LDHA^KD-2^ was used to represent low LDHA expression, Ctrl^OE^ as moderate LDHA expression, and LDHA^OE^ as high LDHA expression. LDHA and β-actin were detected on the same blot, with β-actin serving as the loading control. **M** Representative tumor images and final tumor weight quantification in mice engrafted with CD133⁺ CSCs exhibiting low, medium, or high LDHA expression, treated with either PBS or Met (*n* = 5 mice per group). **N** Tumor growth curves over the course of treatment (*n* = 5 mice per group). **O** Immunofluorescence staining of tumor tissues for CD133 expression, with quantitative analysis (*n* = 5 mice per group). **P** Immunohistochemical staining of tumor tissues for Cleaved Caspase-3 and Ki67 expression, with quantitative analysis presented (*n* = 5 mice per group). Data are presented as mean ± SD. Statistical significance was determined by two-sided unpaired Student’s *t*-test for comparisons in **D,** **G** and **K**, and by two-sided Pearson correlation analysis for **E** and **H**. For factorial comparisons in **M,** **O** and **P**, statistical significance was determined by two-sided two-way ANOVA followed by Tukey’s multiple-comparison test. Tumor growth curves in *N* were analyzed by ordinary two-way ANOVA followed by Tukey’s multiple-comparison test. Scale bars, 20 μm for immunohistochemical images (**P**) and immunofluorescence images (**F,** **J** and **O**). Met, metformin; ns, no significance; *P < 0.05; **P < 0.01; ***P < 0.001. Source data are provided as a Source Data file.

We first analyzed LDHA expression in CRC using public datasets and found that it is significantly overexpressed in tumors (Fig. 8A). High LDHA levels were associated with unfavorable outcomes: patients with elevated LDHA had significantly shorter overall survival (OS) (Fig. S22A), and multiple datasets showed a consistent trend toward reduced progression-free survival (PFS)^49–51^. Consistent with prior reports, elevated LDHA has also been linked to diminished therapeutic responsiveness, including chemotherapy resistance across several tumor types, including CRC^52–55^. Additionally, CRC patients could be stratified into LDHA-high and LDHA-low expression subgroups (Fig. 8B). To validate these observations functionally, we established ten PDOs and confirmed inter-sample differences in LDHA expression (Fig. 8C). Under untreated conditions, lactate production capacities were comparable across the PDOs and not correlated with LDHA levels. However, following metformin treatment, PDOs with high LDHA expression showed increased lactate production, with a significant positive correlation between LDHA levels and lactate output (Fig. 8D, E).

We next examined CD133 expression across these PDOs. Among the ten samples, eight showed increased CD133 expression after metformin treatment, while two exhibited no change, likely due to intrinsic heterogeneity (Fig. 8F, G). Correlation analysis revealed no association between CD133 and LDHA levels under basal conditions, but a significant positive correlation emerged after metformin treatment (Fig. 8H). Based on these results, we selected two LDHA-high PDOs (P2 and P9) for LDHA knockdown and two LDHA-low PDOs (P4 and P6) for LDHA overexpression (Fig. 8I). LDHA knockdown in P2 and P9 significantly reduced metformin-induced CD133, whereas LDHA overexpression in P4 and P6 increased CD133 following metformin (Fig. 8J, K).

We then established CD133⁺ colorectal CSCs with distinct LDHA expression levels: low (LDHA^KD^), medium (Ctrl), and high (LDHA^OE^) (Fig. 8L). In vitro, LDHA-high CSCs displayed markedly increased FAO relative to LDHA-low cells (Fig. S22B). TEM revealed more numerous mitochondria with densely packed cristae, and Seahorse FAO assays confirmed preserved oxidative capacity, indicating metabolic compensation despite ETC inhibition (Fig. S22C, S22D). By contrast, lipid peroxidation did not differ among groups (Fig. S22E). To exclude the contribution of lactate oxidation to this phenotype, we treated cells with α-KB, but this intervention neither reversed resistance nor altered apoptosis (Fig. S23A–S23C). Similarly, using UK5099 had no significant effect on IC₅₀ values or apoptosis under either LG or HG conditions (Fig. S23D–S23E). Pharmacologic inhibition of fatty-acid uptake with SMS121 or blockade of FAO with etomoxir partially reversed metformin resistance and increased apoptosis, indicating that LDHA-driven resistance relies on FAO-mediated metabolic compensation. Conversely, in LDHA-low CSCs, NAC suppressed ROS accumulation and partially phenocopied the metabolic and survival features of LDHA-high cells under metformin (Fig. S24A–S24E). These findings, consistent with the results shown in Fig. S21, demonstrate that LDHA-driven metformin resistance depends on FAO-mediated metabolic compensation rather than lactate oxidation or NAD⁺/NADH homeostasis.

In vivo experiments revealed that metformin significantly suppressed tumor growth and reduced CD133-high areas in the LDHA-low and medium groups. In contrast, LDHA-high tumors exhibited resistance to metformin, accompanied by an expansion of CD133-high areas (Fig. 8M–P). In these tumors, interstitial lactate concentrations were markedly elevated, accompanied by increased CD36 and c-JUN expression (Fig. S25A, S25B), consistent with our in vitro findings that the lactate-c-JUN/CD36 axis mediates resistance. Moreover, pan-Kla and H3K18la levels were significantly increased in LDHA-high tumors, but reduced in LDHA-low and medium tumors in response to metformin (Fig. S25C), suggesting that LDHA expression level is a critical determinant of metformin responsiveness in CD133⁺ CSCs.

Finally, to test whether glucose availability influences this mechanism, we transplanted LDHA-high CD133⁺ CSCs into LCD-fed mice and LDHA-low CD133⁺ CSCs into HCD-fed mice. Upon metformin treatment, LDHA-low tumors remained sensitive under HCD, with reduced CD133-high areas, whereas LDHA-high tumors under LCD retained resistance and showed increased CD133-high regions (Fig. S26A–S26E). In these tumors, metformin continued to elevate pan-Kla and H3K18la despite the low-carbohydrate setting. By contrast, in LDHA-low tumors under HCD, metformin did not affect pan-Kla but significantly reduced H3K18la (Fig. S26F). Together, these results indicate that LDHA expression level governs the lactylation status and metformin sensitivity of CD133⁺ colorectal CSCs. Targeting LDHA may represent a promising strategy to overcome metformin resistance in CRC, independent of dietary glucose conditions.

## Discussion

CRC is the third most common cancer worldwide, and recurrence and metastasis remain the leading causes of patient mortality^56^. CSCs possess self-renewal capacity, high tumorigenicity, and intrinsic drug resistance, and thus play crucial roles in therapeutic resistance, metastasis, and recurrence^57^. A comprehensive understanding of CSC biology and the development of targeted strategies to eliminate these cells are therefore essential for achieving durable tumor eradication. Metformin, a potential CSC-targeting agent, may lose efficacy due to metabolic adaptation in CSCs, leading to therapy resistance. Understanding the mechanisms and conditions underlying this resistance is crucial for improving metformin’s therapeutic effectiveness.

In modern society, high-carbohydrate diets, characterized by a high GI and GL, have become a prominent dietary pattern. Such diets can rapidly elevate blood glucose and sustain higher glucose levels over time, thereby continuously supplying glucose to tissues. This dietary context may influence metformin’s antitumor activity, underscoring the importance of assessing how carbohydrate availability shapes treatment outcomes.

Previous studies have shown that glucose restriction enhances the sensitivity of cancer cells to biguanides^24^, and that glucose may be more limiting than oxygen within the tumor microenvironment^58^, providing a metabolic context favorable for metformin’s antitumor effects. However, both in vivo and in vitro studies indicate that metformin can accelerate glucose uptake by cancer cells, increasing intratumoral glucose levels^21,59^. We have observed similar results in our experiments, particularly in HCD-fed and D30-treated mice, where intratumoral glucose levels were higher following metformin treatment (Fig. 1I, K, S1I, S2B). Despite the similar concentrations of metformin in the cytoplasm and culture medium in vitro^60^, the effective concentration of metformin in the microenvironment is much lower, allowing CSCs to continue growing in hyperglycemic mice (Fig. 1E, S2D). Furthermore, unlike bulk differentiated cancer cells that rely on glycolysis, CSCs display unique metabolic phenotypes and may heavily depend on glycolysis or OXPHOS depending on the cancer type^61^. Metformin can selectively induce apoptosis in CD133^+^ CSCs in pancreatic PDAC that depend on OXPHOS, as they cannot switch to glycolysis to cope with subsequent energy crises^11^. Similarly, cholangiocarcinoma CSCs that highly depend on OXPHOS are also effective targets for metformin^10^. However, CSCs are highly heterogeneous and plastic, continually adapting to microenvironmental changes through genetic, epigenetic, and microenvironmental factors. In PDAC, a small subset of CD133^+^ cells exhibit low mitochondrial mass and rely on glycolytic metabolism, rendering them resistant to metformin^11^. This provides another explanation for the negative results observed in clinical trials of metformin for cancer treatment^14,62,63^. In our study, CD133⁺ colorectal CSCs adopted a highly glycolytic phenotype after metformin treatment under both LG and HG. Under LG, however, they could not sustain glucose-dependent energy production or mount adaptive metabolic reprogramming, leading to apoptosis.

Metformin inhibits mitochondrial complex I, thereby perturbing not only glucose metabolism but also other mitochondrial functions—including aspartate supply for biosynthesis, mitochondrial lipid synthesis, and nucleotide homeostasis^64–66^. Accordingly, beyond glucose, it is crucial to consider other metabolites (amino acids and fatty acids) that supply ATP, support redox balance, and provide biomass precursors that can offset metformin’s effects. Our data identify CD36-mediated LD metabolism as a major contributor to metformin resistance. LDs are dynamic organelles that store imported lipids and are integral to cellular stress responses^67^. In acidic environments, autocrine TGF-β2 signaling enhances LD biogenesis, and fatty acids released from LDs undergo FAO to generate ATP and support EMT^68^. Consistent with this, metformin-resistant CSCs display increased LD formation and LD-driven FAO, elevating ATP and NADPH to sustain energy and protect against oxidative stress (Fig. 4A–C and S8C–E, H). Because cancer cells increase FFA uptake and LD formation under acidosis^29,68^ and metformin raises lactate and acidifies media, we tested whether acidosis accounts for LD accumulation. Metformin did not further increase LDs under HG, whereas CD36 knockdown significantly reduced LD formation below PBS controls (Fig. 4B, C), indicating that LD accumulation in resistant cells is CD36-dependent rather than acidosis-driven. CD36 also helps distinguish CSC subsets. In breast cancer, CD36 expression in FFA-avid cells influences stemness markers CD44 and Oct4^69^; in glioblastoma spheres, CD36 co-expresses with CD44⁺, CD133⁺, and ITGA6^70^; and CD36⁺ leukemic stem cells exploit gonadal adipose tissue as a metabolic niche to evade chemotherapy^19^. Moreover, palmitic acid or a high-fat diet specifically enhances the metastatic potential of CD44-bright oral squamous cell carcinomas (OSCCs) in a CD36-dependent manner^20^. Together with our findings, these observations support a central role for CD36-driven LD metabolism in CSC resistance to metformin.

Lactylation is a recently recognized epigenetic modification whose role in cancer resistance is increasingly appreciated. Lactylation of lysine 388 on NBS1 and lysine 673 on MRE11 promotes DNA damage repair, thereby mediating chemoresistance^16,17^. Histone H3K9 lactylation (H3K9la) can activate transcription of LUC7L2, driving retention of intron 7 in MLH1, reducing MLH1 expression, inhibiting the mismatch-repair (MMR) pathway, and ultimately conferring temozolomide resistance in glioblastoma^71^. In our study, metformin-resistant CSCs produced large amounts of lactate under HG (Fig. 5F, H). This lactate activated the transcription factor c-JUN via H3K18la, which in turn upregulated CD36. Although lactate, as a product of the Warburg effect, has been extensively studied, its production in response to metformin has been underappreciated and often regarded merely as a byproduct^72^. Our data indicate that metformin-induced lactate in CSCs can function as a resistance factor, warranting further investigation, and offering a potential explanation for negative clinical trial outcomes with metformin in oncology. LDHA is a key enzyme in lactate production; notably, lactate generated in the nucleus can be directly converted to lactyl-CoA, mediating histone lactylation^48^. In our experiments, reducing LDHA expression in CD133⁺ CSCs effectively prevented the development of metformin resistance independent of glucose levels. Thus, LDHA may represent a promising combinatorial target to enhance metformin-based cancer therapy.

While our data suggest that CD133⁺ CSC expansion under HG is driven mainly by selective survival after metabolic adaptation, distinguishing this from potential dedifferentiation will require lineage tracing. Moreover, although we identify histone lactylation as an epigenetic link between LDHA-derived lactate and c-JUN/CD36 activation, contributions from other acyl marks or parallel metabolic pathways cannot be fully excluded. Future studies should incorporate lineage tracing and directly test the causal sufficiency of lactylation in driving these transcriptional and phenotypic changes.

**Fig. 9:**
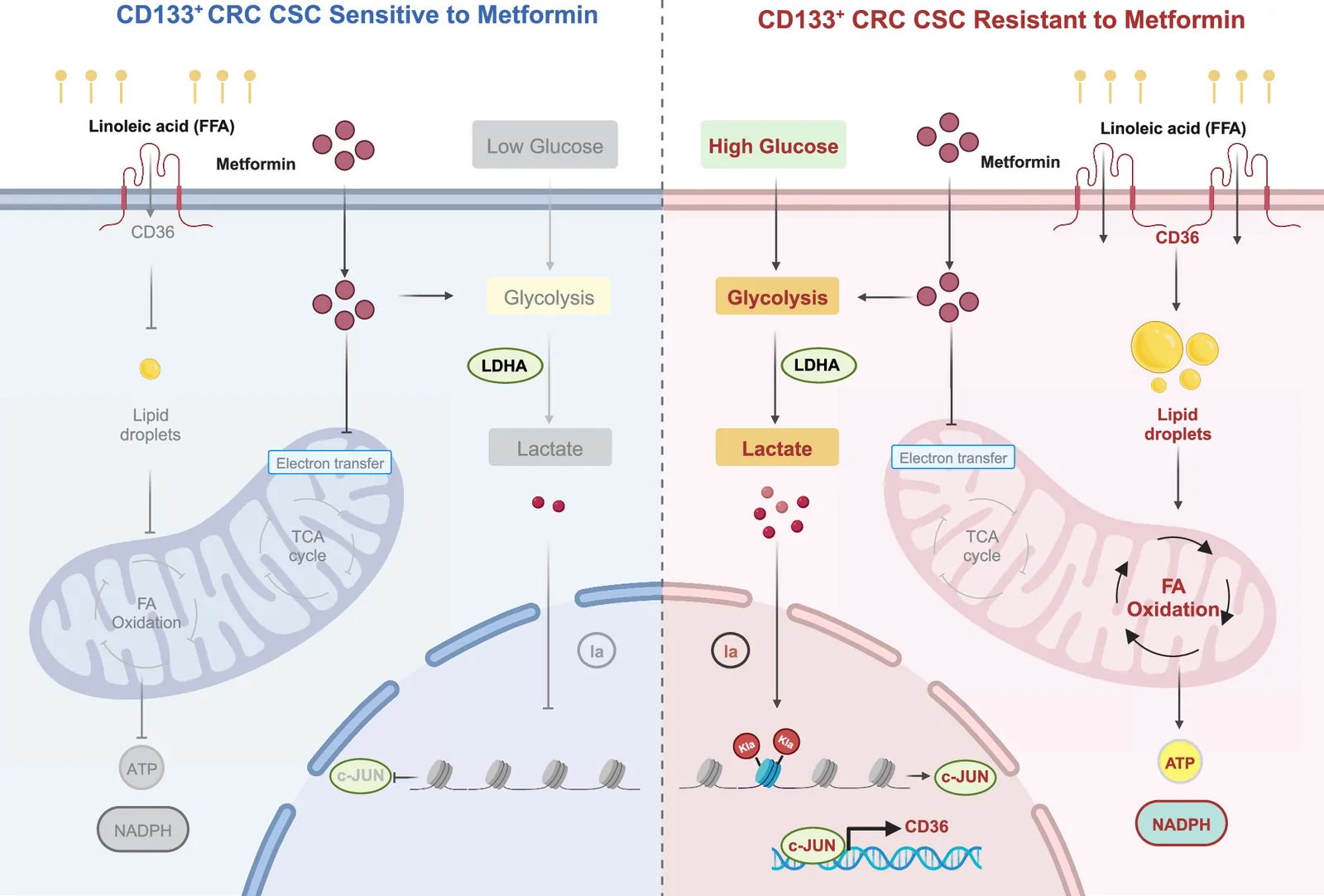
Schematic diagram of the mechanism underlying metformin resistance in CD133⁺ colorectal CSCs. Under high-glucose conditions, metformin induces LDHA-dependent lactate production in CD133⁺ colorectal CSCs. Lactate enhances histone H3K18 lactylation, thereby activating c-JUN and upregulating CD36. CD36 promotes fatty-acid uptake and lipid-droplet (LD) biogenesis. LD-derived fatty acids are mobilized for β-oxidation to generate ATP and NADPH, alleviating energy stress and limiting ROS accumulation and oxidative stress. This lactate–H3K18la–c-JUN–CD36 axis drives lipid metabolic adaptation and ultimately confers resistance to metformin. Targeting LDHA or CD36 restores metformin sensitivity and enhances its antitumor efficacy against CD133⁺ colorectal CSCs. Created in BioRender. Li, X. (2026) https://BioRender.com/2qboh1g.

In summary, under HG conditions, metformin-treated CD133⁺ colorectal CSCs generate lactate via LDHA. Lactate activates c-JUN through H3K18la, which in turn upregulates CD36. CD36 enhances FFA uptake and LD biogenesis; FFAs stored in LDs are mobilized and subsequently undergo β-oxidation to produce ATP and NADPH, alleviating energy and oxidative-stress crises and thereby conferring metformin resistance (Fig. 9). This work extends the focus from glucose metabolic reprogramming to lipid metabolic adaptation, revealing the pronounced metabolic plasticity of CD133⁺ CSCs under metabolic therapy. We identify LDHA and CD36 as critical mediators of metformin resistance in CSCs, underscoring their value as therapeutic targets. More broadly, effective CSC-directed strategies will need to simultaneously disrupt metabolic adaptation to improve efficacy and overcome resistance.

## Methods

### Animals

Female BALB/c nude mice and BALB/c mice (4–6 weeks old) were purchased from Beijing Vitalstar Biotechnology Co., Ltd. All animals were maintained under specific pathogen-free (SPF) conditions under a 12-h light/12-h dark cycle, at 20–26 °C and 40–70% relative humidity, and cared for in accordance with Association for Assessment and Accreditation of Laboratory Animal Care International guidelines. All experimental procedures were approved by the Animal Ethics Committee of Beijing Shijitan Hospital, Capital Medical University (approval No. sjtkyll-lx-2021(39)). Standard breeding chow was obtained from Beijing Vitalstar Biotechnology Co., Ltd. The macronutrient composition of the high-carbohydrate diet (HCD) and low-carbohydrate diet (LCD) is summarized in Table 1. Mice had ad libitum access to food and water throughout the study unless otherwise indicated. To modulate baseline glycemia, mice were preconditioned for 2 weeks on either HCD or LCD prior to tumor cell inoculation, and the assigned diets were maintained throughout the experiment. In designated experiments, hyperglycemia was induced by providing 30% dextrose water (D30) ad libitum beginning 3 weeks before implantation and continuing throughout the study. Following dietary and drinking-water interventions, random tail-vein blood samples were collected and glucose was measured using a FreeStyle glucometer with FreeStyle test strips.

**Table 1:** Composition of high-carbohydrate and low-carbohydrate diets

Composition	High Carbohydrate		Low Carbohydrate	
	gm%	kcal/%	gm%	kcal/%
Protein	15	14	51	50
Carbohydrate	52	66	24	30
Fat	9	20	9	20
Total		100		100
kcal/gm	4.1		4.1	
Ingredient	gm	kcal	gm	kcal
Animal Protein	142	568	501	2004
100% Amylopectin (amioca Starch)	316	1264	140	560
Sucrose	180	720	80	320
Fructose	150	600	67	268
Dietary Fiber	50	0	50	0
Vegetable Oil	90	810	90	810
TBHQ	0.008	0	0.014	0
Mineral mixture	35	0	35	0
Vitamin mixture	10	40	10	40
Choline Bitartrate	2	0	2	0
Total	975.008	4002	975.014	4002

### Tumor growth and treatment

For flank xenografts, 5 × 10⁶ HCT116 or SW620 cells in 200 μL PBS were subcutaneously injected into the right flank of each mouse. For the syngeneic model, 5 × 10⁶ CT26 cells in 200 μL PBS were injected similarly. Tumor treatment was initiated when tumors became palpable (approximately 120–150 mm³). Mice assigned to the metformin group received 200 mg/kg intraperitoneally every 2 days.

To investigate the role of lipid droplets (LDs) in metformin resistance of CD133⁺ colorectal cancer stem cells, mice were treated with a DGAT1 inhibitor (3 mg/kg, 15% Captisol, oral gavage, every 2 days) to inhibit LD biogenesis, or an ATGL inhibitor (10 mg/kg, olive oil vehicle, oral gavage, every 2 days) to block LD mobilization. To assess the role of lactate, sodium lactate (1.68 g/kg, i.p., every 2 days) was administered alone or in combination with α-cyano-4-hydroxycinnamic acid (α-CHCA; 25 mg/kg, i.p., every 2 days). Where indicated, mice additionally received SP600125 (10 mg/kg, i.p.), etomoxir (20 mg/kg, i.p.), SMS121 (10 mg/kg, i.p.), sodium acetate (NaAc; 200 mM in drinking water), and/or C646 (5 mg/kg, i.p., three times per week) according to experimental design. For ROS modulation, mice were treated with N-acetylcysteine (NAC; 150 mg/kg, i.p.) alone or in combination with metformin on the same dosing schedule.

Tumor progression was monitored every 2 days by measuring tumor length and width using digital calipers, and tumor volume was calculated as (length × width²)/2. Animals were regularly monitored for general health status and signs of distress. Experimental endpoints were defined according to predefined study criteria and institutional animal welfare guidelines. Mice were euthanized at the planned experimental endpoint or earlier if humane endpoint criteria were met. Humane endpoints included tumor ulceration or necrosis, impaired mobility, body weight loss exceeding 20% of baseline, severe lethargy, persistent hunched posture, or other signs of significant distress. The maximum tumor volume permitted by institutional guidelines was 2000 mm³, and this limit was not exceeded in any animal included in the study.

### Cell lines

HCT116, SW620, and HCT15 cell lines, obtained from the American Type Culture Collection (ATCC), were cultured in RPMI 1640 medium supplemented with 10% fetal bovine serum and 1% penicillin-streptomycin. All cultures were maintained at 37 °C in a humidified incubator with 5% CO_2_. For experiments requiring different glucose concentrations, glucose-free RPMI 1640 medium was used to prepare medium containing 5.5 mM and 25 mM glucose. Metformin treatment was administered at a concentration of 10 mM for a duration of 24 h.

### Patient-derived organoids

This study followed the Declaration of Helsinki guidelines and was approved by Ethics Committee of Beijing Shijitan Hospital, Capital Medical University. Participants gave written informed consent before joining. A total of 10 patients with colorectal cancer were enrolled, including 6 males and 4 females, with an age range of 47–71 years. Sex/gender information was recorded based on self-report provided by the participants at the time of enrollment. No specific sex or gender criteria were applied for inclusion. No financial compensation was provided to the participants.

OrganoGel, Organoid construction and culture kits were sourced from Shanghai Youningwei Biotechnology Co., Ltd. (abs9495, abs9445). The detailed steps are outlined below: (1) Sample Preparation: Colorectal cancer samples from patients were placed in a culture dish, photographed, and recorded with details including size, color, consistency, and tissue type. (2) Initial Treatment: The samples were immersed in 2–3 mL of primary culture buffer within a 60 mm cell culture dish. After three washes with the same buffer, the tissue was minced into approximately 1–3 mm³ fragments and transferred to a 15 mL centrifuge tube. (3) Digestion Process: Human colorectal cancer primary tissue digestion solution was added to the 15 mL centrifuge tube, and the mixture was incubated at 37 °C for 7 min. Digestion was stopped by adding three times the volume of primary culture buffer once numerous single cells or cell clusters smaller than 70 μm were observed under a microscope. (4) Filtration and Centrifugation: The digest was filtered through a 100 μm sieve, centrifuged at 300 × g, 5 min, 4 °C, and the supernatant was discarded. The pellet was resuspended in about 1 mL of primary culture buffer and transferred to a 1.5 mL Eppendorf tube for another centrifugation. (5) Matrix Formation and Culturing: The pellet was resuspended in matrix gel, plated, and allowed to gelatinize in a 37 °C incubator for 10–15 min before adding 500 μL of human colorectal cancer organoid culture medium. The medium was replaced every 2 days, and organoids were passaged every 10 days using mechanical and enzymatic disruption. For the metformin treatment group, 2 mM metformin was added 3–4 days post-seeding of the organoids, and continuous treatment was administered for 5 days. The size and number of organoids were then observed and recorded under a microscope.

### Western Blot

Cell and tumor tissue lysates were prepared using RIPA buffer on ice to extract proteins. Protein concentrations were quantified using a BCA Protein Assay Kit (Boster Biological Technology). Proteins were then separated on a 10% SDS-PAGE gel and electroblotted onto a PVDF membrane. For high-molecular-weight proteins such as p300, an 8% SDS-PAGE gel was used. Membranes were blocked with 5% non-fat milk in TBST for 1 h at room temperature, followed by overnight incubation at 4 °C with primary antibodies. After washing three times with TBST, the membranes were exposed to HRP-conjugated secondary antibodies for 1 h at room temperature. Protein bands were visualized with an enhanced chemiluminescence detection system. Subsequent densitometric quantification of the immunoblots was performed using Image Lab software provided by Bio-Rad.

### Quantitative PCR analysis of mRNA expression

Total RNA was extracted and purified from cells using total RNA extraction kit (TIANGEN Biotech), and cDNA was synthesized using the FastKing RT Kit (TIANGEN Biotech) according to the manufacturer’s protocol. qPCR was conducted using the primers listed in Table S2, employing 2 × PowerUp SYBR Green Master Mix from Applied Biosystems and quantified using QuantStudio™ Real-Time PCR Software. The thermal cycling conditions were set at 50 °C for 2 min, 95 °C for 2 min, followed by 40 cycles of 15 s at 95 °C and 1 min at 60 °C. The ΔΔCT data were analyzed using QuantStudio Design & Analysis Software v1.5.1.

### Flow cytometry

To assess CD133^+^ cell populations, treated cells were stained with anti-CD133-PE and incubated for 30 min at 4 °C in the dark. After washing off excess antibody, cells were resuspended in PBS and analyzed using flow cytometry. Data from all experiments were quantitatively analyzed using FlowJo software.

### Measurement of intratumoral glucose levels in mice

To measure glucose concentrations in tumor tissues, samples were initially collected, rapidly frozen in liquid nitrogen, and stored at −80 °C. Tissues were thawed and homogenized using Beyotime’s E6600 TissueMaster™ and glucose detection lysis buffer at a 1:10 weight-to-volume ratio. After homogenization, the samples were centrifuged at 12,000 × *g* for 5 min, and the supernatant was collected for glucose measurement. For the assay, 5 µL of supernatant was mixed with 185 µL of glucose assay reagent and heated at 95 °C for 8 min. Absorbance was measured at 630 nm, and glucose concentrations in the tissue were calculated based on a standard curve.

### Magnetic bead sorting of CD133^+^ cells

CD133⁺ cells were isolated from SW620 and HCT15 cells using a CD133 MicroBead Kit (Miltenyi Biotec, 130-097-049). Following the manufacturer’s protocol, cells were labeled and applied to a MACS Column in a MACS Separator. CD133^+^ cells were magnetically retained, while unlabeled cells were removed. Post-separation, the CD133^+^ fraction was eluted from the column, providing a purified cell population.

### Colony formation assay

CD133⁺ cells were isolated by magnetic-activated cell sorting (MACS) and treated with metformin (10 mM) for 24 h under LG or HG conditions. Following treatment, 1500 cells were seeded per well in 6-well plates containing complete RPMI-1640 with 10% FBS and cultured for 7–10 days until visible colonies formed. At endpoint, colonies were washed with PBS, fixed with 4% paraformaldehyde for 15 min, and stained with 0.1% crystal violet for 30 min. After washing and air-drying, colonies were quantified using ImageJ.

### Sphere-formation assay

CD133⁺ cells were isolated by MACS (Miltenyi Biotec) and pretreated with metformin (10 mM) for 24 h under LG or HG conditions before spheroid culture. Single-cell suspensions were seeded at 1,000 cells/well in 6-well ultra-low attachment plates in serum-free DMEM/F12 (Gibco, 11320033) supplemented with B27 (Gibco, 17504044), bFGF 20 ng/mL (R&D, 233-FB), and EGF 20 ng/mL (Gibco, PHG0313). Cultures were maintained at 37 °C, 5% CO₂, with medium refreshed every 2 days. Sphere formation was monitored for 1–2 weeks; spheres >50 µm in diameter were counted under an inverted microscope.

### Cell viability and apoptosis assay

Cell viability was assessed using a CCK-8 assay kit (LABLEAD, CK001). Cells were seeded in 96-well plates at 2000–5000 cells per well and allowed to attach overnight. For dose–response assays, cells were treated with increasing concentrations of metformin for 24 h, and IC₅₀ values were calculated by nonlinear regression using GraphPad Prism. For fixed-dose functional assays, cells were treated with metformin at 10 mM for 24 h unless otherwise indicated. After treatment, 100 μL of CCK-8 working solution was added to each well, and absorbance at 450 nm was measured after incubation for 1 h at 37 °C.

For apoptosis analysis, post-treatment cells were collected and double-stained with Annexin V-FITC/PI using an apoptosis analysis kit (LABLEAD, C1002). Briefly, cells were washed twice with cold PBS, resuspended in binding buffer, incubated with 10 μL Annexin V-FITC and 5 μL PI for 15 min at room temperature in the dark, and analyzed by flow cytometry. Annexin V-positive cells, including early and late apoptotic cells, were used for quantification.

For standard CRC chemotherapeutic controls, cells were treated under LG or HG conditions with 5-fluorouracil (5-FU; 10 μM for HCT116 and 20 μM for SW620), capecitabine (CAPE; 10 μM for HCT116 and 5 μM for SW620), irinotecan (CPT-11; 2 μM for HCT116 and 2 nM for SW620), or oxaliplatin (L-OHP; 2 μM for HCT116 and 1 μM for SW620), followed by CCK-8 viability and Annexin V-FITC/PI apoptosis assays.

For mechanistic perturbation experiments, cells were treated with exogenous lactate (Lac; 10 mM, 24 h), sodium acetate (NaAc; 10 mM, 24 h), the p300 inhibitor C646 (10 μM, 24 h), the JNK inhibitor SP600125 (10 μM, 24 h), α-ketobutyrate (α-KB; 10 mM, 24 h), N-acetylcysteine (NAC; 5 mM, 24 h), the mitochondrial pyruvate carrier inhibitor UK5099 (10 μM, 24 h), the monocarboxylate transporter inhibitor α-cyano-4-hydroxycinnamic acid (α-CHCA; 5 mM, 24 h), the DGAT1 inhibitor A922500 (DGAT1i; 10 μM, 24 h), or the ATGL inhibitor HY-15859 (ATGLi; 20 μM, 24 h), as indicated. For inhibitor-combination experiments, inhibitors were added 1 h before metformin treatment and maintained throughout the 24-h metformin exposure unless otherwise stated. For CD36 blockade, cells were treated with the CD36 inhibitor SMS121 (10 μM, 24 h) or the CD36-blocking antibody JC63.1 (10 μg/mL, 24 h). Vehicle-treated cells were included as controls in all experiments.

For CD36, c-JUN, or LDHA gain- or loss-of-function experiments, the indicated genetically modified HCT116 or SW620 CD133⁺ CSCs were seeded and treated under the same conditions described above, followed by CCK-8 viability or Annexin V-FITC/PI apoptosis analysis. For experiments involving exogenous LA, cells were supplemented with LA at 50 μM as indicated.

### Tissue immunohistochemistry and immunofluorescence

Tissue samples were fixed in formalin, embedded in paraffin, and subjected to immunohistochemical staining to evaluate cell proliferation and apoptosis using antibodies against Ki-67 (Proteintech, 28074-1-AP, 1:1000) and Cleaved Caspase-3 (Proteintech, 25128-1-AP, 1:5000), respectively. CD133⁺ CSC regions were identified by staining with an anti-CD133 antibody (Cell Signaling Technology, 64326, 1:200). To assess lactylation levels, pan-lactylation (PTM BIO, PTM-1401RM, 1:100) and H3K18 lactylation (PTM BIO, PTM-1406RM, 1:100) were detected; histone H3K18 acetylation (H3K18ac) was detected using an anti-H3K18ac antibody (Abcam, ab1191, 1:200). Primary antibody incubations were followed by staining with Alexa Fluor 555-conjugated secondary antibody (Cell Signaling Technology, #4413, 1:500).

### Cell and organoid immunofluorescence

For immunofluorescence staining of CD133, HCT116, SW620, and HCT15 cells were fixed with 4% paraformaldehyde, stained overnight with an anti-CD133 primary antibody (Cell Signaling Technology, 64326, 1:200), followed by staining with Alexa Fluor 555-conjugated secondary antibody (Cell Signaling Technology, #4413, 1:500). Nuclei were stained with DAPI and imaged using a fluorescence microscope (ECLIPSE NI, Nikon). For paraffin-embedded organoid sections, after deparaffinization and antigen retrieval with citric acid buffer, the same staining protocol was applied. The sections were then imaged with a confocal microscope (SIM, Nikon) to visualize the results.

### Cell bioenergetic assays

The Seahorse Extracellular Flux (XF24) Analyzer (Agilent Technologies, XF24) was used to assess the OCR, ECAR, and cell energy phenotype of cancer cells using the respective Agilent test kits (XF Cell Mito Stress Test, Seahorse XF Glycolytic Rate Assay, XF Cell Energy Phenotype Test Kit). Cells were seeded in a Seahorse XF cell culture microplate at densities of 3 × 10^4^ (HCT116) and 2.5 × 10^4^ (SW620 CD133^+^) per well and treated with metformin in either 5.5 mM or 25 mM glucose medium for 24 h. Sensor cartridges were hydrated overnight in Seahorse XF calibrant at 37 °C in a non-CO_2_ incubator. The following day, Seahorse XF RPMI Base Medium was supplemented as required and warmed to 37 °C. After removing the growth medium, cells were washed twice and incubated with 500 μL of assay medium at 37 °C for 1 h before measurements. Reagents from the test kits were reconstituted in the assay medium as per instructions and analyzed on the Seahorse XF24 Analyzer.

For the XF Cell Mito Stress Test, basal respiration was calculated as the baseline OCR (prior to inhibitor injections) after subtracting non-mitochondrial respiration (OCR remaining after antimycin A/rotenone). Maximal respiration was defined as the FCCP-stimulated OCR minus non-mitochondrial respiration. For the Seahorse XF Glycolytic Rate Assay, basal glycolytic rate was defined as the glycolytic proton efflux rate (glycoPER) measured under baseline conditions before mitochondrial inhibition, whereas compensatory glycolytic rate was defined as glycoPER measured after rotenone/antimycin A injection, reflecting glycolytic upregulation when mitochondrial respiration is blocked. Rate parameters were calculated using Wave software (Agilent) with the assay default calculation windows.

Mitochondrial fatty acid oxidation capacity was further analyzed using the XF Long Chain Fatty Acid Oxidation Stress Test Kit following the manufacturer’s protocol. Briefly, 8 × 10^4^ HCT116 cells were pre-incubated overnight in substrate-limited RPMI 1640 medium containing 0.5 mM glucose, 1 mM glutamine, and 0.5 mM carnitine to promote fatty acid–dependent respiration. On the day of the assay, the medium was replaced with fresh RPMI 1640 assay medium containing the same supplements, and cells were equilibrated for 45–60 min in a non-CO₂ incubator at 37 °C. The OCR was recorded after sequential injections of PBS or etomoxir (40 μM, CPT1 inhibitor), oligomycin (1 μM), FCCP (1 μM), and antimycin A/rotenone (0.5 μM each). FAO-dependent respiration was calculated as the etomoxir-sensitive OCR fraction.

Definition of FAO OCR metrics. Basal FAO respiration was defined as the etomoxir-sensitive OCR under basal conditions (PBS minus etomoxir) before mitochondrial stressors were injected. Acute FAO response was defined as the immediate decrease in OCR upon etomoxir injection compared with the PBS control at the basal phase. Maximum FAO respiration was defined as the etomoxir-sensitive OCR under FCCP-stimulated conditions (PBS minus etomoxir) following FCCP injection.

After each assay, wells were immediately washed with PBS and subjected to normalization using a CCK-8–based viability assay. Absorbance was measured at 450 nm using a BioTek Cytation 5 multimode reader to quantify relative cell number. OCR and ECAR values were normalized to cell viability (absorbance per well) before statistical analysis.

### NADPH and ATP assay

NADPH was measured using a NADPH Assay Kit (Abbkine, KTB1010), and ATP was measured using an ATP Assay Kit (Beyotime, S0027), according to the manufacturers’ instructions. For NADPH, around 1 × 10^6^ cells were lysed in 100 µL NADPH extraction buffer, heated at 60 °C for 5 min, and then treated with 20 µL Assay Buffer and 100 µL NADP Extraction Buffer. After vortexing and centrifuging at 14,000 rpm for 5 min, the supernatant’s absorbance at 450 nm was read on a plate reader before and after a 30-min room temperature incubation. For ATP, cells were lysed in 6-well plates, centrifuged at 4 °C and 12,000 × *g* for 5 min, and the supernatant’s luminescence was measured on a plate reader against a standard curve to determine ATP levels.

### Measurement of NAD⁺/NADH Ratio

The intracellular NAD⁺ and NADH contents were measured using a commercial NAD⁺/NADH assay kit (Abbkine, KTB1020) following the manufacturer’s instructions. Briefly, cells were collected after treatment, lysed on ice, and centrifuged at 12,000 × *g* for 10 min at 4 °C. The supernatant was divided into two portions for separate quantification of total NAD (NAD⁺ + NADH) and NADH after heat inactivation of NAD⁺. Absorbance was read at 450 nm using a BioTek Cytation 5 multimode reader, and the NAD⁺/NADH ratio was calculated according to the standard curve provided with the kit.

### L-lactate assay

Lactate concentrations in cells and tumor tissues were measured using the L-Lactate Assay Kit (Colorimetric/Fluorometric) (Abcam, ab65330) following the manufacturer’s instructions. For cellular assays, HCT116 and SW620 CD133⁺ cells were cultured in 5.5 mM or 25 mM glucose medium, treated with metformin for 24 h, harvested, and homogenized in Lactate Assay Buffer. After centrifugation, the supernatant was deproteinized using perchloric acid/KOH to eliminate residual lactate dehydrogenase activity. Samples were then incubated with the reaction mix for 30 min at room temperature, and absorbance was measured at 570 nm using a BioTek Cytation 5 reader. For extracellular lactate, aliquots of conditioned medium from the same wells were collected before harvest and assayed in parallel; lactate values were then normalized to cell viability measured by CCK-8 in the same wells immediately after medium collection (BioTek Cytation 5). For intracellular lactate, assays were initiated with equal numbers of cells per condition to control for cell-count differences at the outset.

For measurement of intratumoral lactate, approximately 10–30 mg of freshly excised tumor tissue was washed in cold PBS and homogenized in 4–6 volumes of Lactate Assay Buffer on ice. After centrifugation at 12,000 × *g* for 5 min at 4 °C, the supernatant was deproteinized with PCA/KOH, and lactate levels were measured under the same assay conditions as above. Lactate concentrations were calculated from a standard curve and normalized to tumor weight.

### ROS detection

ROS production was measured using the ROS Assay Kit (Beyotime, S0033). After the indicated treatments, cells were incubated with 10 μM DCFH-DA at 37 °C for 20 min. Fluorescence was then quantified using a fluorescence spectrophotometer to assess ROS levels.

### Triglyceride quantification

Intracellular triglyceride levels were measured using a TG detection kit (Beyotime, S0219S) according to the manufacturer’s instructions. Briefly, cells were lysed and the supernatants were incubated with the working reagent at 37 °C for 30 min in the dark. Fluorescence intensity was measured at Ex/Em = 540/590 nm using a microplate reader (BioTek), and TG concentrations were calculated based on a standard curve.

### FAO assay

Fatty acid oxidation activity was determined using a Fatty Acid Oxidation Assay Kit (Elabscience, E-BC-K784-M), following the manufacturer’s protocol. Briefly, cells were harvested, washed twice with cold PBS, and lysed on ice. After centrifugation, supernatants were collected and incubated with the reaction working solution at 37 °C for 30 min. The absorbance was measured at OD = 450 nm using a microplate reader (BioTek). FAO activity was normalized to total protein concentration, and results were expressed as relative activity compared with the control group.

### Lipid peroxidation assay

Lipid peroxidation was assessed using the BODIPY™ 581/591 C11 fluorescent probe (Beyotime, S0043S), according to the manufacturer’s protocol. Briefly, cells were incubated with 2 µM BODIPY 581/591 C11 at 37 °C for 30 min in the dark, washed twice with PBS, and immediately analyzed by flow cytometry (BD LSRFortessa). The fluorescence shift from red (oxidized, ~590 nm) to green (~510 nm) was used to quantify the degree of lipid peroxidation. Data were processed using FlowJo software.

### Oil red O staining

To assess the effect of CD36 overexpression or knockdown on LD formation, cells following lentiviral transduction were seeded in 6-well plates and treated with LA with or without a DGAT1i for 24 h. Cells were washed with PBS, fixed in 4% paraformaldehyde for 20 min, and stained with Oil Red O (3 mg/mL in 60% isopropanol) for 30 min. For Oil Red O staining of tumor tissues, tumors were harvested from mice treated as indicated (including metformin alone or in combination with DGAT1i and/or ATGLi). Fresh tumor specimens were embedded in OCT and cryosectioned. Frozen sections were fixed in 4% paraformaldehyde and stained with Oil Red O. Nuclei were counterstained with hematoxylin, and images were acquired by bright-field microscopy (Eclipse Ti, Nikon). Fiji (ImageJ) was used to quantify LD and nuclear areas to assess lipid accumulation.

### Lentiviral vector infection, short interfering RNA (siRNA) transfection and stable cell line construction

In this study, lentiviral vectors for CD36^OE^ and CD36^KD^, LDHA^OE^ and LDHA^KD^, and c-JUN^OE^ and c-JUN^KD^, were obtained from Shanghai Genechem Co., Ltd. HCT116 and SW620 CD133^+^ cells were infected according to the manufacturer’s instructions. Protein samples were collected 72 h after transduction for western blot analysis. For stable cell line selection, cells were cultured in medium containing 2 µg/mL puromycin for 48 h post-infection, enabling the isolation of single-cell clones with uniform and stable expression. LDHA and GPR81 siRNA transfections were conducted using GenMute siRNA Transfection Reagent (SignaGen Laboratories) once the cells reached 30–50% confluence. siRNA gene knockdown validation was determined 72 h after siRNA transfections. The siRNAs were sourced from RiboBio Company (Guangzhou, China), with sequences listed in Table S3.

### Organoid lentivirus transfection

Organoids were dissociated into single cells and seeded into 24-well plates at a density of approximately 2 × 10⁵ cells per well. The medium was replaced with 1 mL of fresh organoid culture medium containing 6 μg/mL polybrene, followed by the addition of viral supernatant. Cells were incubated at 37 °C for 24 h. Loosely attached cells were then gently resuspended, pooled, and centrifuged at 300 × *g* for 5 min at 4 °C. The resulting cell pellet was resuspended in organoid passaging buffer and transferred to a 1.5 mL microcentrifuge tube for a second centrifugation. Based on the estimated tissue volume, Matrigel was added at a 1:25 ratio, and 25 μL of the mixture was plated per well onto a pre-chilled 24-well plate. After gelation at 37 °C for 10–15 min, 500–750 μL of pre-warmed organoid culture medium was added to each well. Once organoid proliferation was observed (typically after 2–3 days), selection was carried out using organoid culture medium containing puromycin to enrich successfully transduced organoids.

### Transmission electron microscopy (TEM)

Cells were fixed in 2.5% glutaraldehyde at 4 °C overnight, post-fixed with 1% osmium tetroxide, dehydrated through a graded ethanol series, and embedded in EPON 812 resin. Ultrathin sections (70–80 nm) were cut with an ultramicrotome, stained with uranyl acetate and lead citrate, and examined using a transmission electron microscope (JEOL JEM-1400) operating at 80 kV. Images were captured digitally, and mitochondrial morphology and LDs numbers were quantified using ImageJ.

### ChIP assay

ChIP assays were conducted using a ChIP kit (Cell Signaling Technology, #9003S). HCT116 cells were cultured in either 5.5 mM or 25 mM glucose medium for 24 h, fixed with formaldehyde, and then prepared for ChIP by quantifying to 1 × 10^6^ cells per sample. Following immunoprecipitation, the purified DNA served as templates for standard PCR. The PCR products were electrophoresed on a 1% agarose gel and visualized under UV light using a FluorChem FC3 system (ProteinSimple, San Jose, CA, USA). Primer sequences utilized are detailed in Table S4.

### Cloning and DNA construction

The CD36 promoter was amplified from HCT116 genomic DNA through PCR with specific primers (see Table S5) and then cloned into the pGL3-Basic Vector (Promega, Madison, WI, USA) at the Kpn I and EcoRI restriction sites. To introduce point mutations into the CD36 promoter, the MutExpress II Fast Mutagenesis Kit V2 (Vazyme, China) was employed according to the provided protocol. Details of the mutagenic primer sequences are available in Table S5.

### Dual luciferase reporter assays

To explore the impact of lactate and c-JUN on the activity of the CD36 promoter, cells underwent transfection with reporter plasmids coupled with relevant experimental plasmids or siRNAs. Forty-eight hours aftertransfection, the cells were lysed and the activities of both firefly and Renilla luciferases were assessed following the manufacturer’s guidelines. Replicates of each experiment were conducted three times using a TriStar LB941 multimode microplate reader (Berthold Technologies, Bad Wildbad, Germany) to ensure reliability and accuracy of the results.

### CUT&Tag assay

The CUT&Tag assay was performed using the Vazyme Hyperactive In-Situ ChIP Library Prep Kit (TD902), following the manufacturer’s instructions. Initially, 1 × 10^6^ treated cells were exposed to ConA beads for 10 min at room temperature. Cells were then incubated with Histone H3K18la (PTM BIO, PTM-1427RM) and normal IgG antibodies at a 1:50 dilution for 2 h. After antibody binding, the cells were centrifuged and washed. A mouse anti-rabbit IgG secondary antibody was applied at a 1:100 dilution for 1 h. Following this, cells were incubated with 0.04 μM pA-Tn5 Transposon for 1 h, and DNA fragments were subsequently recovered. Library construction was completed using the Vazyme TruePrep Index Kit V2 for Illumina (TD202) with 15 PCR cycles. The PCR library was purified using Vazyme VAHTS DNA Clean Beads (N411-01-AA) and sequenced on an Illumina NovaSeq 6000 platform by Novogene Bioinformatics Technology using a PE150 sequencing strategy, according to the required library concentration and data output.

### ChIP-seq assay

For H3K18ac ChIP-seq, chromatin immunoprecipitation was performed using an anti-H3K18ac antibody (Abcam, ab1191) in HCT116 cells cultured under LG or HG conditions with PBS or Met treatment. Libraries were prepared from immunoprecipitated DNA and then processed for sequencing. ChIP-seq experiments and primary bioinformatic processing were supported by Shanghai Zhongke New Life Bioinformatics Technology Co., Ltd. Libraries were prepared, size-selected, and PCR-amplified. Library size distribution was assessed using an Agilent 5400 system (Agilent, USA) and quantified by qPCR (effective concentration: 1.5 nM). After quality control, libraries were pooled and sequenced on an Illumina platform to generate 150-bp paired-end reads (PE150). Adapter sequences and low-quality reads were removed using fastp (v0.23.1), and clean reads were aligned to the reference genome using BWA (v0.7.12). Peaks were called using MACS2 (v2.2.7.1) with a *q*-value threshold of 0.05. For visualization, BAM and normalized bigWig files were loaded into IGV (Integrative Genomics Viewer) to inspect enrichment profiles, and representative tracks, including the c-JUN promoter/TSS region, were displayed using identical genomic coordinates and visualization settings across conditions.

### LC-MS-based metabolomics

Untargeted metabolomics was performed on CD133⁺ SW620 cells from 4 experimental groups (*n* = 6 biologically independent samples per group; 24 samples in total). Samples were extracted with methanol/acetonitrile/water (2:2:1, v/v/v) containing internal standards, homogenized, incubated at −20 °C for 2 h, and centrifuged at 25,000 × *g* for 15 min at 4 °C. Supernatants were collected for analysis, and pooled QC samples were prepared by mixing aliquots from all samples. Metabolite profiling was performed using a Waters UPLC I-Class Plus system coupled to a Q Exactive high-resolution mass spectrometer (Thermo Fisher Scientific, USA). Chromatographic separation was carried out on a Waters ACQUITY UPLC BEH C18 column (1.7 μm, 2.1 × 100 mm). Data were acquired in both positive- and negative-ion modes. Raw data were processed using Compound Discoverer 3.2 and MetaX, including peak extraction, metabolite annotation, probabilistic quotient normalization, QC-based robust LOESS signal correction, and removal of metabolites with a coefficient of variation >30% in QC samples. Multivariate analyses including PCA and PLS-DA were performed after log₂ transformation and Pareto scaling. Differential metabolites were identified based on VIP > 1, together with fold-change and univariate statistical criteria. Metabolite annotation and pathway analysis were performed using HMDB and KEGG.

### RNA-seq

Total RNA was extracted from metformin-treated SW620 CD133^+^ cells using TRIzol reagent (Invitrogen), with its integrity and concentration assessed via an Agilent 2100 Bioanalyzer (Agilent Technologies, Palo Alto, CA, USA). Only RNA samples with an RIN greater than 7 were processed for library construction following the NEBNext® Ultra™ RNA Library Prep Kit for Illumina® protocol. These libraries were sequenced on an Illumina HiSeq system using a 2 × 150 bp paired-end configuration. Image processing and base calling were executed using HiSeq Control Software (HCS) + OLB + GAPipeline-1.6 (Illumina). Adapters and low-quality sequences were removed to yield clean reads, which were then aligned to the human genome (hg38) using the STAR aligner (v.2.7.0e). Differentially expressed genes were identified using DESeq2, with significance set at an adjusted *P*-value of less than 0.05.

### Quantification and statistical analysis

Statistical analyses were performed using GraphPad Prism 10. Depending on the experimental design, statistical significance was determined using two-sided Student’s *t*-test, two-sided two-way ANOVA followed by Tukey’s multiple-comparison test, or the log-rank (Mantel–Cox) test, as appropriate. The statistical tests used for each experiment are indicated in the corresponding figure legends. The significance of the results was denoted in the figures: *P < 0.05; **P < 0.01; ***P < 0.001, indicating levels of statistical significance.

### Statistics and reproducibility

Quantitative animal experiments were performed using biologically independent mice, as indicated in the corresponding figure legends. For immunohistochemical and immunofluorescence analyses of mouse tumors, representative images are shown, and quantification was performed using samples from *n* = 5 biologically independent mice per group unless otherwise stated.

For patient-derived organoid experiments, immunofluorescence analyses were performed in three independent experiments. Representative images are shown, and quantification was performed from organoids imaged across these independent experiments, as indicated in the corresponding figure legends. Quantitative cell-based assays, including apoptosis assays, CCK-8 assays, lipid droplet staining, and other quantified analyses, were performed as indicated in the corresponding figure legends. For western blot experiments shown without densitometric quantification, representative blots are presented. For Western blot experiments with densitometric analysis, quantification was performed from three independent experiments using Image Lab software, unless otherwise stated. Representative western blots and microscopy images are shown unless otherwise stated.

No statistical method was used to predetermine sample size. Sample sizes were chosen based on commonly used sample sizes in the field and our previous experience with these experimental models. No data were excluded from the analyses. Formal randomization was not performed. For animal experiments, mice were allocated to treatment groups when tumors reached comparable baseline volumes. Investigators were not blinded to group allocation during experiments or outcome assessment.

### Reporting summary

Further information on research design is available in the Nature Portfolio Reporting Summary linked to this article.

## Supplementary information

Supplementary Information

Reporting Summary

Transparent Peer Review file

## Source data

Source Data

## Data Availability

The sequencing datasets generated in this study, including RNA-seq, ChIP-seq and CUT&Tag data, have been deposited in the GSA-Human database under accession codes HRA018092, HRA018096 and HRA018076. The mass spectrometry-based metabolomics raw data generated in this study have been deposited in the OMIX database under accession code OMIX016483. Publicly available transcriptomic comparison data were obtained from GEPIA, which is based on TCGA and GTEx datasets. Source data are provided with this paper.
