# Supplementary material for: Glucose-induced histone lactylation confers metformin resistance in colorectal cancer stem cells
Source: Nat Commun. 2026 Sep 7;17:10594. doi: 10.1038/s41467-026-77051-9 (PMC13646297; doi:10.1038/s41467-026-77051-9)
Supplement: Supplementary file 1 [file 41467_2026_77051_MOESM1_ESM.pdf]

Supplementary Figure 1

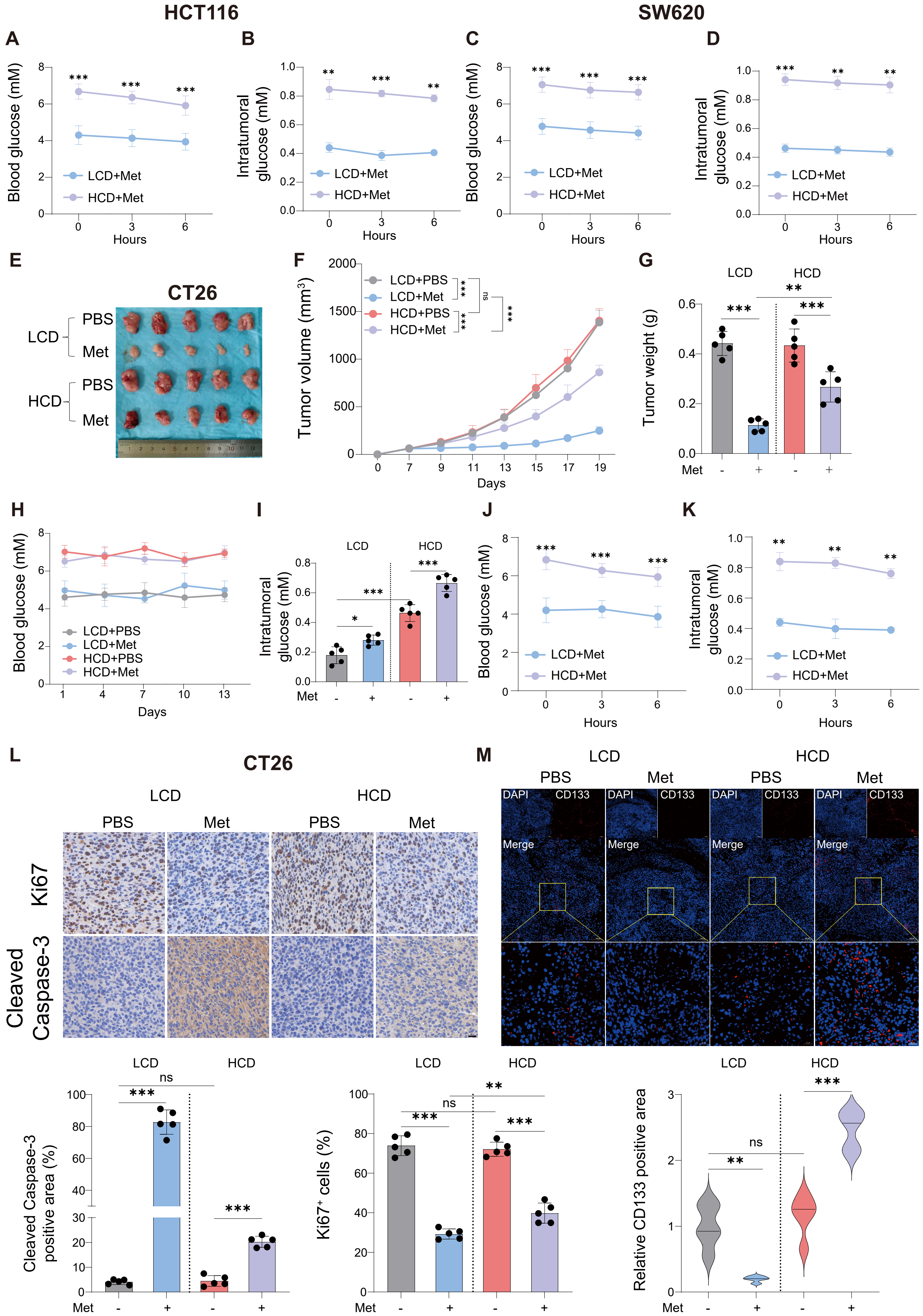

Supplementary Figure 2

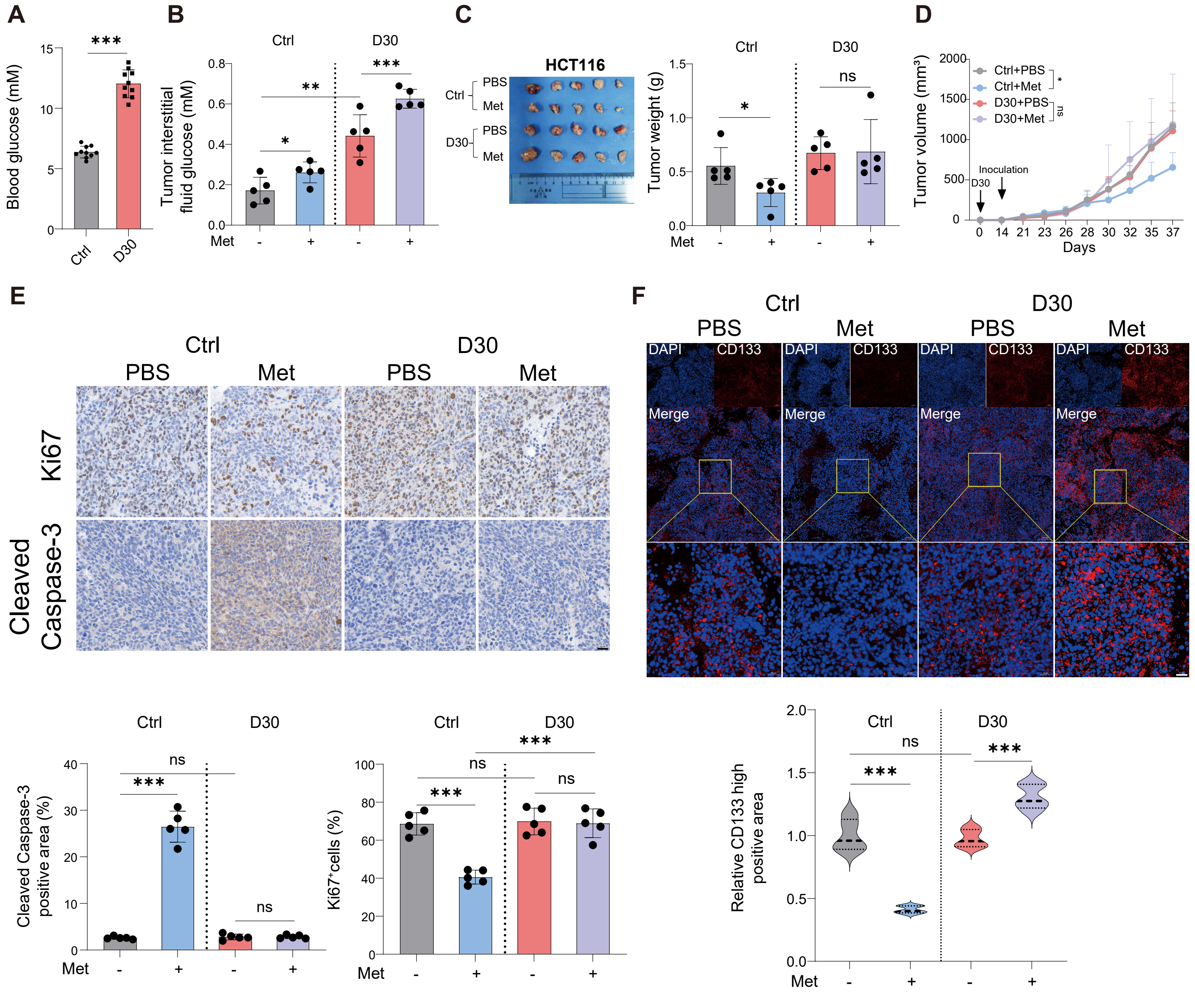

Supplementary Figure 3

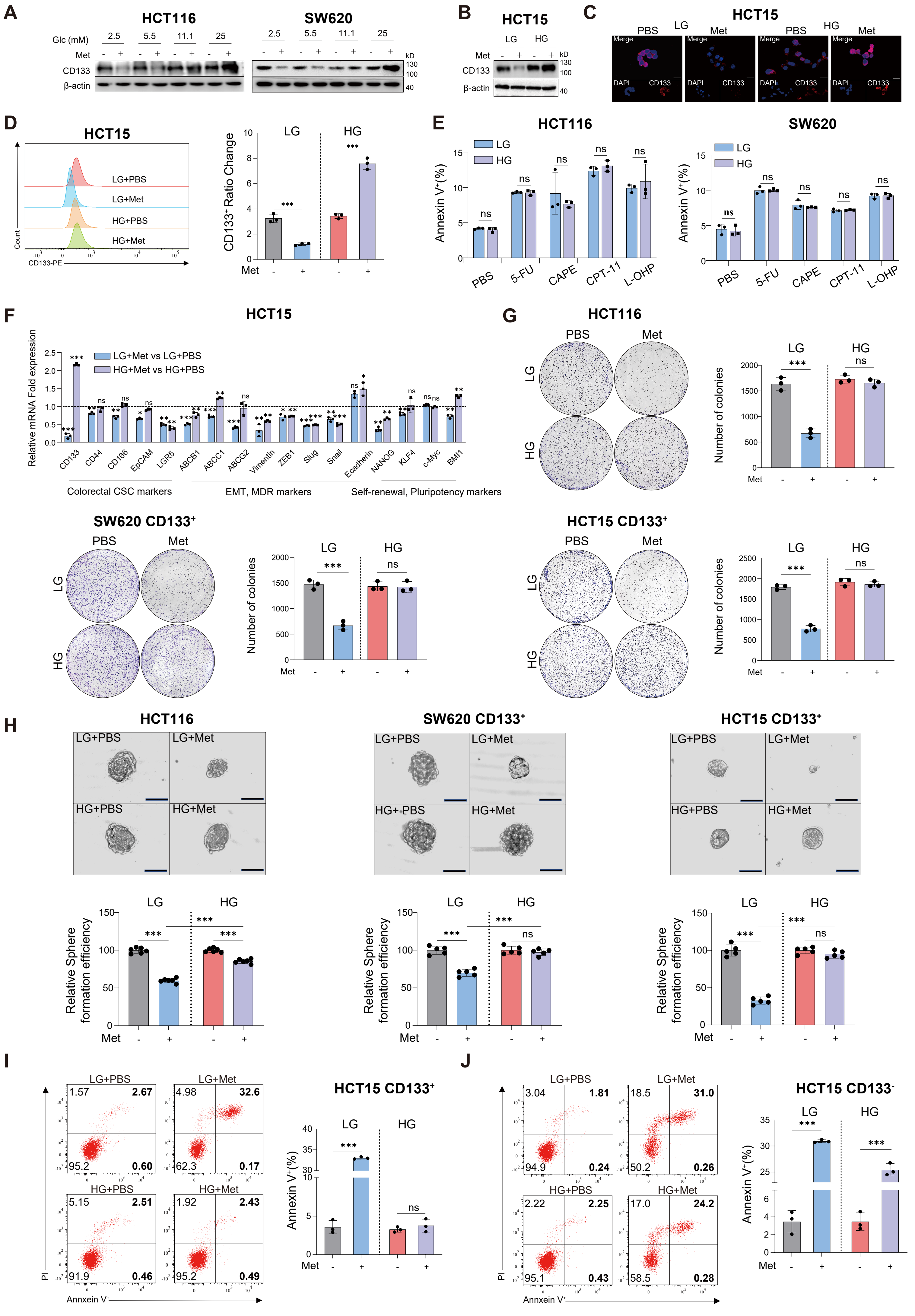

Supplementary Figure 4

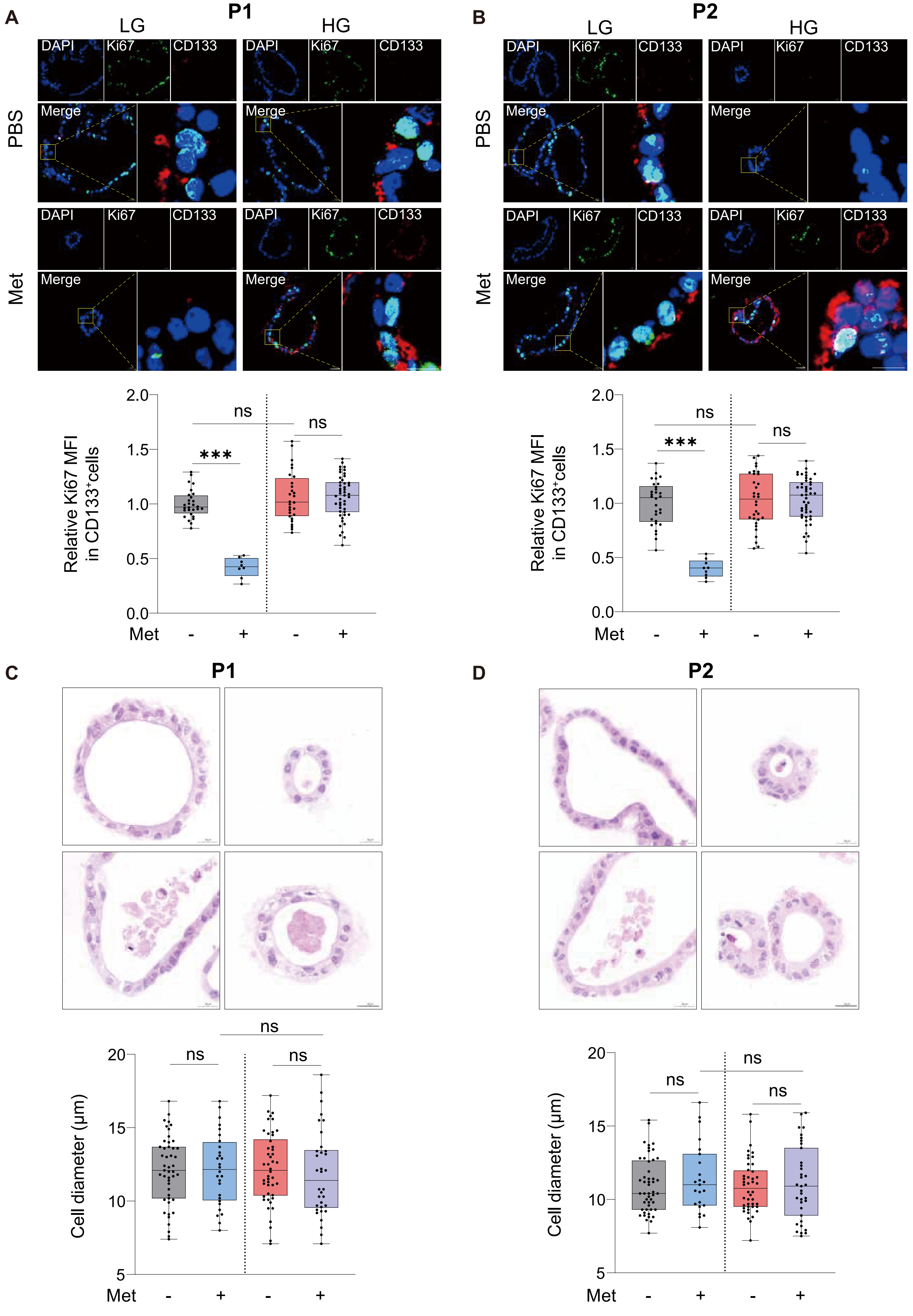

Supplementary Figure 5

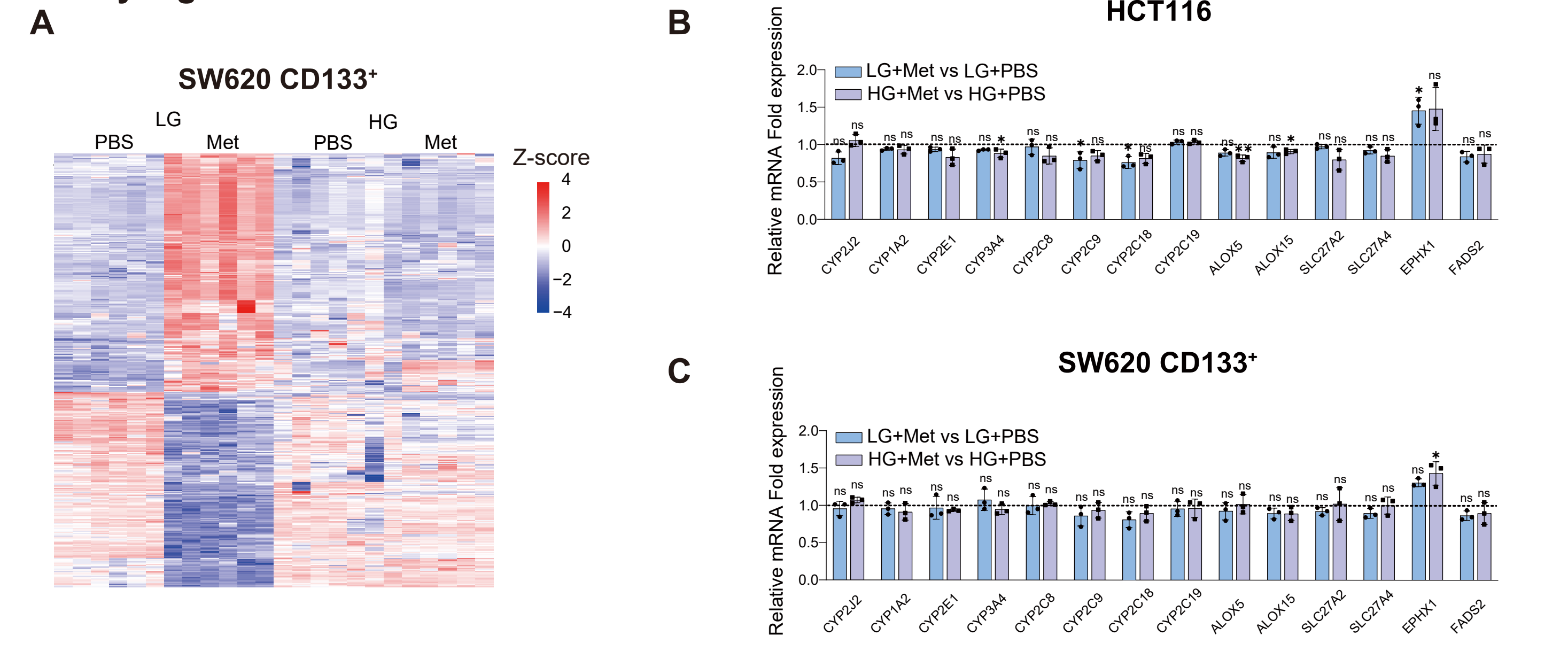

Supplementary Figure 6

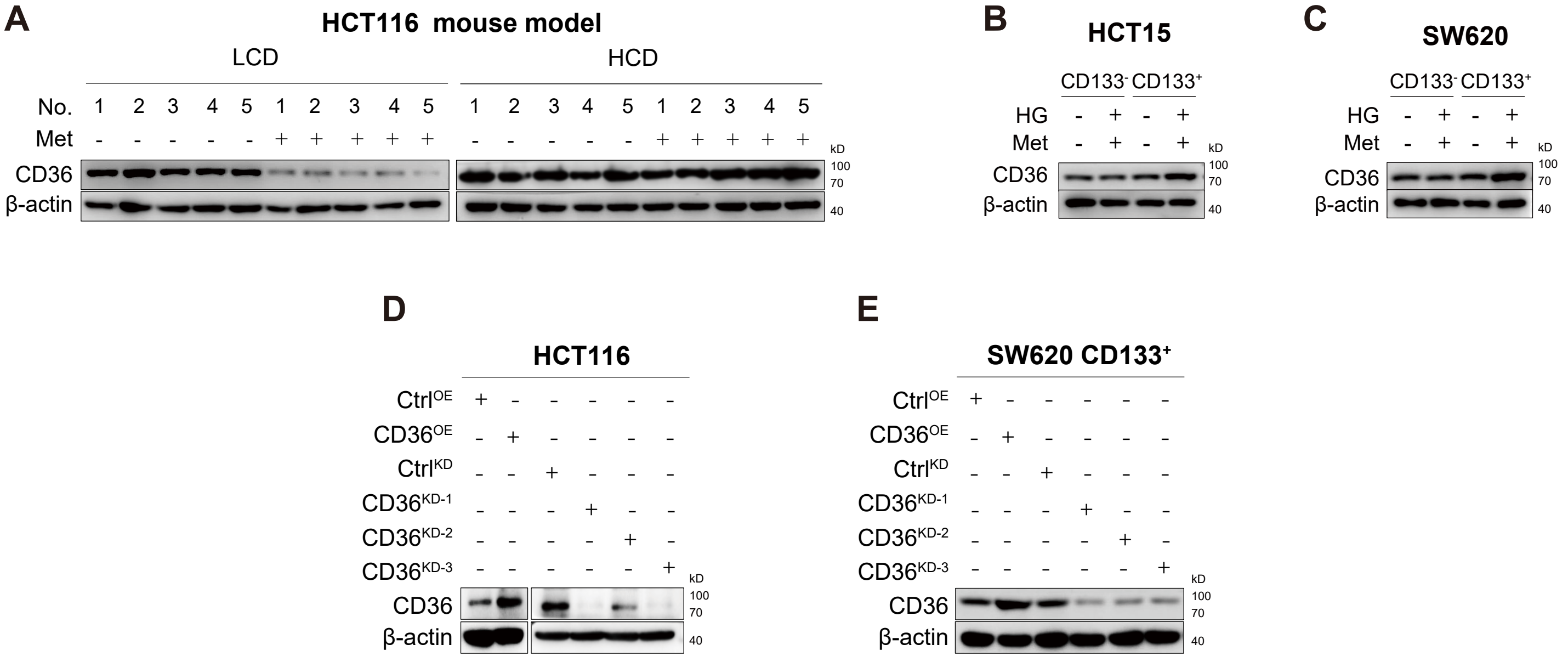

Supplementary Figure 7

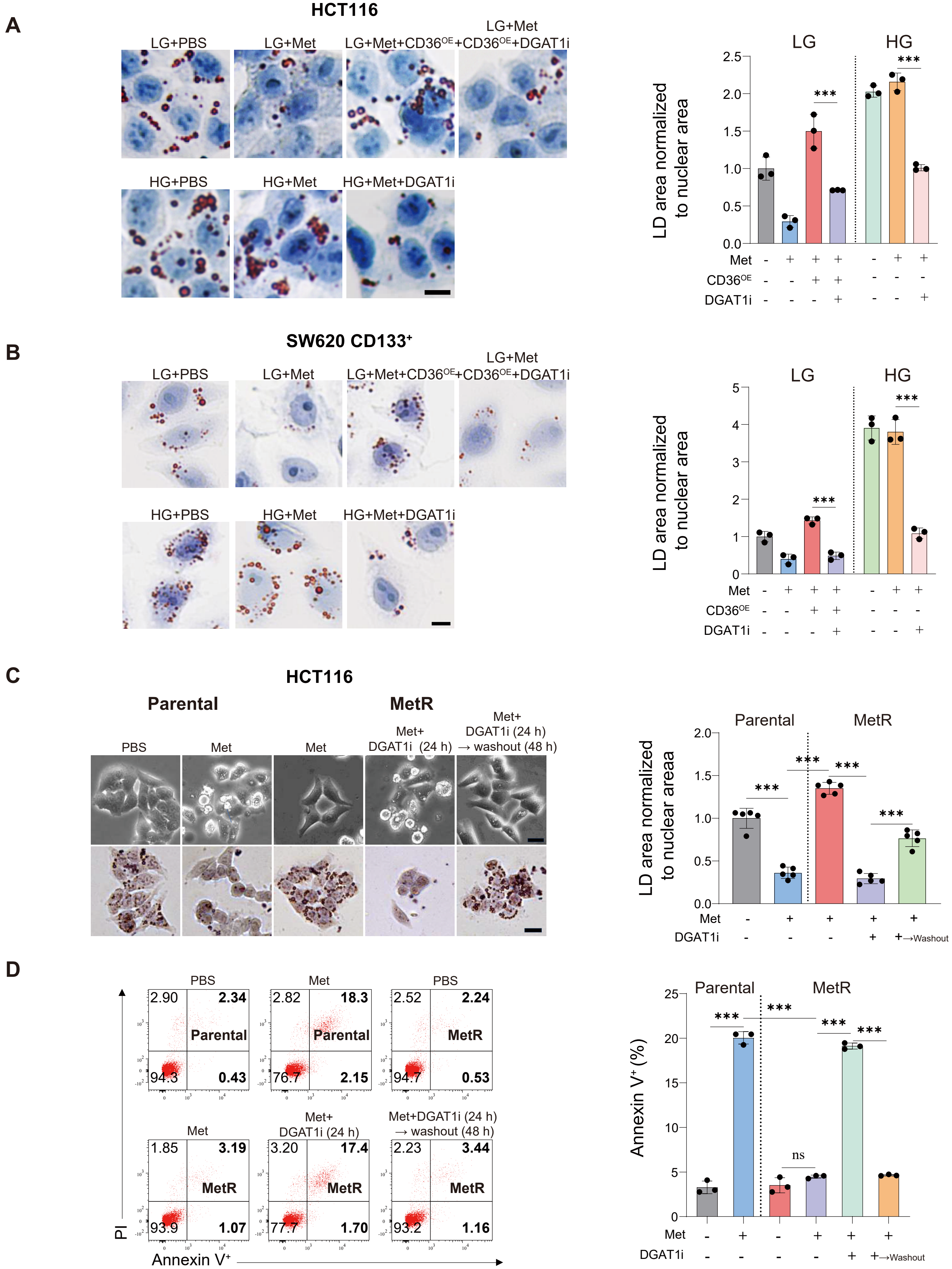

Supplementary Figure 8

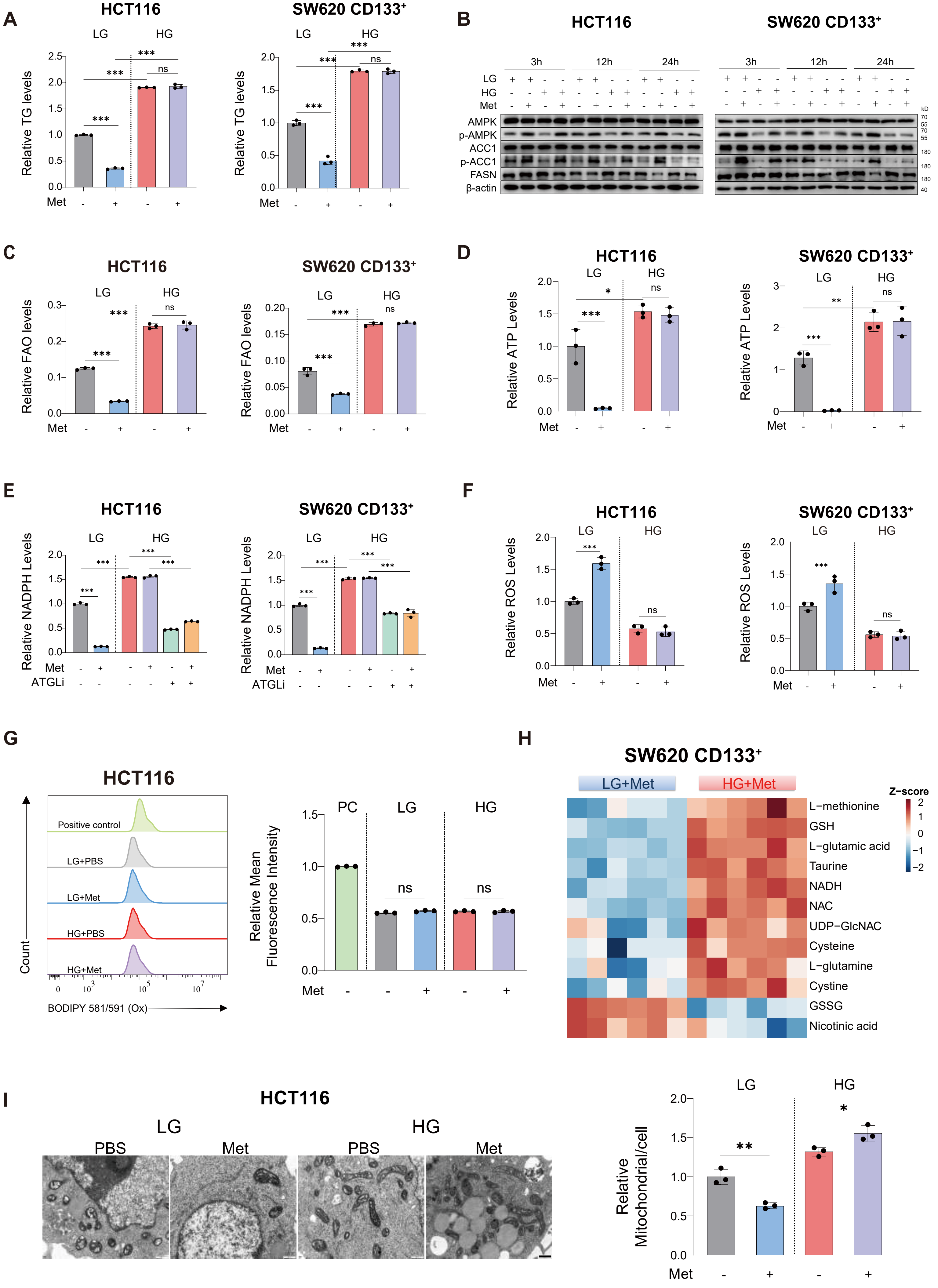

Supplementary Figure 8

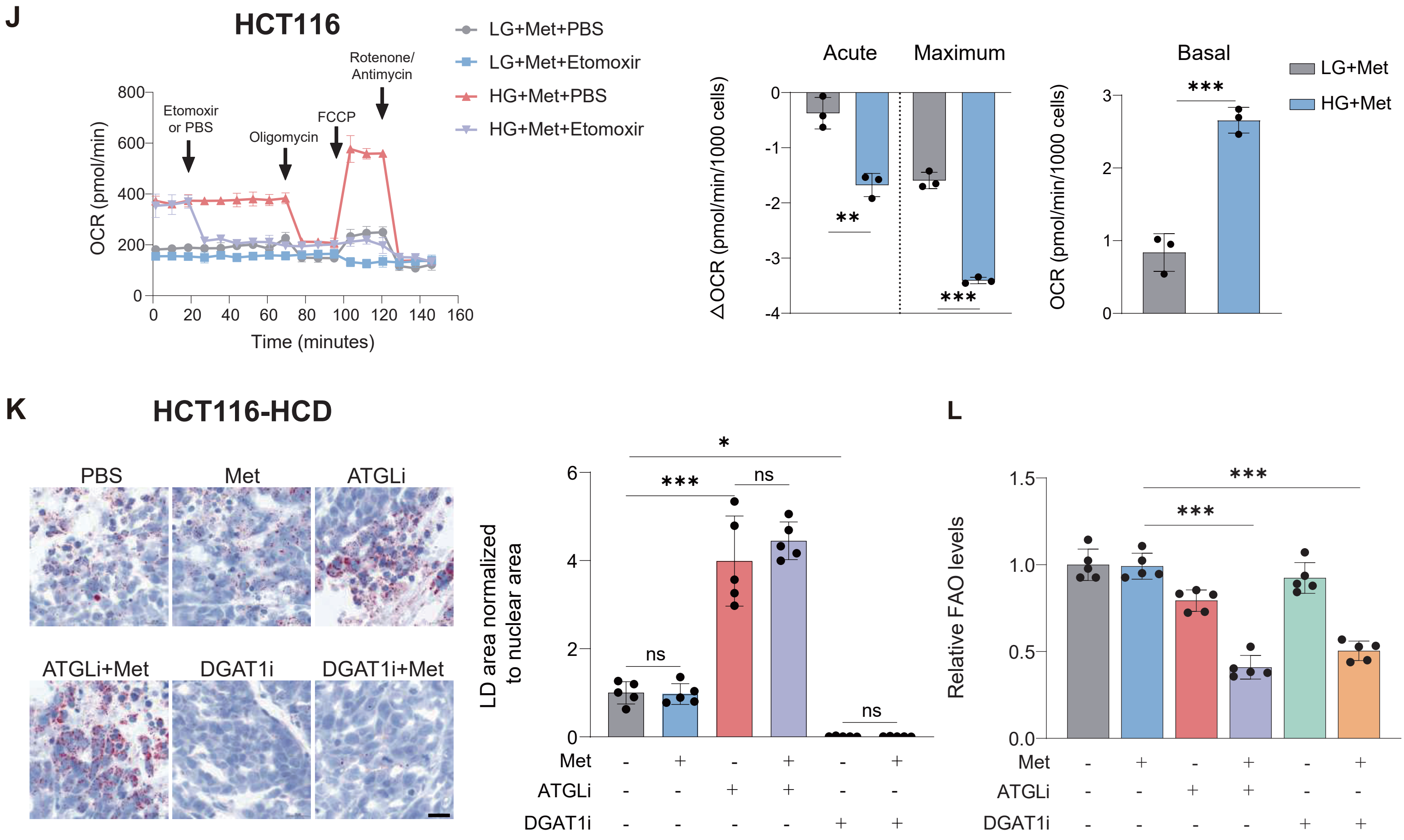

Supplementary Figure 9

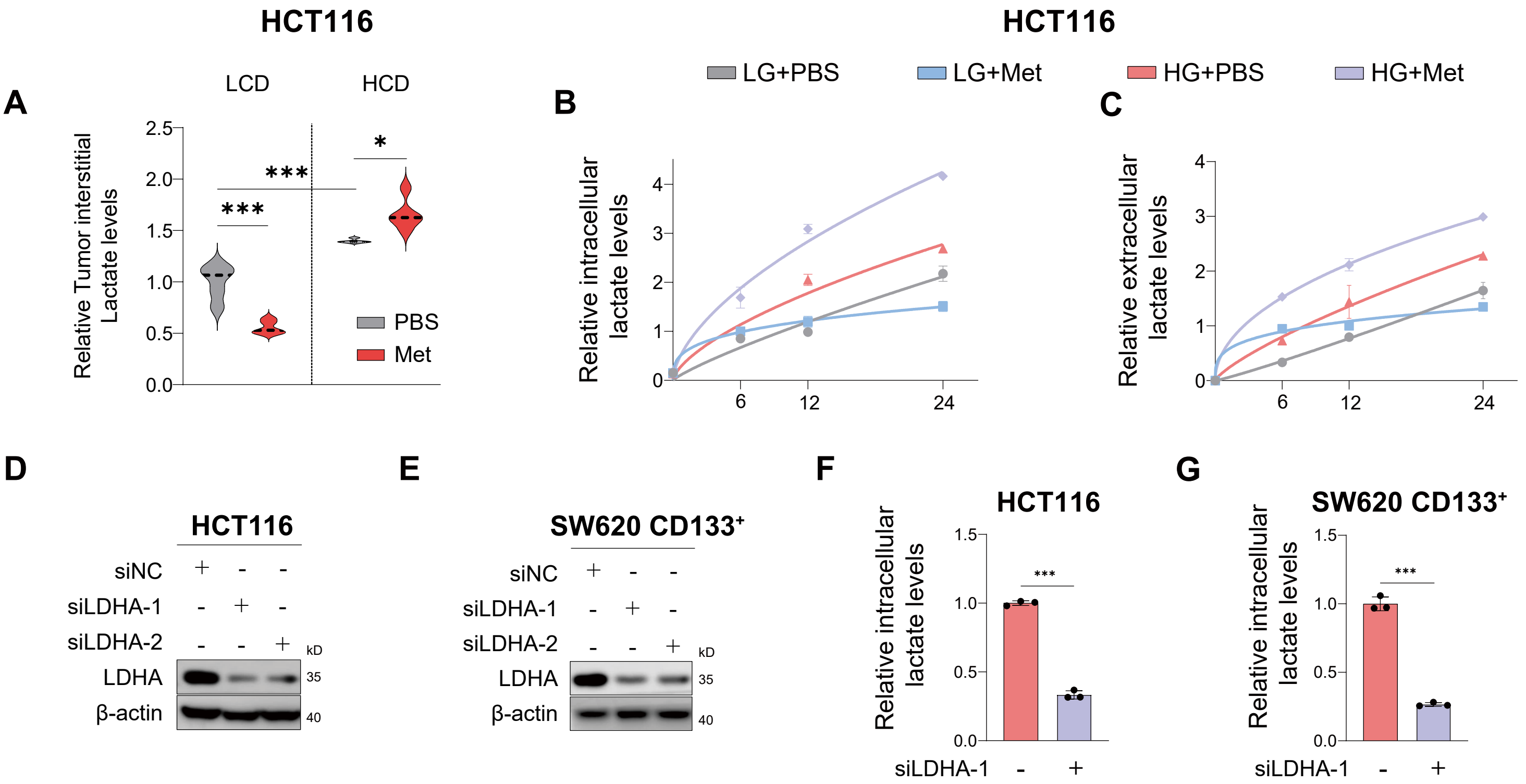

Supplementary Figure 10

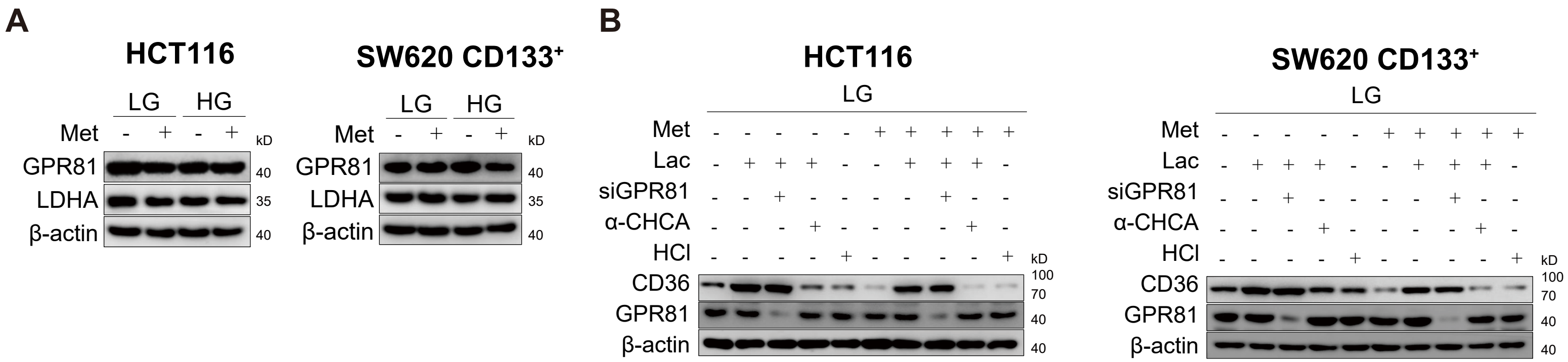

Supplementary Figure 11

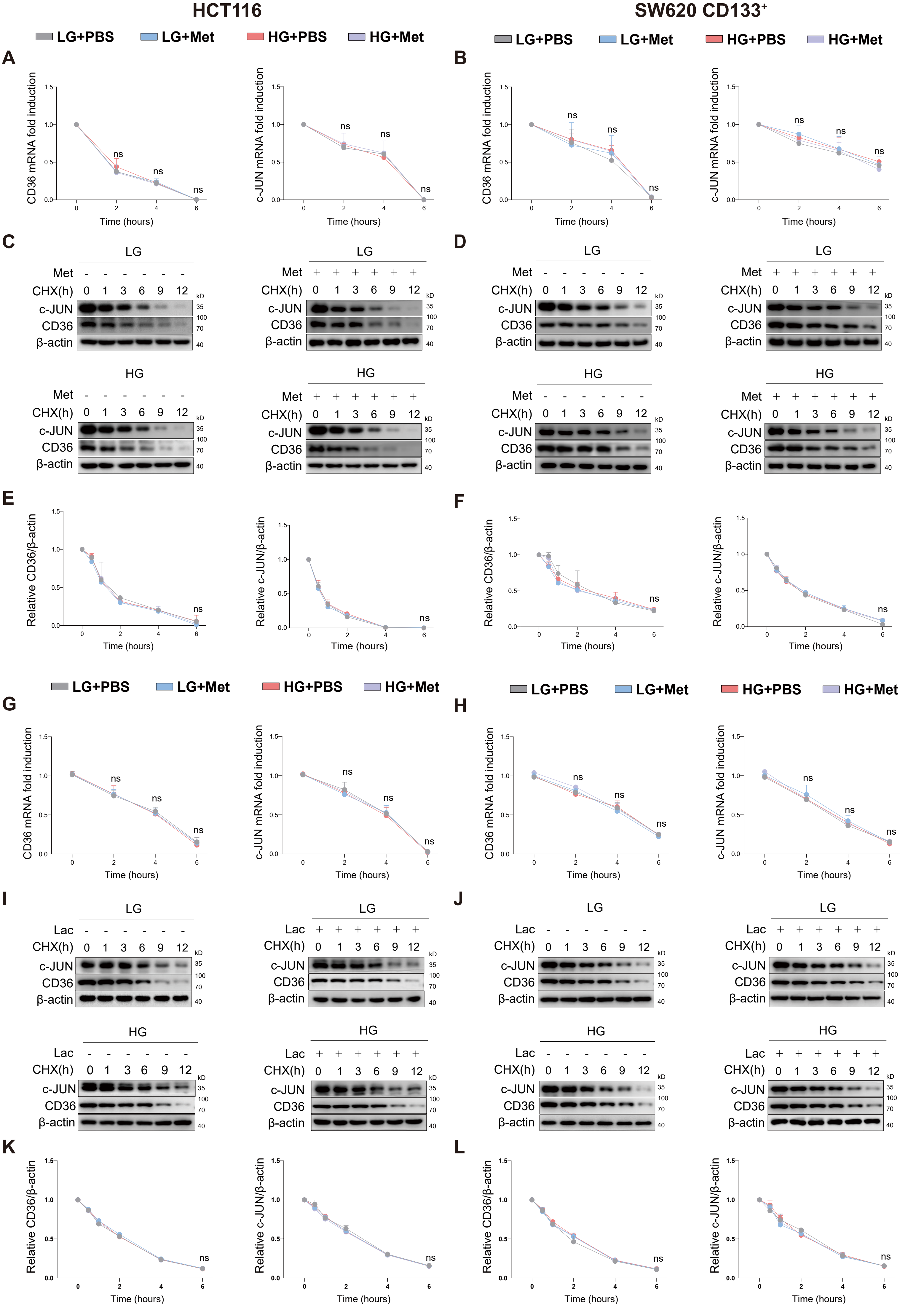

Supplementary Figure 12

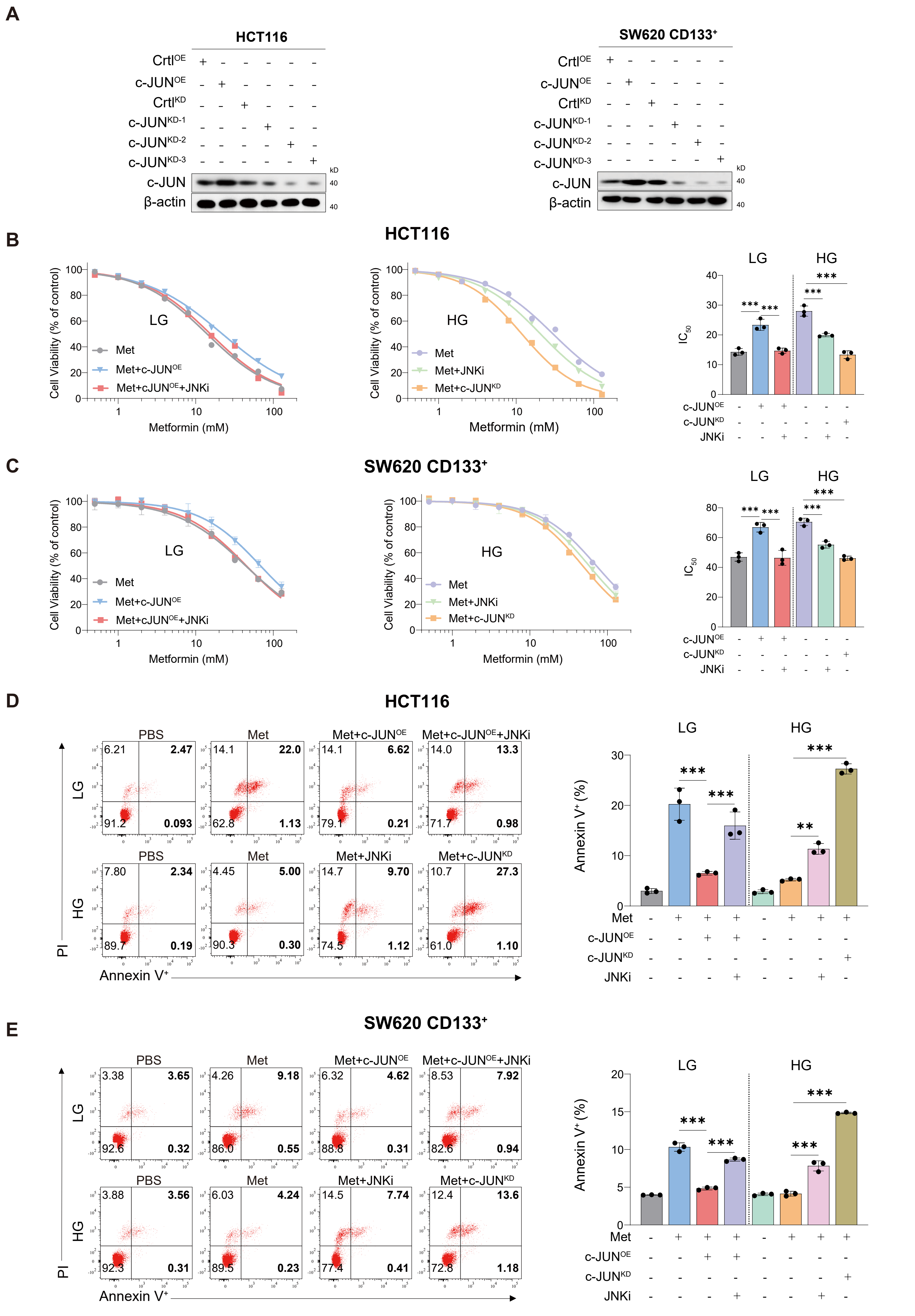

Supplementary Figure 13

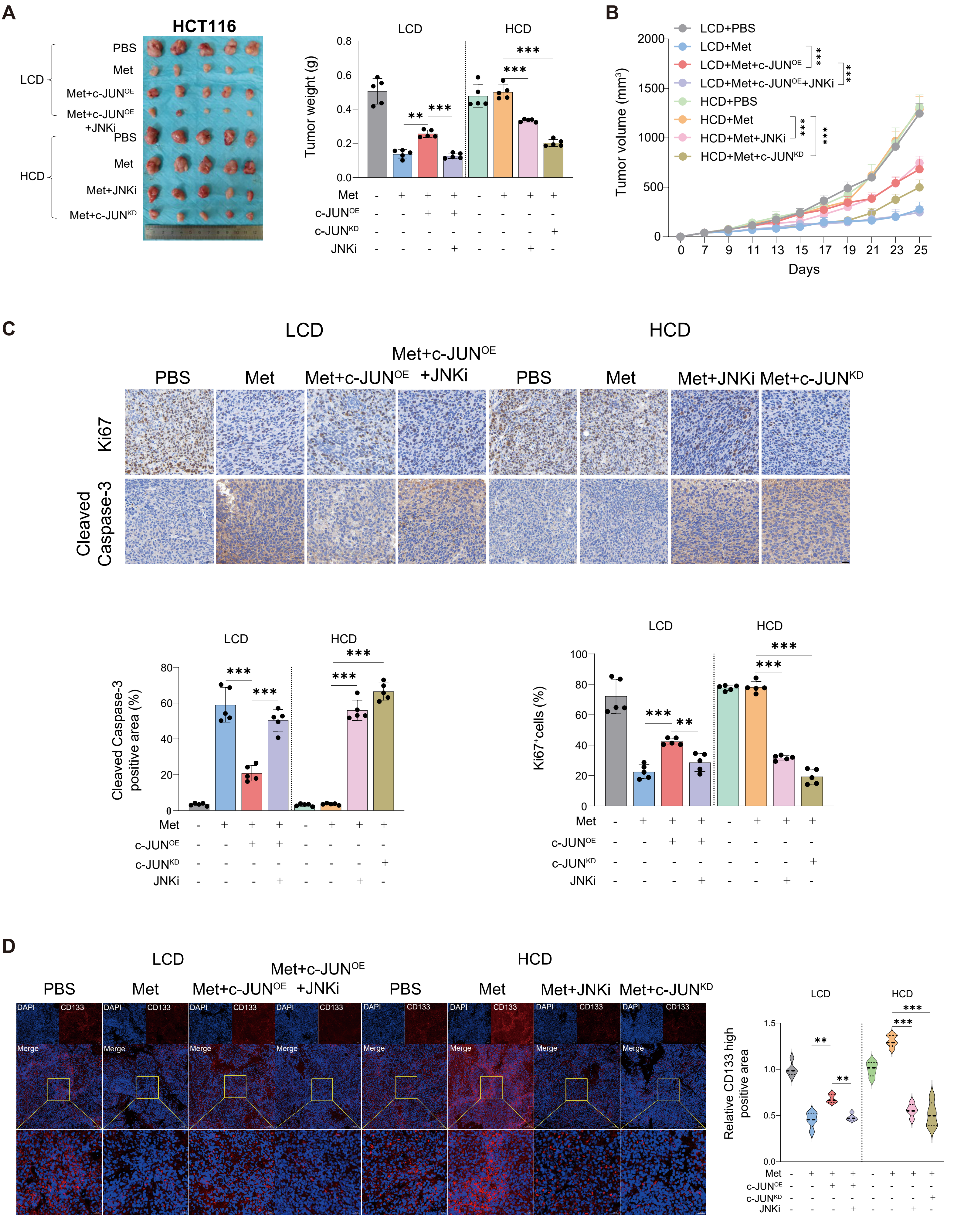

Supplementary Figure 14

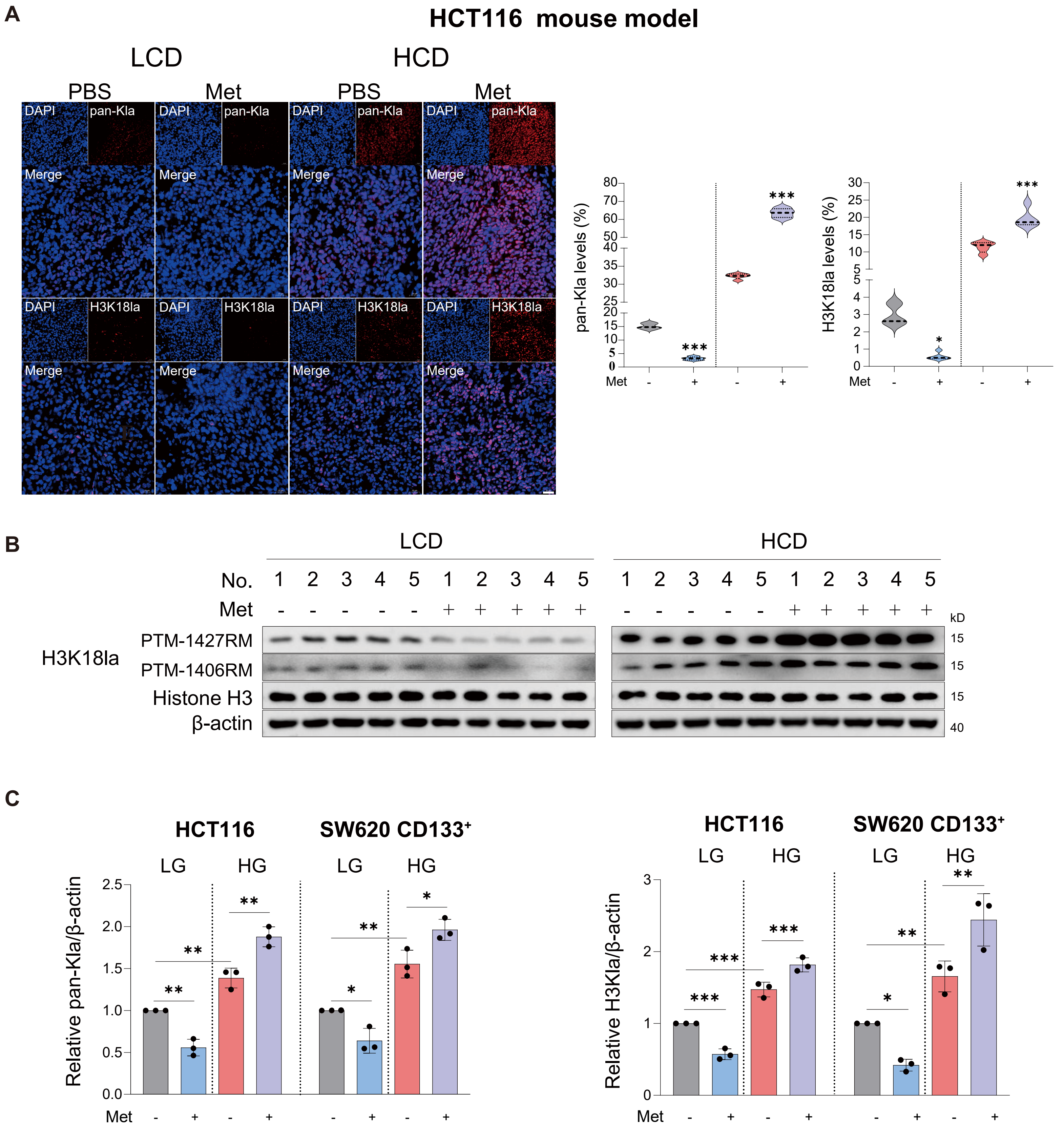

Supplementary Figure 15

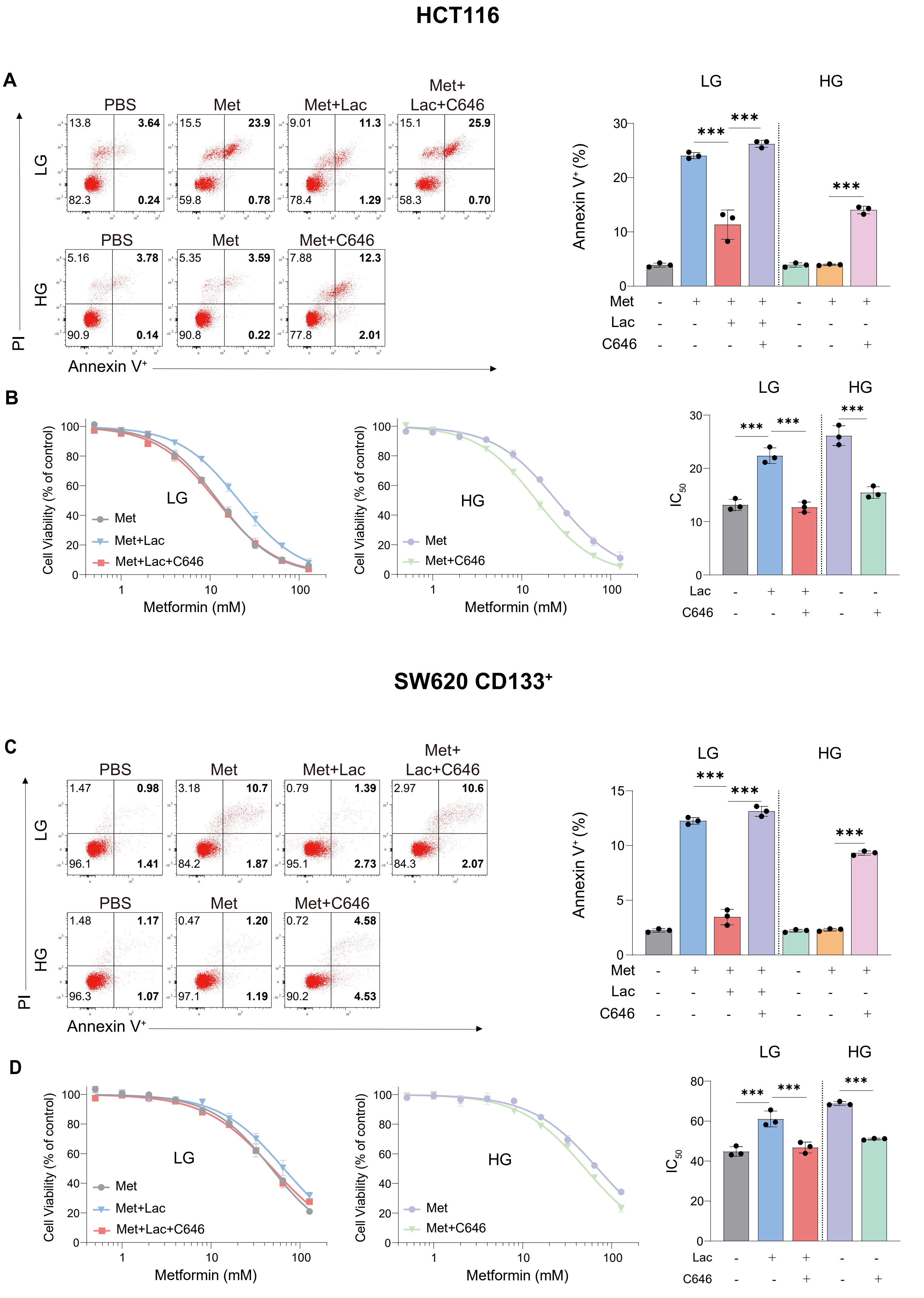

Supplementary Figure 16

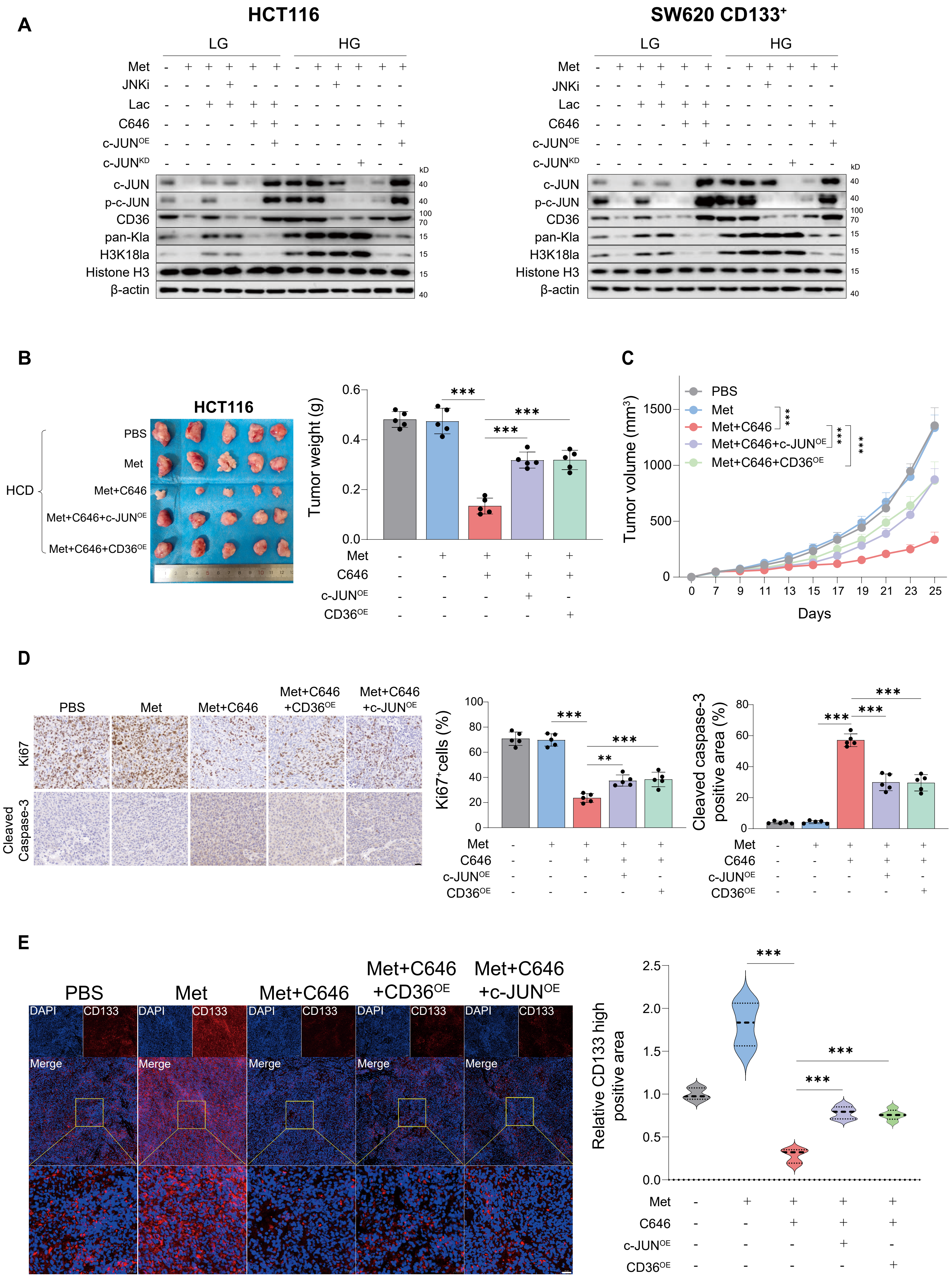

Supplementary Figure 17

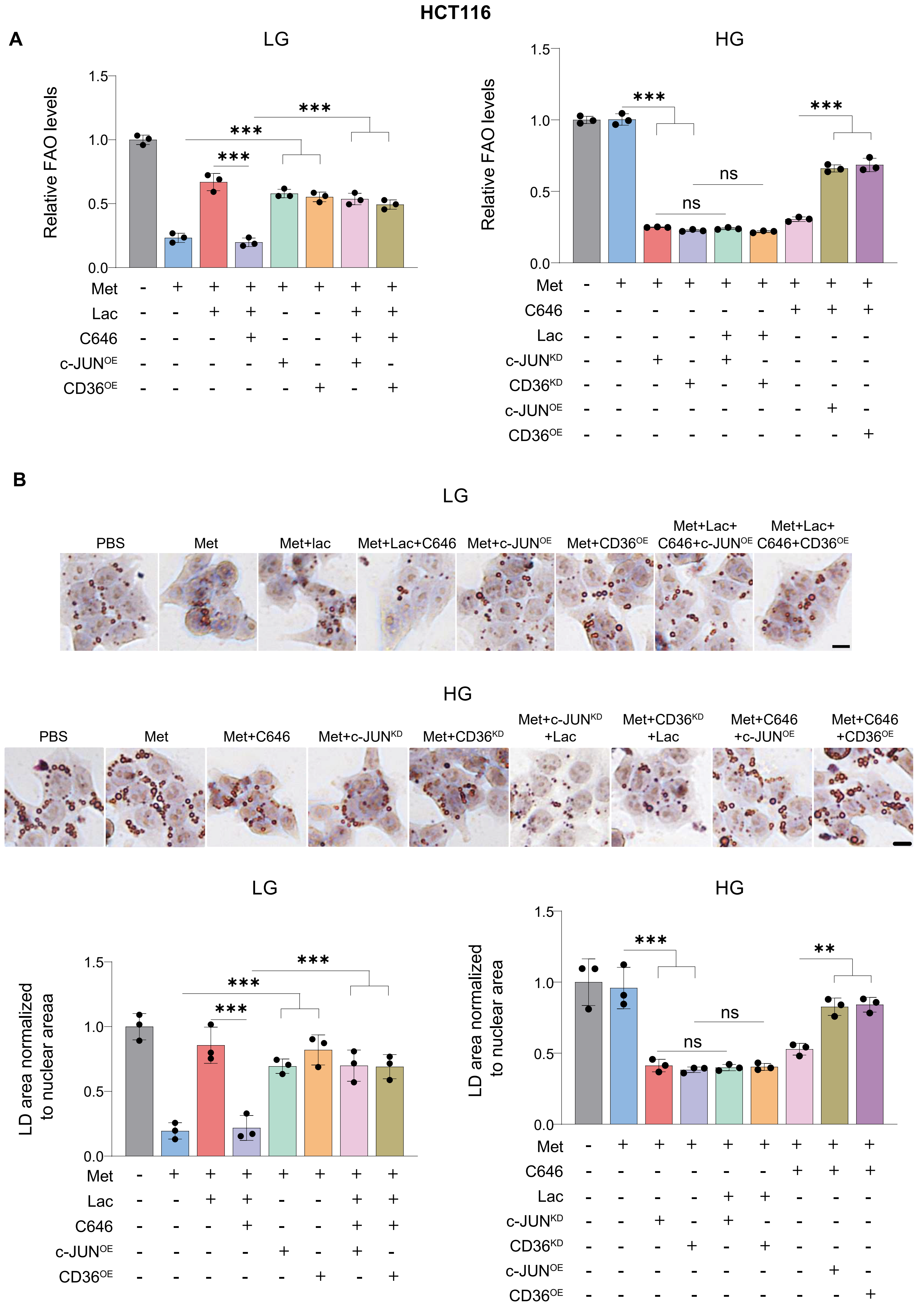

Supplementary Figure 18

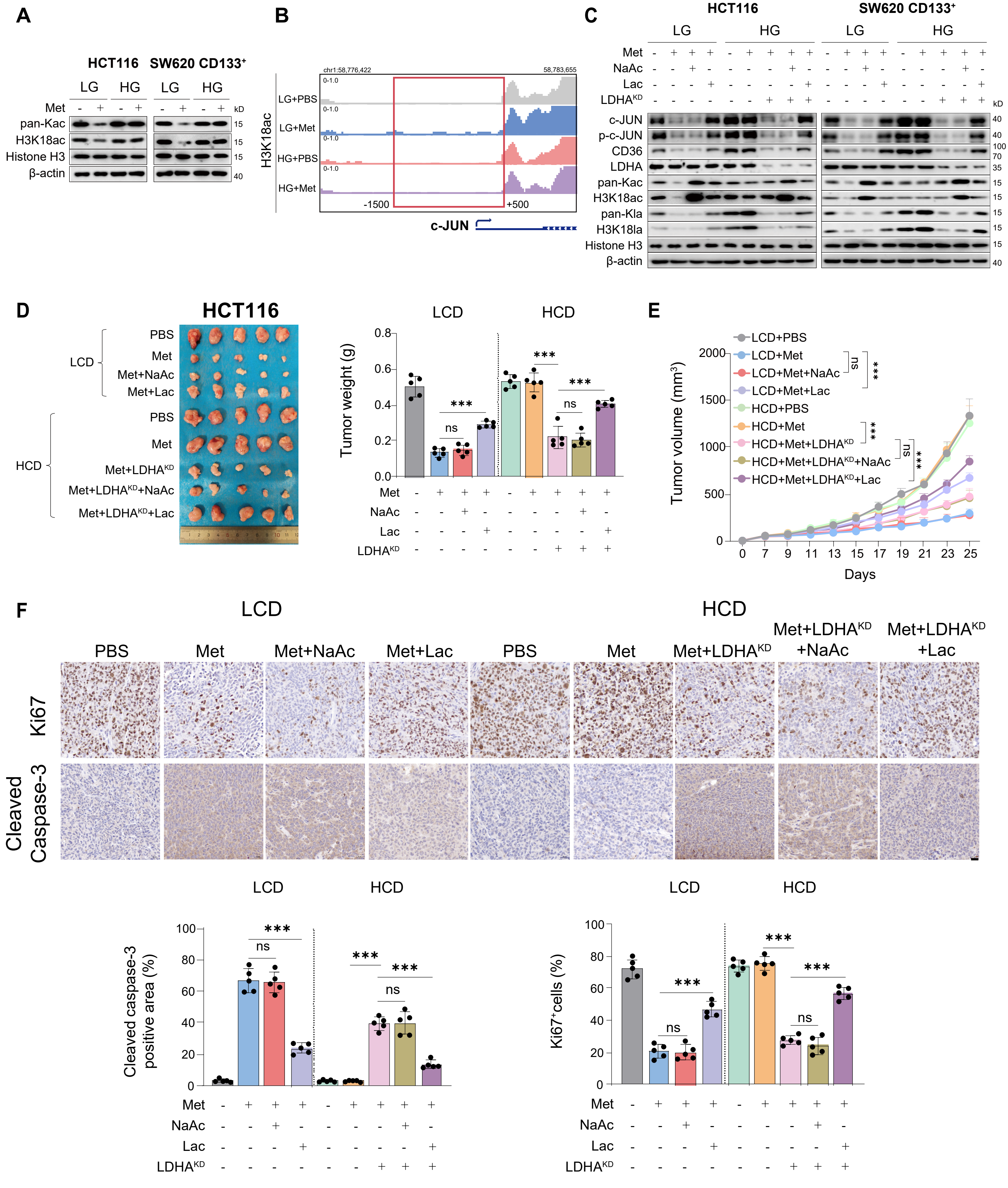

Supplementary Figure 18

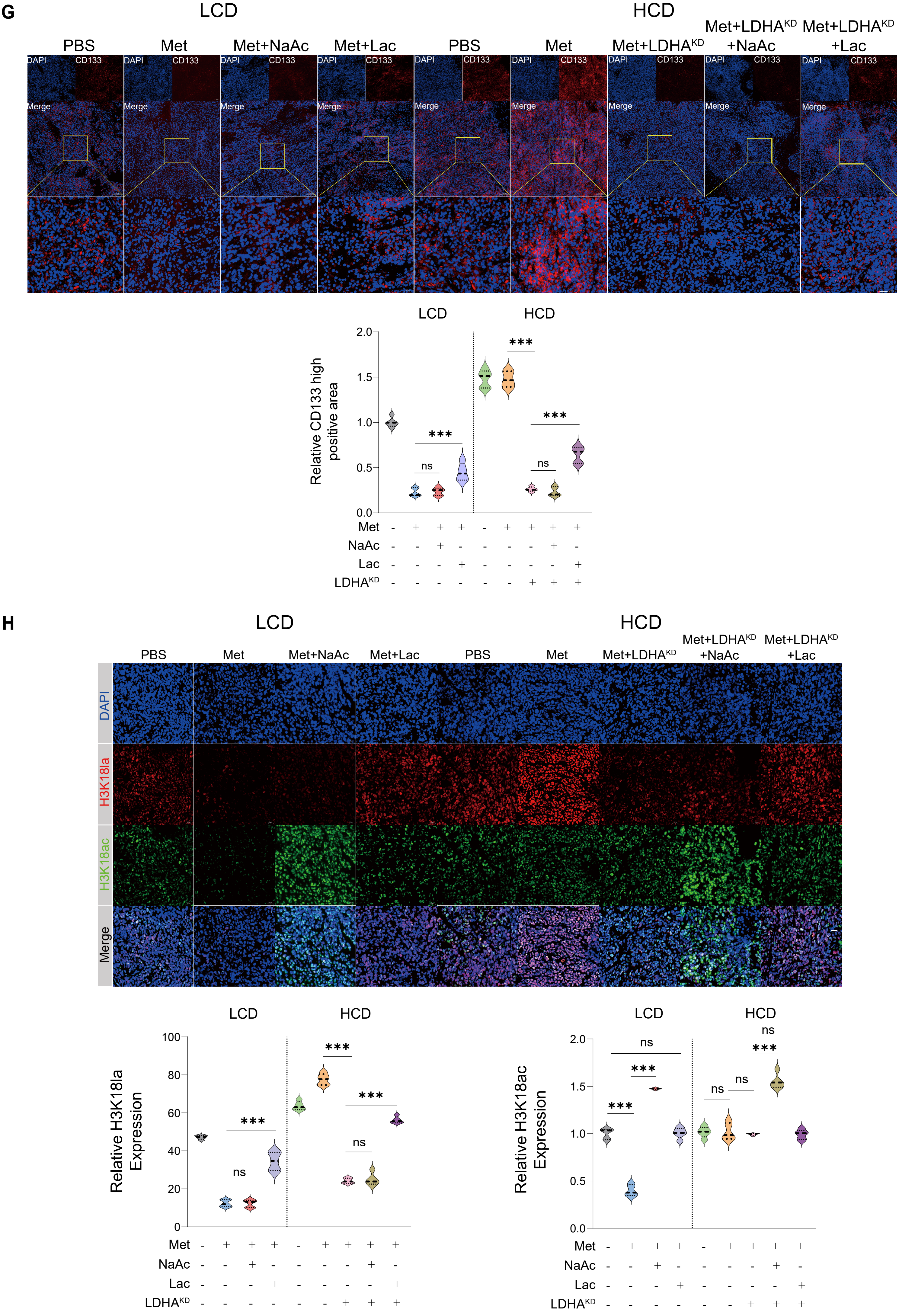

Supplementary Figure 19

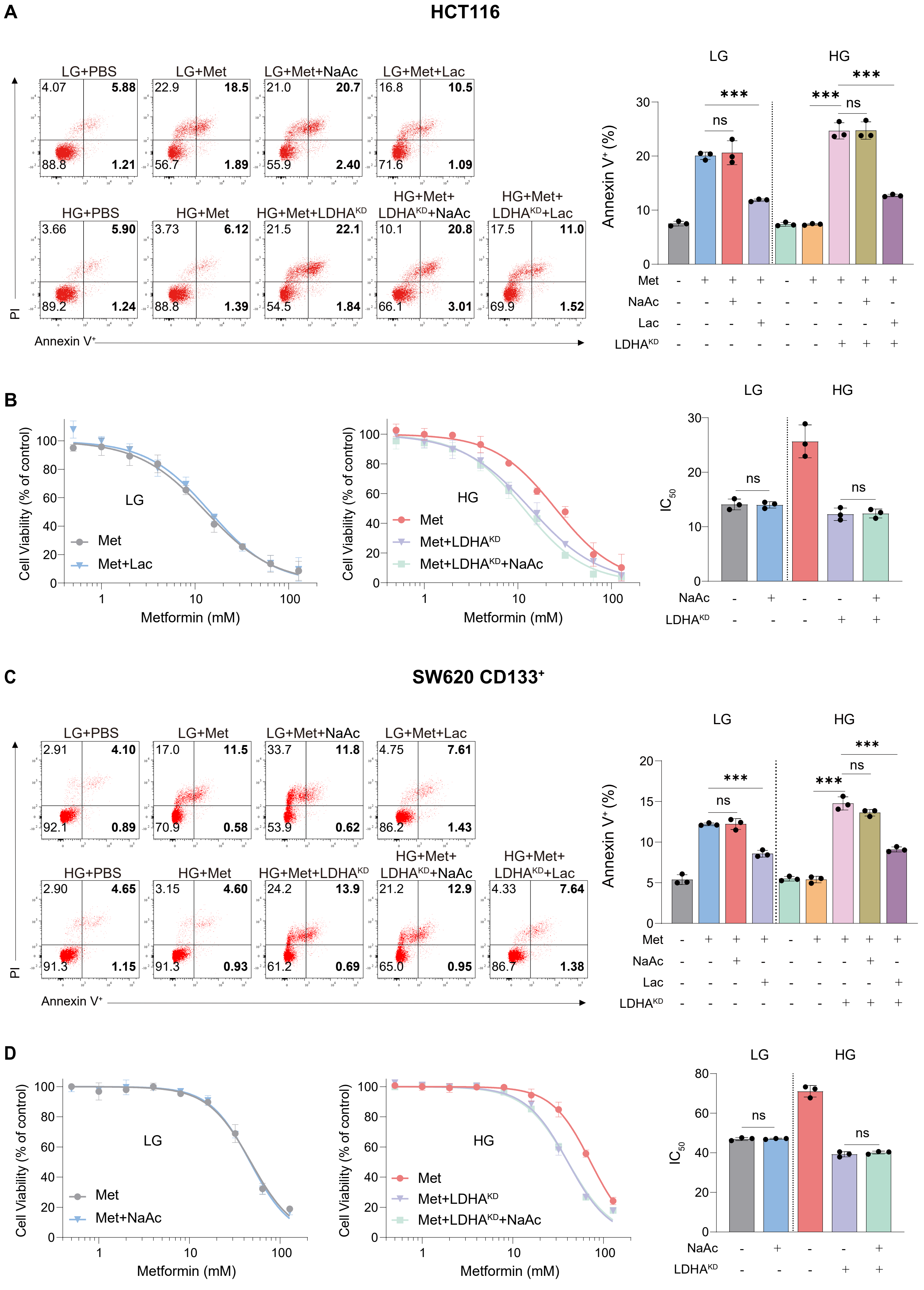

Supplementary Figure 20

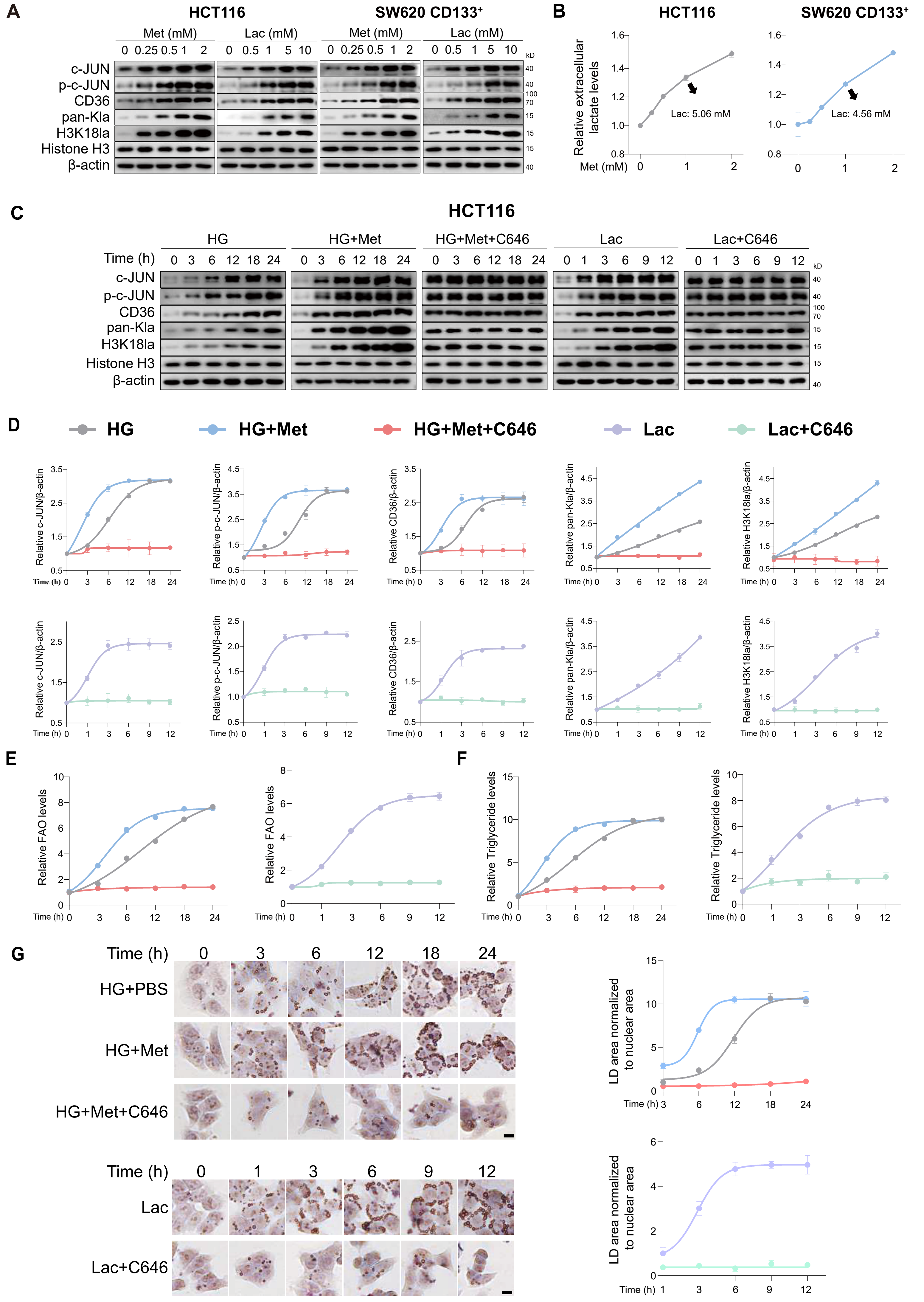

Supplementary Figure 21

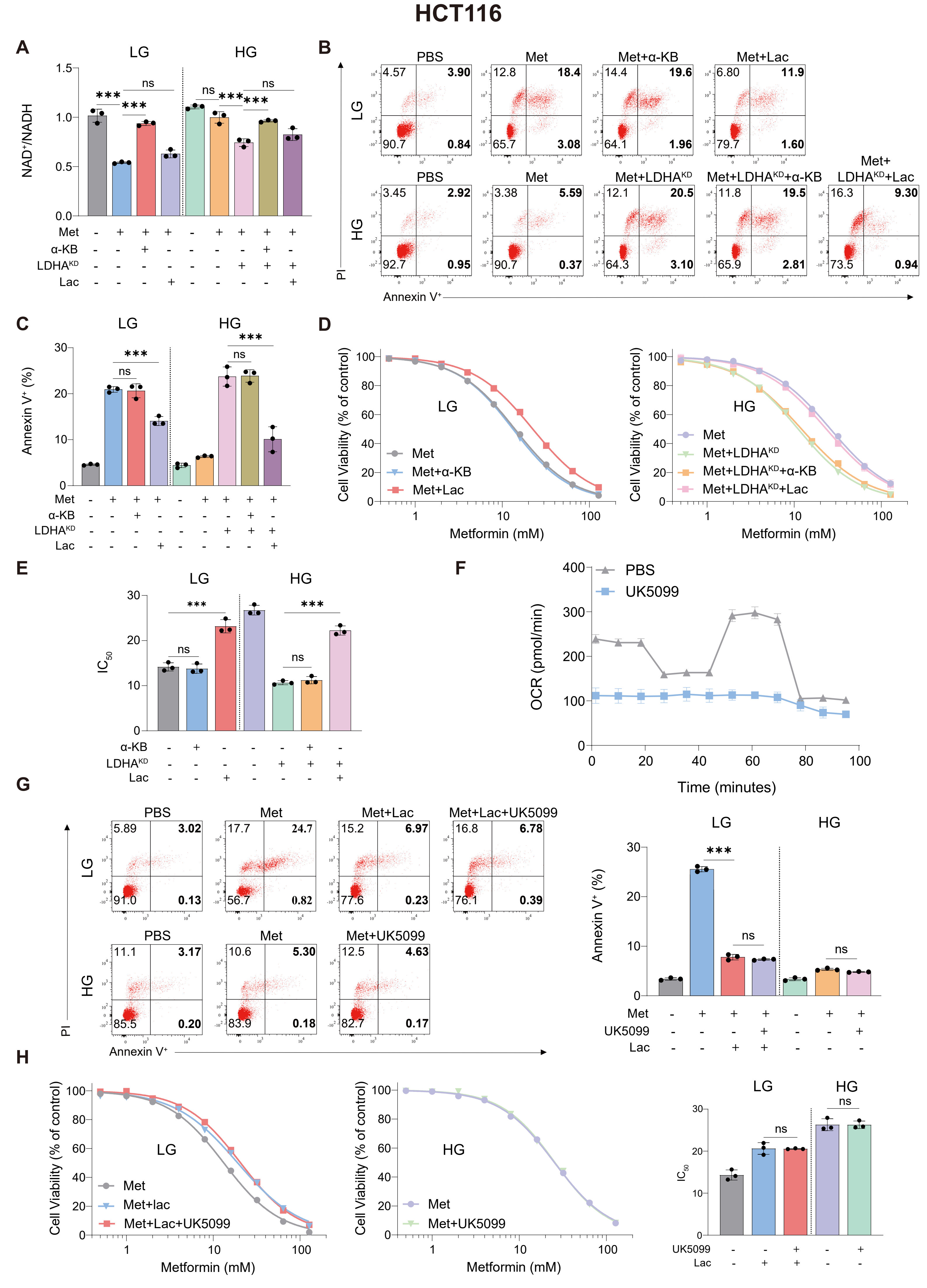

Supplementary Figure 22

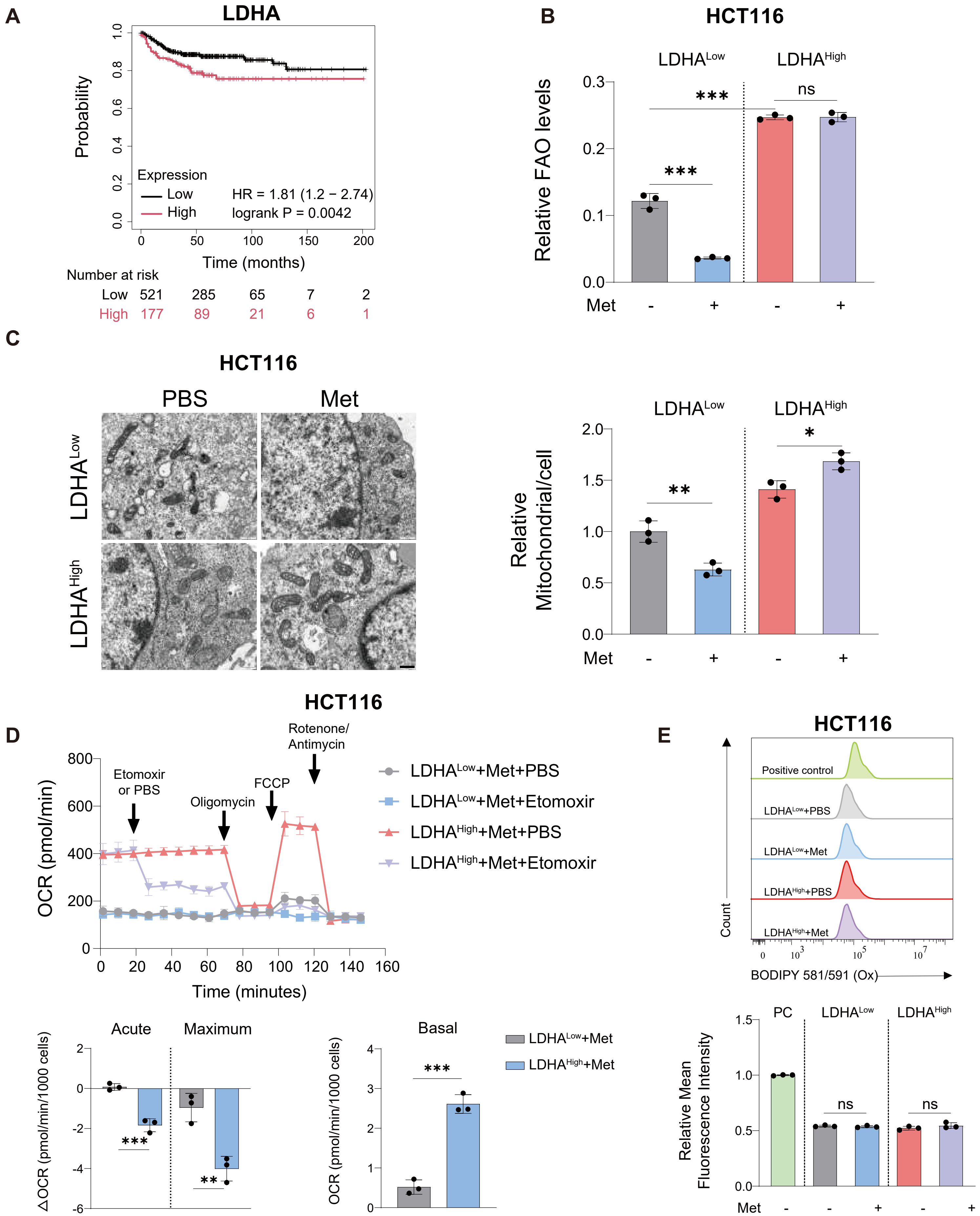

Supplementary Figure 23

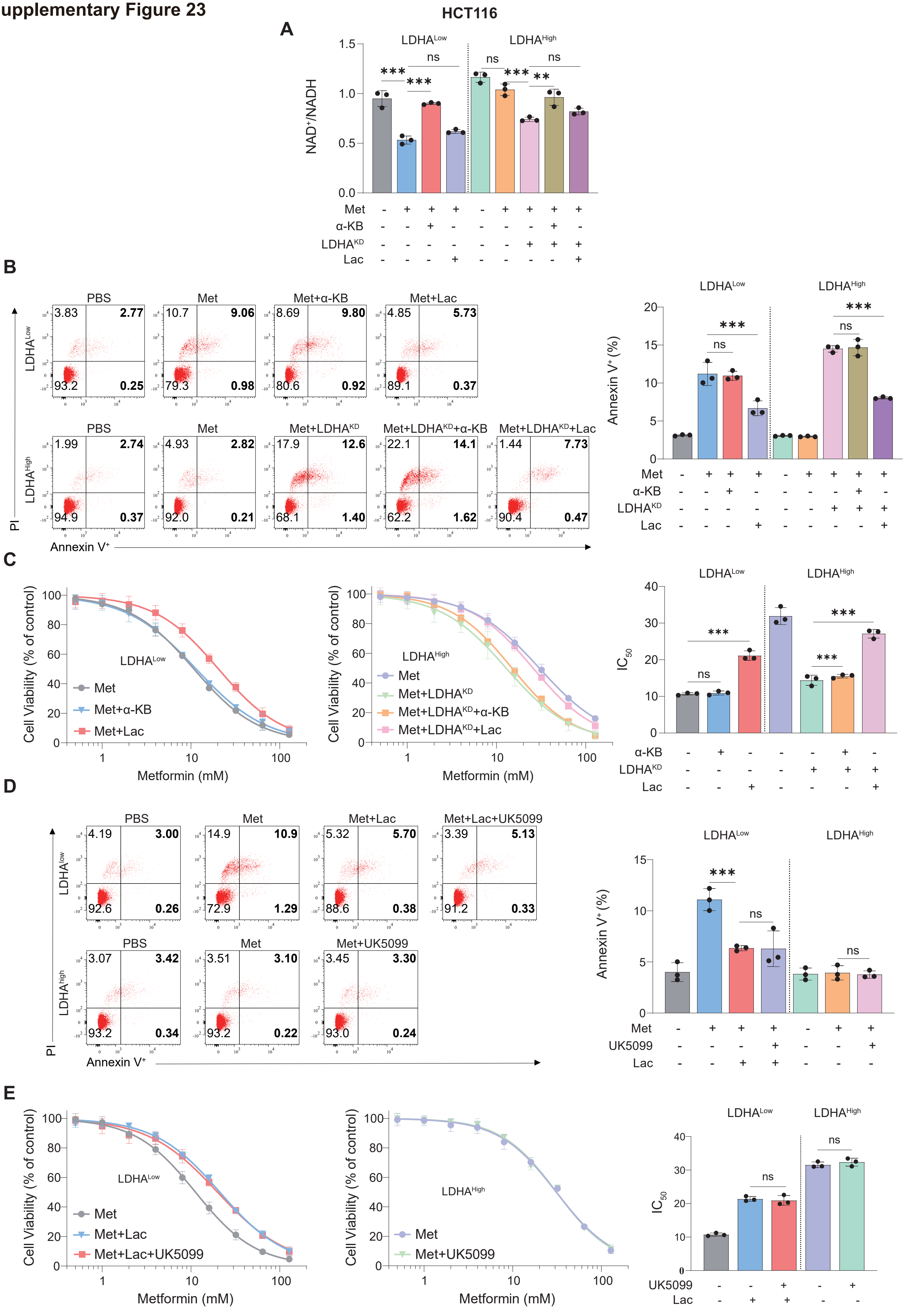

Supplementary Figure 24

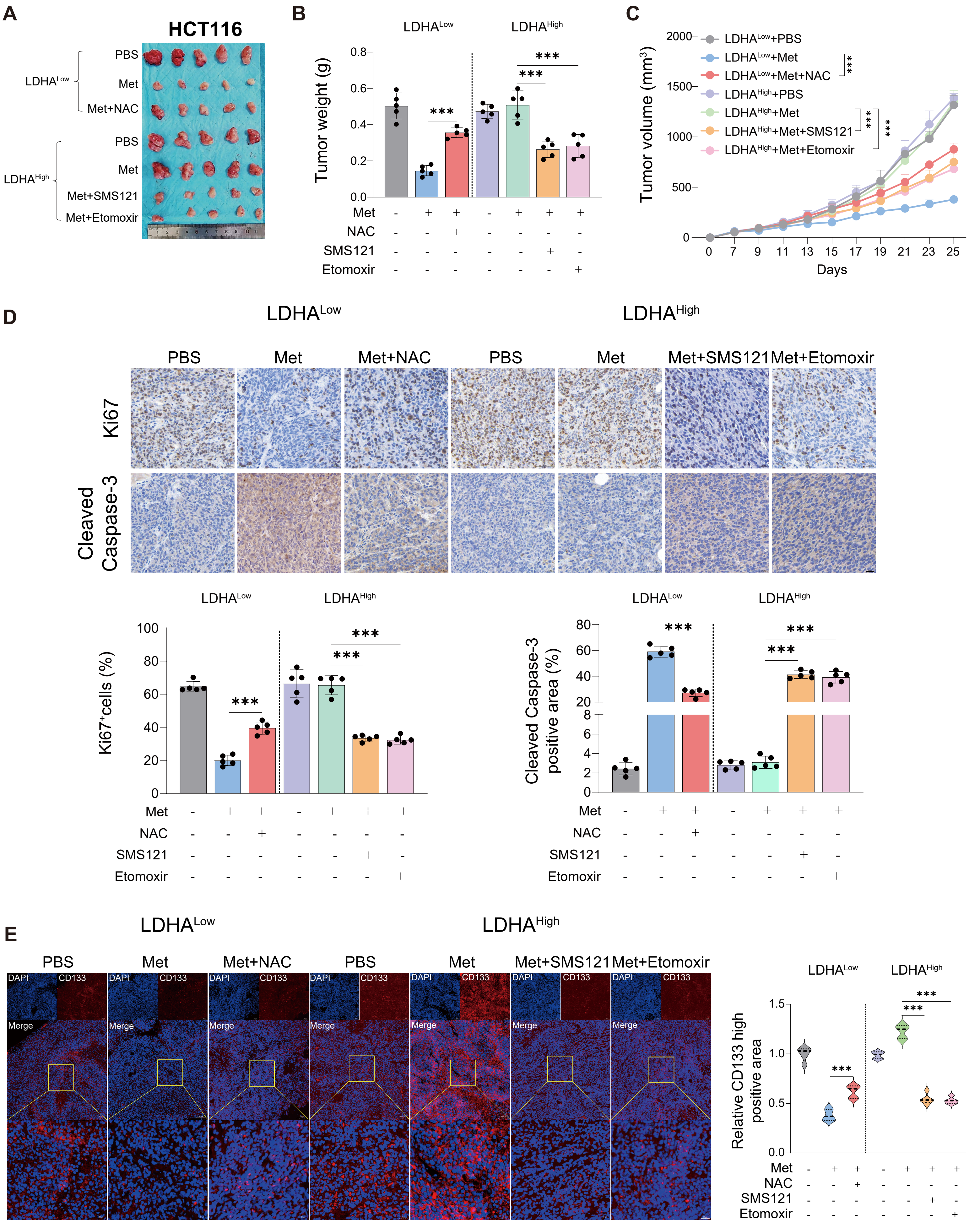

Supplementary Figure 25

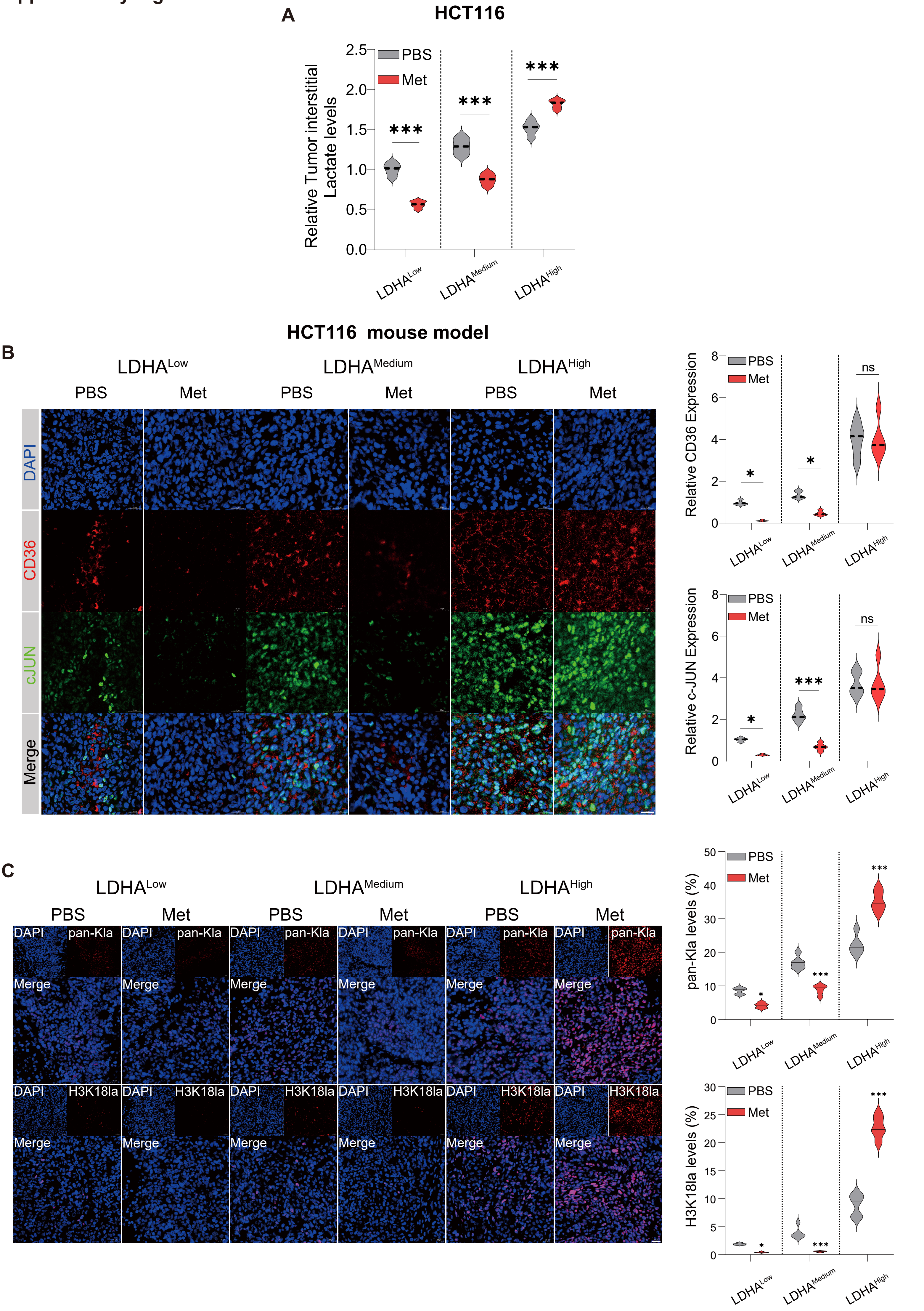

Supplementary Figure 26

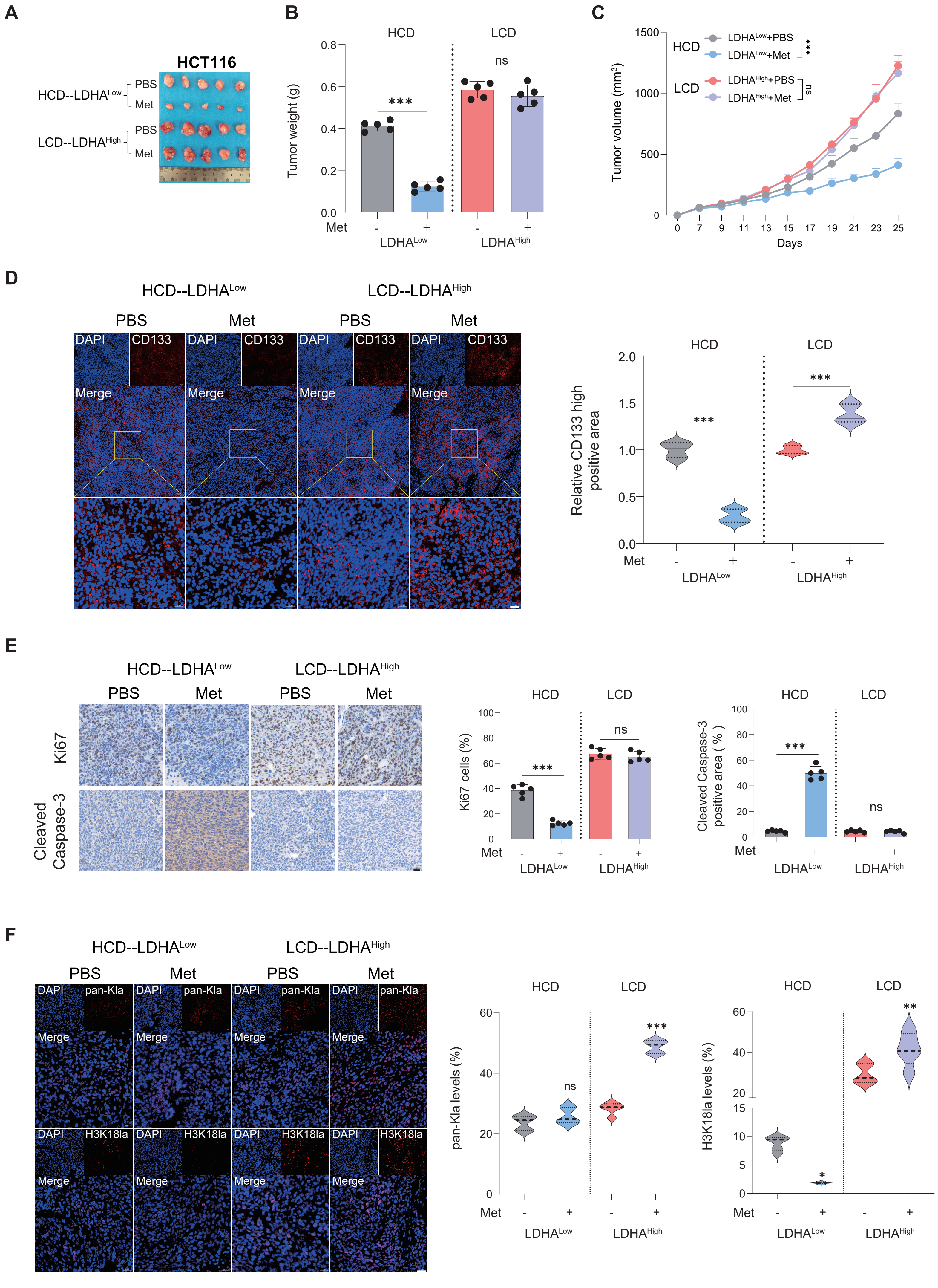

Supplementary Tables

Supplementary Table 1. Putative binding sequences of c-JUN in *CD36* promoter region.

Name	Score	Relative score	Start	End	Strand	Predicted sequence
c-JUN	12.456	0.925	1676	1689	+	gaattctgactcat
c-JUN	10.943	0.906	1849	1862	+	ggaaactgactcaa

Supplementary Table 2. Primers for qRT-PCR.

Primer Name	Sequences (5'-3')
OCT4-Forward	GGTTGAGTAGTCCCTTCGC
OCT4-Reverse	CACATCGGCCTGTGTATATC
NANOG-Forward	TTTGTGGGCCTGAAGAAAACT
NANOG-Reverse	AGGGCTGTCCTGAATAAGCAG
SOX2-Forward	GCCGAGTGGAAACTTTTGTCG
SOX2-Reverse	GGCAGCGTGTACTTATCCTTCT
MYC-Forward	CGGAACTCTTGTGCGTAAGG
MYC-Reverse	ACTCAGCCAAGGTTGTGAGG
BMI1-Forward	TTGCTTTGGTCGAACTTGG
BMI1-Reverse	GTGCTTCTTTTGCAGACTGG
CD44-Forward	CTGCCGCTTTGCAGGTGTA
CD44-Reverse	CATTGTGGGCAAGGTGCTATT
CD133-Forward	GATTCATACTGGTGGCTGGGTGG
CD133-Reverse	GCAGGTGAAGAGTGCCGTAAGT
LGR5-Forward	CTTCCAACCTCAGCGTCTTC
LGR5-Reverse	TTTCCCGCAAGACGTAACTC
VIMENTIN-Forward	ACACCCTGCAATCTTTCAGACA
VIMENTIN-Reverse	GATTCCACTTTGCGTTCAAGGT
ZEB1-Forward	AAGAAAGTGTTACAGATGCAGCTG
ZEB1-Reverse	CCCTGGTAACACTGTCTGGTC
SNAIL-Forward	CCTCCCTGTCAGATGAGGAC
SNAIL-Reverse	CAAGGAATACCTCAGCCTGG
SLUG-Forward	ACAGCGAACTGGACACACAT
SLUG-Reverse	GATGGGGCTGTATGCTCCT
E-CADHERIN-Forward	AGGCCAAGCAGCAGTACATT
E-CADHERIN-Reverse	ATTCACATCCAGCACATCCA
ABCB1-Forward	TTGCTGCTTACATTCAGGTTTCA
ABCB1-Reverse	AGCCTATCTCCTGTCGCATTA
β-ACTIN-Forward	AAGATCAAGATCATTGCTCCTC
β-ACTIN-Reverse	CAACTAAGTCATAGTCCGCC
CD166-Forward	CCAAGATGGAGGAGGAATATG
CD166-Reverse	CCATTCCTGTACCATGTGAT
KLF4-Forward	CCCACATGAAGCGACTTCCC
KLF4-Reverse	CAGGTCCAGGAGATCGTTGAA
EPCAM-Forward	CGCAGCTCAGGAAGAATGTG

EPCAM-Reverse	TGAAGTACACTGGCATTGACG
ABCC1-Forward	ATGTCACGTGGAATACCAGC
ABCC1-Reverse	GAAGACTGAACTCCCTTCCT
ABCG2-Forward	AGATGGGTTTCCAAGCGTTCAT
ABCG2-Reverse	CCAGTCCCAGTACGACTGTGACA
CYP2J2-Forward	TGTCAAAGCACACAGGCAATCC
CYP2J2-Reverse	GCAGAGCCCATCGCAGAGTT
CYP2C8-Forward	TTTCATCCTGGGCTGTGCT
CYP2C8-Reverse	AGTAGAGGGAAATTATTGCAGACC
CYP2C9-Forward	AAGAGGAGCATTGAGGACCG
CYP2C9-Reverse	GCACAGCCCAGGATGAAAGT
CYP2C18-Forward	AAGGGCACGACCATAATAACA
CYP2C18-Reverse	TGCTGAGAAAGGCATGAAGTAG
CYP2C19-Forward	ACGTGTCATTGGCAGAAACC
CYP2C19-Reverse	CCCTTGGGAATGAGGTAGTTT
CYP1A2-Forward	AACAAGGGACACAACGCTGAAT
CYP1A2-Reverse	GGAAGAGAAACAAGGGCTGAGT
CYP2E1-Forward	ACAGAGACCACCAGCACAACT
CYP2E1-Reverse	ATGAGCGGGGAATGACACAGA
CYP3A4-Forward	CCTTACACATACACACCCTTTGGAAGT
CYP3A4-Reverse	CATGTACAGAATCCCCGGTTA
ALOX15-Forward	ACCTTCCTGCTCGCCTAGTGTT
ALOX15-Reverse	GGCTACAGAGAATGACGTTGGC
ALOX5-Forward	GGAGAACCTGTTCATCAACCGC
ALOX5-Reverse	CAGGTCTTCCTGCCAGTGATTC
SLC27A2-Forward	TGTGCCAGGTCATGAGGGTCG
SLC27A2-Reverse	CTCAATGGTATCTTGTATCCTCAGGAA
SLC27A4-Forward	GGAACCAGAGCAGAAGTC
SLC27A4-Reverse	GCCACCTACAGATGTCAG
CD36-Forward	GGAGCCATCTTTGAGCCTTCA
CD36-Reverse	GAACCAAACTGAGGAATGGATCT
EPHX1-Forward	GATCGATAAGTTCCGTTTCACC
EPHX1-Reverse	ATCCTTAGTCTTGAAGTGAGGAT
FADS2-Forward	GACCACGGCAAGAACTCAAAG
FADS2-Reverse	GAGGGTAGGAATCCAGCCATT

Supplementary Table 3. siRNA sequences for gene silencing.

Gene	GenBank accession number	Target sequence (5′–3′)
GPR81	NM_032554.3	CTGCTAGACTCTATTTCCT
LDHA	NM_001165414.1	GCCAUCAGUAUCUUAAUGATT

Supplementary Table 4. Primers for ChIP-PCR.

Primer	Sequences (5’-3’)
CD36 promoter (-325, -312)-Forward	TGGATGGCAGAGCGAGACTCCGTTT

CD36 promoter (-325, -312)-Reverse	CTTGTCCACTCTTTAACTCTGAGCT
CD36 promoter (-152, -139)-Forward	TAGAAAGCTCAGAGTTAAAGAGTGG
CD36 promoter (-152, -139)-Reverse	CTCCTTTCCTGTTTTTCTCACCTCT

Supplementary Table 5. Primers for constructing CD36 promoter and site-directed mutagenesis.

Primer Name	Sequences (5’-3’)
CD36-promoter-F	CTAGCTAGCTGAAAAGGACACTAAGGATGCAAAGGT
CD36-promoter-R	CCCAAGCTTGCTTTTGGATGGACTAACCCCGTGATG
CD36-325-mutation-312-F	AGaattcatgcgttttGATAGATTTGTTTAGAAATGTTTTTCTTTTT
CD36-325-mutation-312-R	TCaaaacgcatgaattCTTTTTCCTTTCCTTTTCTTCTTTTC
CD36-152-mutation-139-F	ggaaacatgcgttaATACACGGAAAACAGGATTTAGTAAACG
CD36-152-mutation-139-R	GTATtaacgcatgtttccTTATCTTTAAAGTGGAAATAATATCCCAT

Reagent or resource.

Reagents or resource	Source	Cat no.
Antibodies		
Anti-β-actin	ProteinTech	HRP-66009
Anti-c-JUN	ProteinTech	66313-1-Ig
Anti-p-c-JUN	Cell Signaling Technology	9164
Anti-LDHA	ProteinTech	66287-1-Ig
Anti-GPR81	ProteinTech	20146-1-AP
Anti-AMPK	ProteinTech	10929-2-AP
Anti-p-AMPK	Cell Signaling Technology	2535
Anti-ACC	ProteinTech	21923-1-AP
Anti-p-ACC	ProteinTech	29119-1-AP
Anti-FASN	ProteinTech	10624-2-AP
Anti-Ki67	ProteinTech	28074-1-AP
Anti-Cleaved Caspase 3	ProteinTech	25128-1-AP
Anti-CD133	Cell Signaling Technology	64326
Alexa Fluor® 555-conjugated anti-rabbit IgG secondary antibody	Cell Signaling Technology	4413
Anti-p300	Cell Signaling Technology	86377
Acetylated-Lysine (Ac-K) Antibody	Cell Signaling Technology	9441
Anti-CD36	Abcam	ab252922
PE anti-human CD133	BioLegend	397904
Anti-Histone H3	PTM BIO	PTM-3015
Anti-L-Lactyl-Histone H3 (Lys18)	PTM BIO	PTM-1427RM
Anti-L-Lactyl Lysine	PTM BIO	PTM-1401RM
Anti-L-Lactyl-Histone H3 (Lys18)	PTM BIO	PTM-1406RM
Anti-Histone H3 (acetyl K18)	Abcam	ab1191
Chemicals, peptides, and recombinant proteins		
RPMI 1640	EallBio	03.4007C

Glucose-free RPMI 1640	EallBio	03.4003C
Fetal bovine serum (FBS)	HyClone	SH30084.03
Puromycin	Boster Biol Tech	AR1007
1% penicillin/streptomycin	Boster Biol Tech	AR1203
D-glucose	Sigma-Aldrich	D8270
L-glutamine	Sigma-Aldrich	G3126
L-(+)-Lactic acid	Sigma-Aldrich	L-6402
Linoleic acid	Sigma-Aldrich	L5900
ActD	Sigma-Aldrich	SBR00013
α-KB	Sigma-Aldrich	K401
RIPA	Boster Biol Tech	AR0102
PMSF	Boster Biol Tech	AR1192
DMEM/F12	Gibco	11320033
B27	Gibco	17504044
EGF	Gibco	PHG0313
DGAT1i	MedChemExpress	A922500
ATGLi	MedChemExpress	HY-15859
C646	MedChemExpress	HY-13823
SMS121	MedChemExpress	HY-163541
CHX	MedChemExpress	HY-12320
5-FU	MedChemExpress	HY-90006
CAPE	MedChemExpress	HY-B0016
CPT-11	MedChemExpress	HY-16562
L-OHP	MedChemExpress	HY-17371
NAC	MedChemExpress	HY-B0215
UK5099	MedChemExpress	HY-90349
SP600125	MedChemExpress	HY-12041
TRIzol	Invitrogen	T9424
DAPI dye	Beyotime Biotechnology	C1005
XF RPMI base medium	Agilent Technologies	103576-100
ECL	Pierce	23891
Metformin HCl	APEXBio	B1970
bFGF	R&D	233-FB
JC63.1	Abcam	ab23680
Cell Lysis Buffer (10X)	Cell Signaling Technology	9803
α-CHCA	Selleck	S8612
Critical commercial assays		
CCK-8 assay kit	LABLEAD	CK001
Apoptosis Analysis Kit	LABLEAD	C1002
NADPH Assay kit	Abbkine	KTB1010
NAD^+/NADH Assay kit	Abbkine	KTB1020
L-Lactate Assay kit	Abcam	ab65330
FAO Colorimetric Assay Kit	Elabscience	E-BC-K784-M
ATP Assay kit	Beyotime	S0027

ROS Assay Kit	Beyotime	S0033
Oil Red O Assay Kit	Beyotime	C0158
Glucose detection kit	Beyotime	S0201
TG detection kit	Beyotime	S0219S
Lipid peroxidation Kit	Beyotime	S0043S
ChIP assays Kit	Cell Signaling Technology	9003S
MutExpress II Fast Mutagenesis Kit	Vazyme	C214-01
CD133 MicroBead Kit	Miltenyi Biotec	130-097-049
MS Columns	Miltenyi Biotec	130-042-201
MidiMACS Separator	Miltenyi Biotec	130-042-302
MultiStand	Miltenyi Biotec	130-042-303
XF Cell Energy Phenotype Test Kit	Agilent Technologies	103325-100
XF Cell Mito Stress Test kit	Agilent Technologies	103015-100
XF Glycolytic Rate Assay Kit	Agilent Technologies	103344-100
Long-chain fatty acid oxidation stress test	Agilent Technologies	103672-100
GFR OrganoGel Phenol red free	Youningwei Biotechnology	abs9495
Organoid construction and culture kits	Youningwei Biotechnology	abs9445
Experimental models: organisms/strains		
BALB/c nude mice	Vital River	N/A
BALB/c mice	Vital River	N/A
Experimental models: cell lines		
HCT116	ATCC	CCL-247
SW620	ATCC	CCL-227
HCT15	ATCC	CCL-225
Oligonucleotides		
See Table S2 for qRT-PCR primers	BGI-write	This paper
See Table S3 for siRNA sequences	RiboBio	This paper
See Table S4 for ChIP primers	BGI-write	This paper
See Table S5 for constructing CD36 promoter primers and mutation primers	BGI-write	This paper
Software and algorithms		
GraphPad Prism 10	GraphPad	https://www.graphpad.com/scientificsoftware/prism
FlowJo V10	FlowJo, LLC	https://www.flowjo.com/solutions/flowjo
Fiji	NIH	https://fiji.sc
BioRender	BioRender	https://app.biorender.com
Integrative Genomics Viewer (IGV)	Broad Institute	http://software.broadinstitute.org/software/igv/

Supplementary Figure Legends

Supplementary Figure 1. High carbohydrate diet attenuates the antitumor effect of metformin in xenograft and CT26 models.

(A–D) Random blood glucose (**A, C**) and intratumoral glucose (**B, D**) measured at 0, 3, and 6 h after Met administration in mice bearing HCT116 (**A, B**) or SW620 (**C, D**) tumors under low-carbohydrate diet (LCD) or high-carbohydrate diet (HCD) conditions (n = 5 mice per group).

(E) Representative endpoint CT26 tumors from mice treated with phosphate-buffered saline (PBS) or Met under LCD or HCD feeding conditions.

(F) Tumor growth curves during treatment (n = 5 mice per group).

(G) Tumor weights at the endpoint, stratified by diet and treatment (n = 5 mice per group).

(H) Longitudinal random blood glucose during the experiment (tail-vein sampling every 3 days).

(I) Intratumoral glucose at endpoint (n = 5 mice per group).

(J, K) Random blood glucose (**J**) and intratumoral glucose (**K**) at 0, 3, and 6 h after Met administration in CT26-bearing mice under LCD or HCD (n = 5 mice per group).

(L) Immunohistochemistry (IHC) for Ki67 and Cleaved Caspase-3 in CT26 tumors with quantitative analysis (n = 5 mice per group).

(M) Immunofluorescence (IF) for CD133 in CT26 tumors with quantitative analysis (n = 5 mice per group).

Mice received intraperitoneal Met at 200 mg/kg every other day. Blood glucose was measured via tail-vein sampling; intratumoral glucose was measured as indicated.

Data are presented as mean ± SD. Statistical significance was determined by two-sided two-way ANOVA followed by Tukey's multiple-comparison test for **A–D** and **G–M**. Tumor growth curves in **F** were analyzed by ordinary two-way ANOVA followed by Tukey's multiple-comparison test. Scale bars, 20 μm for IHC images **(L)** and IF images **(M)**. LCD, low-carbohydrate diet; HCD, high-carbohydrate diet; Met, metformin; PBS, phosphate-buffered saline; ns, no significance; *$P < 0.05$; **$P < 0.01$; ***$P < 0.001$. Source data are provided as a Source Data file.

Supplementary Figure 2. High-glucose drinking water attenuates the antitumor effect of metformin in CRC.

(A) Peripheral glucose levels in mice receiving normal water or 30% glucose water (D30) for 3 weeks (n = 10 mice per group).

(B) Intratumoral glucose levels in mice receiving normal or D30 water and treated with PBS or Met (n = 5 mice per group).

(C) Representative tumor images and final tumor weight quantification (n = 5 mice per group).

(D) Tumor growth curves over the course of treatment (n = 5 mice per group).

(E) IHC for Cleaved Caspase-3 and Ki67 with quantification (n = 5 mice per group).

(F) IF for CD133 with quantification (n = 5 mice per group).

Data are presented as mean ± SD. Statistical significance was determined by two-sided unpaired Student's t-test for **A** and **C**, and by two-sided two-way ANOVA followed by Tukey's multiple-comparison test for **B**, **E** and **F**. Tumor growth curves in **D** were analyzed by ordinary two-way ANOVA followed by Tukey's multiple-comparison test. Scale bars, 20 μm for IHC images (E) and IF images (F). Met, metformin; D30, 30% dextrose water; ns, no significance; *$P < 0.05$; **$P < 0.01$; ***$P < 0.001$. Source data are provided as a Source Data file.

***P < 0.001. Source data are provided as a Source Data file.

Supplementary Figure 3. $CD133^+$ colorectal CSCs exhibit resistance to metformin in high glucose conditions in different cell lines.

(A) Western blots showing the effect of Met on CD133 expression in HCT116 and SW620 cells cultured at different glucose concentrations. CD133 and β-actin were detected on the same blot, and β-actin served as the loading control.

(B–D) In HCT15 cells: Western blot (**B**), immunofluorescence (**C**; DAPI, blue; CD133, red), and flow cytometry (**D**) examining CD133 levels or the proportion of $CD133^+$ cells after Met treatment under low glucose (LG) or high glucose (HG) (n = 3 independent biological replicates). CD133 and β-actin were detected on the same blot, and β-actin served as the loading control.

(E) Percentage of cell death in HCT116 and SW620 cultured under LG or HG with PBS, 5-fluorouracil (5-FU; 10 μM for HCT116, 20 μM for SW620), capecitabine (CAPE; 10 μM for HCT116, 5 μM for SW620), irinotecan (CPT-11; 2 μM for HCT116, 2 nM for SW620), or oxaliplatin (L-OHP; 2 μM for HCT116, 1 μM for SW620) (n = 3 independent biological replicates).

(F) Gene expression in HCT15, presented as fold change relative to PBS (n = 3 independent biological replicates).

(G) Colony-formation assay of $CD133^+$ cells from HCT116, SW620, and HCT15 under LG or HG with or without Met; representative images (left) and colony counts (right) (n = 3 independent biological replicates).

(H) Sphere-formation assays of $CD133^+$ cells from the indicated cell lines were performed under LG or HG conditions with or without Met. Representative images are shown at the top, and relative sphere-formation efficiency is shown at the bottom (n = 3 independent biological replicates for HCT116; n = 5 independent biological replicates for SW620 $CD133^+$ and HCT15 $CD133^+$).

(I, J) Flow-cytometric analysis of apoptosis in $CD133^+$ (**I**) and $CD133^−$ (**J**) HCT15 cells under LG or HG with or without Met; Annexin V/PI plots and quantification (n = 3 independent biological replicates).

Data are presented as mean ± SD. Statistical significance was determined by two-sided unpaired Student's t-test for gene-expression comparisons in **F** and for LG versus HG comparisons within each treatment in **E**. For factorial comparisons in **D**, **G**, **H**, **I** and **J**, statistical significance was determined by two-sided two-way ANOVA followed by Tukey's multiple-comparison test. Scale bars, 75 μm for sphere images **(H)**. LG, low glucose; HG, high glucose; Met, metformin; ns, no significance; *P < 0.05; **P < 0.01; ***P < 0.001. Source data are provided as a Source Data file.

Supplementary Figure 4. $CD133^+$ cells in patient-derived organoids remain proliferatively active under high-glucose conditions despite metformin treatment.

(A, B) IF of PDOs from two patients (P1 and P2) cultured under LG or HG with or without Met; co-staining of CD133 (red) and Ki67 (green); DAPI (blue). Quantification of Ki67 mean fluorescence intensity (MFI) in $CD133^+$ cells is shown below.

(C, D) H&E staining of PDO sections from P1 (**C**) and P2 (**D**) under LG or HG with or without Met. Cell-diameter quantification indicates no change in cell size.

Box plots show the median and interquartile range; whiskers indicate the range. For **A** and **B**, relative Ki67 MFI in $CD133^+$ cells was quantified from individual organoids in different Matrigel domes under the indicated treatment conditions. For **C** and **D**, cell diameter was quantified from

individual organoids in different Matrigel domes under the indicated treatment conditions. Representative images are shown. Statistical significance was determined by two-sided two-way ANOVA followed by Tukey's multiple-comparison test for **A–D**. Scale bars, 20 µm **(A–D)**. PDOs, patient-derived organoids; LG, low glucose; HG, high glucose; Met, metformin; ns, no significance; *P < 0.05; **P < 0.01; ***P < 0.001. Source data are provided as a Source Data file.

Supplementary Figure 5. Differential metabolites and gene expression related to the LA metabolism pathway in metformin-sensitive and -resistant cells.

(A) Heatmap of 446 overlapping intracellular metabolites in Met-sensitive vs Met-resistant cells (Z-score normalized).

(B, C) Gene-expression changes in CD133$^+$ CSCs, normalized to PBS (n = 3 independent biological replicates).

Data are presented as mean ± SD. Statistical significance was determined by two-sided unpaired Student's t-test for each gene in **B** and **C**, as indicated. HG, high glucose; LG, low glucose; Met, metformin; ns, no significance; *P < 0.05; **P < 0.01; ***P < 0.001. Source data are provided as a Source Data file.

Supplementary Figure 6. CD36 expression is elevated in CD133$^+$ colorectal CSCs exposed to high glucose plus metformin and validated by genetic modulation.

(A) Western blots of CD36 in the HCT116 mouse model under LCD or HCD, treated with PBS or Met. CD36 and β-actin were detected on the same blot, and β-actin served as the loading control.

(B, C) Western blots of CD36 in CD133$^-$ and CD133$^+$ subpopulations of HCT15 (**B**) and SW620 (**C**) under LG or HG with or without Met. CD36 and β-actin were detected on the same blot, and β-actin served as the loading control.

(D, E) Validation of CD36 overexpression and knockdown in HCT116 (**D**) and SW620 CD133$^+$ (**E**) using control vector (Ctrl^{OE}), CD36 overexpression (CD36^{OE}), control shRNA (Ctrl^{KD}), or CD36-targeting shRNAs (CD36$^{KD-1/-2/-3}$). CD36 and β-actin were detected on the same blot, and β-actin served as the loading control.

Representative results from five independent experiments. LCD, low-carbohydrate diet; HCD, high-carbohydrate diet; LG, low glucose; HG, high glucose; Met, metformin. Source data are provided as a Source Data file.

Supplementary Figure 7. DGAT1i inhibits LD formation in metformin-resistant CD133$^+$ CSCs.

(A, B) Oil Red O staining and quantification of lipid droplets (LDs) in CD133$^+$ CSCs cultured under LG or HG conditions in the presence of 50 µM linoleic acid (LA), and treated with PBS or Met, with or without CD36 overexpression (CD36^{OE}) or DGAT1 inhibitor (DGAT1i) as indicated (n = 3 independent biological replicates).

(C) Phase-contrast and Oil Red O images of parental and metformin-resistant (MetR) HCT116 cells treated with Met alone, Met plus DGAT1i for 24 h, or Met plus DGAT1i for 24 h followed by DGAT1i withdrawal and washout for 48 h (n = 5 independent biological replicates).

(D) Flow-cytometric apoptosis analysis of parental and MetR HCT116 cells under the conditions described in (C). Annexin V$^+$ percentages are shown (n = 3 independent biological replicates).

Data are presented as mean ± SD. Statistical significance was determined by ordinary one-way

ANOVA followed by Tukey's multiple-comparison test for comparisons involving multiple treatment groups in **A–D**. Scale bars, 10 µm **(A, B)** and 20 µm **(C)**. LG, low glucose; HG, high glucose; LA, linoleic acid; Met, metformin; DGAT1i, diacylglycerol acyltransferase 1 inhibitor; LD, lipid droplet; ns, no significance; *P < 0.05; **P < 0.01; ***P < 0.001. Source data are provided as a Source Data file.

Supplementary Figure 8. High glucose promotes lipid droplet accumulation through enhanced fatty acid uptake and oxidation to sustain energy and redox balance in metformin-resistant colorectal CSCs.

(A) Intracellular triglycerides (TG) in HCT116 and SW620 $CD133^+$ cells under LG or HG with or without Met (n = 3 independent biological replicates).

(B) Western blots of AMPK signaling and lipogenic enzymes (p-AMPK, AMPK, p-ACC, ACC, FASN) after Met treatment for 3, 12, or 24 h under LG or HG. AMPK, ACC1, and β-actin were detected on the same blot, whereas p-AMPK, p-ACC1, and FASN were detected on separate blots. The samples derive from the same experiment, and all blots were processed in parallel.

(C) FAO activity under the indicated conditions (n = 3 independent biological replicates).

(D–F) ATP (**D**), NADPH (**E**), and ROS (**F**) levels in HCT116 and SW620 $CD133^+$ cells under LG or HG with or without Met (n = 3 independent biological replicates).

(G) Lipid peroxidation (BODIPY 581/591 C11) in HCT116 cells (n = 3 independent biological replicates).

(H) Heatmap of redox-related metabolites in SW620 $CD133^+$ cells under LG + Met vs HG + Met (Z-score normalized).

(I) Transmission electron microscopy (TEM) of HCT116 cells; increased mitochondrial number and dense cristae under HG + Met; quantification at right (n = 3 independent biological replicates).

(J) Mitochondrial FAO capacity assessed using the Seahorse XF Long Chain Fatty Acid Oxidation Stress Test Kit. Oxygen-consumption rates (OCRs) were continuously monitored; basal, acute, and maximum OCR values were quantified (n = 3 independent biological replicates).

(K) Oil Red O staining of HCT116 tumors treated with PBS, Met, ATGLi, DGAT1i, or combinations; LD area relative to nuclear area quantified (n = 5 mice per group).

(L) Quantification of FAO levels in HCT116 tumors under HCD following the indicated treatments (Met, ATGLi, DGAT1i, or their combinations), with results normalized to the control group (n = 5 mice per group).

Data are presented as mean ± SD. Statistical significance was determined by two-sided two-way ANOVA followed by Tukey's multiple-comparison test for factorial comparisons in **A**, **C**, **D**, **F**, **G** and **I**. For comparisons involving multiple treatment groups in **E**, **K** and **L**, statistical significance was determined by ordinary one-way ANOVA followed by Tukey's multiple-comparison test. For Seahorse FAO quantification in **J**, statistical significance was determined by two-sided unpaired Student's t-test. Scale bars, 500 nm **(I)** and 10 µm **(K)**. LG, low glucose; HG, high glucose; Met, metformin; LD, lipid droplet; FAO, fatty-acid oxidation; FASN, fatty-acid synthase; ACC, acetyl-CoA carboxylase; AMPK, AMP-activated protein kinase; GSH, glutathione; GSSG, oxidized glutathione; NAC, N-acetylcysteine; ATGLi, adipose triglyceride lipase inhibitor; DGAT1i, diacylglycerol acyltransferase 1 inhibitor; ROS, reactive oxygen species; TG, triglycerides; PC, positive control; ns, no significance; *P < 0.05; **P < 0.01; ***P < 0.001. Source data are provided as a Source Data file.

Supplementary Figure 9. Metformin affects lactate production in CSCs under different glucose conditions and LDHA regulates lactate levels.

(A) Relative intratumoral lactate levels from HCT116 xenografts under LCD or HCD, treated with PBS or Met (n = 5 mice per group).

(B, C) Time-course of intracellular (**B**) and extracellular (**C**) Lac in $CD133^+$ CSCs under LG or HG with PBS or Met (n = 3 independent biological replicates).

(D, E) Western blots validating LDHA knockdown with two siRNAs in HCT116 (**D**) and SW620 $CD133^+$ (**E**). LDHA and β-actin were detected on the same blot, with β-actin serving as the loading control.

(F, G) Intracellular (**F**) and extracellular (**G**) Lac after LDHA knockdown (n = 3 independent biological replicates).

Data are presented as the mean ± SD. Statistical significance was determined by two-sided two-way ANOVA with Tukey's multiple-comparison test for **A**, and by two-sided unpaired Student's t-test for **F** and **G** where indicated. LCD, low-carbohydrate diet; HCD, high-carbohydrate diet; LG, low glucose; HG, high glucose; Met, metformin; ns, no significance; $^*P < 0.05$; $^{**}P < 0.01$; $^{***}P < 0.001$. Source data are provided as a Source Data file.

Supplementary Figure 10. Metformin does not alter GPR81 or LDHA expression, and lactate regulates CD36 independently of GPR81 signaling and extracellular acidification.

(A) Western blot analysis of GPR81 and LDHA expression in $CD133^+$ CSCs under LG and HG conditions, treated with PBS or Met. LDHA and β-actin were detected on the same blot, whereas GPR81 was detected on a separate blot. The samples derive from the same experiment, and the blots were processed in parallel.

(B) Western blot analysis of CD36 and GPR81 expression in $CD133^+$ CSCs under LG conditions, treated with PBS or Met, Lac, siGPR81, α-CHCA, and HCl. CD36 and β-actin were detected on the same blot, whereas GPR81 was detected on a separate blot. The samples derive from the same experiment, and the blots were processed in parallel.

HG, high glucose; LG, low glucose; Met, metformin; Lac, lactate; HCl, hydrochloric acid. Source data are provided as a Source Data file.

Supplementary Figure 11. Metformin and lactate do not alter the mRNA stability or protein degradation of CD36 and c-JUN.

(A, B) CSCs treated with actinomycin D (ActD, 0.5 μg/mL) under LG or HG with PBS or Met (10 mM); *CD36* and *c-JUN* mRNA measured by qPCR over time (n = 3 independent biological replicates).

(C–F) CSCs treated with cycloheximide (CHX, 10 μM) under LG or HG with or without Met; representative Western blots (**C, D**) and quantitative analysis of protein degradation (**E, F**) (n = 3 independent biological replicates). CD36 and β-actin were detected on the same blot, whereas c-JUN was detected on a separate blot. The samples derive from the same experiment, and the blots were processed in parallel.

(G, H) ActD treatment under LG or HG in the presence or absence of exogenous Lac (10 mM); *CD36* and *c-JUN* mRNA by qPCR over time (n = 3 independent biological replicates).

(I–L) CHX treatment under LG or HG with or without Lac; Western blots (**I, J**) and degradation

analysis (**K, L**) (n = 3 independent biological replicates). CD36 and β-actin were detected on the same blot, whereas c-JUN was detected on a separate blot. The samples derive from the same experiment, and the blots were processed in parallel.

Data are presented as mean ± SD. Statistical significance was determined by two-sided two-way ANOVA followed by Tukey's multiple-comparison test for **A**, **B**, E–H, **K** and **L**, with comparisons performed at the indicated time points or at the final time point, as indicated. Representative western blots are shown in C, D, I and J. LG, low glucose; HG, high glucose; Met, metformin; Lac, lactate; CHX, cycloheximide; ns, no significance; *P < 0.05; **P < 0.01; ***P < 0.001. Source data are provided as a Source Data file.

Supplementary Figure 12. Functional modulation of c-JUN alters metformin sensitivity in CD133^{+} colorectal CSCs.

(A) Western blots confirming c-JUN overexpression (c-JUN^{OE}) and knockdown (c-JUN$^{KD-1/-2/-3}$) in HCT116 and SW620 CD133^{+} CSCs. c-JUN and β-actin were detected on separate blots. The samples derive from the same experiment, and the blots were processed in parallel.

(B, C) Dose–response curves and IC_{50} values for Met in HCT116 (**B**) and SW620 CD133^{+} (**C**) under LG or HG following c-JUN overexpression, knockdown, or pharmacologic inhibition of upstream JNK signaling (SP600125; JNKi) (n = 3 independent biological replicates).

(D, E) Flow-cytometric apoptosis in HCT116 (**D**) and SW620 CD133^{+} (**E**) for the same conditions; Annexin V/PI plots and quantification (n = 3 independent biological replicates).

Data are presented as mean ± SD. Statistical significance was determined by ordinary one-way ANOVA followed by Tukey's multiple-comparison test for comparisons involving multiple treatment groups in **B–E**. LG, low glucose; HG, high glucose; Met, metformin; JNKi, JNK inhibitor; ns, no significance; *P < 0.05; **P < 0.01; ***P < 0.001. Source data are provided as a Source Data file.

Supplementary Figure 13. In vivo suppression of c-JUN signaling restores metformin sensitivity in CD133^{+} colorectal CSCs.

(A) Representative images and endpoint tumor-weight quantification from HCT116 xenografts under LCD or HCD. Mice received PBS, Met, Met with c-JUN overexpression (c-JUN^{OE}), Met with c-JUN knockdown (c-JUN^{KD}), or Met with JNKi (SP600125) (n = 5 mice per group).

(B) Tumor growth curves over time (n = 5 mice per group).

(C) IHC for Ki67 and Cleaved Caspase-3 with quantification (n = 5 mice per group).

(D) IF for CD133 with quantification of the CD133-high area (n = 5 mice per group).

Data are presented as mean ± SD. Statistical significance was determined by ordinary one-way ANOVA followed by Tukey's multiple-comparison test for comparisons involving multiple treatment groups in **A**, **C** and **D**. Tumor growth curves in **B** were analyzed by ordinary two-way ANOVA followed by Tukey's multiple-comparison test. Scale bars, 20 µm **(C, D)**. LCD, low-carbohydrate diet; HCD, high-carbohydrate diet; Met, metformin; JNKi, JNK inhibitor; ns, no significance; *P < 0.05; **P < 0.01; ***P < 0.001. Source data are provided as a Source Data file.

Supplementary Figure 14. High-glucose conditions enhance H3K18 lactylation and c-JUN expression in metformin-resistant CD133^{+} CRC models.

(**A**) IF images and quantification of pan-Kla and H3K18la in CD133^{+} CRC tissues from mice under LCD or HCD with PBS or Met (n = 5 mice per group).

(B) Western blots of H3K18la in tumor tissues from the same model. H3K18la (PTM-1427RM) and β-actin were detected on the same blot, whereas H3K18la (PTM-1406RM) and Histone H3 were detected on separate blots. The samples derive from the same experiment, and all blots were processed in parallel.

(C) Western blots quantifying pan-Kla and H3K18la in HCT116 and SW620 $CD133^+$ CSCs under LG or HG with or without Met (n = 3 independent biological replicates).

Data are presented as mean ± SD. Statistical significance was determined by two-sided two-way ANOVA followed by Tukey's multiple-comparison test for **A** and **C**. Scale bars, 20 μm **(A)**. LCD, low-carbohydrate diet; HCD, high-carbohydrate diet; LG, low glucose; HG, high glucose; Met, metformin; ns, no significance; *P < 0.05; **P < 0.01; ***P < 0.001. Source data are provided as a Source Data file.

Supplementary Figure 15. Inhibition of histone lactylation reverses lactate-induced metformin resistance in $CD133^+$ colorectal CSCs.

(A, B) HCT116 cells cultured under LG or HG conditions were treated with PBS or Met, with Lac supplementation and/or the p300 inhibitor C646 as indicated (n = 3 independent biological replicates). **(A)** Representative flow cytometry plots of Annexin V/PI staining and quantification of Annexin V^+ apoptotic cells. **(B)** Met dose-response cell viability curves and quantification of IC_{50} values.

(C, D) SW620 $CD133^+$ CSCs were treated as in **(A, B)** (n = 3 independent biological replicates). **(C)** Representative Annexin V/PI flow cytometry plots and quantification of Annexin V^+ cells. **(D)** Met dose-response viability curves and IC_{50} quantification.

Data are presented as mean ± SD. Statistical significance was determined by ordinary one-way ANOVA followed by Tukey's multiple-comparison test for **A–D.** LG, low glucose; HG, high glucose; Met, metformin; Lac, lactate; C646, p300 inhibitor; ns, no significance; *P < 0.05; **P < 0.01; ***P < 0.001. Source data are provided as a Source Data file.

Supplementary Figure 16. c-JUN/CD36 acts downstream of histone lactylation to sustain metformin resistance in $CD133^+$ colorectal CSCs.

(A) Western blot analysis of c-JUN, p-c-JUN, CD36, pan-Kla, and H3K18la in HCT116 and SW620 $CD133^+$ CSCs cultured under LG or HG conditions and treated with Met and/or Lac, with additional JNKi, the p300 inhibitor C646, c-JUN overexpression (c-JUN^{OE}), or c-JUN knockdown (c-JUN^{KD}) as indicated. CD36, pan-Kla, and β-actin were detected on the same blot, whereas Histone H3, H3K18la, c-JUN, and p-c-JUN were detected on separate blots. The samples derive from the same experiment, and all blots were processed in parallel.

(B, C) HCT116 xenografts established in mice fed an HCD and treated with PBS or Met, with C646 alone or combined with c-JUN^{OE} or $CD36^{OE}$ as indicated. **(B)** Representative endpoint tumors and quantification of tumor weights. **(C)** Tumor growth curves during treatment (n = 5 mice per group).

(D) IHC staining of xenograft tumors for Ki67 and cleaved Caspase-3 under the indicated treatments, with quantitative analysis shown (n = 5 mice per group).

(E) IF staining of xenograft tumors for CD133, with quantification of CD133-high positive areas under the indicated treatments (n = 5 mice per group).

Data are presented as mean ± SD. Statistical significance was determined by ordinary one-way ANOVA followed by Tukey's multiple-comparison test for **B**, **D** and **E**. Tumor growth curves in **C**

were analyzed by ordinary two-way ANOVA followed by Tukey's multiple-comparison test. Scale bars, 20 μm **(D, E)**. LG, low glucose; HG, high glucose; HCD, high-carbohydrate diet; Met, metformin; Lac, lactate; JNKi, JNK inhibitor; C646, p300 inhibitor; ns, no significance; *P < 0.05; **P < 0.01; ***P < 0.001. Source data are provided as a Source Data file.

Supplementary Figure 17. Lipid metabolic remodeling occurs downstream of the lactylation–c-JUN/CD36 axis.

(A) Quantification of relative FAO activity in colorectal CSCs cultured under LG or HG conditions and treated with Met and/or Lac, with additional p300 inhibition C646, c-JUN overexpression (c-JUN^{OE}) or CD36 overexpression ($CD36^{OE}$), and/or c-JUN knockdown (c-JUN^{KD}) or CD36 knockdown ($CD36^{KD}$) as indicated (n = 3 independent biological replicates).

(B) Representative images of Oil Red O (ORO) staining under LG and HG conditions with the indicated treatments, with quantification of LD area normalized to nucleus area shown below (n = 3 independent biological replicates).

Data are presented as mean ± SD. Statistical significance was determined by ordinary one-way ANOVA followed by Tukey's multiple-comparison test for comparisons involving multiple treatment groups in **A** and **B**. Scale bars, 10 μm **(B)**. LG, low glucose; HG, high glucose; Met, metformin; Lac, lactate; C646, p300 inhibitor; ns, no significance; *P < 0.05; **P < 0.01; ***P < 0.001. Source data are provided as a Source Data file.

Supplementary Figure 18. Histone acetylation is dispensable for lactate/LDHA-driven metformin resistance and c-JUN/CD36 activation.

(A) Western blot analysis of pan-Kac and H3K18ac in HCT116 and SW620 $CD133^+$ CSCs cultured under LG or HG conditions with or without Met, as indicated. Histone H3 and β-actin were detected on the same blot, whereas pan-Kac and H3K18ac were detected on separate blots. The samples derive from the same experiment, and all blots were processed in parallel.

(B) ChIP-seq tracks showing the enrichment of H3K18ac at the *c-JUN* promoter region in HCT116 cells under different glucose conditions, treated with PBS or Met. The red box highlights the region of interest (−1500 to +500 relative to the TSS of c-JUN).

(C) Western blot analysis of c-JUN, p-c-JUN, CD36, LDHA, pan-Kac/H3K18ac, and pan-Kla/H3K18la in HCT116 and SW620 $CD133^+$ CSCs cultured under LG or HG and subjected to the indicated treatments, including Met (10 mM), NaAc (10 mM), Lac (10 mM), and/or LDHA knockdown ($LDHA^{KD}$). CD36, LDHA, Histone H3, and β-actin were detected on the same blot. c-JUN and pan-Kla were detected on the same blot, whereas p-c-JUN and pan-Kac were detected on the same blot. H3K18ac and H3K18la were detected on separate blots. The samples derive from the same experiment, and all blots were processed in parallel.

(D) Representative endpoint tumors and quantification of tumor weights in xenograft models under LCD or HCD with the indicated treatments (Met, NaAc, Lac, and/or $LDHA^{KD}$) (n = 5 mice per group).

(E) Tumor growth curves for the corresponding groups shown in **(D)** (n = 5 mice per group).

(F) Representative IHC staining for Ki67 and cleaved Caspase-3 in tumor tissues from the indicated groups, with quantification shown below (n = 5 mice per group).

(G) Representative IF staining of tumor tissues for CD133 and quantification of CD133-high positive areas across groups (n = 5 mice per group).

(H) Representative IF staining of tumor sections from xenografts under LCD or HCD with the indicated treatments (Met, NaAc, Lac, and/or LDHA knockdown (LDHA^{KD})). DAPI (blue), H3K18la (red), and H3K18ac (green) are shown with merged images. Quantification of relative H3K18la and H3K18ac expression is shown below (n = 5 mice per group).

Data are presented as mean ± SD. Statistical significance was determined by ordinary one-way ANOVA followed by Tukey's multiple-comparison test for **D**, **F**, **G** and **H**. Tumor growth curves in **E** were analyzed by ordinary two-way ANOVA followed by Tukey's multiple-comparison test. Scale bars, 20 µm **(F–H)**. LG, low glucose; HG, high glucose; HCD, high-carbohydrate diet; LCD, low-carbohydrate diet; Met, metformin; Lac, lactate; NaAc, sodium acetate; ns, no significance; *P < 0.05; **P < 0.01; ***P < 0.001. Source data are provided as a Source Data file.

Supplementary Figure 19. Sodium acetate fails to mimic lactate-mediated rescue of metformin sensitivity in CD133$^+$ colorectal CSCs.

(A, B) HCT116 cells cultured under LG or HG conditions were treated with PBS or Met, with sodium acetate (NaAc), lactate (Lac), and/or LDHA knockdown (LDHA^KD) as indicated (n = 3 independent biological replicates). **(A)** Representative flow cytometry plots of Annexin V/PI staining and quantification of Annexin V$^+$ apoptotic cells. **(B)** Met dose–response cell viability curves and quantification of IC_{50} values.

(C, D) SW620 CD133$^+$ CSCs were treated as in **(A, B)** (n = 3 independent biological replicates). **(C)** Representative Annexin V/PI flow cytometry plots and quantification of Annexin V$^+$ cells. **(D)** Met dose–response viability curves and IC_{50} quantification.

Data are presented as mean ± SD. Statistical significance was determined by ordinary one-way ANOVA followed by Tukey's multiple-comparison test for **A–D**. LG, low glucose; HG, high glucose; Met, metformin; NaAc, sodium acetate; Lac, lactate; ns, no significance; *P < 0.05; **P < 0.01; ***P < 0.001. Source data are provided as a Source Data file.

Supplementary Figure 20. Time-course and dose–response analyses reveal saturation of lactylation-driven c-JUN/CD36 signaling and lipid metabolic outputs.

(A) Western blot analysis of c-JUN, p-c-JUN, CD36, pan-Kla, and H3K18la in HCT116 and SW620 CD133$^+$ CSCs treated with increasing concentrations of Met (0–2 mM) or Lac (0–10 mM). CD36, Histone H3, and β-actin were detected on the same blot, whereas pan-Kla, H3K18la, c-JUN, and p-c-JUN were detected on separate blots. The samples derive from the same experiment, and all blots were processed in parallel.

(B) Quantification of extracellular lactate levels in culture supernatants from HCT116 and SW620 CD133$^+$ CSCs following Met treatment; the lactate concentration at which c-JUN and CD36 expression reaches a plateau is indicated.

(C) Time-course Western blotting of c-JUN, p-c-JUN, CD36, pan-Kla, and H3K18la in HCT116 cells under HG ± Met, HG + Met + C646, Lac, or Lac + C646 at the indicated time points. CD36, Histone H3, and β-actin were detected on the same blot, whereas pan-Kla, H3K18la, c-JUN, and p-c-JUN were detected on separate blots. The samples derive from the same experiment, and all blots were processed in parallel.

(D) Densitometric quantification of **(C)**, showing the kinetics of c-JUN/p-c-JUN/CD36 versus pan-Kla/H3K18la under HG ± Met and Lac conditions, with or without C646 (n = 3 independent biological replicates).

(E–G) Time-course analysis of lipid metabolic remodeling under HG ± Met or Lac, with or without C646, including FAO activity **(E)**, TG levels **(F)**, and Oil Red O staining with quantification of LD area normalized to nuclear area **(G)** (n = 3 independent biological replicates).

Data are presented as the mean ± SD. Met, metformin; Lac, lactate; HG, high glucose; C646, p300 inhibitor. Statistical significance is shown as indicated in the panels (ns, no significance; *P < 0.05; **P < 0.01; ***P < 0.001). Source data are provided as a Source Data file.

Supplementary Figure 21. Lactate oxidation and mitochondrial pyruvate import are dispensable for metformin resistance in $CD133^+$ colorectal CSCs

(A) Intracellular NAD^+/NADH ratios in HCT116 under LG or HG with the indicated treatments (n = 3 independent biological replicates).

(B, C) Flow-cytometric apoptosis after treatments with Met, α-KB, Lac, or LDHA knockdown ($LDHA^{KD}$) (n = 3 independent biological replicates).

(D, E) Cell viability dose-response curves and IC_{50} values under LG or HG with the indicated treatments (n = 3 independent biological replicates).

(F) Representative oxygen consumption rate (OCR) traces in HCT116 cells treated with PBS or UK5099 (n = 3 independent biological replicates).

(G) Apoptosis following inhibition of mitochondrial pyruvate import with the MPC inhibitor UK5099, in the presence or absence of Lac and Met (n = 3 independent biological replicates).

(H) Dose-response curves and IC_{50} values after Met with or without UK5099 (n = 3 independent biological replicates).

Data are presented as mean ± SD. Statistical significance was determined by ordinary one-way ANOVA followed by Tukey's multiple-comparison test for **A**, **C**, **E**, **G** and **H**. LG, low glucose; HG, high glucose; Met, metformin; Lac, lactate; α-KB, α-ketobutyrate; MPC, mitochondrial pyruvate carrier; UK5099, MPC inhibitor; ns, no significance; *P < 0.05; **P < 0.01; ***P < 0.001. Source data are provided as a Source Data file.

Supplementary Figure 22. High LDHA expression maintains mitochondrial FAO activity and oxidative capacity in metformin-resistant $CD133^+$ colorectal CSCs.

(A) Kaplan–Meier survival analysis stratified by LDHA expression in colon cancer (Kaplan–Meier Plotter database).

(B) FAO levels in HCT116 with low or high LDHA expression after PBS or Met (n = 3 independent biological replicates).

(C) Transmission electron microscopy (TEM) of HCT116 with LDHA-low or LDHA-high treated with PBS or Met to evaluate mitochondrial morphology; mitochondrial number per cell quantified (n = 3 independent biological replicates).

(D) Mitochondrial FAO capacity of HCT116 with LDHA-low or LDHA-high assessed using the Seahorse XF Long Chain Fatty Acid Oxidation Stress Test Kit; OCRs recorded in real time; basal, acute, and maximum OCR values quantified (n = 3 independent biological replicates).

(E) Lipid peroxidation by BODIPY 581/591 C11 in LDHA-low and LDHA-high HCT116 under PBS or Met (n = 3 independent biological replicates).

Data are presented as mean ± SD. Statistical significance was determined by the log-rank (Mantel–Cox) test for **A**. For factorial comparisons in **B**, **C** and **E**, statistical significance was determined by two-sided two-way ANOVA followed by Tukey's multiple-comparison test. For Seahorse FAO

quantification in **D**, statistical significance was determined by two-sided unpaired Student's t-test. Met, metformin; FAO, fatty-acid oxidation; OCR, oxygen-consumption rate; TEM, transmission electron microscopy; PC, positive control; ns, no significance; *P < 0.05; **P < 0.01; ***P < 0.001. Source data are provided as a Source Data file.

Supplementary Figure 23. Lactate oxidation and mitochondrial pyruvate import are dispensable for metformin resistance in $CD133^+$ LDHA-high colorectal CSCs.

(A) Intracellular $NAD^+/NADH$ ratios in HCT116 LDHA-low and LDHA-high $CD133^+$ colorectal CSCs under the indicated treatments (n = 3 independent biological replicates).
(B) Flow cytometry analysis of apoptosis after treatments with Met, α-KB, Lac, or LDHA knockdown ($LDHA^{KD}$) (n = 3 independent biological replicates).
(C) Cell viability dose–response curves and IC_{50} values under LG or HG with the indicated treatments (n = 3 independent biological replicates).
(D) Apoptosis after inhibition of mitochondrial pyruvate import using the MPC inhibitor UK5099 in the presence or absence of Lac and Met (n = 3 independent biological replicates).
(E) Dose-response curves and IC_{50} values after Met with or without UK5099 (n = 3 independent biological replicates).
Data are presented as mean ± SD. Statistical significance was determined by ordinary one-way ANOVA followed by Tukey's multiple-comparison test for comparisons involving multiple treatment groups within each LDHA-expression group in **A–E**. Met, metformin; Lac, lactate; α-KB, α-ketobutyrate; UK5099, mitochondrial pyruvate carrier inhibitor; ns, no significance; *P < 0.05; **P < 0.01; ***P < 0.001. Source data are provided as a Source Data file.

Supplementary Figure 24. Inhibition of fatty-acid uptake or oxidation re-sensitizes $CD133^+$ LDHA-high colorectal xenografts to metformin, whereas ROS scavenging induces resistance in $CD133^+$ LDHA-low tumors.

(A) Representative endpoint tumors from HCT116 xenografts established with LDHA-low or LDHA-high cells and treated with PBS, Met, Met+NAC, Met+CD36 inhibitor (SMS121), or Met+FAO inhibitor (Etomoxir) (n = 5 mice per group).
(B) Endpoint tumor weights (n = 5 mice per group).
(C) Tumor growth curves (n = 5 mice per group).
(D) IHC for Ki67 and Cleaved Caspase-3 with quantification (n = 5 mice per group).
(E) IF for CD133 with quantification of the CD133-high area (n = 5 mice per group).
Data are presented as mean ± SD. Statistical significance was determined by ordinary one-way ANOVA followed by Tukey's multiple-comparison test for **B**, **D** and **E**. Tumor growth curves in **C** were analyzed by ordinary two-way ANOVA followed by Tukey's multiple-comparison test. Scale bars, 20 μm (**D, E**). LDHA, lactate dehydrogenase A; FAO, fatty-acid oxidation; NAC, N-acetylcysteine; Met, metformin; SMS121, CD36 inhibitor; ns, no significance; *P < 0.05; **P < 0.01; ***P < 0.001. Source data are provided as a Source Data file.

Supplementary Figure 25. LDHA status associates with intratumoral lactate and CD36/c-JUN expression during metformin treatment in HCT116 xenografts.

(A) Relative Lac in tumor interstitial fluid from LDHA-low, LDHA-medium, and LDHA-high groups treated with PBS or Met (n = 5 mice per group).

(B) IF of CD36 (red) and c-JUN (green) with DAPI (blue) in the same groups; quantification is shown at right (n = 5 mice per group).
(C) Representative images and quantitative analysis of pan-Kla and H3K18la in LDHA-low, LDHA-medium, and LDHA-high HCT116 xenografts treated with PBS or Met.
Data are presented as mean ± SD. Statistical significance was determined by two-sided two-way ANOVA with Tukey's multiple-comparison test for **A–C**. Scale bars, 20 μm. HCD, high-carbohydrate diet; LCD, low-carbohydrate diet; Met, metformin; ns, no significance; *P < 0.05; **P < 0.01; ***P < 0.001. Source data are provided as a Source Data file.

Supplementary Figure 26. LDHA determines the sensitivity of CD133$^+$ colorectal CSCs to metformin, independently of glucose availability.

(A) CD133$^+$ CSCs with high LDHA expression were transplanted into mice fed a LCD, whereas CD133$^+$ CSCs with low LDHA expression were transplanted into mice fed a HCD. Representative tumors from mice treated with PBS or metformin are shown (n = 5 mice per group).
(B) Tumor weights (n = 5 mice per group).
(C) Tumor growth curves (n = 5 mice per group).
(D) IF for CD133 with quantification (n = 5 mice per group).
(E) IHC for Cleaved Caspase-3 and Ki67 with quantification (n = 5 mice per group).
(F) IF staining and quantification of pan-Kla and H3K18la in HCD–LDHA-low and LCD–LDHA-high CD133$^+$ CRC tissues treated with PBS or Met (n = 5 mice per group).
Data are presented as mean ± SD. Statistical significance was determined by two-sided two-way ANOVA followed by Tukey's multiple-comparison test for **B** and **D–F**. Tumor growth curves in **C** were analyzed by ordinary two-way ANOVA followed by Tukey's multiple-comparison test. Scale bars, 20 μm **(D–F)**. HCD, high-carbohydrate diet; LCD, low-carbohydrate diet; Met, metformin; ns, no significance; *P < 0.05; **P < 0.01; ***P < 0.001. Source data are provided as a Source Data file.
