# Supplementary material for: Glucose-induced histone lactylation confers metformin resistance in colorectal cancer stem cells
Source: Nat Commun. 2026 Sep 7;17:10594. doi: 10.1038/s41467-026-77051-9 (PMC13646297; doi:10.1038/s41467-026-77051-9)
Supplement: Supplementary file 2 [file 41467_2026_77051_MOESM2_ESM.pdf]

nature portfolio

Corresponding author(s): HanPing Shi, ZhiHao Wu, Li Deng

Last updated by author(s): Apr 26, 2026

Reporting Summary

Nature Portfolio wishes to improve the reproducibility of the work that we publish. This form provides structure for consistency and transparency in reporting. For further information on Nature Portfolio policies, see our Editorial Policies and the Editorial Policy Checklist.

Statistics

For all statistical analyses, confirm that the following items are present in the figure legend, table legend, main text, or Methods section.

n/a	Confirmed	
☐	☒	The exact sample size (*n*) for each experimental group/condition, given as a discrete number and unit of measurement
☐	☒	A statement on whether measurements were taken from distinct samples or whether the same sample was measured repeatedly
☐	☒	The statistical test(s) used AND whether they are one- or two-sided<br>*Only common tests should be described solely by name; describe more complex techniques in the Methods section.*
☐	☒	A description of all covariates tested
☐	☒	A description of any assumptions or corrections, such as tests of normality and adjustment for multiple comparisons
☐	☒	A full description of the statistical parameters including central tendency (e.g. means) or other basic estimates (e.g. regression coefficient) AND variation (e.g. standard deviation) or associated estimates of uncertainty (e.g. confidence intervals)
☐	☒	For null hypothesis testing, the test statistic (e.g. *F*, *t*, *r*) with confidence intervals, effect sizes, degrees of freedom and *P* value noted<br>*Give P values as exact values whenever suitable.*
☒	☐	For Bayesian analysis, information on the choice of priors and Markov chain Monte Carlo settings
☒	☐	For hierarchical and complex designs, identification of the appropriate level for tests and full reporting of outcomes
☒	☐	Estimates of effect sizes (e.g. Cohen's *d*, Pearson's *r*), indicating how they were calculated

Our web collection on statistics for biologists contains articles on many of the points above.

Software and code

Policy information about availability of computer code

Data collection	Western blotting: Amersham Image 600 (v1.2.0)<br>Quantitative PCR: QuantStudio™ Real-Time PCR Software (v1.7.2)<br>Flow cytometry: FACSDiva software (v9.0)<br>Seahorse:Wave (v2.6.1)<br>Immunofluoescence images: NIS-Elements software (v5.20)<br>Immunohistochemical images: NIS-Elements software (v5.20)<br>Multi-Detection microplate reader: BioTek® Gen5 (v2.01.14)<br>LC-MS-based metabolomics: Analyst (vl.6.3), MultiQuant (v3.0.3)<br>RNA-seq data analysis: STAR aligner (v2.7.0e), DESeq2 (v1.30.1), HCS + OLB + GAPipeline-1.6 (Illumina)<br>CUT&Tag: TruePrep Index Kit V2 for Illumina (library prep, Vazyme); data visualization: IGV (v2.15.4)
Data analysis	Image J (v1.8.0)<br>QuantStudio Design &Analysis Software (v1.5.1)<br>Graph Pad Prism (v10.0)<br>FlowJoTM (v10.8.1)

For manuscripts utilizing custom algorithms or software that are central to the research but not yet described in published literature, software must be made available to editors and reviewers. We strongly encourage code deposition in a community repository (e.g. GitHub). See the Nature Portfolio guidelines for submitting code & software for further information.

Data

Policy information about availability of data

All manuscripts must include a data availability statement. This statement should provide the following information, where applicable:

- Accession codes, unique identifiers, or web links for publicly available datasets
- A description of any restrictions on data availability
- For clinical datasets or third party data, please ensure that the statement adheres to our policy

The sequencing datasets generated in this study, including RNA-seq, ChIP-seq and CUT&Tag data, have been deposited in the GSA-Human database under accession codes HRA018092, HRA018096 and HRA018076. The mass spectrometry-based metabolomics raw data generated in this study have been deposited in the OMIX database under accession code OMIX016483. Publicly available transcriptomic comparison data were obtained from GEPIA, which is based on TCGA and GTEx datasets. Source data are provided with this paper.

Research involving human participants, their data, or biological material

Policy information about studies with human participants or human data. See also policy information about sex, gender (identity/presentation), and sexual orientation and race, ethnicity and racism.

Reporting on sex and gender	No gender information on patients with colorectal cancer was provided.
Reporting on race, ethnicity, or other socially relevant groupings	Information on race, ethnicity, or other socially relevant groups of the donors was not provided.
Population characteristics	Patients with colorectal cancer
Recruitment	Patients with colorectal cancer who meet the surgical conditions
Ethics oversight	Approved by the Ethics Committee of Beijing Shijitan Hospital Affiliated to Capital Medical University (Approval number: sjtkyll-lx-2021(39)).

Note that full information on the approval of the study protocol must also be provided in the manuscript.

Field-specific reporting

Please select the one below that is the best fit for your research. If you are not sure, read the appropriate sections before making your selection.

☒ Life sciences ☐ Behavioural & social sciences ☐ Ecological, evolutionary & environmental sciences

For a reference copy of the document with all sections, see nature.com/documents/nr-reporting-summary-flat.pdf

Life sciences study design

All studies must disclose on these points even when the disclosure is negative.

Sample size	The sample size was not pre-determined using statistical methods. For studies in animals, the sample size was determined by our initial experiments.Regarding the organoid data, the sample size was not calculated. In the subsequent period of recruitment, all eligible colorectal cancer patients were allowed to join the study.For in vitro studies, the sample size of each experiment is indicated in the figure caption. Each biological replica is defined as an independent cell culture.
Data exclusions	No data were excluded
Replication	All the experiments were conducted independently at least three times, and the results were similar to the legends.
Randomization	Cells and mice were randomly assigned to the experimental group or the control group.
Blinding	The histopathological assessment of the tumor tissue was independently completed by two pathologists. All other data are based on quantitative analysis of phenotypes and do not require blinding.

Reporting for specific materials, systems and methods

We require information from authors about some types of materials, experimental systems and methods used in many studies. Here, indicate whether each material, system or method listed is relevant to your study. If you are not sure if a list item applies to your research, read the appropriate section before selecting a response.

Materials & experimental systems

n/a	Involved in the study	
☐	☒	Antibodies
☐	☒	Eukaryotic cell lines
☐	☐	Palaeontology and archaeology
☐	☒	Animals and other organisms
☒	☐	Clinical data
☒	☐	Dual use research of concern
☒	☐	Plants

Methods

n/a	Involved in the study	
☐	☒	ChIP-seq
☐	☒	Flow cytometry
☒	☐	MRI-based neuroimaging

Antibodies

Antibodies used

Anti-β-actin ProteinTech HRP-66009 1:10000 for WB
Anti-cJUN ProteinTech 66313-1-Ig 1:1000 for WB
Anti-LDHA ProteinTech 66287-1-Ig 1:1000 for WB
Anti-GPR81 ProteinTech 20146-1-AP 1:1000 for WB
Anti-Ki67 ProteinTech 28074-1-AP 1:1000 for IHC
Anti-Cleaved Caspase 3 ProteinTech 25128-1-AP 1:5000 for IHC
Anti-CD133 Cell Signaling Technology 64326 1:1000 for WB, 1:200 for IHC
Alexa Fluor 555 anti-human CD133 Cell Signaling Technology 4413 1:500 for IF
Anti-p300 Cell Signaling Technology 86377 1:1000 for WB
Anti-CD36 Abcam ab252922 1:1000 for WB
PE anti-human CD133 BioLegend 397904 5 μL per million cells
Anti-Histone H3 (acetyl K18) Abcam ab1191 1:1000 for WB
Acetylated-Lysine (Ac-K) Antibody Cell Signaling Technology 9441 1:1000 for WB
Anti-Histone H3 PTM BIO PTM-3015 1:1000 for WB
Anti-L-Lactyl-Histone H3 (Lys18) PTM BIO PTM-1427RM
Anti-L-Lactyl Lysine PTM BIO PTM-1401RM 1:100 for IF
Anti-L-Lactyl-Histone H3 (Lys18) PTM BIO PTM-1406RM 1:100 for IF

Validation

All the antibodies used in this study were verified according to the manufacturer's instructions.

Eukaryotic cell lines

Policy information about cell lines and Sex and Gender in Research

Cell line source(s)

HCT116, SW620, and HCT15 cell lines, obtained from the American Type Culture Collection (ATCC)

Authentication

All cells have been identified by STR authentication.

Mycoplasma contamination

All the mycoplasma contamination tests of the cell lines were negative.

Commonly misidentified lines (See ICLAC register)

Common misidentification of cell lines was not used in this study.

Palaeontology and Archaeology

Specimen provenance

Provide provenance information for specimens and describe permits that were obtained for the work (including the name of the issuing authority, the date of issue, and any identifying information). Permits should encompass collection and, where applicable, export.

Specimen deposition

Indicate where the specimens have been deposited to permit free access by other researchers.

Dating methods

If new dates are provided, describe how they were obtained (e.g. collection, storage, sample pretreatment and measurement), where they were obtained (i.e. lab name), the calibration program and the protocol for quality assurance OR state that no new dates are provided.

☐ Tick this box to confirm that the raw and calibrated dates are available in the paper or in Supplementary Information.

Ethics oversight

Identify the organization(s) that approved or provided guidance on the study protocol, OR state that no ethical approval or guidance was required and explain why not.

Note that full information on the approval of the study protocol must also be provided in the manuscript.

Animals and other research organisms

Policy information about studies involving animals; ARRIVE guidelines recommended for reporting animal research, and Sex and Gender in Research

Laboratory animals	Female BALB/c nude mice and BALB/c mice (4–6 weeks old) were purchased from Beijing Vitalstar Biotechnology Co., Ltd.
Wild animals	No wild animals were included in this study.
Reporting on sex	All mice were female.
Field-collected samples	There were no field-collected samples used in this study.
Ethics oversight	All animal experiments were approved by the Ethics Committee of Beijing Shijitan Hospital Affiliated to Capital Medical University

Note that full information on the approval of the study protocol must also be provided in the manuscript.

Plants

Seed stocks	*Report on the source of all seed stocks or other plant material used. If applicable, state the seed stock centre and catalogue number. If plant specimens were collected from the field, describe the collection location, date and sampling procedures.*
Novel plant genotypes	*Describe the methods by which all novel plant genotypes were produced. This includes those generated by transgenic approaches, gene editing, chemical/radiation-based mutagenesis and hybridization. For transgenic lines, describe the transformation method, the number of independent lines analyzed and the generation upon which experiments were performed. For gene-edited lines, describe the editor used, the endogenous sequence targeted for editing, the targeting guide RNA sequence (if applicable) and how the editor was applied.*
Authentication	*Describe any authentication procedures for each seed stock used or novel genotype generated. Describe any experiments used to assess the effect of a mutation and, where applicable, how potential secondary effects (e.g. second site T-DNA insertions, mosiacism, off-target gene editing) were examined.*

ChIP-seq

Data deposition

☒ Confirm that both raw and final processed data have been deposited in a public database such as GEO.

☒ Confirm that you have deposited or provided access to graph files (e.g. BED files) for the called peaks.

Data access links<br>*May remain private before publication.*	https://download.cncb.ac.cn/gsa-human/HRA018096
Files in database submission	H1_Input.bam<br>H1_IP.bam<br>H2_Input.bam<br>H2_IP.bam<br>H3_Input.bam<br>H3_IP.bam<br>H4_Input.bam<br>H4_IP.bam
Genome browser session<br>(e.g. UCSC)	No longer applicable

Methodology

Replicates	*Describe the experimental replicates, specifying number, type and replicate agreement.*
Sequencing depth	*Describe the sequencing depth for each experiment, providing the total number of reads, uniquely mapped reads, length of reads and whether they were paired- or single-end.*
Antibodies	*Describe the antibodies used for the ChIP-seq experiments; as applicable, provide supplier name, catalog number, clone name, and lot number.*
Peak calling parameters	*Specify the command line program and parameters used for read mapping and peak calling, including the ChIP, control and index files used.*
Data quality	*Describe the methods used to ensure data quality in full detail, including how many peaks are at FDR 5% and above 5-fold enrichment.*

Software

Describe the software used to collect and analyze the ChIP-seq data. For custom code that has been deposited into a community repository, provide accession details.

Flow Cytometry

Plots

Confirm that:

- [x] The axis labels state the marker and fluorochrome used (e.g. CD4-FITC).
- [x] The axis scales are clearly visible. Include numbers along axes only for bottom left plot of group (a 'group' is an analysis of identical markers).
- [x] All plots are contour plots with outliers or pseudocolor plots.
- [x] A numerical value for number of cells or percentage (with statistics) is provided.

Methodology

Sample preparation	To assess CD133+ cell populations, treated cells were stained with anti-CD133-PE and incubated for 30 minutes at 4°C in the dark. After washing off excess antibody, cells were resuspended in PBS.
Instrument	Flow Cytometry Instruments | BD Biosciences
Software	FlowJoTM (v10.8.1)
Cell population abundance	The abundance of $CD133^+$ and apoptotic cell populations was assessed via flow cytometry. For $CD133^+$ cells, post-treatment populations were stained with anti-CD133-PE and quantified using FlowJo software, with $CD133^+$ percentages calculated relative to total viable cells. For apoptosis assays, cells were dual-stained with Annexin V-FITC and PI, allowing discrimination of live (Annexin V^-/PI^-), early apoptotic (Annexin V^+/PI^-), and late apoptotic/necrotic (Annexin V^+/PI^+) populations. Each condition was tested in at least three biological replicates, and abundance was consistently calculated across experiments.
Gating strategy	Initial gating was performed using FSC-A vs. SSC-A to exclude debris and select viable cell populations, followed by FSC-H vs. FSC-A gating to exclude doublets. For CD133 analysis, PE fluorescence was used to distinguish $CD133^+$ and $CD133^-$ populations, with gates defined based on unstained and isotype control samples. For apoptosis detection, Annexin V-FITC and PI fluorescence channels were gated into four quadrants: live (Q4, Annexin V^-/PI^-), early apoptotic (Q3, Annexin V^+/PI^-), late apoptotic (Q2, Annexin V^+/PI^+), and necrotic/dead (Q1, Annexin V^-/PI^+). Gating was standardized across experiments, and all data were analyzed using FlowJo software (v10.8.1).

- [ ] Tick this box to confirm that a figure exemplifying the gating strategy is provided in the Supplementary Information.
