# Supplementary material for: Glucose-induced histone lactylation confers metformin resistance in colorectal cancer stem cells
Source: Nat Commun. 2026 Sep 7;17:10594. doi: 10.1038/s41467-026-77051-9 (PMC13646297; doi:10.1038/s41467-026-77051-9)
Supplement: Supplementary file 3 [file 41467_2026_77051_MOESM3_ESM.pdf]

nature portfolio

Peer Review File

Glucose-induced histone lactylation confers metformin resistance in colorectal cancer stem cells

Corresponding Author: Professor Han-Ping Shi

This file contains all reviewer reports in order by version, followed by all author rebuttals in order by version.

Version 0:

Reviewer comments:

Reviewer #1

(Remarks to the Author)
In this extremely data-dense manuscript, Xiang-Rui Li and colleagues address the role of glucose concentration in resistance to metformin treatment of colorectal cancer. The fundamental observation on which the manuscript is based demonstrates that metformin inhibits cancer cell/tumor growth and that cells/tumors grown in high glucose concentrations are resistant to this growth inhibition. The authors focus their studies on lipid metabolism and on lactate as integral to the resistance process.

I find the basic story here to be largely convincing (with some caveats outlined below) and the majority of the research here appears to be of high quality. I offer the authors the following comments to help them improve their work.

Major points:
1. Data density. The figure panels in this work are exceedingly information dense – to the point that the data itself is usually very difficult to see. I urge the authors to limit presentation in the main figures to only data absolutely necessary to the points made in the text to permit enlargement of the figures to a size legible to most readers.
2. I find many points in the manuscript where I do not completely agree with the conclusions drawn from the data (outlined below).
3. I find many points in the manuscript where primary data and graphic summary of primary data do not look to me as though they agree (this could be because there is a problem, it could be that I do not understand the presentation)
4. I find many points where callouts in the text do not match figure numbering OR there are typos that I found confusing OR the figure is just confusing to me.
5. I do not agree with one of the primary conclusions of the work – that lactylation of histones at cJUN regulates its expression. Key data for this conclusion are presented in Figure 6. I find Figure 6O to be less than compelling for H3K18la (pan-lactyl is OK). I do not find the global westerns for K18la to be compelling – and this data requires replicates and error bars. I do not agree at all with the conclusion offered in lines 445-446. The key data are in Figures 6Q and 6R. Figure 6Q shows that cJUN levels are essentially unchanged by Met in high glucose conditions and lacylation of K18 at the promoter only occurs with addition of metformin. It is completely unclear to me how this is consistent with K18la 'inducing' cJUN expression as stated in line 445.

Minor points:
1. Places where I do not agree completely with stated conclusions:
a. Lines 194-196: the authors state that 'metformin suppressed the proliferation marker Ki67 and induced the apoptotic marker cleaved caspase-3…'
To my eye this is not true in SW620 (Figure 1 P and Q)
b. Lines 223-225: the authors state that 'this regulation of CD133 expression was not observed for other stemness markers, epithelial – mesenchymal transition (EMT)-related genes, or multidrug (MDR)-associated genes…'
To my eye almost every gene tested S2F does not change.
c. Lines 265-267. I cannot understand the relationship between the heat map in Figure 3D and the validation in Figure S3B and S3C. to my eye, all genes validated have no metformin dependent change in the supplemental data. I am unclear on how the authors reach the conclusion that Met-dependent genes are enriched in LA metabolism
d. Lines 306-307 – lipid droplets correlate with resistance to metformin – there is no demonstration of causality here
2. Places where primary data and graphic summary to not agree to my eye

a. Figure 2L and 2M – Figure 2L the bar graph for Metformin containing tests do not match the primary data (both glucose conditions). Figure 2 M is the same.
b. Primary data (flow cytometry) in 4D looks quite different than the bar graphs, same for 4E
c. Figure 5I – graphs do not match primary data to my eye
3. Places where callouts do not match, there are typos, or its just confusing
a. Primary data are impossible to see – almost all IF figures and many IHC figures. These are just too small
b. Fig 3B – please indicate which cell line is which
c. Line 272 refers to HCT116 and references S3A. Figure S3A is labelled SW620. I believe the callout in text should probably be S4A.
d. Line 276 – same comment. I believe the authors should be referencing Figures S4B and C rather than S3 B and C
e. Possible typo – or I just do not understand what the authors are discussing – in line 292-294. What exactly are LD conditions? Do the authors mean LG conditions? Same for HD. Should this read HG?
f. Typo in line 303 – 4D-3G should likely read 4D-4G?
g. Line 368 – why is Figure S8A cited in text before S7C-F?
h. Figure 6D – what does the comparison of HG-PBS vs HG-PBS mean? Should this be HG_PBS vs HG_Met?
i. I do not understand why cJun does not respond to Met in 6F but does in 6H (high glucose condition)?
j. Figure 6K – are there two amplicons of exactly the same size? Whatever the answer, this assay requires quantitative PCR with replicates and standard statistical analysis.
k. Line 432 – LD conditions? I think this is a typo that should be LG?
l. I believe the text callout in line 477 should be Figure 7Q, not 7P

Reviewer #2

(Remarks to the Author)
The manuscript by Li et al. describes an intereting mechanism of resistance to metformin in colorectal cancer stem cells. Metformin, an anti-diabetic drug known for inhibiting tumor growth in various cancer models, has shown encouraging results in in vitro and preclinical studies, but clinical trials have failed to demonstrate any benefits except some in colorectal cancer. It has been established that metformin targets cancer stem cells more specifically. The authors suggest that their article may explain a potential mechanism behind this resistance to metformin. Through several experimental models—including cell lines, tumoroids, and xenograft injections—they show that metformin induces a well-documented metabolic switch. This switch increases glycolysis and lactate production and alters lipid metabolism. While the mechanistic study is convincing and well-supported by the results, some controls are missing in the in vivo experiments.
Although it is challenging to extrapolate the results of high glucose conditions in vitro to a high carbohydrate diet in real life, the conclusion of this article is that diet may impact the anti-tumoral effects of metformin.
Major Points
Figure 1: The authors convincingly demonstrate that metformin treatment reduces tumor growth in mice on a low carbohydrate diet in a xenograft model, but not in a high carbohydrate regimen. Glucose concentration significantly decreases in metformin-treated mice, while it remains unchanged in those on a low diet. This suggests that metformin increases glucose consumption by tumor cells only under high carbohydrate conditions.
A major drawback of the vivo experiments is that they were performed on immunodeficient mice. Do the authors observe the same results in immunocompetent animals? Immune cells may play a role in controlling glucose homeostasis. It would be interesting to validate these results in a syngeneic model.
Figure 2: Would it be possible to express CD133 under low glucose conditions to establish a direct link between apoptosis and CD133 expression and reverse the observed effects?
Figure 3: Is there a clear connection between linoleic acid metabolism and CD36? Wouls that be possible to supplement the cells in Linoleic acid? What is the level of CD36 overexpression in CD133+ cells? The authors should compare this to the expression in cells treated with high glucose and metformin.
Figure 4: It is clear that metformin increases lipid droplet formation, but did the authors check triglyceride levels in the cells? Since metformin has been shown to inhibit lipogenesis in cancer cells, did the authors monitor lipid synthesis in their model? Given that fatty acid oxidation (FAO) is implicated in ATP production and ATP levels decrease following metformin treatment, did the authors analyze AMPK phosphorylation in the different models? Lipid accumulation should be monitored in vivo to validate the efficiency of the different inhibitors (Figure 4N).
Figure 5: The authors demonstrate that metformin increases glycolytic flux in cancer cells, resulting in increased lactate production. The authors should measure lactate concentrations in tumor interstitial fluids to confirm this effect in vivo.
Figure 6: Since lactate regulates CD36 expression through the transcription factor c-Jun, it would be interesting to determine if lactate also affects the phosphorylation of c-Jun. There are well-characterized JNK inhibitors; I suggest studying their effects in vitro and in vivo.
Figure 7: Is there a correlation between LDHA expression and overall survival or progression-free survival in colorectal cancer? The authors should analyze several databases to reinforce their conclusion. Is there a correlation between LDHA expression and the response to anti-cancer treatment in clinical settings for colorectal cancer? The characterization of lactate levels, as well as CD36 and c-JUN expression, is missing in the in vivo experiments.
Minor Points
• Typo: Line 222 contains a repetition of the word "proportion."
• Typo: Line 291 should correctly state "Lipid Droplets (LD) and low glucose (LG)."

Reviewer #3

(Remarks to the Author)
Li et al describe the acquisition of metformin-resistance in colorectal cancer due to lactate accumulation as a function of a high-glucose/carbohydrate diet. This increase in lactate is proposed to lead to histone lactylation of the c-Jun promoter, inducing c-Jun expression which in turn drives CD36 expression. CD36 mediates the uptake of fatty acids and lipid droplet formation. The authors propose that these stored lipids improve the survival of cancer stem cells and patient-derived organoids to metformin treatment by mitigating oxidative (ROS) and energy stress (through FA oxidation for ATP/NADPH production) as a means of chemoresistance.
While most experiments are well controlled and show clear effects, I have major concerns that limit my enthusiasm for the suggested model.
Major Concerns:
1. The authors propose that LDHA-driven metformin-resistance is associated with increased FA uptake and storage. However, it is unclear if this is due to the FAs being utilized for energy or if the FAs are stored (lipid droplets) to act as a buffer for ROS (eg. PMIDs: 25594180, 30400205)
a. What is the main mechanism (storage or utilization of FAs) of LDHA driven metformin-resistance associated with this phenotype?
b. Does FA oxidation increase in the metformin-resistance condition?
c. If this is associated with FA oxidation for ATP/NADPH production, how does this occur under conditions of ETC impairment (metformin)?
d. Can the LDHA-high phenotype (Figure 7) be reversed by blocking FA uptake (CD36i – SMS121) or FA oxidation (Etomoxir)? Or can the LDHA-low phenotype be reversed by reducing ROS, e.g. N-acetyl-cysteine?
2. Lactate plays an important role in maintaining the NAD+/NADH ratio under reduced ETC function. Have the authors assessed whether it is the maintenance of this redox balance that is driving metformin-resistance? Can alpha-ketobutyrate/LbNox also induce metformin resistance?
3. Is lactylation the main driver of the c-Jun/CD36 expression? The phenotypes could be equally explained by the expression of c-Jun being driven by increased histone acetylation or even other non-canonical acylations. Ascribing the effects to histone lactylation specifically seems an overreach without mechanistic experiments showing that lactylation is both necessary and sufficient for the c-Jun expression changes.
4. It is not entirely clear that the high lactate phenotypes are driven by lactylation rather than by oxidation of lactate. The authors could assess the contributions of glucose derived-lactate/acetyl-CoA to c-Jun expression through glucose fate tracing experiments in the LG/HG conditions. In addition, if the phenotypes are mediated by histone lactylation, inhibiting the entry of lactate and/or lactate-derived pyruvate into the mitochondria should have little effect; in fact, they may even phenocopy metformin resistance. The authors should show this with inhibition or knock-out of the mitochondrial pyruvate carrier, for examplet.

Minor comments:
1. Fig 1F,G,Q– the HD effect seems limited to HCT116, not really seen in SW620 (even in HD conditions: tumor size decreases, cleaved casp3 increases, Ki67 decreases). Authors should provide explanation or show models that phenocopy the effects seen in HCT116.
2. Figure 1P-S,S1: why does Ki67 (marker of proliferation) reduce/not change, but CD133 increases – are these CD133+ cells still proliferating normally? Is the CD133+ population due to de-differentiating to CSC fate, or increased proliferation of CD133+ cells?
3. Lines 229-230. Figure 2I, J: Why does PDO size decrease but number of CD133+ cells increase? How rapidly do these cells proliferate? Is there a difference in cell volume that contributes to reduced PDO size?
4. Paragraph 231-236; Figure S2G – do the authors sort CD133+ cells to assess colony forming capacity?
5. Lines 236-238: Magnetic sorting for CD133+ – clarify if metformin treatment is after sorting – how long are the cells treated here?
6. Line 240-241, Figure 2: In SW620 the CD133+ cells, AnnexinV increases in both HG and LG condition but not in CD133- as stated (cytometry plots and panels for HCT15 showed correct observations) – perhaps quantification panels are swapped in Figure 2L/M?
7. Lines 253-257, Fig 3G-K: do the authors experimentally confirm 24h is not sufficient for AnnexinV to increase?
8. Lines 347-349 – higher ECAR (Figure 5A,B) should imply media acidification and higher lactate levels (same time point for lactate assay + Seahorse ECAR i.e. 24 hours post metformin treatment). Line 352-353 – is the data normalised to cell number or content to compensate for these differences?
9. Line 395, Figure S9 – this experimental set up (mRNA + protein stability) does not look at the effect of lactate but rather metformin.

Typos/Errors:
1. Figure 2 L, M – incorrect panel quantification (see minor comment #6)
2. Line 272 – Fig S4A?
3. Line 276 – typos: (CD6)
4. Line 276- Fig S4B, C?
5. Line 292; use different acronyms; LD=low carb diet + LD=lipid droplets?
6. Fig4 L, M: labels wrong – no LG condition but shown in quantification.
7. Fig5 I, J: Missing label and information for metformin in quantification panels
8. Fig 6D – labels wrong?
9. Fig 6N – missing a + sign?

Version 1:

Reviewer comments:

Reviewer #2

(Remarks to the Author)
The authors have satisfactorily answered my questions, so I give a favorable opinion for the publication of this article.

Reviewer #3

(Remarks to the Author)
In the revised manuscript, the authors have addressed many of the prior concerns. However, my primary concern remains. I do not think the authors have ruled out that lactylation is simply a passenger phenomenon mediated by the high levels of lactate in the high glucose and high glucose metformin conditions. That is, c-JUN activation and CD36 upregulation could be mediated through canonical p300-dependent histone acetylation and histone-lactylation could be simply correlated because of its high levels.

Major comments:
1. In S17 and S18, the authors show that 'Pharmacologic inhibition of fatty-acid uptake with SMS121 or blockade of FAO with etomoxir partially reversed metformin resistance and increased apoptosis, indicating that LDHA-driven resistance relies on FAO-mediated metabolic compensation. Given that the title of the paper calls on histone lactylation and a c-JUN/CD36 axis, the authors need to show that these effects (fatty acid update and fatty acid oxidation) occur downstream of lactylation-induced, c-JUN mediated CD36 expression.
2. If fatty acid oxidation is enabling ATP and NADPH synthesis in high-glucose/metformin conditions, shouldn't the size of the lipid droplets or TG levels? However, the authors show no change in lipid content (LD size or TG levels) in the Metformin treated groups in HG.
3. The authors claim that cJUN, phospho cJUN and CD36 are maximally activated (saturated) in high glucose conditions (Figure 6A-B) and thus metformin cannot further increase cJUN activity and CD36 expression. Nonetheless, since the purported mechanism for resistance is increased lactate leading to cJUN lactylation, why do the authors not see high levels of lactylation at the cJUN promoter (Figure 6Q)? This seems contrary to lactate being the mediator of resistance and something else leading to increased cJUN activation and CD36 expression.
4. Given that HG+PBS and HG+Met have similar levels of TG, LD size, and FAO, how do the authors link this with their findings that lactate (higher in HG+Met, Figure 6Q, 5F) drives the FA uptake via cJUN/CD36 to mediate Met resistance?
5. Lines 488-492, the authors claim that 'Together, these results demonstrate that lactate-induced metformin resistance occurs independently of lactate oxidation and NAD^+/NADH homeostasis, and instead primarily relies on lactate-driven epigenetic regulation-specifically, H3K18 lactylation, that promotes c-JUN transcription and consequently upregulates CD36 expression.' This is not concluded by the data. The data only shows that histone lactylation is happening at these sites in a manner that correlates with high glucose and lactate (with or without metformin). No causative value can be ascribed to the lactylation based on these results.

Minor comments:
1. In Figure 2E, in the text the authors claim "In HCT116-identified by flow as CD133+-dominant-metformin under HG further enriched the highly CD133-expressing subset". However, looking at the figure, the cutoff for CD133 high seems arbitrary and the main effect of metformin seems to be that in LG the proportion of CD133- increases markedly. This is not seen in HG for the positive fraction though is still significantly elevated in the high though this increase is diminished.
2. For Figures 2I-K and S2I-J, the percentages in the bar graphs do not add up to the percentages in the scatter plot. What is being plotted?
3. For Supplementary Figure 4A and 4B, the authors should confirm that the Ki67 staining is specific. It is unusual for Ki67 to not be entirely localized to the nucleus.
4. For Figure 3B, I would suggest using the same color scheme here; the current color choices for CD133+ and CD133- cells are confusing.
5. Line 291 CD36 should be CD36OE
6. The statement, "Importantly, metabolomics was performed 24h after metformin when CD133+ CSC viability under LG had not yet declined significantly (Fig. 2J), arguing against non-specific changes due to cell death." is misleading as cells could be on their way to cell death due to LG dependent changes but not yet sufficiently stressed to trigger Annexin V positivity.
7. Line 352-353: In vivo data does not show FAO in S8K, only LD size.
8. Figure 4B. If CD36 upregulates linoleic acid production, why do the authors add LA to Figure 4B and 4C? What do these results look like without LA?
9. Figure 5AB. What do the authors mean by basal and compensatory glycolytic rates? These do not seem to be defined in the text.
10. Line 411 LDHA should be siLDHA.
11. As recommended, the authors have probed possible effects of lactate through NAD regeneration or oxidation in the mitochondria. The alpha-ketobutyrate experiments are conclusive, with aKB restoring NAD (SFig15A) and having no effect on apoptosis or viability. However, when the authors probe whether lactate can still have effects when prevented from entering mitochondria through MPC, there is no control for whether at the doses used, UK5099 had any effect. Thus, the

authors cannot conclude that the effects of lactate are not mediated by its oxidation.
12. In Figure 6N, CD36 expression is high in most HG conditions. Given that the HG, metformin treatment is still high in siLDHA-1 treated cells (though LDH levels are not shown), this suggests that the regulation of CD36 is not dependent on lactate levels.
13. In Figure 6N-Q, do the levels of histone acetylation mirror those of lactylation and do the lactylated areas (by CUT&RUN) also show with an acetylation antibody?Figure 6D – are the labels still incorrect?
14. Lines 315-317: "Blocking LD biogenesis markedly delayed or prevented resistance, whereas inhibitor withdrawal partially restored resistance with renewed lipid accumulation, indicating that LDs are critical for Met resistance in CD133+ CRC CSCs". This is a premature conclusion. At most, the authors can claim that DGAT1i is necessary and sufficient for lipid droplet formation and viability, but the role of lipid droplets is entirely unanswered by this data.

Version 2:

Reviewer comments:

Reviewer #3

(Remarks to the Author)
The authors have addressed most of my comments and concerns.

The experiments showing that acetate supplementation fails to rescue are an important addition. However, what is the dose of acetate used in the in vitro experiments? In vivo, the administration route and dose is very different (200 mM in drinking water for NaAc versus 1.68 g/kg, i.p., q2d NaLac). At least for the in vitro experiments, these doses should be equivalent and/or the authors should show that the acetate supplementation provided is sufficient to increase general histone acetylation but not to rescue the phenotype that is rescue by lactate.

Version 3:

Reviewer comments:

Reviewer #3

(Remarks to the Author)
The authors have addressed my remaining concerns.

Open Access This Peer Review File is licensed under a Creative Commons Attribution 4.0 International License, which permits use, sharing, adaptation, distribution and reproduction in any medium or format, as long as you give appropriate credit to the original author(s) and the source, provide a link to the Creative Commons license, and indicate if changes were

made.
In cases where reviewers are anonymous, credit should be given to 'Anonymous Referee' and the source.
The images or other third party material in this Peer Review File are included in the article’s Creative Commons license, unless indicated otherwise in a credit line to the material. If material is not included in the article’s Creative Commons license and your intended use is not permitted by statutory regulation or exceeds the permitted use, you will need to obtain permission directly from the copyright holder.
To view a copy of this license, visit https://creativecommons.org/licenses/by/4.0/

Dear editors and reviewers,

Warm greetings of the day and hope this finds you well.

Thank you for your constructive and insightful advice about the manuscript. Those comments are all valuable and very helpful for revising and improving our paper. We have studied your comments carefully and made corrections accordingly. The point-by-point responses are summarized below. All modifications have been shown in **red and bold font** in the manuscript.

Sincerely,

Han Ping Shi, MD, PhD, FACS

Chairman

Dept. of GI Surgery

Dept. of Clinical Nutrition

Beijing Shijitan Hospital

Capital Medical University

<Comments from Reviewer #1>

Major Points:

Comment 1: Data density. The figure panels in this work are exceedingly information dense – to the point that the data itself is usually very difficult to see. I urge the authors to limit presentation in the main figures to only data absolutely necessary to the points made in the text to permit enlargement of the figures to a size legible to most readers.

Response: We thank the reviewer for the constructive comments on figure presentation. We agree that some figures were overly information-dense, which may have affected readability. We have made every effort to reduce the density of the main figures by relocating certain non-essential results to the Supplementary Figures. We adjusted the sizes of the immunohistochemistry (IHC) and immunofluorescence (IF) images to improve visibility.

Comment 2-4: 2. I find many points in the manuscript where I do not completely agree with the conclusions drawn from the data (outlined below).

3. I find many points in the manuscript where primary data and graphic summary of primary data do not look to me as though they agree (this could be because there is a problem, it could be that I do not understand the presentation).

4. I find many points where callouts in the text do not match figure numbering OR there are typos that I found confusing OR the figure is just confusing to me.

Response: We thank the reviewer for the careful evaluation of our manuscript. We acknowledge that the initial submission contained some inconsistencies between data and conclusions, discrepancies between primary data and graphical summaries, and errors in figure callouts. We regret these oversights and apologize for the confusion caused. In the revised manuscript, we have corrected these issues and clarified the presentation. Detailed revisions and point-by-point explanations are provided below.

Comment 5: I do not agree with one of the primary conclusions of the work – that lactylation of histones at cJUN regulates its expression. Key data for this conclusion are presented in Figure 6. I find Figure 6O to be less than compelling for H3K18la (pan-lactyl is OK). I do not find the global westerns for K18la to be compelling and

this data requires replicates and error bars. I do not agree at all with the conclusion offered in lines 445-446. The key data are in Figures 6Q and 6R. Figure 6Q shows that cJUN levels are essentially unchanged by Met in HG conditions and lacylation of K18 at the promoter only occurs with addition of metformin. It is completely unclear to me how this is consistent with K18la 'inducing'c-JUN expression as stated in line 445.

Response: We sincerely thank the reviewer for the rigorous evaluation of this central conclusion. We acknowledge that in the initial submission, the available data may not have been sufficient to fully support the statement that "H3K18 lactylation at the c-JUN promoter directly induces c-JUN expression." In the revised manuscript, we have performed additional experiments and provided more detailed quantitative results to address the reviewer's concerns and to strengthen the persuasiveness of our conclusions. Below we provide point-to-point responses:

(a) Figure 6O (H3K18la signal not compelling)

To address this concern, we performed additional validation using Western blot analysis. Specifically, we employed two independent antibodies against H3K18la (Anti-L-Lactyl-Histone H3 [Lys18] Rabbit, PTM BIO, PTM-1427RM and PTM-1406RM) to ensure both the specificity and reproducibility of the signal. We have added this sentence in the Results at **Line 462**, and the supporting data are provided in Supplementary **Fig. S14B**.

(b) Global western blots for H3K18la (lack of replicates and error bars)

We have now repeated the experiments in three independent replicates (see below) and included quantification with error bars in the revised Supplementary **Fig. S14C**.

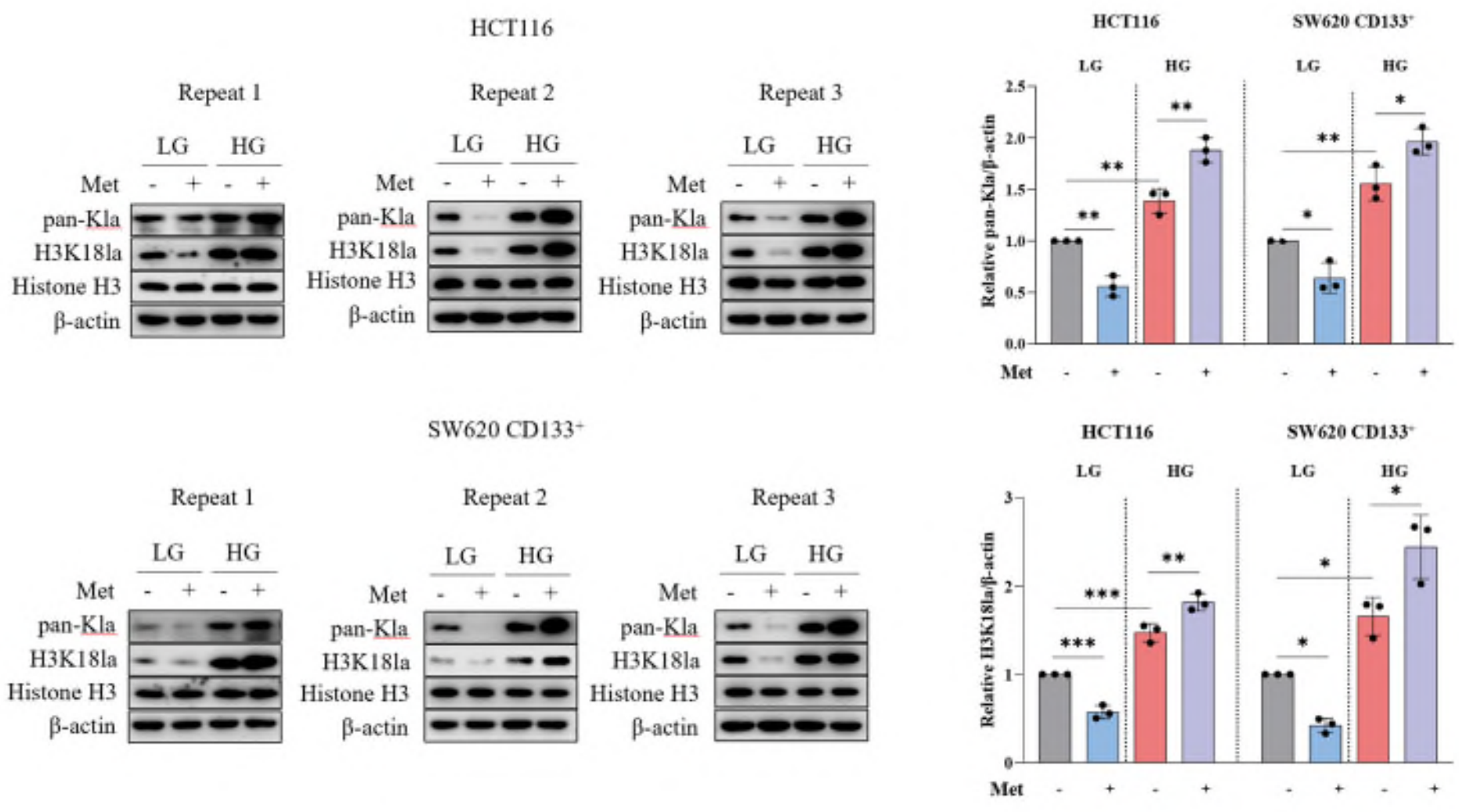

(c) Line 445–446 (claim that "K18la induces c-JUN expression")

We thank the reviewer for this important comment. We believe that the lack of a further increase in c-JUN expression upon metformin treatment under HG conditions is primarily due to a saturation effect of lactate. Our additional experimental results show that as the concentration of metformin increases, lactate levels rise in a dose-dependent manner, accompanied by a corresponding increase in c-JUN expression. However, once lactate levels exceeded a certain threshold (approximately 5.06 mM in HCT116 cells and 4.56 mM in SW620 CD133^{+} cells), c-JUN expression reached a plateau and no longer increased. To further validate this phenomenon, we supplemented the culture medium with exogenous lactate and again observed that c-JUN expression did not increase beyond the same threshold. These results indicate that while lactate can indeed induce c-JUN expression, its effect is saturable. We have added this sentence in the Results at **Line 471-477**, and the supporting data are provided in **Supplementary Fig. S14D**. In addition, as shown in Figure 6G, knockdown of LDHA by shRNA reduced lactate production and markedly decreased c-JUN expression, whereas in Figure 6N, supplementation with exogenous lactate under LG conditions promoted c-JUN expression. Taken together, these results further support the role of lactate in regulating c-JUN expression, while also demonstrating its saturable effect, which provides a mechanistic explanation for why metformin treatment under HG conditions did not further enhance c-JUN expression.

Minor points:

Comment 1a: Lines 194-196: the authors state that 'metformin suppressed the proliferation marker Ki67 and induced the apoptotic marker cleaved caspase-3…' To my eye this is not true in SW620 (Figure 1 P and Q) .

Response: We believe that this confusion may have arisen from the wording in our original text, which did not clearly distinguish between the HCT116 and SW620 models. As shown in Figure 1M, in SW620 xenografts metformin significantly reduced Ki67 expression and increased Cleaved Caspase-3 expression under both LCD and HCD conditions. However, it is important to note that compared with LCD, the pro-apoptotic and anti-proliferative effects of metformin were weaker under HCD conditions, and this difference was statistically significant. In contrast, in HCT116 xenografts, the effects of metformin were prominent under LCD conditions but were markedly attenuated under HCD conditions. We have revised the Results at **Line 192-197** accordingly to more accurately describe the differences between HCT116 and SW620.

Comment 1b: Lines 223-225: the authors state that 'this regulation of CD133 expression was not observed for other stemness markers, epithelial–mesenchymal transition (EMT)-related genes, or multidrug (MDR)-associated genes…' To my eye almost every gene tested S2F does not change.

Response: We agree that our original wording may have been ambiguous. Our intention was to convey that among the various stemness markers, EMT-related genes, and MDR-associated genes examined, CD133 was the only marker that consistently exhibited a glucose-dependent regulation by metformin across all three cell lines — being downregulated under LG conditions and upregulated under HG conditions. In contrast, while some of the other genes showed changes under certain conditions or in specific cell lines, these alterations were variable and did not display the same consistent pattern as CD133. We have revised the text in the Results section at **Line 225-230** to more clearly reflect this point.

Comment 1c: Lines 265-267. I cannot understand the relationship between the heat map in Figure 3D and the validation in Figure S3B and S3C. to my eye, all genes validated have no metformin dependent change in the supplemental data. I am unclear on how the authors reach the conclusion that Met-dependent genes are enriched in LA metabolism.

Response: The issue here primarily arose from a lack of clarity in our original wording. Our research strategy was as follows: first, metabolomic analysis revealed that in $CD133^{+}$ CSCs treated with metformin under LG conditions, significantly altered metabolites were enriched in multiple pathways, among which linoleic acid (LA) metabolism was the most prominent. Based on this finding, we integrated RNA-seq data to examine key genes within the LA metabolism pathway and further validated them in $CD133^{+}$ CSCs. It is important to emphasize that our conclusion was not that all genes in this pathway were significantly altered. Rather, through the combination of multi-omics analysis and RT-PCR validation, we found that most genes showed limited changes, whereas CD36 consistently exhibited stable and significant alterations in both cell models, and was therefore identified as a key candidate gene. We have revised the relevant text in the manuscript at **Line 273-281** to avoid ambiguity and to more accurately convey our research rationale.

Comment 1d: Lines 306-307 – lipid droplets correlate with resistance to metformin – there is no demonstration of causality here.

Response: We agree with the reviewer that our initial results alone were not sufficient to fully establish causality. In Figure 4, we had already observed that inhibition of lipid droplet formation under HG conditions significantly reduced the resistance of $CD133^{+}$ CSCs to metformin, suggesting an important role for lipid droplets in this process. In the course of revision, we conducted additional experiments to further validate this relationship. Specifically, when lipid droplet inhibitors were added during the establishment of resistant cell populations, the development of the resistant phenotype was markedly delayed or even prevented; upon withdrawal of the inhibitors, resistance partially reappeared, accompanied by an increase in intracellular lipid droplet levels.

Moreover, quantitative lipid droplet staining further confirmed a direct correlation between the extent of lipid droplet formation and the degree of resistance. These results provide stronger evidence for a causal role of lipid droplets in metformin resistance. We have incorporated these new data into **Supplementary Fig. S7C–S7D** and revised the text at **Lines 313–316** to more accurately reflect our findings.

Comment 2: a. Figure 2L and 2M – Figure 2L the bar graph for Metformin containing tests do not match the primary data (both glucose conditions). Figure 2 M is the same.

b. Primary data (flow cytometry) in 4D looks quite different than the bar graphs, same for 4E

c. Figure 5I – graphs do not match primary data to my eye.

Response: We sincerely thank the reviewer for the careful identification of these issues. Upon re-examination, we confirmed that the inconsistencies arose from figure-preparation errors in the initial submission: the bar graphs for Fig. 2L and 2M (now **Fig. 2J and 2K**) and for Fig. 4D and 4E were inadvertently swapped, and the bar graph in Fig. 5I was not properly matched to the corresponding flow-cytometry data. In the revised manuscript, we have thoroughly rechecked the raw data, corrected these errors, and redrawn the relevant bar graphs to ensure full consistency with the representative primary data. We apologize for these oversights and again thank the reviewer for the helpful feedback.

Comment 3a: Primary data are impossible to see – almost all IF figures and many IHC figures. These are just too small.

Response: We thank the reviewer for pointing this out. In the revised manuscript, we have enlarged all IF and IHC panels and replaced them with higher-resolution images, thereby ensuring that the primary data are now clearly visible.

Comment 3b. Fig 3B – please indicate which cell line is which

Response: We have now explicitly indicated the cell line in Figure 3B as SW620.

Comment 3c. Line 272 refers to HCT116 and references S3A. Figure S3A is labelled SW620. I believe the callout in text should probably be S4A.

Response: This was an error in the original manuscript. In the prior version, the correct callout should indeed have been Fig. S4A. In the revised manuscript, after figure renumbering, the corresponding panel is now **Fig. S6A**. We have updated the text at **Line 284** accordingly.

Comment 3d. Line 276 – same comment. I believe the authors should be referencing Figures S4B and C rather than S3 B and C

Response: We agree. In the prior version, the correct callouts should indeed have been Figs. S4B, S4C. In the revised manuscript, after figure renumbering, the corresponding panels are now Figs. S6D, S6E. We have updated the text at **Line 291** accordingly.

Comment 3e. Possible typo – or I just do not understand what the authors are discussing – in line 292-294. What exactly are LD conditions? Do the authors mean LG conditions? Same for HD. Should this read HG?

Response: To avoid confusion, we have standardized abbreviations throughout the manuscript: LG/HG denote low- and high-glucose cell-culture conditions; LCD/HCD denote low- and high-carbohydrate diet conditions in animal studies; and LD refers to lipid droplet. The corresponding text at Line **303-305** has been revised, and the main text, figure labels, and legends have been updated consistently across the manuscript.

Comment 3f. Typo in line 303 – 4D-3G should likely read 4D-4G?

Response: Corrected as suggested to “4D–4G” at **Line 312.**

Comment 3g. Line 368 – why is Figure S8A cited in text before S7C-F?

Response: We thank the reviewer for the helpful comment. Because Fig. S10A and Fig. S10B originate from the same experimental set, we kept LDHA expression as Supplementary Fig. S10A so that it serves as the shared baseline control for the subsequent GPR81/medium acidification analyses (Supplementary Fig. S10B). To avoid confusion, we have moved the first in-text citation of Fig. S10A to follow Fig. S9D–S9G. These changes, implemented at **Lines 392–394**, were made solely to improve organization and narrative flow and do not affect any data or conclusions.

Comment 3h. Figure 6D – what does the comparison of HG-PBS vs HG-PBS mean? Should this be HG_PBS vs HG_Met?

Response: This was a labeling mistake. It has been corrected to "HG-PBS vs HG-Met".

Comment 3i. I do not understand why cJun does not respond to Met in 6F but does in 6H (high glucose condition)?

Response: We thank the reviewer for raising this point. As discussed in our response to Major Point 5, lactate-mediated induction of c-JUN has a saturable effect. Thus, under HG conditions, c-JUN should not further respond to Met. To clarify, we re-prepared samples and repeated the Western blot experiments, which confirmed that c-JUN expression indeed shows no significant change under HG ± Met in Figure 6H. The revised panel now reflects this result.

Comment 3j. Figure 6K – are there two amplicons of exactly the same size? Whatever the answer, this assay requires quantitative PCR with replicates and standard statistical analysis.

Response: Figure 6K shows PCR products corresponding to two c-JUN-binding regions in the CD36 promoter (−325 to −312 and −152 to −139). Because the amplicons are of similar size, the bands appear identical on agarose gels. To strengthen the evidence, we now provide ChIP-qPCR quantification with three biological replicates (mean ± SD, with statistical testing) in the revised manuscript. The new quantification

is included in **Fig. 6K**, and the corresponding description has been added to the Results (c-JUN–CD36/ChIP subsection) at **Lines 450–451** of the revised text.

Comment 3k. Line 432 – LD conditions? I think this is a typo that should be LG?

Response: Corrected to LCD conditions (dietary context) in **Line 461**, consistent with our abbreviation policy.

Comment 3l. I believe the text callout in line 477 should be Figure 7Q, not 7P

Response: We agree and have corrected the callout to Figure 7Q in **Line 543**.

<Comments from Reviewer #2>

Major Points:

Comment 1：Figure 1: The authors convincingly demonstrate that metformin treatment reduces tumor growth in mice on a low carbohydrate diet in a xenograft model, but not in a high carbohydrate regimen. Glucose concentration significantly decreases in metformin-treated mice, while it remains unchanged in those on a low diet. This suggests that metformin increases glucose consumption by tumor cells only under high carbohydrate conditions.

A major drawback of the vivo experiments is that they were performed on immunodeficient mice. Do the authors observe the same results in immunocompetent animals? Immune cells may play a role in controlling glucose homeostasis. It would be interesting to validate these results in a syngeneic model.

Response: We sincerely thank the reviewer for this valuable comment. We fully agree that immune cells may influence systemic glucose homeostasis and thereby affect the antitumor activity of metformin. In this study, we employed immunodeficient mice because xenografts derived from human colorectal cancer stem cells must be established in immunodeficient hosts, which is the standard practice in the field. In addition, we have performed validation experiments in immunocompetent mice. Specifically, we established a CT26 syngeneic tumor model in BALB/c mice and treated the animals with metformin under different dietary conditions. The results showed that a high-carbohydrate diet similarly attenuated the antitumor effect of metformin and was accompanied by an increased proportion of $CD133^{+}$ tumor cells, consistent with the trend observed in immunodeficient mice **(Fig. S1E-S1L)**. We have incorporated these data and corresponding descriptions into the revised Results section in **Line 198-202** to further emphasize the consistency of our findings in an immunocompetent model.

Comment 2：Figure 2: Would it be possible to express CD133 under low glucose conditions to establish a direct link between apoptosis and CD133 expression and reverse the observed effects?

Response: We thank the reviewer for this constructive suggestion. We performed ectopic overexpression of CD133 under low-glucose conditions and confirmed effective overexpression by protein and flow cytometry analysis. However, CD133 overexpression did not increase cellular resistance to metformin compared with controls. This result suggests that CD133 primarily serves as a marker of the CSC state rather than being sufficient to drive resistance on its own **(See the Figure below)**.

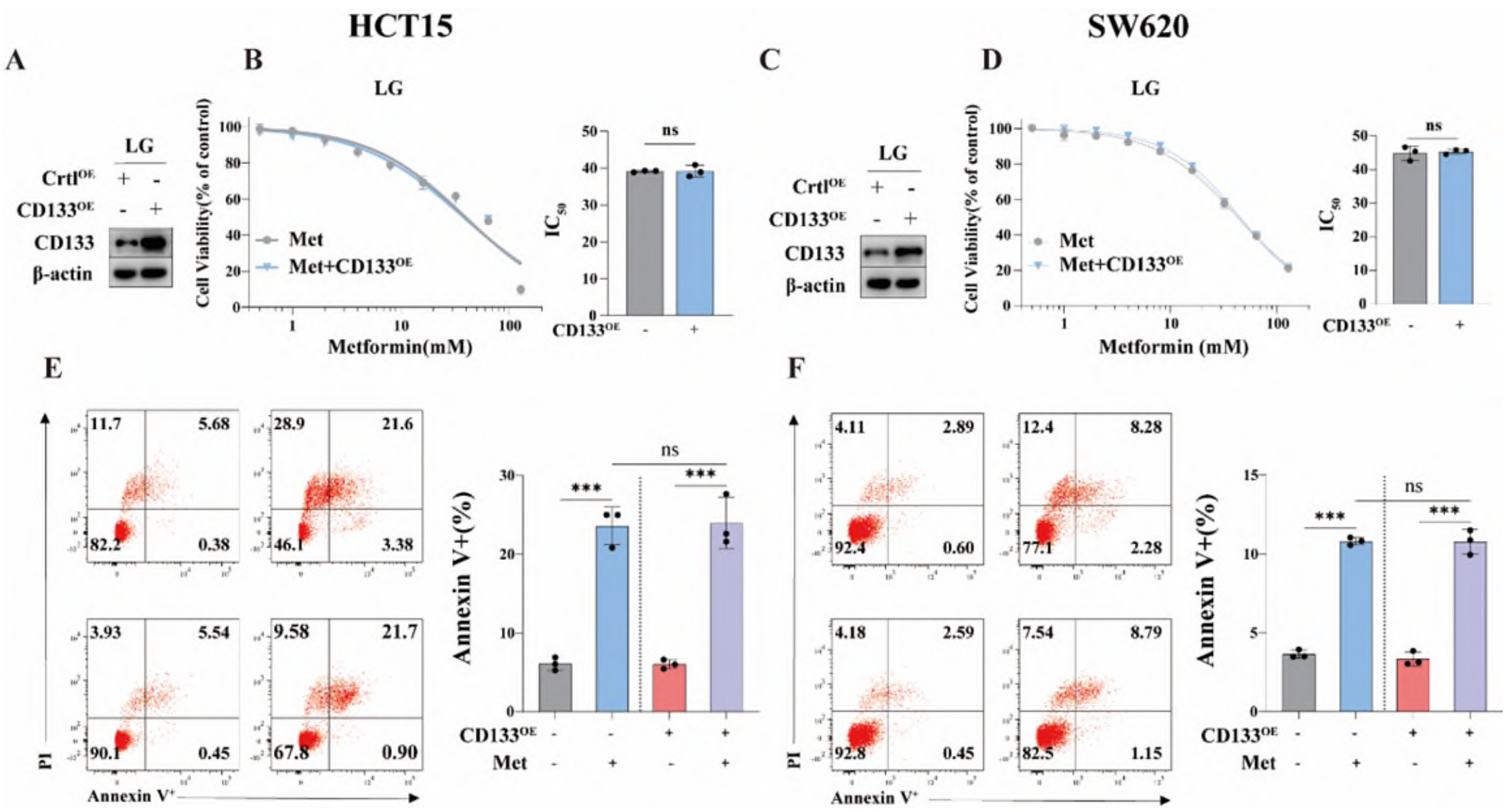

Comment 3：Figure 3: Is there a clear connection between linoleic acid metabolism and CD36? Wouls that be possible to supplement the cells in Linoleic acid? What is the level of CD36 overexpression in CD133+ cells? The authors should compare this to the expression in cells treated with high glucose and metformin.

Response： (1) CD36 is a broadly expressed long-chain fatty acid transporter that mediates the uptake of unsaturated fatty acids, including linoleic acid (LA). Previous studies have shown that LA upregulates CD36 expression in immune cells via a PPAR-dependent mechanism, thereby promoting lipid uptake [1-2]. However, to date, no direct evidence has demonstrated a causal link between LA metabolism and CD36 expression in tumor cells.

(2) In our study, we supplemented $CD133^{+}$ cells with varying concentrations of LA, but did not observe any further increase in CD36 expression. This suggests that LA

regulation of CD36 may be cell type–dependent (**See the Figure A, B below**).

(3) Furthermore, in HCT15 and SW620 cells, we compared the expression levels of CD36 between $CD133^{-}$ and $CD133^{+}$ subpopulations under basal and treatment conditions. At baseline, CD36 expression was comparable between the two subsets. However, upon exposure to high-glucose and metformin treatment, a marked induction of CD36 was observed exclusively in the $CD133^{+}$ population, whereas $CD133^{-}$ cells exhibited minimal alteration. These findings suggest that CD36 upregulation in response to metabolic stress is predominantly confined to the CSC-enriched fraction **(Fig.S6B, S6C)**. We have incorporated the corresponding results into the revised Results section in **Line 284-288**.

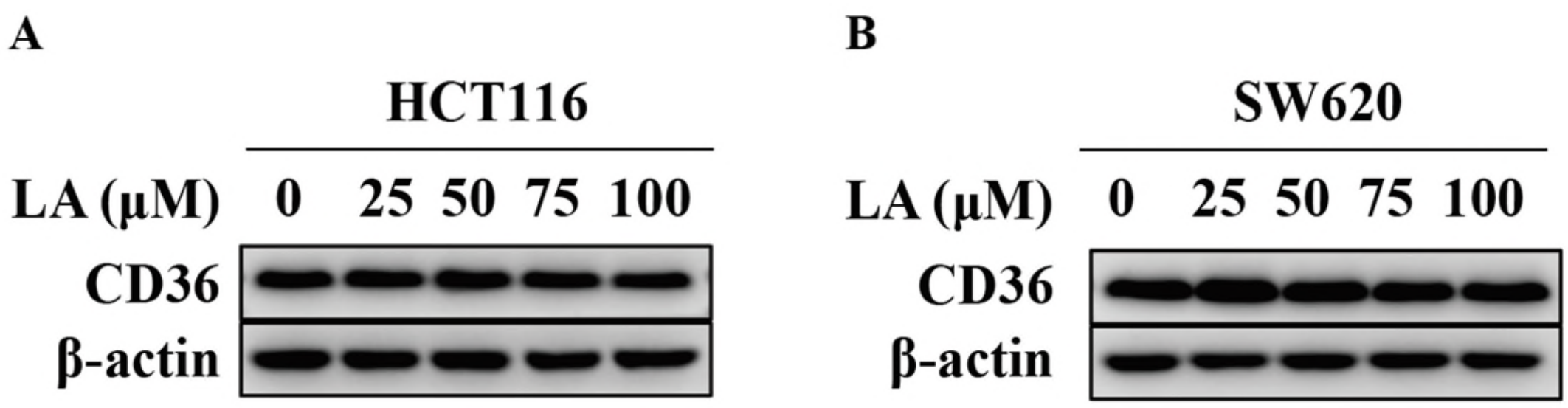

1. Vallvé JC, Uliaque K, Girona J, Cabré A, Ribalta J, Heras M, Masana L. Unsaturated fatty acids and their oxidation products stimulate CD36 gene expression in human macrophages. Atherosclerosis. 2002 Sep;164(1):45-56. doi: 10.1016/s0021-9150(02)00046-1IF: 5.7 Q1. PMID: 12119192.

2. Weldon S, Mitchell S, Kelleher D, Gibney MJ, Roche HM. Conjugated linoleic acid and atherosclerosis: no effect on molecular markers of cholesterol homeostasis in THP-1 macrophages. Atherosclerosis. 2004 Jun;174(2):261-73. doi: 10.1016/j.atherosclerosis.2004.02.007. PMID: 15136056.

Comment 4：Figure 4: It is clear that metformin increases lipid droplet formation, but did the authors check triglyceride levels in the cells? Since metformin has been shown to inhibit lipogenesis in cancer cells, did the authors monitor lipid synthesis in their model? Given that fatty acid oxidation (FAO) is implicated in ATP production and ATP levels decrease following metformin treatment, did the authors analyze

AMPK phosphorylation in the different models? Lipid accumulation should be monitored in vivo to validate the efficiency of the different inhibitors (Figure 4N).

Response： We sincerely thank the reviewer for the insightful comments. To address the concerns regarding lipid droplet formation, triglyceride (TG) content, lipogenesis, and energy-sensing pathways, we performed additional experiments, and the results are summarized below:

(1) TG levels-Fig. S8A; Lines 316-321

Quantification using a commercial kit was consistent with lipid droplet staining: TG levels were significantly higher under high glucose (HG) and HG+Met conditions compared with low glucose (LG). In LG, metformin markedly reduced TG levels, whereas in HG, TG levels were comparable between PBS and Met groups.

(2) Lipogenesis-Fig. S8B; Lines 321-326

We examined FASN and p-ACC at 3 h, 12 h, and 24 h:

Under LG+Met conditions, p-ACC remained elevated throughout 3－24 h, and FASN expression markedly decreased at 24 h, indicating that de novo lipogenesis was persistently suppressed during the entire observation period.

Under HG+Met conditions, the increase in p-ACC at 3－12 h suggests a transient inhibition of lipogenesis; however, by 24 h, p-ACC levels returned close to baseline, and FASN showed only a slight decrease. In this context, the 24-h accumulation of lipid droplets is more consistent with enhanced uptake and storage of exogenous fatty acids rather than an increase in de novo fatty acid synthesis.

Therefore, we infer that lipogenic capacity remains continuously inhibited under LG+Met, whereas under HG+Met, lipogenesis is transiently suppressed and subsequently restored, with the 24-h lipid droplet accumulation mainly driven by fatty acid uptake and storage.

(3) AMPK phosphorylation-Fig. S8B; Lines 339-344

In LG, p-AMPK remained persistently elevated. In HG, metformin strongly activated p-AMPK at 3–12 h, but levels gradually declined by 24 h. This temporal pattern is consistent with lipid droplet mobilization/FAO compensation, suggesting that cells in LG remain in persistent energy crisis, while in HG they can partially alleviate

metformin-induced stress by enhancing fatty acid oxidation.

(4) In vivo lipid droplet monitoring-Fig. 4N, Fig. S8K; Lines 352-353

In tumor tissues (Fig. 4N), Oil Red O staining and quantification showed comparable lipid droplet levels between PBS and Met groups under a high-carbohydrate diet. Inhibition of ATGL led to marked lipid droplet accumulation, whereas DGAT1 inhibition significantly reduced lipid droplet content.

Comment 5：Figure 5: The authors demonstrate that metformin increases glycolytic flux in cancer cells, resulting in increased lactate production. The authors should measure lactate concentrations in tumor interstitial fluids to confirm this effect in vivo.

Response： To determine whether metformin similarly modulates lactate in vivo, we measured lactate in tumor interstitial fluid (TIF) from xenograft-bearing mice. Under LCD, metformin significantly decreased TIF lactate, whereas under HCD, TIF lactate was further elevated in metformin-treated mice relative to HCD controls—consistent with our in vitro findings. The data are shown in **Fig. S9A**, and the corresponding text has been updated at **Lines 373-375**.

Comment 6：Figure 6: Since lactate regulates CD36 expression through the transcription factor c-Jun, it would be interesting to determine if lactate also affects the phosphorylation of c-Jun. There are well-characterized JNK inhibitors; I suggest studying their effects in vitro and in vivo.

Response： We thank the reviewer for this valuable comment. In response, we evaluated c-JUN phosphorylation after lactate treatment. p-c-JUN mirrored total c-JUN dynamics, indicating lactate-induced c-JUN activation. The corresponding p-c-JUN bands have been added to the revised Western blots (**Fig. 6F, 6G, 6H, 6N, 6P; Lines 427-433**). Furthermore, we tested the functional role of c-JUN in metformin resistance by using the JNK inhibitor SP600125 and by modulating c-JUN expression in vitro (**Figs. S12B-S12E; Lines 436-439**) and in vivo (**Fig. S13; Lines 439-442**). The related results and descriptions have been updated in the revised manuscript.

Comment 7*：*Figure 7: Is there a correlation between LDHA expression and overall survival or progression-free survival in colorectal cancer? The authors should analyze several databases to reinforce their conclusion. Is there a correlation between LDHA expression and the response to anti-cancer treatment in clinical settings for colorectal cancer? The characterization of lactate levels, as well as CD36 and c-JUN expression, is missing in the in vivo experiments.

Response：Drawing on public datasets and prior studies, we found that high LDHA expression is closely associated with poor clinical outcomes in CRC. Patients with elevated LDHA showed significantly shorter overall survival (OS) (**Fig. S16A; Lines 499-501**), and multiple datasets also indicated a trend toward reduced progression-free survival (PFS) [1 – 3]. Numerous reports further link LDHA/LDH to therapeutic response. Histologically, high expression of LDH5—the predominant functional isoform of LDHA—correlates with lower chemotherapy response rates and shorter PFS, and can partially predict benefit from anti-angiogenic therapy [4]. Mechanistically, the METTL3 – LDHA axis drives 5-FU resistance via metabolic reprogramming in CRC models in vitro and in vivo [5], while the IGF2BP1 – LDHA axis stabilizes LDHA mRNA to enhance glycolysis and increase therapeutic tolerance; disrupting this pathway markedly improves drug sensitivity [6]. In addition, LDHA-mediated protein lactylation contributes to DNA repair and chemoresistance: LDHA-driven lactylation of XLF at K288 (via GCN5) augments NHEJ activity and confers chemoresistance, and inhibiting XLF-K288 lactylation synergizes with 5-FU in CRC PDX models [7] The related descriptions at **Lines 501-504** have been updated in the revised manuscript.

In the revised manuscript, we also include intratumoral lactate measurements and CD36/c-JUN expression analyses to corroborate concordance between in vivo and in vitro findings (**Fig. S19; Lines 538-541**).

1. Wang Y, Nie H, Liao Z, He X, Xu Z, Zhou J, et al. Expression and Clinical Significance of Lactate Dehydrogenase A in Colon Adenocarcinoma. Front Oncol. 2021;11:700795. PMID: 34307169.
2. Li G, Wang Z, Xu J, Wu H, Cai S, He Y. The prognostic value of lactate

dehydrogenase levels in colorectal cancer: a meta-analysis. BMC Cancer. 2016;16:249. PMID: 27016045.

3. Marmorino F, Salvatore L, Barbara C, et al. Serum LDH predicts benefit from bevacizumab beyond progression in metastatic colorectal cancer. Br J Cancer. 2017;116(3):318–323. PMID: 28081548.
4. Koukourakis MI, Giatromanolaki A, Sivridis E, et al. Prognostic and predictive role of lactate dehydrogenase 5 expression in colorectal cancer patients treated with PTK787/ZK 222584 (vatalanib) antiangiogenic therapy. Clin Cancer Res. 2011;17(14):4892–4900. PMID: 21632858.
5. Zhang K, Zhang T, Yang Y, et al. N6-methyladenosine-mediated LDHA induction potentiates chemoresistance of colorectal cancer cells through metabolic reprogramming. Theranostics. 2022;12(10):4802–4817. PMID: 35832094
6. Zhang XL, Li KJ, Feng JX, Liu GJ, Feng YL. Blocking the IGF2BP1-promoted glucose metabolism of colon cancer cells via direct de-stabilizing mRNA of the LDHA enhances anticancer effects. Mol Ther Nucleic Acids. 2021;23:835–846. PMID: 33614233.
7. Jin M, Li J, Zhou X, et al. Lactylation of XLF promotes non-homologous end-joining repair and chemoresistance in cancer. Mol Cell. 2025; Epub ahead of print. PMID: 40680721.

Minor Points

Comment 1：Typo: Line 222 contains a repetition of the word "proportion."

Response： We thank the reviewer for the careful reading. We have rechecked the revised manuscript and updated the text at **Lines 222-224** accordingly.

Comment 2：Typo: Line 291 should correctly state "Lipid Droplets (LD) and low glucose (LG)."

Response: To avoid confusion, we have standardized abbreviations throughout the manuscript: LG/HG denote low- and high-glucose cell-culture conditions; LCD/HCD denote low- and high-carbohydrate diet conditions in animal studies; and LD refers to

lipid droplet. The corresponding text at **Line 303-305** has been revised, and the main text, figure labels, and legends have been updated consistently across the manuscript.

<Comments from Reviewer #3>

Major Points:

Comment 1: The authors propose that LDHA-driven metformin-resistance is associated with increased FA uptake and storage. However, it is unclear if this is due to the FAs being utilized for energy or if the FAs are stored (lipid droplets) to act as a buffer for ROS (eg. PMIDs: 25594180, 30400205)

a. What is the main mechanism (storage or utilization of FAs) of LDHA driven metformin-resistance associated with this phenotype?

b. Does FA oxidation increase in the metformin-resistance condition?

c. If this is associated with FA oxidation for ATP/NADPH production, how does this occur under conditions of ETC impairment (metformin)?

d. Can the LDHA-high phenotype (Figure 7) be reversed by blocking FA uptake (CD36i－SMS121) or FA oxidation (Etomoxir)? Or can the LDHA-low phenotype be reversed by reducing ROS, e.g. N-acetyl-cysteine?

Response: We sincerely thank the reviewer for these insightful and constructive comments. We have performed a series of additional experiments to clarify the relationship between fatty acid (FA) metabolism, LDHA-driven metformin resistance, and mitochondrial compensation. The detailed results are summarized below.

(a－b) Regarding whether LDHA-driven metformin resistance primarily depends on FA storage or utilization, our results demonstrate that the core mechanism involves FA utilization (oxidation for energy production) rather than storage for buffering oxidative stress. Using a fatty acid oxidation (FAO) assay kit, we found that resistant cells exhibited significantly elevated FAO levels. Meanwhile, the FA peroxidation levels showed no significant difference among various cell groups, including L, L+M, H, H+M, LDHA-low, LDHA-low+Met, LDHA-high, and LDHA-high+Met. (**Fig S8C, S8G, line 329-334; Fig S16B, line 520-521; Fig S16E, line 524**). These findings indicate that resistant cells do not rely on FA storage in lipid droplets to mitigate ROS, but instead enhance FA oxidation to generate energy and reducing power to sustain metabolic balance.

(c) In **Figure S8E**, we used the ATGL inhibitor (ATGLi) to block lipid droplet

breakdown and observed that NADPH levels markedly decreased, whereas apoptosis increased in resistant cells (Fig. 4H, 4I). This suggests that FAO is crucial for maintaining both energy metabolism and redox balance in resistant cells.

Further transmission electron microscopy (TEM) and Seahorse analyses showed that metformin-resistant or LDHA-overexpressing cells contained increased numbers of mitochondria with dense and intact cristae structures. Long-chain FAO stress testing further confirmed that these cells retained FA oxidation capacity. Despite metformin-induced inhibition of the electron transport chain (ETC), resistant cells could maintain ATP production through compensatory mitochondrial biogenesis and functional remodeling (**Fig S8I, S8J, Line 344-347**; **Fig S16C, S16D, Line 521-524**).

(d) To further address the reviewer' request for intervention validation, we treated LDHA-overexpressing cells with the CD36 inhibitor (SMS121) and the FAO inhibitor (Etomoxir). Both treatments significantly reversed the metformin-resistant phenotype induced by LDHA overexpression. Conversely, supplementation with N-acetyl-cysteine (NAC) in LDHA-knockdown cells partially alleviated metformin-induced cell death (**Fig S18, Line 528-533**). Together, these findings demonstrate that LDHA promotes FA uptake and oxidation to maintain energy homeostasis and antioxidant capacity, thereby driving cancer stem cell resistance to metformin.

Comment 2: Lactate plays an important role in maintaining the NAD+/NADH ratio under reduced ETC function. Have the authors assessed whether it is the maintenance of this redox balance that is driving metformin-resistance? Can alpha-ketobutyrate/LbNox also induce metformin resistance?

Response: We thank the reviewer for this valuable comment. To determine whether metformin resistance is driven by lactate-mediated maintenance of the NAD^+/NADH ratio, we measured NAD^+/NADH levels across multiple models. The results showed that metformin treatment markedly decreased the NAD^+/NADH ratio under low-glucose (LG) conditions and in LDHA-low cells, consistent with the inhibition of mitochondrial electron transport chain (ETC) function. In contrast, under high-glucose (HG) conditions and in LDHA-overexpressing cells, the NAD^+/NADH ratio was

partially restored; however, this restoration could not account for the resistant phenotype.

Furthermore, we supplemented cells with exogenous lactate (Lac) or α-ketobutyrate (α-KB) to modulate the NAD^+/NADH balance. The results showed that α-KB partially restored the NAD^+/NADH ratio but failed to improve cell viability or reduce metformin-induced apoptosis, suggesting that restoration of the NAD^+/NADH ratio is not the key determinant of resistance. In contrast, lactate supplementation did not significantly alter the NAD^+/NADH ratio but markedly increased the IC_{50} and reduced apoptosis, indicating that lactate-induced resistance is not dependent on the maintenance of NAD^+/NADH balance.

These updates have been incorporated into the Results at **Lines 479–485 and 524–526**, with supporting data provided in **Supplementary Fig. S15A-S15C and Fig. S17 A-S17**C.

Comment 3: Is lactylation the main driver of the c-Jun/CD36 expression? The phenotypes could be equally explained by the expression of c-Jun being driven by increased histone acetylation or even other non-canonical acylations. Ascribing the effects to histone lactylation specifically seems an overreach without mechanistic experiments showing that lactylation is both necessary and sufficient for the c-Jun expression changes.

Response: We agree that the regulation of c-JUN/CD36 expression may be influenced by multiple epigenetic modification pathways, including histone acetylation and other non-canonical acylations. In this study, we primarily focused on the role of histone lactylation in this process, and our ChIP-qPCR and functional intervention experiments demonstrated a direct association between histone lactylation and the transcriptional activity of the c-JUN promoter, supporting its critical role in activating the c-JUN/CD36 axis.

However, we also recognize that histone lactylation may not be the only regulatory factor involved. Other epigenetic modifications (e.g., acetylation, butyrylation) and

metabolic signaling pathways may also modulate the c-JUN/CD36 axis and contribute to metformin resistance. While our data support a critical role for lactylation, we do not exclude additional mechanisms. We will investigate these pathways in future work, and have added corresponding statements to the revised Discussion at **Lines 634-640** to provide a more comprehensive interpretation of lactylation's role in this process.

Comment 4: It is not entirely clear that the high lactate phenotypes are driven by lactylation rather than by oxidation of lactate. The authors could assess the contributions of glucose derived-lactate/acetyl-CoA to c-Jun expression through glucose fate tracing experiments in the LG/HG conditions. In addition, if the phenotypes are mediated by histone lactylation, inhibiting the entry of lactate and/or lactate-derived pyruvate into the mitochondria should have little effect; in fact, they may even phenocopy metformin resistance. The authors should show this with inhibition or knock-out of the mitochondrial pyruvate carrier, for examplet.

Response: We fully understand the reviewer's key point that the high-lactate phenotype might result from lactate oxidation metabolism—that is, lactate entering mitochondria, being converted to pyruvate, and thereby participating in the NAD^+/NADH redox cycle. To verify this possibility, we conducted a series of functional validation experiments described in Comment 2, and our results demonstrated that the maintenance of the NAD^+/NADH balance by lactate is not the primary mechanism driving this phenotype. Furthermore, following the reviewer's suggestion, we employed the mitochondrial pyruvate carrier (MPC) inhibitor UK5099 to block the mitochondrial oxidation of lactate and lactate-derived pyruvate. As shown in our newly added data (**Figs. S15D–S15E and S17D–S17E**), MPC inhibition did not significantly affect IC_{50} values or apoptosis under either LG or HG conditions, nor in LDHA-high or LDHA-low states. Importantly, lactate supplementation still enhanced cellular resistance. These findings align with the reviewer' s prediction: if histone lactylation is the dominant mechanism, blocking mitochondrial lactate import should not alter the phenotype. The corresponding rationale and data have been added to the revised Discussion at **Lines 486-487 and Lines 527-528**. Taken together, our results support the conclusion that the

activation of the c-JUN/CD36 pathway and metformin resistance are primarily driven by histone lactylation, rather than by lactate oxidation or NAD^+/NADH redox maintenance.

We fully agree that glucose metabolic flux tracing would further clarify the dynamic contribution of the glucose－lactate pathway. However, our laboratory currently lacks stable isotope tracing capability; we plan to incorporate these analyses in future studies. The revised Discussion reflects this at **Lines 632-634**.

Minor Points:

Comment 1: Fig 1F,G,Q－ the HD effect seems limited to HCT116, not really seen in SW620 (even in HD conditions: tumor size decreases, cleaved casp3 increases, Ki67 decreases). Authors should provide explanation or show models that phenocopy the effects seen in HCT116.

Response: We understand the reviewer's concern. Flow cytometry and immunofluorescence analyses of tumor tissues **(Fig. 1N, 1O)** revealed that HCT116 tumors consist almost entirely of $CD133^+$ cells, whereas SW620 tumors contain a markedly smaller proportion of $CD133^+$ cells, being predominantly composed of $CD133^-$ bulk populations. Our conclusions mainly focus on $CD133^+$ colorectal cancer stem cells (CSCs), which exhibit pronounced metformin resistance under high-glucose conditions. Therefore, in the SW620 model—dominated by $CD133^-$ bulk tumor cells—metformin still suppresses tumor growth and induces apoptosis even under high-glucose conditions, as reflected by decreased tumor volume, increased cleaved caspase-3, and reduced Ki67 expression.

These findings further support our central hypothesis that metformin resistance is primarily mediated by $CD133^+$ CSCs rather than representing a general property of all tumor cells. Explanatory text has been added to the revised Results at **Lines 230－239**.

Comment 2: Figure 1P-S, S1: why does Ki67 (marker of proliferation) reduce/not change, but CD133 increases are these CD133+ cells still proliferating normally? Is the CD133+ population due to de-differentiating to CSC fate, or increased

proliferation of CD133+ cells?

Comment 3: Lines 229-230. Figure 2I, J: Why does PDO size decrease but number of CD133+ cells increase? How rapidly do these cells proliferate? Is there a difference in cell volume that contributes to reduced PDO size?

Comment 4: Paragraph 231-236; Figure S2G do the authors sort CD133+ cells to assess colony forming capacity?

Response: We thank the reviewer for these insightful questions. To clarify the observation that Ki67 expression decreases or remains unchanged while the proportion of $CD133^+$ cells increase in tumor tissues, we sorted $CD133^+$ and $CD133^–$ subpopulations and analyzed their apoptotic responses **(Fig. 2J-2K; Fig. S3I-2J).** The results showed that metformin markedly induced apoptosis in $CD133^–$ cells under both high- and low-glucose conditions, whereas $CD133^+$ cells exhibited clear resistance to metformin under high-glucose conditions.

Accordingly, in HCT116 tumors under HCD—which consist almost entirely of $CD133^+$ cells—metformin treatment resulted in unchanged Ki67 expression but a further increase in $CD133^+$ cell proportion **(Fig. 1L, 1N)**. In contrast, SW620 tumors, which contain a much lower proportion of $CD133^+$ cells and are predominantly composed of $CD133^–$ cells, still underwent metformin-induced apoptosis under HCD conditions, leading to an overall reduction in tumor volume while the remaining cells became relatively enriched in $CD133^+$ cells.

Regarding the proliferative status of $CD133^+$ cells, we observed that Ki67 remained highly expressed in HCT116 tumor tissues, comparable to control groups, suggesting that these $CD133^+$ CSCs maintained active proliferation **(Fig. 1L, 1N)**. Similarly, in PDOs, $CD133^+$ cells retained high Ki67 expression under high-glucose plus metformin treatment, whereas Ki67 expression was markedly reduced under low-glucose plus metformin conditions (**Fig. S4A, S4B; Lines 253-256**).

HE staining and cell diameter measurements further confirmed that metformin did not affect cell size, indicating that the reduced PDO volume resulted from a decrease in cell number rather than cell shrinkage (**Fig. S4C, S4D; Lines 256-259**).

Moreover, $CD133^+$ cells were first isolated by magnetic sorting and then treated with

metformin under both glucose conditions to assess their clonogenic potential. The results showed that metformin markedly suppressed the colony-forming ability of $CD133^{+}$ CSCs under low-glucose conditions, whereas this inhibitory effect was largely abolished under high-glucose conditionss (**Fig. S3G; Lines 240=242**).

Taken together, these findings indicate that the increase in $CD133^{+}$ CSC proportion primarily results from metabolic adaptation and selective survival, rather than from de-differentiation or abnormal proliferation. Whether a subset of $CD133^{-}$ cells can convert into CSCs remains an open question that will be further investigated in our future studies. We have added corresponding clarifications to the Discussion sections of the revised manuscript (**Line 632-634**)

Comment 5: Lines 236-238: Magnetic sorting for CD133+ clarify if metformin treatment is after sorting-how long are the cells treated here?

Response: In our experiments, $CD133^{+}$ and $CD133^{-}$ cells were first isolated by magnetic sorting, then treated with metformin for 24 h under either high- or low-glucose conditions prior to functional assays. Sphere-formation assays were maintained for 1–2 weeks, and colony-formation assays for 7 – 10 days. We have clarified these procedures in the revised Methods at **Lines 762-775**.

Comment 6: Line 240-241, Figure 2: In SW620 the CD133+ cells, AnnexinV increases in both HG and LG condition but not in CD133- as stated (cytometry plots and panels for HCT15 showed correct observations)-perhaps quantification panels are swapped in Figure 2L/M?

Response: We thank the reviewer for carefully identifying this error. The reviewer is correct-the quantification panels for Figure 2L and Figure 2M (SW620) were inadvertently swapped in the original submission. We have carefully rechecked the raw flow cytometry data and corrected the figure in the revised version.

Comment 7: Lines 253-257, Fig 3G-K: do the authors experimentally confirm 24h is not sufficient for Annexin V to increase?

Response: The metformin concentration and treatment duration used in our metabolomic experiments were determined based on the 24-hour IC_{50} curves. We selected 10 mM metformin for 24 hours, a dose markedly below the 24-hour IC_{50} values for the three colorectal cancer cell lines (HCT116 = 17.1 mM; HCT15 = 40.9 mM; SW620 = 56.1 mM; see figure below). This experimental design aimed to capture early metabolic alterations prior to extensive apoptosis (**Line 271-272**).

In addition, a time-course experiment under high-glucose conditions using the same dose was performed. The results showed that in HCT116 cells, Annexin V^+ remained unchanged at 24 h but significantly increased at 48 h, which may be attributed to increased glucose consumption in the culture medium, leading to reduced resistance of the cells.

Collectively, these findings support the rationale for performing metabolomic analysis at 24 h, indicating that the observed metabolic alterations represent metformin-induced metabolic reprogramming rather than secondary effects caused by large-scale cell death.

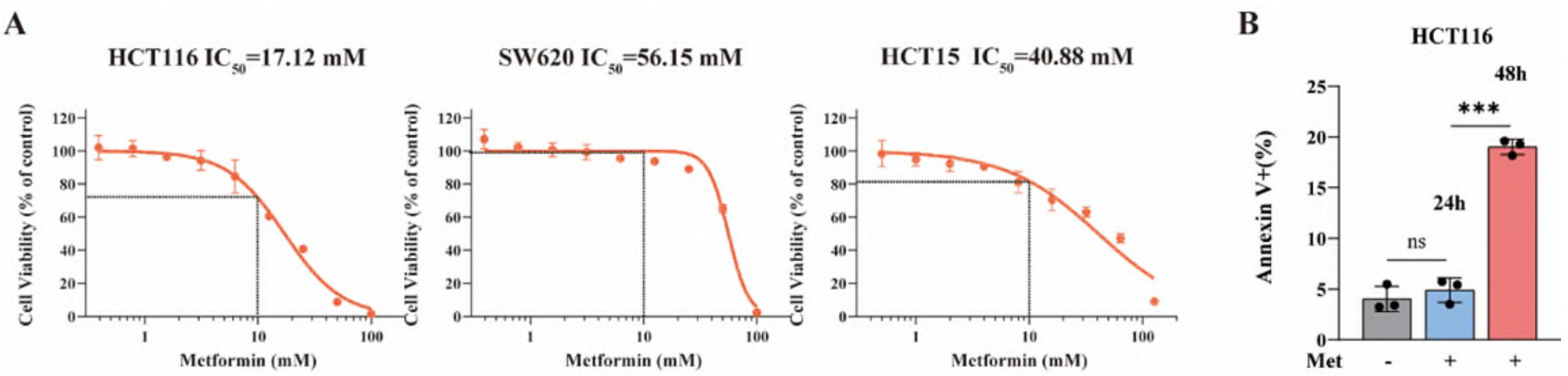

Comment 8: Lines 347-349 – higher ECAR (Figure 5A,B) should imply media acidification and higher lactate levels (same time point for lactate assay + Seahorse ECAR i.e. 24 hours post metformin treatment). Line 352-353 – is the data normalised to cell number or content to compensate for these differences?

Response: We thank the reviewer for this important comment. ECAR measurements (Fig. 5A, 5B) and lactate quantification (Fig. 5F) were both performed 24 hours after metformin treatment, ensuring that the two datasets correspond to the same experimental time point. To correct for potential differences in cell number, we proceeded as follows:

ECAR: Immediately after the Seahorse run, we performed CCK-8 viability

measurements in the same wells, with absorbance read on a BioTek Cytation 5 plate reader, and normalized ECAR values to viability.

Extracellular (medium) lactate: Lactate was measured using a commercial kit, and CCK-8 was performed in the same wells (BioTek Cytation 5) to enable viability normalization of lactate values.

Intracellular lactate: We started each assay with an equal number of cells per condition prior to extraction, thereby controlling for cell-number differences at the outset.

These normalization and equal-input controls ensure that the observed metabolic differences reflect true treatment effects, rather than cell-density variation. We have clarified these points in the revised Methods at **Line 418-420**.

Comment 9: Line 395, Figure S9-this experimental set up (mRNA + protein stability) does not look at the effect of lactate but rather metformin.

Response: We thank the reviewer for this valuable comment. To further confirm the role of lactate in this process, we have added experiments involving exogenous lactate treatment in the revised manuscript to evaluate its effects on the mRNA stability and protein degradation of c-JUN and CD36. The results showed that lactate treatment yielded findings consistent with those of metformin, with no significant changes observed in mRNA decay rates or protein stability (**Fig. S9A-S9E**). We have clarified this point in the revised Results at **Line 418-420 and Line 457-458**.

Typos/Errors:

1. Figure 2 L, M-incorrect panel quantification (see minor comment #6)

Response: We have corrected this.

2. Line 272-Fig S4A?

Response: This was an error in the original manuscript. In the prior version, the correct callout should indeed have been Fig. S4A. In the revised manuscript, after figure renumbering, the corresponding panel is now **Fig. S6A**. We have updated the text at **Line 285** accordingly.

3. Line 276-typos: (CD6)

Response: We have corrected this to CD36 at **Line 291**

4. Line 276-Fig S4B, C?

Response: We agree. In the prior version, the correct callouts should indeed have been Figs. S4B, S4C. In the revised manuscript, after figure renumbering, the corresponding panels are now Figs. S6D, S6E. We have updated the text at **Line 291** accordingly.

5. Line 292; use different acronyms; LD=low carb diet + LD=lipid droplets?

Response: To avoid confusion, we have standardized abbreviations throughout the manuscript: LG/HG denote low- and high-glucose cell-culture conditions; LCD/HCD denote low- and high-carbohydrate diet conditions in animal studies; and LD refers to lipid droplet. The corresponding text at **Line 303-305** has been revised, and the main text, figure labels, and legends have been updated consistently across the manuscript.

6. Fig4 L, M: labels wrong-no LG condition but shown in quantification.

Response: Response: You are correct—there was no LG condition. We have removed LG from the labels/quantification, and the corrected panels are now **Fig. 4H–4I**.

8. Fig5 I, J: Missing label and information for metformin in quantification panels

Response: We have added the missing metformin labels and related information to the quantification panels of Fig. 5I－5J, and updated the figure

9. Fig 6D-labels wrong?

Response: This was a labeling mistake. It has been corrected to“HG-PBS vs HG-Met”.

10. Fig 6N-missing a + sign?

Response: We have added the missing “+” in Fig. 6N and updated the figure

Dear Editors and Reviewer #3,

We sincerely thank Reviewer #3 for the continued careful evaluation of our manuscript and for the insightful comments provided during the previous round of review.

We acknowledge that several key concerns were not fully resolved in the first revision. In the current revision, we have therefore re-examined these issues in depth and conducted additional experiments to more rigorously address the reviewer's major concerns, particularly regarding whether histone lactylation represents a causal regulatory mechanism rather than a metabolic byproduct.

We believe that these new data substantially strengthen the mechanistic conclusions of our study and clarify the points that previously remained uncertain.

Below, we provide detailed, point-by-point responses to all comments. All modifications have been shown in **red and bold font** in the manuscript.

Sincerely,

Han Ping Shi, MD, PhD, FACS

<Comments from Reviewer #3>

In the revised manuscript, the authors have addressed many of the prior concerns. However, my primary concern remains. I do not think the authors have ruled out that lactylation is simply a passenger phenomenon mediated by the high levels of lactate in the high glucose and high glucose metformin conditions. That is, c-JUN activation and CD36 upregulation could be mediated through canonical p300-dependent histone acetylation and histone-lactylation could be simply correlated because of its high levels.

Response: Thank you for the reviewer' s careful evaluation of our revised manuscript and for acknowledging the improvements made. Regarding the central concern—that the histone lactylation observed under high-glucose and high-glucose plus metformin conditions may merely be a "passenger" phenomenon reflecting elevated lactate, while c-JUN activation and CD36 upregulation could instead be driven by canonical p300-dependent histone acetylation-we have added a series of additional experiments in the revision to further test a causal role for lactylation in activating the c-JUN/CD36 axis and promoting the resistant phenotype, rather than a simple correlation. Our new data support that lactate/lactylation is not a passive marker; instead, H3K18 lactylation promotes c-JUN transcription, thereby activating the c-JUN/CD36 axis and driving downstream lipid metabolic remodeling and metformin resistance. In contrast, increasing or altering histone acetylation does not recapitulate these effects, and the key phenotypes show a specific dependence on lactylation perturbation.

Below, we provide a point-by-point response to the reviewer' s major and minor comments and indicate the corresponding new experiments and figures where appropriate.

Major Points:

Comment 1: 1. In S17 and S18, the authors show that 'Pharmacologic inhibition of fatty-acid uptake with SMS121 or blockade of FAO with etomoxir partially reversed metformin resistance and increased apoptosis, indicating that LDHA-driven resistance relies on FAO-mediated metabolic compensation. Given that the title of the paper calls on histone lactylation and a c-JUN/CD36 axis, the authors need to show

that these effects (fatty acid update and fatty acid oxidation) occur downstream of lactylation-induced, c-JUN mediated CD36 expression.

Response: Thank you for raising this important point. To address the concern of whether fatty-acid uptake and FAO indeed occurs downstream of lactylation-driven, c-JUN-mediated CD36 upregulation, we added additional experiments in the revised manuscript (Fig. S17) to clarify the pathway hierarchy. We found that inhibition of histone lactylation with C646 markedly reduced FAO activity and lipid droplet accumulation, indicating that lactylation is required for this lipid metabolic phenotype. Importantly, even under lactylation blockade, enforced expression of c-JUN or CD36 could at least partially rescue FAO and lipid droplet accumulation, whereas knockdown of c-JUN or CD36 phenocopied lactylation inhibition and rendered exogenous lactate unable to promote FAO or lipid droplet accumulation. Collectively, these results place fatty-acid uptake and FAO as downstream metabolic outputs of lactylation-driven activation of the c-JUN/CD36 axis. The corresponding description has been added to Lines 492－499.

Comment 2-4: 2. If fatty acid oxidation is enabling ATP and NADPH synthesis in high-glucose/metformin conditions, shouldn’t the size of the lipid droplets or TG levels? However, the authors show no change in lipid content (LD size or TG levels) in the Metformin treated groups in HG.

3. The authors claim that cJUN, phospho cJUN and CD36 are maximally activated (saturated) in high glucose conditions (Figure 6A-B) and thus metformin cannot further increase cJUN activity and CD36 expression. Nonetheless, since the purported mechanism for resistance is increased lactate leading to cJUN lactylation, why do the authors not see high levels of lactylation at the cJUN promoter (Figure 6Q)? This seems contrary to lactate being the mediator of resistance and something else leading to increased cJUN activation and CD36 expression.

4. Given that HG+PBS and HG+Met have similar levels of TG, LD size, and FAO, how do the authors link this with their findings that lactate (higher in HG+Met, Figure 6Q, 5F) drives the FA uptake via cJUN/CD36 to mediate Met resistance?

Response: We thank the reviewer for the critical question regarding why the endpoint readouts of TG content, lipid droplet (LD) accumulation/size, FAO activity, and c-JUN/CD36 expression are similar between HG+PBS and HG+Met. Our newly added dose–response and time-course experiments (Supplementary Fig. 20) indicate that this reflects a saturable (plateau) behavior of downstream transcriptional and lipid-metabolic outputs under HG conditions, rather than arguing against a role for lactate/lactylation in resistance. Specifically, the time-course data show that under HG, TG, LD accumulation, FAO, and c-JUN/CD36 changes approach a plateau at ~18 h. In contrast, under HG+Met, metformin-induced metabolic reprogramming rapidly increases lactate accumulation, thereby accelerating the establishment of these downstream outputs so that they reach a plateau earlier (~6–12 h). Accordingly, at the 24 h endpoint used in this study, the lack of an obvious difference between HG and HG+Met in these readouts is consistent with the expected plateau kinetics. This interpretation is also in line with the endpoint metabolomics analysis in Fig. 3, in which fatty-acid–related metabolic readouts converge between HG and HG+Met once the resistant state is established, suggesting that both conditions have reached a similar metabolically adapted steady state.

Notably, we observed distinct temporal dynamics between histone lactylation and downstream phenotypes: pan-Kla/H3K18la continues to increase with prolonged metformin exposure, whereas c-JUN/CD36 and lipid-metabolic outputs plateau earlier. To distinguish correlation from causality, we performed additional perturbation experiments. Pharmacological inhibition of histone lactylation with C646 significantly suppressed TG levels, LD accumulation/size, FAO, and c-JUN/CD36 expression (Supplementary Fig. 20), indicating that lactylation is not merely a bystander but actively sustains the adaptive metabolic program and the resistant phenotype. Together with additional evidence in Fig. 7 and Supplementary Figs. 15–20 (including lactylation-dependent promotion of c-JUN transcription, activation of the c-JUN/CD36 axis, and epistasis/rescue experiments), these data support that lipid-metabolic remodeling represents a downstream output of lactylation-driven activation of the c-JUN/CD36 axis rather than an unrelated accompanying change. **The corresponding**

description has been added to Lines 510–530.

Collectively, our new data explain the convergence of HG and HG+Met endpoint readouts at 24 h and further support the mechanistic framework in which lactate/histone lactylation drives c-JUN/CD36 activation, lipid-metabolic reprogramming, and metformin resistance.

Comment 5: Lines 488-492, the authors claim that 'Together, these results demonstrate that lactate-induced metformin resistance occurs independently of lactate oxidation and NAD^+/NADH homeostasis, and instead primarily relies on lactate-driven epigenetic regulation-specifically, H3K18 lactylation, that promotes c-JUN transcription and consequently upregulates CD36 expression.' This is not concluded by the data. The data only shows that histone lactylation is happening at these sites in a manner that correlates with high glucose and lactate (with or without metformin). No causative value can be ascribed to the lactylation based on these results.

Response: In the revised manuscript, we added multiple sets of functional perturbation and rescue/blockade experiments. Collectively, these data support that H3K18 lactylation is not merely a passive bystander correlated with elevated lactate, but rather contributes to c-JUN transcriptional activation and CD36 upregulation, thereby promoting lipid metabolic remodeling and enhancing metformin tolerance in $CD133^+$ colorectal CSCs. The newly added evidence is summarized as follows:

1) Causal contribution of lactylation to the resistant phenotype (Figure 7; Supplementary Figure 15)

We first tested whether lactylation is required for the development of resistance by directly inhibiting lactylation. Under HG conditions, pharmacological inhibition of lactylation with the p300 inhibitor C646 significantly attenuated metformin tolerance and increased apoptosis in $CD133^+$ CSCs. Under LG conditions, exogenous lactate (Lac) induced a resistant phenotype, which was effectively reversed upon C646 treatment (Fig. 7C–7F; Fig. S15A–S15D). These functional results indicate that modulating lactylation levels markedly alters cellular responses to Met, supporting an essential role

for lactylation in the resistance phenotype.

2) Evidence that lactylation acts upstream of the c-JUN/CD36 axis (Figure 7; Supplementary Figure 16)

Mechanistically, we further positioned lactylation relative to the c-JUN/CD36 cascade. Inhibition of lactylation concomitantly reduced c-JUN, p-c-JUN, and CD36 expression (Fig. 7G). Under HG conditions, LDHA knockdown decreased endogenous lactate production and lactylation, leading to suppression of c-JUN/CD36. Supplementation with exogenous Lac restored lactylation and rescued c-JUN/CD36 expression; however, this rescue was abolished when lactylation was re-inhibited (Fig. 7H), supporting a causal sequence of Lac → lactylation → c-JUN/CD36. Moreover, JNK inhibition or c-JUN knockdown blocked CD36 induction downstream of elevated lactylation, whereas c-JUN overexpression remained sufficient to drive CD36 expression even when lactylation was inhibited (Fig. S16A). In vivo experiments yielded consistent conclusions (Fig. S16B-S16E).

3) Fatty-acid uptake/FAO and lipid droplet accumulation occur downstream of the axis (Supplementary Figure 17)

To directly address whether the lipid metabolic effects indeed lie downstream of "lactylation-induced, c-JUN-mediated CD36 upregulation," we performed additional epistasis-style positioning experiments. Inhibition of lactylation significantly decreased FAO activity and lipid droplet accumulation. Importantly, enforced expression of c-JUN or CD36 could at least partially restore these lipid metabolic phenotypes despite lactylation blockade. Conversely, depletion of c-JUN or CD36 phenocopied lactylation inhibition, and exogenous Lac was no longer able to promote FAO or lipid droplet accumulation (Fig. S17). These data place fatty-acid uptake/FAO and lipid storage as downstream metabolic outputs of the lactylation → c-JUN/CD36 axis.

4) Excluding histone acetylation as the primary mediator (Supplementary Figure

18 – 19)

Given the alternative explanation that canonical p300-dependent acetylation might mediate c-JUN/CD36 activation while lactylation is merely correlative, we systematically evaluated the contribution of acetylation. H3K18ac did not exhibit dynamics consistent with resistance establishment across the tested conditions, and increasing acetylation with sodium acetate (NaAc) failed to phenocopy the effects of Lac/lactylation on c-JUN/CD36 and resistance-associated phenotypes. In contrast, H3K18la changes aligned more closely with functional phenotypes and axis activation (Fig. S18-S19), supporting lactylation rather than acetylation as the predominant epigenetic driver in this context.

In summary, integrating the newly added functional perturbation, rescue/blockade, and hierarchical positioning data, we conclude that H3K18 lactylation under HG promotes c-JUN transcription and activates the c-JUN/CD36 axis, which in turn drives lipid metabolic remodeling and enhances metformin resistance in $CD133^{+}$ colorectal CSCs. **The corresponding results and description have been incorporated into the Results section entitled "Lactate-driven histone lactylation promotes c-JUN transcription and confers metformin resistance."**

Minor points:

Comment 1: In Figure 2E, in the text the authors claim "In HCT116-identified by flow as CD133+-dominant-metformin under HG further enriched the highly CD133-expressing subset". However, looking at the figure, the cutoff for CD133 high seems arbitrary and the main effect of metformin seems to be that in LG the proportion of CD133- increases markedly. This is not seen in HG for the positive fraction though is still significantly elevated in the high though this increase is diminished.

Response: We thank the reviewer for highlighting the potential ambiguity in Fig. 2E regarding the CD133 high cutoff and our previous wording. We agree that the original description was not sufficiently comprehensive or precise. Accordingly, we have

revised the text and clarified the gating strategy in the revised manuscript. Accordingly, we have revised the text in the revised manuscript (**Lines 221 – 226**).

Specifically, flow-cytometric analyses of HCT116 cells show that under LG conditions, the predominant effect of Met is a marked increase in the $CD133^{-}$ population, accompanied by a decrease in the CD133 high subpopulation. In contrast, under HG conditions, the $CD133^{-}$ fraction remains largely unchanged, whereas the CD133 high subpopulation increases (Fig. 2E). Therefore, we revised the manuscript to avoid overemphasizing "enrichment of CD133 high under HG" alone and instead to accurately describe these glucose-dependent shifts in CD133 distribution.

In addition, to address concerns that the CD133 high threshold might be arbitrary, we re-analyzed the data using a standardized gating approach: CD133 high was defined as the top 5% of CD133 fluorescence intensity in the LG+PBS control group, and this single fixed threshold (i.e., the same gate) was then applied identically to all treatment groups to ensure comparability across conditions. The corresponding quantification and plots in Fig. 2E have been updated accordingly.

Comment 2: For Figures 2I-K and S2I-J, the percentages in the bar graphs do not add up to the percentages in the scatter plot. What is being plotted?

Response: Thank you for pointing out the potential concern regarding the percentages shown in Fig. 2I-K and the supplementary figures. We have carefully re-checked Fig. 2I-K and believe that there are no errors in the plotting or calculations.

In addition, we noted that the figure referred to as "Fig. S2I – J" should in fact be "Fig. S3I – J". In Fig. S3I-J, we acknowledge that the percentages in the bar graphs did not correspond properly to the quadrant percentages in the dot plots. We have now corrected the quantification and re-plotted the data, and the revised version has been replaced in the updated manuscript. We sincerely apologize for any confusion this may have caused.

Comment 3: For Supplementary Figure 4A and 4B, the authors should confirm that the Ki67 staining is specific. It is unusual for Ki67 to not be entirely localized to the nucleus.

Response: We agree that the Ki67 signal shown in Supplementary Fig. 4A–B was not fully consistent with the expected predominantly nuclear localization, raising concerns about staining specificity/quality. We have therefore repeated the Ki67 immunostaining with optimized conditions and appropriate controls, and replaced Supplementary Fig. 4A–B with the new representative images and updated quantification in the revised manuscript. We apologize for the confusion caused by the original images.

Comment 4: For Figure 3B, I would suggest using the same color scheme here; the current color choices for CD133+ and CD133- cells are confusing.

Response: As suggested, we have standardized the color scheme in Fig. 3B in the revised manuscript to ensure consistency with the rest of the manuscript and other related figures.

Comment 5: Line 291 CD36 should be CD36OE

Response: This has been corrected in the revised manuscript (**Line 288**).

Comment 6: The statement, "Importantly, metabolomics was performed 24h after metformin when CD133+ CSC viability under LG had not yet declined significantly (Fig. 2J), arguing against non-specific changes due to cell death." is misleading as cells could be on their way to cell death due to LG dependent changes but not yet sufficiently stressed to trigger Annexin V positivity.

Response: Thank you for raising this important point. We agree that the statement could be misleading. Therefore, we have removed this sentence from the revised manuscript

Comment 7. Line 352-353: In vivo data does not show FAO in S8K, only LD size.

Response: We thank the reviewer for catching this. We agree that in the previous version the in vivo panel in Fig. S8K only showed lipid droplet (LD) staining/quantification and did not directly present FAO. To address this, we have now added new in vivo FAO measurements as Fig. S8L. We apologize for the confusion.

Comment 8. Figure 4B. If CD36 upregulates linoleic acid production, why do the authors add LA to Figure 4B and 4C? What do these results look like without LA?

Response: Thank you for raising this point regarding the experimental design in Fig. 4B – 4C. We would like to clarify that CD36 is a canonical fatty-acid transporter whose primary function is to facilitate the uptake of exogenous free fatty acids. In this study, we have no evidence that CD36 promotes the production or biosynthesis of linoleic acid (LA). The enrichment of the LA metabolism pathway in the metabolomics analysis (Fig. 3) more likely reflects activation of lipid metabolic processes, rather than increased LA generation driven by CD36.

Accordingly, in the in vitro lipid droplet assays (Oil Red O staining), LA was supplemented in certain conditions not to “generate LA via CD36,” but to provide an available fatty-acid substrate and thereby amplify CD36-dependent differences in fatty-acid uptake, making lipid droplet accumulation more readily visualized and quantified under bright-field microscopy. In practice, without exogenous LA, the Oil Red O signal is very weak and red-stained lipid droplets are barely detectable in bright-field images (as shown in the images we additionally provided), which limits straightforward comparison across treatment groups.

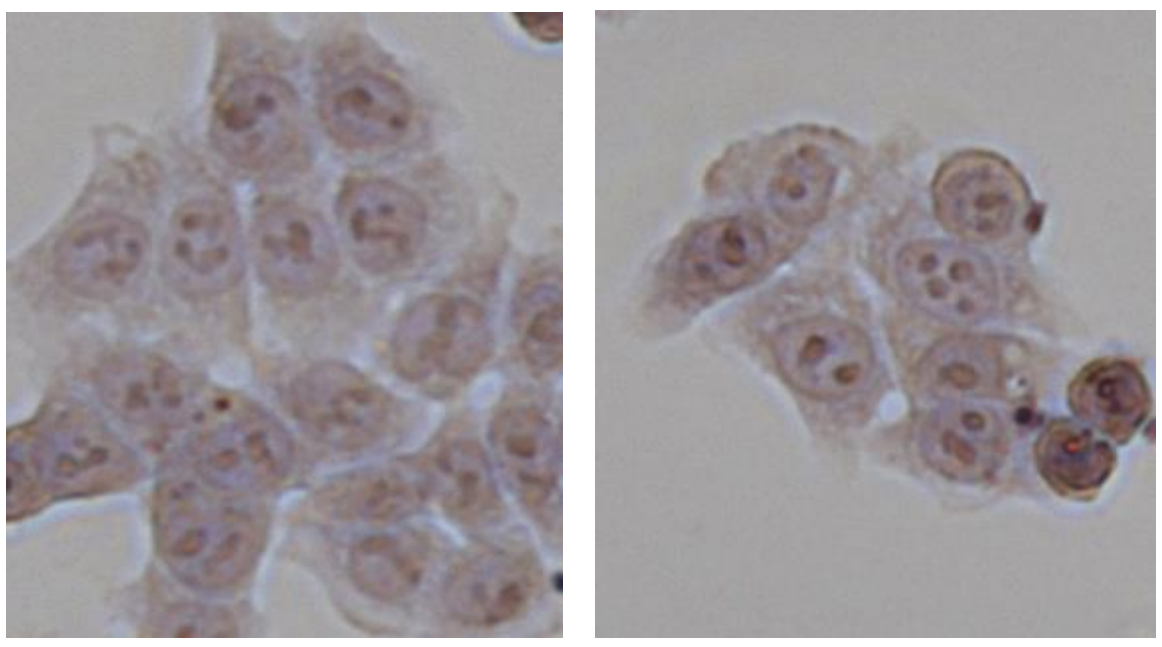

Comment 9. Figure 5AB. What do the authors mean by basal and compensatory glycolytic rates? These do not seem to be defined in the text.

Response: Thank you for pointing out that the terms "basal" and "compensatory" glycolytic rates were not explicitly defined in the original text. We agree that this clarification is important. Accordingly, we have revised the Methods section (Cell bioenergetic assays) to define these Seahorse-derived parameters and to describe how they were calculated **(Lines 880 – 888**). For ease of review, we also provide an explanatory schematic and brief definitions below.

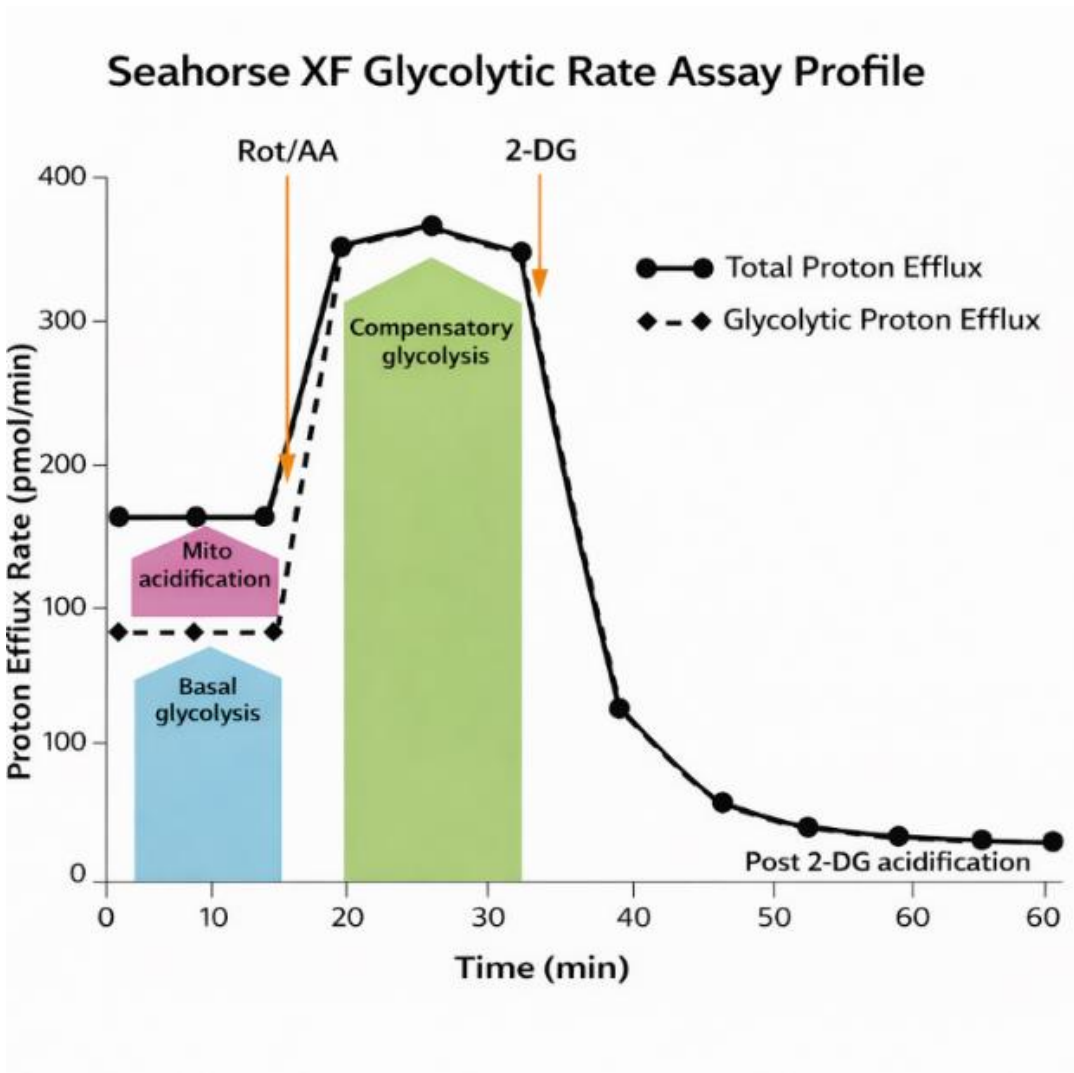

Basal glycolytic rate: The glycolytic flux (acidification rate attributable to glycolysis) under baseline conditions, before imposing mitochondrial stress. It reflects the extent to which cells rely on glycolysis for energy production at steady state. In the Seahorse Glycolysis Rate Assay, this is typically calculated from the glycolytic proton efflux rate (glycoPER) measured prior to the addition of rotenone/antimycin A (Rot/AA).

Compensatory glycolytic rate: The glycolytic rate achieved after mitochondrial respiration is inhibited (e.g., upon Rot/AA injection), when cells increase glycolysis to compensate for the loss of mitochondrial ATP production. It reflects the capacity of cells to upregulate glycolysis under respiratory constraint. In the Seahorse Glycolysis Rate Assay, this is typically calculated from glycoPER measured after Rot/AA addition.

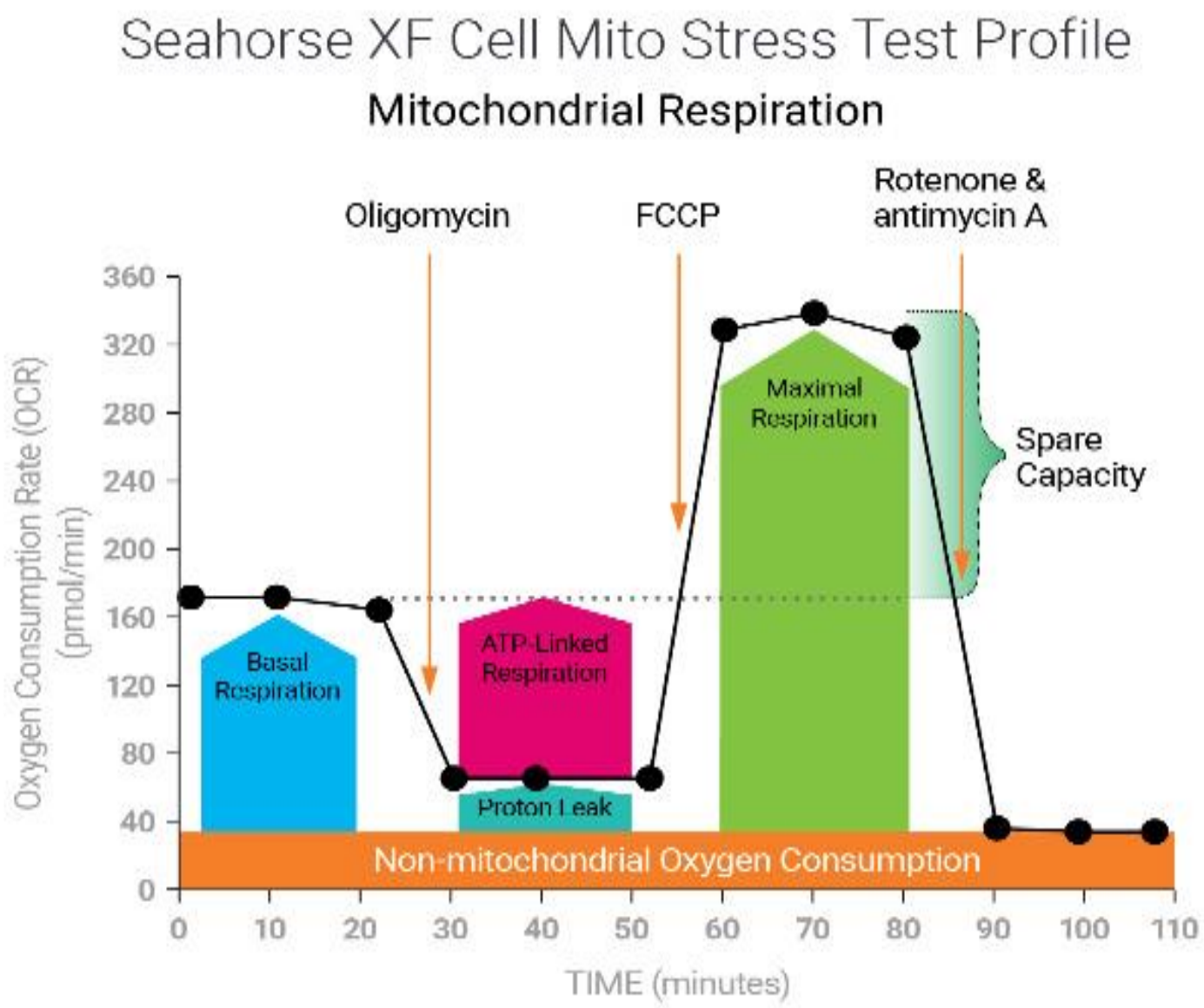

Basal respiration: the oxygen consumption used to meet cellular ATP demand and to offset mitochondrial proton leak, reflecting the cell's energy requirement under basal conditions.

Maximal respiration: the maximal oxygen consumption rate measured after addition of the uncoupler FCCP. By driving the electron transport chain to operate at its maximal capacity, FCCP mimics a physiological "energy demand," leading to rapid oxidation of substrates (glucose, lipids, and amino acids) to cope with this metabolic challenge, thereby indicating the cell's maximal respiratory capacity.

Comment 10. Line 411 LDHA should be siLDHA.

Response: We have corrected "LDHA" to "siLDHA" at **Line 409** in the revised manuscript.

Comment 11. As recommended, the authors have probed possible effects of lactate through NAD regeneration or oxidation in the mitochondria. The alpha-ketobutyrate experiments are conclusive, with aKB restoring NAD (SFig15A) and having no effect on apoptosis or viability. However, when the authors probe whether lactate can still have effects when prevented from entering mitochondria through MPC, there is no

control for whether at the doses used, UK5099 had any effect. Thus, the authors cannot conclude that the effects of lactate are not mediated by its oxidation.

Response: We thank the reviewer for highlighting the lack of an efficacy control for UK5099 in our previous submission. As recommended, we added a functional validation experiment in the revised manuscript. Using a Seahorse respiration assay with pyruvate as the sole carbon source, UK5099 markedly suppressed mitochondrial respiration in $CD133^{+}$ CRC stem cells. Compared with the control, UK5099 significantly reduced basal OCR, ATP-linked OCR, maximal respiratory capacity, and spare respiratory capacity (Fig. S21F), confirming that, at the concentration used in this study, UK5099 effectively blocks MPC-mediated pyruvate entry into mitochondria. We have revised the manuscript text accordingly at **Lines 537–540.**

Comment 12. In Figure 6N, CD36 expression is high in most HG conditions. Given that the HG, metformin treatment is still high in siLDHA-1 treated cells (though LDH levels are not shown), this suggests that the regulation of CD36 is not dependent on lactate levels.

Response: We agree that the original Fig. 6N did not clearly show the difference in CD36 under HG conditions. We therefore repeated the Western blot and replaced Fig. 6N with the updated data, in which the CD36 trend is now clearly distinguishable in siLDHA-1 – treated cells.

Comment 13. In Figure 6N-Q, do the levels of histone acetylation mirror those of lactylation and do the lactylated areas (by CUT&RUN) also show with an acetylation antibody?Figure 6D – are the labels still incorrect?

Response: Thank you for raising this important point. To address whether histone acetylation mirrors lactylation and whether the lactylated regions identified at the c-JUN locus are also enriched for acetylation, we systematically examined H3K18ac in parallel with H3K18la and additionally performed H3K18ac ChIP-seq focusing on the c-JUN promoter. We also confirmed and corrected the labeling issue in Figure 6D. Specifically:

(1) H3K18ac does not mirror the dynamics of histone lactylation.

In vitro, immunoblotting/IF analyses showed that H3K18ac does not display a pattern concordant with the robust increase in pan-Kla/H3K18la observed under HG ± Met conditions (Fig. S18A). Consistently, in vivo staining of tumor tissues further demonstrated that H3K18ac levels do not increase in parallel with the enhanced lactylation signals under HG/Met conditions (Fig. S19A).

(2) H3K18ac ChIP-seq does not show promoter enrichment corresponding to H3K18la at the c-JUN locus.

To directly test whether the lactylated regions are also detectable with an acetylation antibody, we performed H3K18ac ChIP-seq and analyzed the c-JUN promoter region in IGV. In contrast to the pronounced H3K18la enrichment at the c-JUN promoter in HG, metformin-resistant cells, the H3K18ac occupancy/peak profile at the same region did not show a corresponding increase (Fig. S18B).

(3) Figure 6D labeling.

We agree that the labels in Figure 6D were incorrect. This has been corrected in the revised manuscript.

Comment 14. Lines 315-317: "Blocking LD biogenesis markedly delayed or prevented resistance, whereas inhibitor withdrawal partially restored resistance with renewed lipid accumulation, indicating that LDs are critical for Met resistance in CD133+ CRC CSCs". This is a premature conclusion. At most, the authors can claim that DGAT1i is necessary and sufficient for lipid droplet formation and viability, but the role of lipid droplets is entirely unanswered by this data.

Response: We thank the reviewer for raising this important point. We agree that these data alone do not establish lipid droplets per se as the causal determinant of metformin resistance. Accordingly, we have revised the corresponding text to avoid overstating this conclusion (**Lines 315 – 316**).

Dear Editors and Reviewer #3,

We sincerely thank you for the continued evaluation of our manuscript and for the thoughtful and constructive comments provided during the previous round of review. We greatly appreciate the reviewer's careful assessment, which has been highly valuable in helping us further strengthen the rigor and clarity of our study.

In response to these comments, we have carefully re-examined the manuscript and carried out additional revisions and experiments to address the remaining concerns as thoroughly as possible. We believe that these new data and corresponding revisions have substantially strengthened the mechanistic basis of our study and have helped clarify the issues that were previously unresolved.

Below, we provide a detailed point-by-point response to all comments. All changes made in the revised manuscript are marked in **red and bold** for ease of reference.

Sincerely,

Han Ping Shi, MD, PhD, FACS

<Comments from Reviewer #3>

The authors have addressed most of my comments and concerns.

The experiments showing that acetate supplementation fails to rescue are an important addition. However, what is the dose of acetate used in the in vitro experiments? In vivo, the administration route and dose is very different (200 mM in drinking water for NaAc versus 1.68 g/kg, i.p., q2d NaLac). At least for the in vitro experiments, these doses should be equivalent and/or the authors should show that the acetate supplementation provided is sufficient to increase general histone acetylation but not to rescue the phenotype that is rescue by lactate.

Response: We thank the reviewer for this important and constructive comment. In our in vitro experiments, sodium acetate was administered at the same concentration and for the same duration as sodium lactate, specifically 10 mM for 24 hours. We have now clearly specified this information in the figure legends and the Methods section to improve clarity and consistency.

To further address the reviewer's concern, we performed additional validation experiments to determine whether acetate supplementation is sufficient to increase global histone acetylation while failing to reproduce the phenotype rescued by lactate. Specifically, in both HCT116 $CD133^{+}$ cells and SW620 $CD133^{+}$ cells, NaAc treatment significantly increased histone acetylation levels, but did not restore c-JUN/CD36 expression or reverse metformin sensitivity.

These results are now presented in **Fig. S18C** and **Fig. S19B－D**, and further support the conclusion that the effect of lactate cannot be recapitulated by acetate-driven histone acetylation.
